# HIV-1 Neutralizing Antibody Signatures and Application to Epitope-Targeted Vaccine Design

**DOI:** 10.1016/j.chom.2018.12.001

**Published:** 2019-01-09

**Authors:** Christine A. Bricault, Karina Yusim, Michael S. Seaman, Hyejin Yoon, James Theiler, Elena E. Giorgi, Kshitij Wagh, Maxwell Theiler, Peter Hraber, Jennifer P. Macke, Edward F. Kreider, Gerald H. Learn, Beatrice H. Hahn, Johannes F. Scheid, James M. Kovacs, Jennifer L. Shields, Christy L. Lavine, Fadi Ghantous, Michael Rist, Madeleine G. Bayne, George H. Neubauer, Katherine McMahan, Hanqin Peng, Coraline Chéneau, Jennifer J. Jones, Jie Zeng, Christina Ochsenbauer, Joseph P. Nkolola, Kathryn E. Stephenson, Bing Chen, S. Gnanakaran, Mattia Bonsignori, LaTonya D. Williams, Barton F. Haynes, Nicole Doria-Rose, John R. Mascola, David C. Montefiori, Dan H. Barouch, Bette Korber

**Affiliations:** 1 Center for Virology and Vaccine Research, Beth Israel Deaconess Medical Center, Boston, MA 02215, USA; 2 Los Alamos National Laboratory, Los Alamos, NM 87545, USA; 3 New Mexico Consortium, Los Alamos, NM 87545, USA; 4 Departments of Medicine and Microbiology, Perelman School of Medicine, University of Pennsylvania, Philadelphia, PA 19104, USA; 5 Massachusetts General Hospital, Boston, MA 02114, USA; 6 Harvard Medical School, Boston, MA 02114, USA; 7 Division of Molecular Medicine, Children’s Hospital, Boston, MA 02115, USA; 8 Department of Pediatrics, Harvard Medical School, Boston, MA 02115, USA; 9 Departments of Chemistry and Biochemistry, University of Colorado, Colorado Springs, CO 80918, USA; 10 Department of Medicine and CFAR, University of Alabama at Birmingham, Birmingham, AL 35294, USA; 11 Ragon Institute of Massachusetts General Hospital, MIT, and Harvard, Boston, MA 02114, USA; 12 Duke Human Vaccine Institute, Duke University School of Medicine, Durham, NC 27710, USA; 13 Department of Medicine, Duke University School of Medicine, Durham, NC 27710, USA; 14 Department of Immunology, Duke University School of Medicine, Durham, NC 27710, USA; 15 Vaccine Research Center, National Institute of Allergy and Infectious Diseases, National Institutes of Health, Bethesda, MD 20814, USA; 16 Department of Surgery, Duke University School of Medicine, Durham, NC 27710, USA

**Keywords:** HIV-1, broadly neutralizing antibodies, signature analysis, machine learning, vaccine design, V2-apex antibodies, hypervariable regions

## Abstract

Eliciting HIV-1-specific broadly neutralizing antibodies (bNAbs) remains a challenge for vaccine development, and the potential of passively delivered bNAbs for prophylaxis and therapeutics is being explored. We used neutralization data from four large virus panels to comprehensively map viral signatures associated with bNAb sensitivity, including amino acids, hypervariable region characteristics, and clade effects across four different classes of bNAbs. The bNAb signatures defined for the variable loop 2 (V2) epitope region of HIV-1 Env were then employed to inform immunogen design in a proof-of-concept exploration of signature-based epitope targeted (SET) vaccines. V2 bNAb signature-guided mutations were introduced into Env 459C to create a trivalent vaccine, and immunization of guinea pigs with V2-SET vaccines resulted in increased breadth of NAb responses compared with Env 459C alone. These data demonstrate that bNAb signatures can be utilized to engineer HIV-1 Env vaccine immunogens capable of eliciting antibody responses with greater neutralization breadth.

## Highlights

• HIV-1 bNAb sensitivity signatures from 4 large virus panels mapped across 4 Ab classes
• Non-contact hypervariable region characteristics are critical for bNAb sensitivity
• HIV-1 Env 459C used alone as a vaccine can elicit modest tier 2 NAbs in guinea pigs
• V2 bNAb signature-guided modifications in 459C enhanced neutralization breadth

## Graphical Abstract

**Figure:**
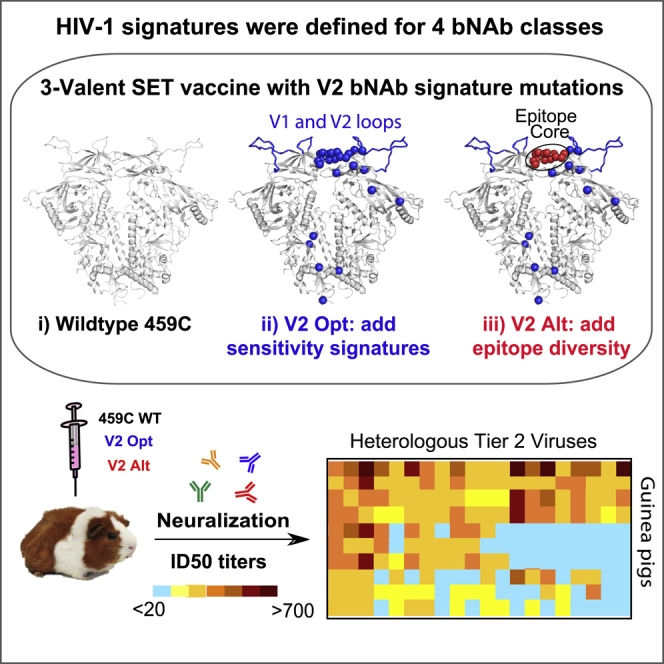

## Teaser

HIV-1 Env amino acid signatures associated with sensitivity to broadly neutralizing antibodies were systematically defined from large neutralization panels. V2 signatures were incorporated in a trivalent vaccine to enhance epitope exposure and to include common epitope variants, resulting in increased neutralization breadth against heterologous viruses in a guinea pig model.

## Introduction

Vaccine induction of broadly neutralizing antibodies (bNAbs) against diverse global tier 2 HIV-1 strains remains an unsolved challenge for the HIV-1 vaccine field. There has been some progress in animal models (Escolano et al., 2016, Saunders et al., 2017), but human trials have yet to elicit bNAbs, although NAbs with varying levels of breadth arise during natural infection (Hraber et al., 2014b). bNAbs typically develop slowly during chronic infection as the virus diversifies under immune pressure and B cell lineages adapt to the evolving virus (Bonsignori et al., 2017b, Doria-Rose et al., 2014, Liao et al., 2013, Wu et al., 2015). bNAb breadth and potency are evaluated using large panels of HIV-1 Envelope (Env) pseudoviruses that sample global HIV-1 diversity (Hraber et al., 2014a) or the C clade diversity of Southern Africa (Rademeyer et al., 2016).

We used data from 4 large neutralization panels for a more comprehensive mapping of viral signatures associated with bNAb sensitivity than undertaken previously (Chuang et al., 2013, Evans et al., 2014, Ferguson et al., 2013, West et al., 2013). Signature sites were identified using a strategy that incorporates a phylogenetic correction (Gnanakaran et al., 2010) for amino acids (AAs) and potential N-linked glycosylation sites (PNGSs) (Crispin and Doores, 2015), and we also explored the impacts of hypervariable region characteristics and clades. Recurrent signature patterns were found among bNAbs with shared specificities (Burton and Hangartner, 2016).

We next used variable V2 apex (V2) epitope bNAb signatures to inform HIV-1 Env immunogen design in a proof-of-concept exploration of an approach we call signature-based epitope targeted (SET) vaccines. Other vaccine design strategies include engaging bNAb germline precursors (Steichen et al., 2016), using polyvalent sets to capture diversity (Korber et al., 2017), lineage-based designs (Bonsignori et al., 2017b), and engineered native-like Envs (e.g., SOSIPs) (Sanders et al., 2013, Steichen et al., 2016). SOSIP vaccines elicit robust autologous NAbs that have limited breadth in rabbits and non-human primates (NHPs) (Pauthner et al., 2017, Sanders et al., 2015).

Our SET vaccine design started with the Env 459C (Bricault et al., 2015), as 459C alone elicited modest neutralization of some tier 2 heterologous strains in guinea pigs. V2-SET immunogens are a trivalent combination of 459C wild-type (WT) plus two additional proteins designed by modifying 459C to include V2 bNAb signatures intended to both enhance V2 epitope exposure and include relevant variation. V2-SET vaccines expressed as either gp140 SOSIP trimers or foldon trimers elicited increased NAb breadth compared to 459C alone in guinea pigs, suggesting the potential utility of bNAb signatures in vaccine design.

## Results

### Neutralization Data

**Figure 1:**
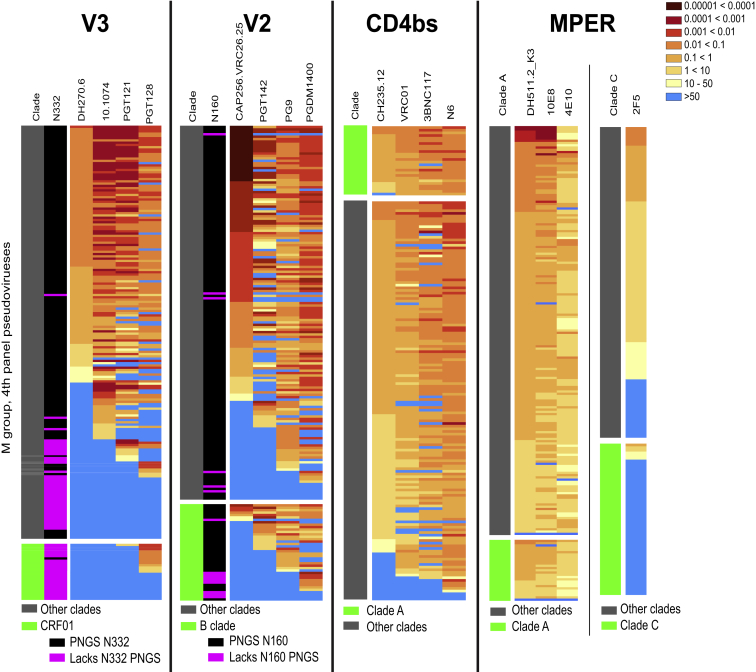
Heatmaps Showing IC_50_ Neutralization Titers for Dataset 4 Darker red hues indicate more potent neutralization and blue indicates undetected responses. Rows represent pseudoviruses, ordered differently in each panel to highlight commonalities in neutralization profiles across bNAbs in each class. The clade with the strongest clade effect is separated and indicated in green to the left. Key PNGSs are indicated by magenta. Among MPER bNAbs 2F5 is considered separately as it has a unique epitope.

Four datasets measuring the sensitivity of bNAbs against panels of HIV-1 Envs were analyzed. Three panels sampled global viral diversity (Hraber et al., 2014a), and the other sampled only C clade, which dominates in Southern Africa (Rademeyer et al., 2016). Table S1 summarizes bNAb dataset inclusion, relationships, and provenance. bNAbs are grouped by epitope class: V2, V3 glycan (V3), CD4 binding site (CD4bs), and membrane proximal external region (MPER) (Burton and Mascola, 2015). V2 and V3 bNAbs often have great potency but limited breadth, CD4bs have expanded breadth, and MPER has high breadth but low potency (Figures 1, S1, and S2). Heatmaps displaying inhibitory concentrations of 50% (IC_50_) data illustrate shared pattern sensitivity across bNAb classes (Figures 1 and S1), enabling the definition of common bNAb class signatures.

### Neutralization Sensitivity and HIV-1 Clades

**Figure 2:**
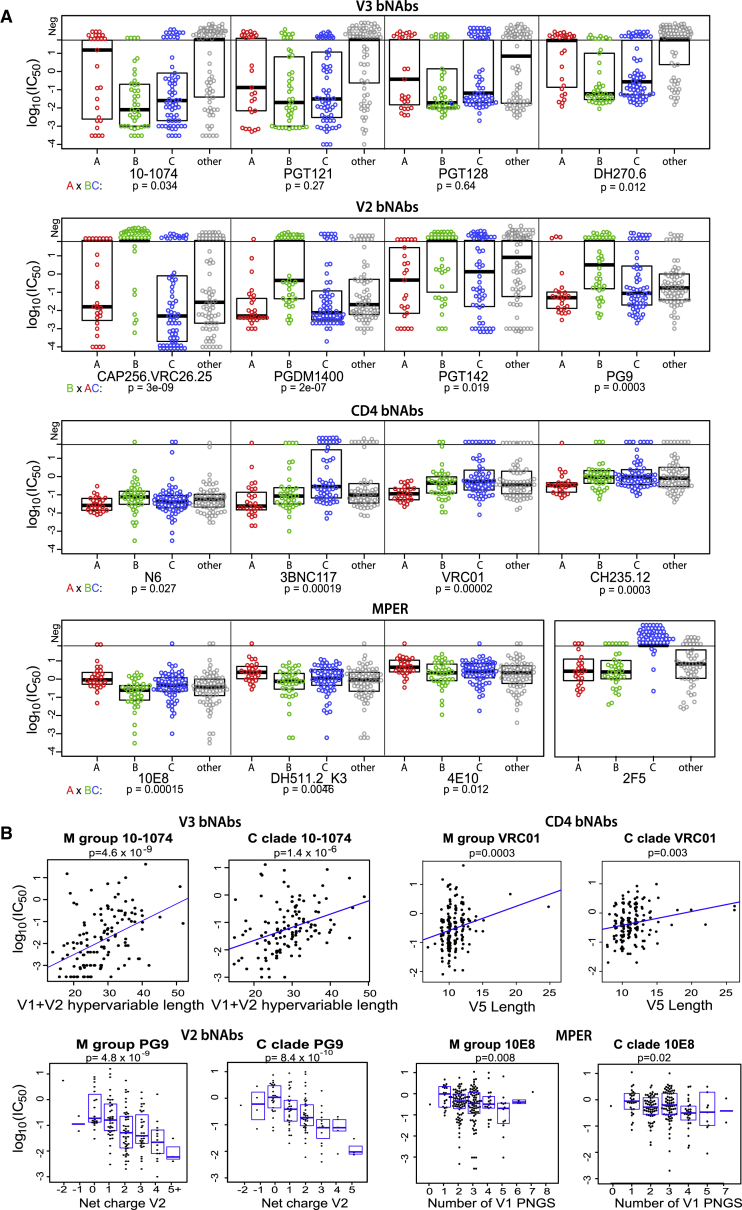
Env Characteristics Associated with bNAb Class Sensitivity (A) Clade associations. Circles illustrate IC_50_ titers from dataset 4, highlighting the 3 best represented clades: A in red, B in green, and C and CRF07 (which is clade C in Env) in blue. All others are gray. Boxplots show medians and quartiles. Patterns of relative clade sensitivity are consistent across bNAb classes. The p values are based on two-sided Wilcoxon tests comparing the most distinctive clade among A, B, and C to the other two clades. Points above the horizontal line were above the threshold of detection. The bolus of negative points in the “other” group for V3 bNAbs is primarily CRF01. (B) Examples of hypervariable loop characteristic correlations with bNAb sensitivity, including one for each bNAb class (complete associations are in Tables S3N–S3Q). M-group and clade C data are from datasets 4 and 3, respectively. The p values are based on Kendall’s tau.

bNAb sensitivity patterns are associated with HIV-1 clades, which have geographic associations (Korber et al., 2009). The V2 bNAb lineage CAP256.VRC26 is less potent against clade B viruses (Doria-Rose et al., 2016); diminished clade B potency extends across the V2 bNAb class (Figures 1, 2A, and S2). CD4bs bNAbs are more sensitive to clade A viruses, and some bNAbs are less sensitive to C clade, in particular 3BNC117 and VRC01 (Figures 1, 2A, and S2). MPER bNAbs are less potent against clade A. Circulating Recombinant Form 01 (CRF01) viruses, common in Southeast Asia, are extremely resistant to V3 bNAbs (Figures 1 and S2), which is driven by a PNGS shift from positions N332 to N334 found in 96% of CRF01 viruses (Figure 1; Table S2). The N332 glycan directly contacts V3 bNAbs (Kong et al., 2013) and is essential for some, although PGT128 can tolerate its loss (Figure 1). CRF01 was already highly diverse in central Africa when a founder seeded the Thai epidemic (Korber et al., 2000); African CRF01 viruses also lack the PNGS at N332. PNGS N332 frequencies vary between clades, correlating with overall clade sensitivity to V3 bNAbs (Table S2); e.g., ∼40% of A clade viruses are resistant to most V3 bNAbs, and 43% lack the PNGS N332 (Figure 2A; Table S2). The N332 PNGS is under-represented among transmitted C clade viruses (Rademeyer et al., 2016), which may impact the efficacy of V3 bNAbs in southern Africa.

### Amino Acid Signatures

**Figure 3:**
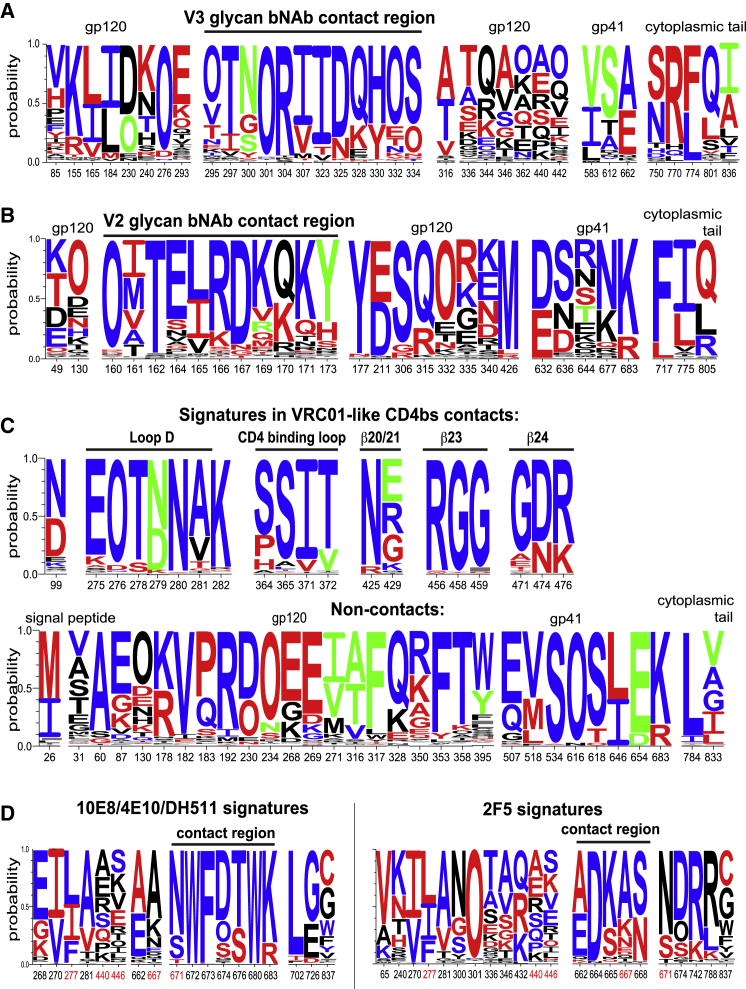
Sequence LOGOs of AA Signatures by Antibody Class This figure highlights the more robust signature sites in that they were supported by multiple lines of evidence—they either had phylogenetically corrected associations supported by at least 2 datasets, were a signature site in a contact residue, or both. Not all bNAbs in a class are associated with every signature. Complete lists with detailed statistics are provided in Table S3. Letter height represents AA frequencies in dataset 4. “O” represents an Asn in a PNGS motif. AAs associated with resistance and sensitivity are red and blue, respectively. AAs shown in green differ for different bNAbs within the class. (A) V3 bNAbs, (B) V2, (C) VH1-2 and VH1-46 CD4bs, and (D) MPER, with 10E8/4E10/DH511 on the left, 2F5 on the right, and red HXB2 position numbers highlighting opposing signatures between the two.

Signature patterns were identified by testing each AA and PNGS across Env alignments for associations with sensitivity to each bNAb in each of the 4 neutralization panels (Figure S1), based on associations with either potency or detectable neutralization (Tables S3A–S3D; Figure 3). Key signature sites were initially identified using a method that corrects for phylogenetic artifacts (Gnanakaran et al., 2010) (Tables S3E–S3H). Once a site was deemed of interest by this stringent criterion, any significant associations within these key sites were listed (Tables S3I–S3L).

### CD4bs bNAbs

While many CD4bs signatures are in contacts (Figures 3 and S3; Table S3) and known to influence sensitivity (Gao et al., 2014, Lynch et al., 2015), most are outside of contact surfaces. We tested two CD4bs resistance signatures directly by introducing them into the CH505 transmitted-founder (TF) backbone. The G458Y signature mutation conferred complete resistance (IC_50_ > 25 μg/mL) to VRC01 and 3BNC117, and both can neutralize the CH505 TF (IC_50_ of 0.14 and 0.03 μg/mL, respectively). The non-contact T234N signature substitution introduced a PNGS at N234 that increased resistance to CD4bs bNAbs 5- to 7-fold, and IC_50_ titers went from 0.36 to 1.82 μg/mL for CH235 and from 0.12 to 0.87 μg/mL for VRC01.

### MPER bNAbs

As their breadth is so great, most 10E8, 4E10, and DH511 signatures are associated with potency, except for rare mutations associated with complete resistance: W672L, F673L, and W680G (Figures 3 and S3; Table S3). The 10E8-specific resistance signature N671T accounts for the increased breadth of DH511 lineage bNAbs over 10E8 (Williams et al., 2017). The MPER epitope signature K683R is also associated with resistance to CD4bs and V2 bNAbs (Figure 3; Table S3), consistent with MPER changes impacting overall neutralization sensitivity (Bradley et al., 2016). Clade C resistance to 2F5 may be explained by clade C not having an otherwise conserved Ala, A667 (Figure S3).

### V3 bNAbs

Glycans that interact directly with V3 bNAbs (Behrens et al., 2016) and are positive signatures for neutralization are at positions N332, N301, and N295 (Figures 3 and S3; Table S3). Similar to CD4bs and MPER bNAbs, V3 bNAb sensitivity signatures in the epitope were relatively conserved, with positive signatures as the most common variant (Figure 3). In contrast, V3 bNAb signature sites between positions 336–442 were extremely variable and so may contribute to more nuanced levels of potency. In the GDIR contact motif (Sok et al., 2016) only D325 is a signature, because the other 3 positions are nearly invariant.

### V2 bNAbs

V2 bNAbs contact glycans at positions N156 and N160. The PNGS N160 is critical for many V2 bNAbs, with a few exceptions that may be enabled by nearby glycans acting in compensatory role (Figure 1; Table S3) (McLellan et al., 2011). In contrast, 11 viruses of the 380 unique viruses in the combined datasets 3 and 4 lacked the PNGS at N156, and these 11 viruses had very similar distributions of IC_50_ titers to viruses that had the PNGS site for all V2 bNAbs tested. PNGSs at positions N130 and N332 are associated with V2 bNAb resistance. The glycan at N130 is near the CAP256.VRC26 contact surface (Figure S3) and it interacts with CH03 (Gorman et al., 2016), but glycans at N130 and N332 may also act indirectly through glycan dynamics (Stewart-Jones et al., 2016) or carbohydrate processing (Behrens et al., 2016). Many V2 bNAb contact signatures have been studied: K169 and K171 loss, and Q170K, confers resistance, while E/D164 increases CAP256.VRC26.25 sensitivity (Doria-Rose et al., 2012, Doria-Rose et al., 2016).

### Evolutionary Counter-pressure

Some resistance signatures come with a fitness cost (Lynch et al., 2015). Also, some signatures are associated with resistance or sensitivity depending on the bNAb class (Figure 3; Table S3), including the PNGS at N130, associated with V2 bNAb resistance and CD4bs VRC03 sensitivity; the PNGS at N332, associated with V3 bNAb sensitivity and V2 bNAb resistance; and L165, associated with V2 bNAb sensitivity and V3 bNAb resistance (Figure 3; Table S3). Antibodies within a class can also have contradictory signatures. Some MPER signature AAs have opposing associations for 2F5 versus 4E10/10E8/DH511 (Figures 3 and S3; Table S3). A negatively charged D279 is associated with VRC01 sensitivity and with 12A12 resistance, likely due to the local charge in the bNAb paratopes (Figure S3) (Klein et al., 2013). CD4bs signatures for CDRH3 bNAbs (Zhou et al., 2015) often had opposing signatures relative to VH1-2 or VH1-46 bNAbs (Table S3).

### Clade Sensitivity

Signature sites offer hypotheses to explain bNAb clade sensitivities. Four contact site candidates were proposed to contribute to the reduced reactivity of CAP256.VRC26 bNAbs with B clade viruses (Doria-Rose et al., 2016). We found an additional 17 signatures that may limit V2 bNAb potency against B clade viruses (Figure S4A). In contrast, CAP256.VRC26 bNAbs are most potent against clade C viruses and 12 signatures may be relevant. CD4bs antibodies have enhanced potency against A clade viruses, and resistance signatures are relatively rare in A clade (Figure S4B); in contrast, 3BNC117 and VRC01 have reduced breadth and potency against C clade viruses (Figure 2).

### Hypervariable Loops and Neutralization Sensitivity

HIV-1 Env hypervariable regions evolve rapidly *in vivo* by insertions and deletions, giving rise to extreme length variation and making alignments spanning these regions unreliable. These changes can mediate bNAb escape (Bonsignori et al., 2016, Gao et al., 2014, Korber et al., 2017). We use alignment-independent characteristics of these regions (length, net charge, and number of PNGSs) to identify patterns associated with bNAb resistance profiles. The strongest of these associations are included in Tables S3M–S3P, and examples are shown in Figure 2B.

Combined V1+V2 length was the strongest correlate with V3 bNAb neutralization resistance—so insertions in either or both regions reduce sensitivity—followed by the number of PNGSs in V1+V2 and V1 length (Figure 2B; Table S3). V1 length variation played a critical role in the development of V3 bNAb DH270 lineage (Bonsignori et al., 2017a). A glycan in hypervariable V1 contacts PGT121-family bNAbs, enabling interactions when the key N332 glycan is absent. However, when N332 glycan is present, consistent with our signature predictions, removing this glycan enhances PGT121-family sensitivity (Garces et al., 2015).

The strongest variable region correlation with V2 bNAb sensitivity was V2 loop net-positive charge (Figure 2B; Table S6). The effect remained strong when only the V2 hypervariable region (positions 185–190) was considered. V2 bNAbs have long anionic CDRH3 loops (Doria-Rose et al., 2012, McLellan et al., 2011), which may drive the preference for positive charge. Also, V1+V2 hypervariable region length was inversely correlated with V2 bNAb sensitivity (Table S3).

Paradoxically, although CD4bs bNAbs can have great breadth, they bind near the V5 region which is subject to extreme length variation. Both V5 length and the number of PNGSs within V5 are associated with reduced CD4bs bNAb potency (Figure 2B; Table S3). CD4bs bNAbs can be selected to tolerate changes in V5 length after they arise as escape mutations *in vivo* (Fera et al., 2014). Long V1 and V2 regions also correlate with CD4bs bNAb resistance (Table S3) and mediate *in vivo* escape (Gao et al., 2014). Hence, long V1 and V2 regions are associated with relative resistance to 3 major classes of bNAbs: CD4bs, V2, and V3. At the population level, there are evolutionary counter-pressures against larger loops, as smaller hypervariable regions tend to be selected at transmission (Derdeyn et al., 2004). Unexpectedly, increasing numbers of PNGSs in V1 correlated with enhanced sensitivity to MPER bNAbs, particularly 10E8 (Figure 2B; Table S3).

### Machine Learning Predictions of Env Neutralization

**Figure 4:**
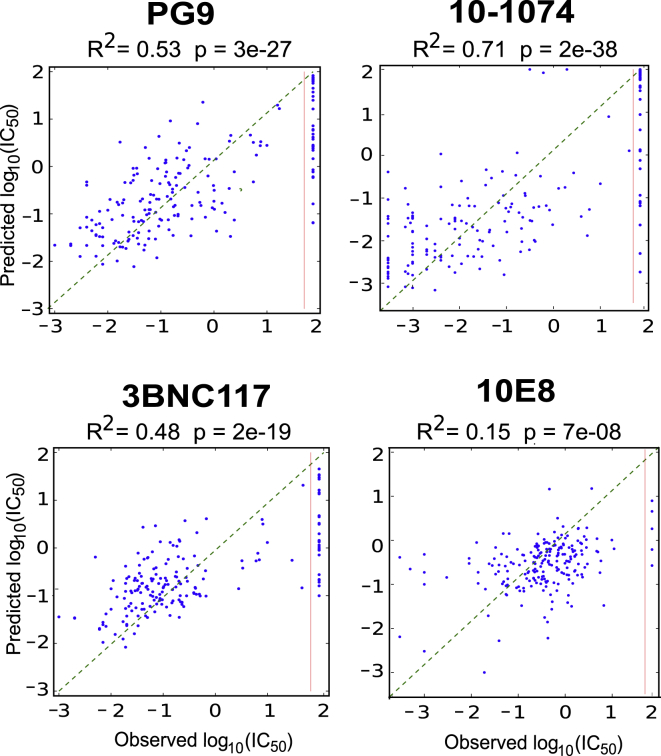
Random Forest Signature-Based Prediction Accuracy Leave-one-out cross-validation regression predictions using dataset 4 for one bNAb from each bNAb class. R^2^ is the standard coefficient of determination; the p value is from Kendall’s tau. The red line marks the threshold of detection. R^2^ values remained significant when negative points were excluded: PG9, R^2^ = 0.35, p = 4 × 10^−14^; 10–1074, R^2^ = 0.27, p = 2 × 10^−9^; 3BNC117, R^2^ = 0.30, p = 3 × 10^−9^; and 10E8, R^2^ = 0.12, p = 9 × 10^−7^.

We next explored using bNAb signatures for sequence-based machine learning predictions of bNAb sensitivity. Using a Random Forest (RF) for IC_50_ regression predictions, we compared prediction accuracies for 3 prefiltering strategies: (1) a standard prefilter, minimal-redundancy-maximal-relevance (mRMR) (Peng et al., 2005); (2) the full bNAb signatures for each antibody class, including AA, PNGS, clade, and variable loop characteristics; and (3) only signatures in contact sites. We evaluated predictions using leave-one-out cross validation for dataset 4, as well as for an independent C clade holdout set. The accuracy using the full signature was superior to mRMR (p = 0.003, paired Wilcoxon, C clade holdout comparison), and to the contact-region-only signature (also p = 0.003) (Table S4); examples are shown in Figure 4. We also tested positive/negative classification predictions using our prefilter strategies and RF, comparing them to a published method called IDEpi (Hepler et al., 2014); the methods were comparable (Table S5). Hypervariable region characteristics were consistently among the most important factors for predicting IC_50_ titers (Table S6).

### V2-SET Vaccines

#### Immunogen Design and Expression

**Figure 5:**
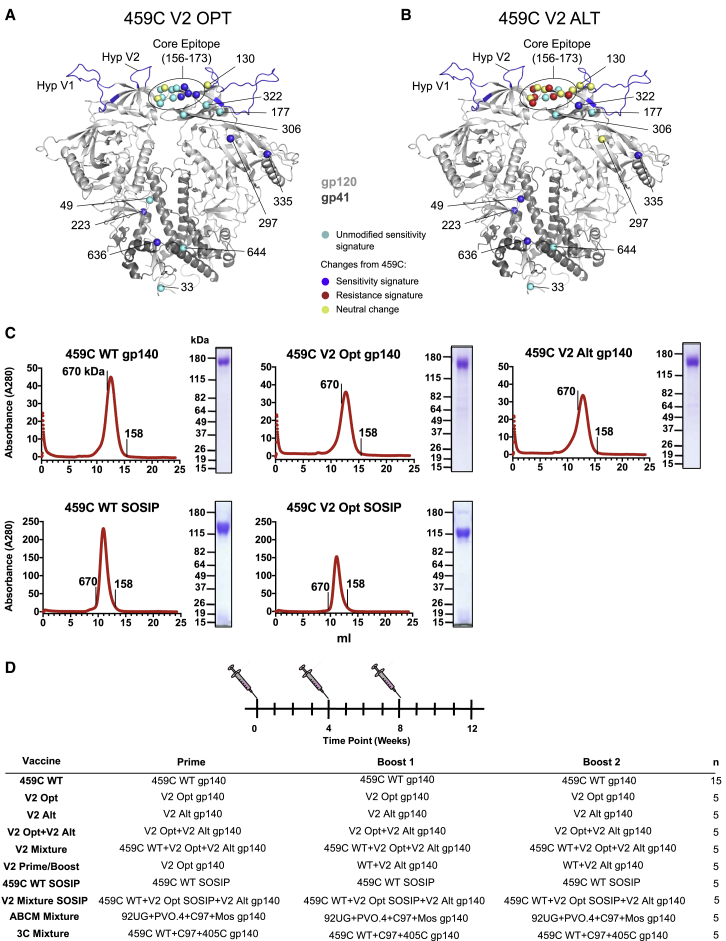
V2-SET Vaccine Design and Production (A and B) Structural mapping (PDB: 5FYJ) of mutations introduced into 459C WT (Table S7) to create (A) Opt and (B) Alt V2-SET vaccine constructs. Spheres are color-coded to indicate AA modifications associated with sensitivity or resistance. Opt constructs uniformly carry sensitivity signatures. Alt constructs carry mutations that enhance sensitivity outside the core epitope, but in the epitope introduce signature mutations complementary to 459C WT and Opt to capture epitope diversity. (C) Gel filtration chromatography traces of gp140 V2-SET immunogens run on a Superose 6 column (foldon) or Superdex 200 column (SOSIP). Coomassie stained SDS-PAGE of purified Envs are next to each trace with molecular weight standards noted. (D) Guinea pig vaccination regimens. Animals were vaccinated intramuscularly in the quadriceps with 100 μg total immunogen at weeks 0, 4, and 8; n is the group size.

We next utilized V2 bNAb signatures to inform vaccine design, intending to increase V2 epitope exposure and represent relevant natural diversity within the epitope. V2-SET trivalent vaccines included 459C WT, which alone induced low-level neutralization of some tier 2 viruses plus two complementary immunogens, called Optimal (Opt) and Alternative (Alt) (Figures 5A and 5B; Table S7). Opt introduced V2 bNAb virus sensitivity signatures into the 459C WT backbone, to enhance epitope expression, exposure, affinity, or relevant carbohydrate processing (Figure 5A). Alt incorporated V2 bNAb sensitivity signatures outside of the contact region; however, within the epitope it captured natural diversity in V2 signature sites—including globally common AAs complementary to those found in 459C WT and Opt—even if associated with relative resistance (Figure 5B). V2-SET immunogens also incorporated modified hypervariable regions with characteristics favoring V2 bNAb sensitivity, including short V1 and V2 hypervariable regions with a positively charged V2 region (Figure 2B; Tables S3N–S3R). The WT Env T250_4 met these criteria and was also highly sensitive to *both* V2 and V3 bNAbs (Figure S1). Hypothesizing T250_4 hypervariable regions might improve polyclonal responses to both bNAb classes, we used T250_4’s V1 and V2 regions in our V2-SET constructs (Table S7).

The V2-SET gp140s were produced in 293T cells by transient transfection as either SOSIP or foldon immunogens. The gp140 foldon contains a C-terminal T4-fibritin trimerization domain following the MPER and is not cleaved by furin (Frey et al., 2008). The gp140 SOSIP is a native-like trimer truncated before the MPER and cleaved by furin (Steichen et al., 2016). Each purified Env protein ran as a single symmetrical peak by size exclusion chromatography, and as a single band on SDS-PAGE (Figure 5C). We could not produce a stable V2 Alt SOSIP.

The antigenic properties of the V2-SET foldon immunogens were probed using surface plasmon resonance (Tables S5A–S5C). Soluble CD4 (Kwong et al., 1998) and V3 bNAb 10–1074 both bound to all three. V2 bNAb PG16 bound Opt and Alt immunogens more robustly than 459C WT, consistent with increased V2 exposure. Trimer-specific V2 bNAbs PGT145 and PGDM1400 bound robustly to SOSIP but not to foldon gp140s, consistent with SOSIP gp140s being native-like (Sanders et al., 2013, Steichen et al., 2016).

#### Immunogenicity of V2-SET Env Vaccines

Guinea pigs were immunized three times at monthly intervals (Figure 5D), intramuscularly in the quadriceps, with a total of 100 μg Env (divided equally among immunogens in cocktails) formulated with CpG/Emulsigen adjuvant. Animals were bled 4 weeks after each vaccination, with peak immunogenicity at week 12. Binding responses were assessed by ELISA using the immunogens and a small panel of additional Envs expressed as gp140s, and V1/V2 gp70 scaffolds (Kayman et al., 1994) (Figures S5D–S5E). All vaccination regimens elicited comparable high magnitude binding responses with similar kinetics. Furthermore, all vaccines elicited tier-1 NAb responses against easy-to-neutralize viruses (Figure S5F).

**Figure 6:**
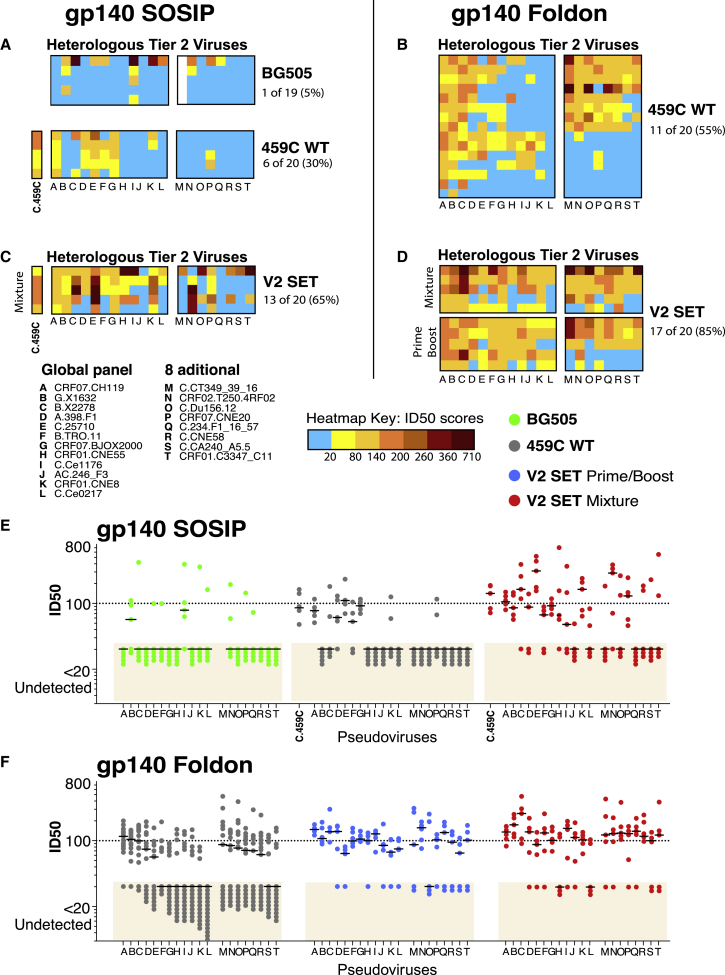
V2-SET Vaccines Improve the Breadth and the Magnitude of Tier 2 NAbs Compared to BG505 and 459C WT (A–D) Heatmaps of neutralizing responses comparing groups of guinea pigs vaccinated with 459C WT, V2-SET, and BG505 vaccines. Monovalent vaccines: (A) BG505 and 459C WT SOSIP and (B) 459C WT gp140 foldon. Trivalent vaccines: (C) V2-SET SOSIP and (D) gp140 foldon vaccines delivered as either a mixture or prime boost. Columns represent tier 2 pseudoviruses (see key), ordered by sensitivity. Rows represent guinea pigs, organized by vaccine group. The potency of ID_50_ responses increases from yellow to dark red, below threshold responses are blue. To compare the breadth of response between different vaccine regimens, the median number of detectable responses is reported for each vaccination regimen to the right of the heatmaps, and detectible responses per animal were compared by a two-sided Wilcoxon test. BG505 and 459C WT SOSIP vaccines were comparable (p = 0.13), and V2-SET SOSIP vaccine responses were broader than either BG505 (p = 0.02) or 459C WT (p = 0.01). Responses elicited by the V2-SET foldon vaccine were broader than responses elicited by the 459C WT foldon vaccine (p = 0.006). (E and F) Magnitudes of tier 2 NAb responses to SOSIP and foldon vaccine groups, respectively. Responses that were at least 10 above the background are considered positive and are shown; the dotted line at an ID_50_ titer of 100 is added for visual emphasis. Colors represent the vaccine groups (see key). Horizontal black lines are the median response to each pseudovirus. Response magnitudes were compared with a nonparametric permutation test (STAR Methods). For SOSIP vaccines, there was no statistical difference in potency between BG505 and 459C WT vaccine groups (p = 0.06), but V2-SET responses were more potent than both BG505 (p = 0.007) and 459C WT (p = 0.008). For gp140 foldon delivery, V2-SET responses were more potent than 459C WT (p = 0.002).

Trivalent V2-SET vaccines augmented the magnitude and breadth of neutralization against 20 tier 2 pseudoviruses (the standard global panel of 12 [deCamp et al., 2014], plus 8 additional) compared with 459C WT alone, using either the gp140 SOSIP or gp140 foldon vaccine platforms (Figure 6). Using the gp140 foldon platform, the V2-SET Mixture (the 3 vaccine components co-delivered) and V2-SET Prime/Boost (V2 Opt prime and V2 Alt+459C WT boost) both improved the magnitude of tier 2 NAbs responses compared to 459C WT alone (p = 0.006 and p = 0.008, respectively, using a non-parametric permutation test). A median of 85% of the heterologous tier 2 viruses were neutralized in the V2-SET groups, compared to 55% in the 459C WT alone group (p = 0.004, two-sided Wilcoxon rank sum) (Figure 6B). Using the SOSIP platform the V2-SET Mixture similarly improved neutralization potency compared to SOSIP 459C WT alone (p = 0.008), and a median of 65% of the heterologous tier 2 viruses tested were neutralized in the V2-SET group, compared to 30% for the 459C WT group (p = 0.008) (Figure 6A). In contrast, the BG505 SOSIP gp140 induced a median of 5% the heterologous tier 2 viruses tested, although one animal had more breadth (Figure 6C).

We next explored the generalizability these findings. First, tier 2 NAb responses with the V2-SET vaccine were similarly enhanced compared to 459C WT using a different adjuvant, MPLA (Nkolola et al., 2014b) (Figures S6A and S6B). Second, while V2 Opt and Alt delivered alone were immunogenic, they did not enhance neutralization breadth like the trivalent combination (Figure S6C). Third, the tier 2 NAb responses were mediated by purified IgG (Figure S7D). Finally, other previously studied multivalent Env cocktails involving natural sequence immunogens, a trivalent clade C vaccine (Bricault et al., 2015) and a tetravalent multiclade vaccine (Bricault et al., 2018), did not significantly enhance tier 2 breadth over 459C WT (Figure S6C). Together, these data suggest that the improved NAb breadth induced with the trivalent V2-SET vaccine was generalizable, dependent on the SET bioinformatic design, and could not be achieved by simple mixtures of WT Env immunogens.

**Figure 7:**
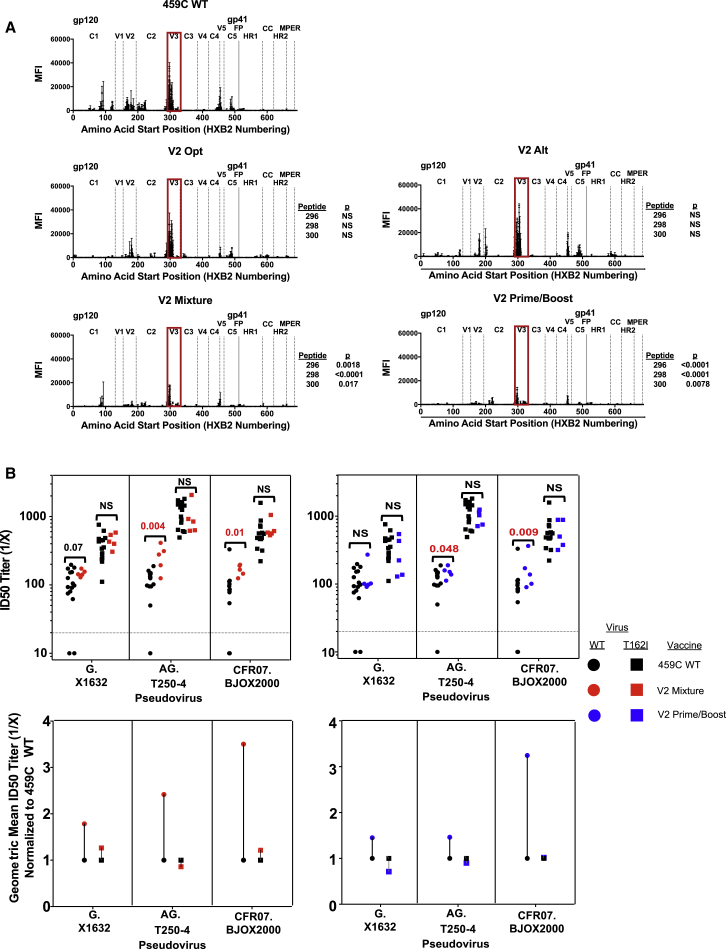
Mapping of Antibody Responses Elicited by V2-SET Vaccine (A) Magnitude and position of binding antibody responses from guinea pig sera to linear 15-mer peptides on peptide microarrays from each gp140 foldon vaccine group. Each dot represents an average MFI (mean fluorescence intensity) per peptide that is positive for antibody binding within each vaccination group, standard deviation shown. Env regions are delineated by vertical lines, the V3 loop highlighted in red. Statistical differences for binding responses to peptides with starting positions in V3 as compared to 459C WT are shown. The p values are based on a Wilcoxon one-sided test; NS means not significant. (B) Neutralizing titers against select pseudoviruses with a N160-dependent enhancement of V2-SET responses over 459C WT. ID_50_ titers in guinea pigs vaccinated with 459C WT and V2-SET vaccines against the native pseudoviruses are shown by dots, and against the N160 glycan deletion mutant (T162I) pseudoviruses by squares. Colors represent the particular gp140 foldon vaccination regimen: black is 459C WT, red is V2-SET mixture, and blue is V2-SET prime/boost. The top plots show ID_50_ titers for each guinea pig. The dotted line at 20 marks the limit of detection. The bottom plots show the geometric means of the NAb titers from the top plot (over the animals vaccinated by the same vaccine and tested on the same pseudovirus as in the top plot), normalized to 459C WT. The p values from Wilcoxon pairwise comparisons are shown in red. NS, not significant.

We also assessed post-vaccination antibody responses for binding to linear peptides spanning Env (Stephenson et al., 2015). Despite similar overall ELISA titers (Figure S5), V2-SET vaccines binding responses to linear V3 peptides were markedly lower than 459C WT, suggesting they were redirected away from non-neutralizing linear V3 epitopes (Figure 7A). To assess whether the improved V2-SET tier 2 NAb responses resulted from V2-specific conformational antibodies, we constructed pseudoviruses that resulted in the loss of the PNGS at position N160—a critical sensitivity signature for V2 bNAbs. The N160-PNGS-loss mutation pseudoviruses showed increased neutralization sensitivity to 459C WT vaccine-elicited NAbs, suggesting that a region partly shielded by the N160 glycan was targeted, but they also abrogated the enhancement in NAb potency achieved with V2-SET vaccine compared to the 459C WT vaccine (Figure 7B), suggesting that the improved the performance of the V2-SET vaccine over 459C WT depended on the PNGS at N160.

## Discussion

We defined HIV-1 Env bNAb signatures using neutralization data from four large virus panels to provide an unprecedented level of bNAb signature mapping—including hundreds of AA and PNGS signatures, as well as critical hypervariable region characteristics—for over 50 NAbs. The accuracy of Env sequence-based machine learning predictions of IC_50_ titers were generally improved by focusing on relevant signatures and hypervariable region characteristics that were consistently highly ranked as key features for accurate predictions; thus supporting the consideration of these characteristics in vaccine design. Such machine-learning-based predictions of bNAb sensitivity levels across populations of Env sequences may ultimately be useful for modeling the relative sensitivity of a set of bNAbs across a regional epidemic targeted for treatment and for interpreting results of bNAb-based prevention and therapeutic clinical studies. Our data also highlight the importance of HIV-1 clades for both bNAb passive infusion studies and vaccine studies.

We also developed a signature-based approach to Env immunogen design using the V2 bNAb signature patterns to inform the design of a trivalent V2-SET vaccine. Induction of heterologous tier 2 NAbs has proven to be a major challenge for the HIV-1 vaccine field, and to date, the breadth of tier 2 NAbs induced by vaccines has been modest in both small and large animal models. The native-like BG505 SOSIP trimer induces potent autologous NAbs but with minimal tier 2 NAb breadth (Pauthner et al., 2017, Sanders et al., 2015). The trivalent V2-SET vaccine induced greater breadth of tier 2 NAb responses than the 459C SOSIP trimer alone, and both proved superior to the BG505 SOSIP trimer. The improved NAb breadth using V2-SET antigens was reproducible in guinea pig vaccination studies and generalizable using two common HIV-1 Env trimer platforms (SOSIP and foldon gp140s) and two adjuvants (CpG/Emulsigen and MPLA). Moreover, the enhanced NAb breadth was not observed with two cocktails of natural sequence HIV-1 Env immunogens. Although the magnitude of the tier 2 NAb responses remained low to moderate, these data demonstrate the proof-of-concept that bNAb signatures can contribute to the design of next-generation HIV-1 Env immunogens.

## STAR★Methods

### Key Resources Table

**Table:** 

REAGENT or RESOURCE	SOURCE	IDENTIFIER
**Antibodies**		

10-1074	Laboratory of Michel Nussenzweig, Rockefeller University	RRID: AB_2491062; Mouquet et al. (2012)
PG16	Polymun	RRID: AB_2491031; Cat#AB016
PGT145	Catalent	RRID: AB_2491054
PGDM1400	Catalent	Sok et al. (2014)
HRP-conjugated goat anti-guinea pig secondary antibody	Jackson ImmunoResearch Laboratories	Cat#106-035-003
Alexa Fluor 647-conjugated AffiniPure Goat Anti-Guinea Pig IgG (H+L)	Jackson ImmunoResearch Laboratories	Cat#706-605-148

**Bacterial and Virus Strains**		

X1632 T162I pseudovirus	Laboratory of Christina Ochsenbauer, University of Alabama Birmingham	N/A
T250-4 T162I pseudovirus	Laboratory of Christina Ochsenbauer, University of Alabama Birmingham	N/A
BJOX2000 T162I pseudovirus	Laboratory of Christina Ochsenbauer, University of Alabama Birmingham	N/A

**Chemicals, Peptides, and Recombinant Proteins**		

HIV-1 gp70 V1/V2 (ConC)	Immune Technology Corp	Cat#IT-001-213p
HIV-1 gp70 V1/V2 (Case A2)	Immune Technology Corp	Cat#IT-001-214p
HIV-1 gp70 V1/V2 (CN54)	Immune Technology Corp	Cat#IT-001-211p
HIV-1 gp70 V1/V2 (A244)	Immune Technology Corp	Cat#IT-001-212p
HBS-EP	GE Healthcare	Cat#BR100188
Soluble CD4	Laboratory of Bing Chen, Children’s Hospital Boston	Freeman et al. (2010)
Amine Coupling Kit	GE Healthcare	Cat#BR100050
Pierce Recombinant Protein A	ThermoScientific	Cat#21184
Emulsigen	MVP Adjuvants	N/A
Monophosphoryl Lipid A from S. minnesota R595	InvivoGen	Cat#vac-mpla
SuperBlock T20 (TBS) Blocking Buffer	Thermo Scientific	Cat#37536
High-Capacity Protein A Agarose	Thermo Scientific	Cat# 89948
459C WT gp140 foldon	Bricault et al. (2015)	N/A
459C V2 Opt gp140 foldon	This paper	N/A
459C V2 Alt gp140 foldon	This paper	N/A
459C WT SOSIP	This paper	N/A
459C V2 Opt SOSIP	This paper	N/A
C97ZA012 gp140 foldon	Nkolola et al., (2010)	N/A
405C gp140 foldon	Bricault et al., (2015)	N/A
92UG037 gp140 foldon	Nkolola et al., (2010)	N/A
PVO.4 gp140 foldon	Li et al., (2005)	Accession number: AY835444
Mosaic gp140 foldon	Nkolola et al., (2014a)	N/A

**Deposited Data**		

Neutralizing antibody signatures will be deposited in the Los Alamos HIV database and accessible through the Genome Browser, the Neutralizing Antibody relational database, and the Env annotation tables.	Los Alamos HIV Database	https://www.hiv.lanl.gov/content/immunology/neutralizing_ab_resources.html, www.hiv.lanl.gov/components/sequence/HIV/featuredb/search/env_ab_search_pub.comp, www.hiv.lanl.gov/content/sequence/genome_browser/browser.html

**Experimental Models: Cell Lines**		

Human: 293T	ATCC	ATCC CRL-3216

**Experimental Models: Organisms/Strains**		

Hartley guinea pigs: Outbred	Elm Hill Labs	N/A

**Oligonucleotides**		

CpG: 5’-TCGTCGTTGTCGTTTTGTCGTT-3’	Midland Reagent Company	N/A

**Recombinant DNA**		

GeneArt	Life Technologies	Cat#817003DE

**Software and Algorithms**		

Softmax Pro-4.7.1	Molecular Devices	https://www.moleculardevices.com/systems/microplate-readers/softmax-pro-7-software
GenePix Pro 7 software	Molecular Devices	https://www.moleculardevices.com/en/asset/br/data-sheets/genepix-pro-software-datasheet-v7-rev-b
GenePix Array List	Stephenson et al., (2015)	N/A
GenSig, a signature analysis web interface	Los Alamos HIV Database	https://www.hiv.lanl.gov/content/sequence/GENETICSIGNATURES/gs.html
CATNAP, Neutralization data resource	Los Alamos HIV Database	https://www.hiv.lanl.gov/components/sequence/HIV/neutralization/
Filtered Forests	Los Alamos HIV Database, github	https://www.hiv.lanl.gov/content/sequence/FLTFORESTS/fltforests.html, https://github.com/hivdb-lanl/FilteredForests

**Other**		

RepliTope Antigen Collection HIV Ultra slides	JPT Peptide Technologies GmbH	Cat#RT-HD_HIV
CM5 Chips	GE Healthcare	Cat#BR100012

### Contact for Reagent and Resource Sharing

Further information and requests for resources and reagents should be directed to and will be fulfilled by the Lead Contact, Dan Barouch (dbarouch@bidmc.harvard.edu).

### Experimental Model and Subject Details

#### Human Subjects

The HIV-1 bNAbs used in this study were all isolated in the context of other studies (Table S1). The Env pseudoviruses are all part of widely used standard panels. Human specimens used to derive these reagents are de-identified and considered exempt by the Duke University IRB, and the exemption approved by the Los Alamos National Lab IRB.

#### Cell Lines

Human endothelial kidney 293T cells (ATCC) were used for transient transfection of HIV-1 Env expressing plasmids and stably transfected human endothelial kidney 293T cells (Codex Biosolutions) were utilized for the production of HIV-1 Env gp140 and SOSIP immunogens.

#### Guinea Pig Vaccinations

Healthy, outbred, research-naïve, female Hartley guinea pigs (bred at and purchased from Elm Hill) at between 350 and 500 grams and about 1 to 2 months of age were used for vaccination studies and housed at the Animal Research Facility of Beth Israel Deaconess Medical Center under approved Institutional Animal Care and Use Committee (IACUC) protocols. Animals were co-housed 2 to 5 animals per cage, based on animal weight. All animals were naïve at the initiation of the study. Guinea pigs (5-15/group) were immunized with Env gp140 immunogens intramuscularly in the quadriceps bilaterally at 4-week intervals (weeks 0, 4, 8) for a total of 3 injections. Vaccine formulations for each guinea pig consisted of a total of 100μg of immunogen per injection formulated in 15% Emulsigen (vol/vol) oil-in-water emulsion (MVP Laboratories) and 50 μg CpG (Midland Reagent Company) or 10 μg Monophosphoryl lipid A (MPLA) (InvivoGen) as adjuvants. We also tested the V2-SET immunogen sequences in the context of the gp140 MD39 SOSIP constructs (Steichen et al., 2016), using a lengthened schedule of vaccinations at weeks 0, 8, and 24. Serum samples were obtained from the vena cava of anesthetized animals four weeks after each immunization as well as prior to vaccination for week 0, naïve sera.

### Method Details

#### Experimental Methods for Vaccine Evaluation

##### Plasmids, Cell Lines, Protein Production, and Antibodies

Our baseline immunogen was the C clade Env 459C, initially selected because it elicited tier 1B NAb responses (Bricault et al., 2015), and subsequently found to induce low levels of select tier 2 NAbs upon evaluation of larger tier 2 pseudovirus panels. The codon-optimized synthetic genes of the V2-SET HIV-1 Env gp140 foldon (gp140) and gp140 MD39 SOSIP (SOSIP) immunogens were produced by GeneArt (Life Technologies). All gp140 constructs contained a consensus leader signal sequence peptide, as well as a C-terminal foldon trimerization tag followed by a His-tag as described previously (Frey et al., 2008, Nkolola et al., 2010). Large scale production of HIV-1 Env gp140 foldon and SOSIPs were produced as described previously (Nkolola et al., 2010, Nkolola et al., 2014a, Steichen et al., 2016). Of note, the gp140 SOSIP immunogens were cleaved by furin and the gp140 foldon immunogens were not. Soluble two-domain CD4 was produced as described previously (Freeman et al., 2010). 10-1074 was generously provided by Michel Nussenzweig (Rockefeller University, New York, NY). PG16 was purchased from Polymun Scientific, PGT145 and PGDM1400 from Catalent, gp70 V1/V2 HIV-1 envelope scaffolds including ConC, Case A2, CN54, and A244 V1/V2 from Immune Technology Corp.

##### Surface Plasmon Resonance Binding Analysis

SPR experiments were conducted on a Biacore 3000 (GE Healthcare) at 25°C utilizing HBS-EP [10 mM Hepes (pH 7.4), 150 mM NaCl, 3 mM EDTA, 0.005% P20] (GE Healthcare) as the running buffer. Immobilization of CD4 (∼1,000 response units (RU)) or protein A (ThermoScientific) to CM5 chips was performed following the standard amine coupling procedure as recommended by the manufacturer (GE Healthcare). Select protein-protein interactions were analyzed using single-cycle kinetics consisting of four cycles of a 1-min association phase and a 4-min dissociation phase without regeneration between injections, followed by an additional cycle of a 1-min association phase and a 15-min dissociation phase, at a flow rate of 50 μL/min. Immobilized IgGs were captured at about 500 RU for 10-1074 and about 3,000 RU for PG16. Soluble gp140 foldon was then passed over the surface at increasing concentrations from 62.5 nM-1,000 nM. Regeneration was conducted with 35 mM NaOH, 1.3 M NaCl (pH 12) at 100 μL/min followed by 5-min equilibration in the HBS-EP buffer. For experiments run with PGDM1400 and PGT145 IgG, immobilized PGDM1400 and PGT145 IgGs were captured at between 150-200 RU. Binding experiments were conducted with a flow rate of 50 μl/min with a 2-minute associate phase and a 5-minute dissociation phase. Soluble gp140 foldon or gp140 SOSIP were then passed over the surface at increasing concentrations from 31.25 nM-500 nM. Regeneration was conducted with one injection (3 seconds) of 35 mM sodium hydroxide, 1.3 M sodium chloride at 100ul/min followed by a 3-minute equilibration phase in HBS-EP. Identical injections over blank surfaces were subtracted from the binding data for analysis. All samples were run in duplicate and yielded similar kinetic results. Single curves of the duplicates are shown in all figures.

##### Endpoint ELISAs

Serum binding antibodies against gp140 foldon and V1/V2 scaffolds were measured by endpoint enzyme-linked immunosorbant assays (ELISAs) as described previously (Nkolola et al., 2010). Briefly, ELISA plates (Thermo Scientific) were coated with individual gp140s or V1/V2 scaffolds (Immune Technology) and incubated overnight. Guinea pig sera were then added in serial dilutions and later detected with an HRP-conjugated goat anti-guinea pig secondary antibody (Jackson ImmunoResearch Laboratories). Plates were developed and read using the Spectramax Plus ELISA plate reader (Molecular Devices) and Softmax Pro-4.7.1 software. End-point titers were considered positive at the highest dilution that maintained an absorbance >2-fold above background values.

##### Peptide Microarrays

RepliTope Antigen Collection HIV Ultra slides (JPT Peptide Technologies GmbH) arrays were generated, conducted, and analyzed using methods as described previously (Stephenson et al., 2015). These slides contain linear 15-mer peptides designed utilizing the HIV-1 global sequence database to provide coverage of HIV-1 global sequences (Stephenson et al., 2015). Briefly, microarray slides were incubated with guinea pig sera diluted 1/200 in SuperBlock T20 (TBS) Blocking Buffer (Thermo Scientific). Binding antibody responses were detected with Alexa Fluor 647-conjugated AffiniPure Goat Anti-Guinea Pig IgG (H+L) (Jackson ImmunoResearch Laboratories). All batches of slides were run in parallel with a control slide incubated with the secondary antibody only for background subtraction. Slides were scanned with a GenePix 4300A scanner (Molecular Devices) and analyzed with GenePix Pro 7 software and GenePix Array List (Stephenson et al., 2015). The threshold values for positivity were calculated as the point at which the chance that signal is noise as low as possible (P<10^-16^). The peak positive antibody binding responses to linear V3 Env peptides were further analyzed comparing the 459C WT and the V2-SET vaccines. Peptides with the highest magnitude binding responses were analyzed comparing geometric means over animals separately against each 15-mer peptide start position. Geometric means were calculated for each vaccination group resulting in a single point per vaccine per peptide sequence.

##### TZM.bl Neutralization Assay for Vaccine Sera

All IC_50_ data for the large neutralization panels were obtained using the validated luciferase-based TZM.bl assay (Sarzotti-Kelsoe et al., 2014); most antibodies to a maximum concentration of 50 μg/ml. For vaccine responses, 20 tier 2 pseudoviruses were used in the TZM.bl neutralization assay: the standardized global panel of 1HIV-1 reference strains independently selected to be representative of larger global panels (deCamp et al., 2014) and a panel of 8 additional tier 2 pseudoviruses selected because they were relatively sensitive to human sera (falling in the top quartile of geometric mean serological reactivity of the tier 2 panel), were sensitive to the V2 bNAb monoclonals (Yoon et al., 2015), and were relatively close in sequence to the V2-SET vaccines in the neutralization signature positions. The 8 additional pseudoviruses were added as an *a priori* attempt to increase the chances of getting a positive signal, but when tested were found to be very comparable in sensitivity to the global panel. The rationally selected tier 2 pseudoviruses included clade C (Du156.12, CT349_39_16, 234_F1_15_57, CNE58, and CA240_A5.5), CRF 02_AG (T250_4), CRF 07_BC (CNE20), and CRF 01_AE (C3347_C11) strains. For purification of guinea pig polyclonal IgG from sera, High-Capacity Protein A Agarose (Thermo Scientific) was utilized following manufacturer’s instructions. After purification by protein A, polyclonal IgG samples were buffer exchanged into 1X phosphate buffered saline, pH 7.4 (Gibco) utilizing a EMD Millipore Amicon Ultra-15 Centrifugal Filter Unit (Millipore) at 4°C. Mutant pseudoviruses were generated with point mutations in V2/glycans to map NAb responses targeting these epitopes. Point mutations aiming to abrogate V2 antibody neutralization were selected to minimize disruptions in the virus backbone by representing mutations that occur commonly in nature. A T162I mutation was introduced into X1632, T250-4, and BJOX2000 to knock out the glycan at position N160; this mutations is relatively common among natural isolates.

#### Sequence and Signature Analysis

##### Signature analysis

To systematically identify sites of interest, we used our phylogenetically corrected approach (Gnanakaran et al., 2010) to minimize false positives due to lineage effects (Bhattacharya et al., 2007), and q-values to constrain false positives due to multiple testing (Storey and Tibshirani, 2003). These sites are summarized by antibody in Tables S3A–S3D, and population frequencies of signatures are displayed as LOGOs in Figure 3. Statistical details for all phylogenetically corrected signatures that met the statistical cutoff (q < 0.2) are provided in a summary table organized by antibody (Tables S3E–S3H). To be more inclusive, within each bNAb class, for sites that have either have a phylogenetically corrected signature for any bNAb within that class or are in the epitope binding region for a crystalized representative of the class, we also list in Tables S3I–S3L all AA and PNGS associations with a q-value <0.2, without the constraint of a phylogenetic correction, organized by Env position (Tables S3I–S3L). For comparison, published bNAb signatures from previous studies (Chuang et al., 2014, Ferguson et al., 2013, West et al., 2013) are also included in Tables S3E–S3L). This comparison shows that our analysis provides more detailed mapping of sites that may be relevant to the overall bNAb sensitivity than has been previously assembled. Because the bNAb field is advancing rapidly and new data are continuously accruing, we have also integrated our signature code into the CATNAP bioinformatics tool into the Los Alamos HIV Immunology Database (Yoon et al., 2015), allowing signature analysis to be conducted on-the-fly as new bNAb data is entered into the database.

Phylogenetically corrected signature methods were described in detail in earlier publications (Bhattacharya et al., 2007, Gnanakaran et al., 2010). Briefly, for a simple uncorrected test, a 2 x 2 contingency table is generated where the data is divided about a phenotypic cutoff (*e.g.* “high” or “low” IC_50_ values split about the median) and whether or not a sequence has a given amino acid at a given position, and a Fisher’s exact test is used to assess statistical significance of each such contingency table. All amino acids are tested in all positions, and a false discovery rate (FDR) adjusted q-value (Storey and Tibshirani, 2003) with a threshold of <0.2 used to define sites of interest, to be inclusive but still limit false negatives. This simple test can also be used to test associations with PNGS.

Even with FDR, without a phylogenetic correction simple signatures can yield an extreme over-abundance of apparent results, and many associations will not be causative, but can be carried along by genetic linkage to a site where the variation has direct consequences. An example illustrating how can happen is provided in Figure S7. In this example, the CRF01 clade is highly resistant to V3 bNAbs, and this is likely to be primarily driven the loss of the critical PNGS at N332 throughout the entire clade. But given the lack of reactivity for V3 bNAbs among CRF01 sequences, any amino acid highly enriched among CRF01 sequences will be associated with V3 bNAb resistance, yet most are likely to not be causative. An association that is still statistically supported after a phylogenetic correction, which requires that the correlation between the amino acid and the phenotype recur in sequences in dispersed locations throughout the tree, is more likely to reveal direct causative associations with the phenotype.

For a phylogenetically corrected test, a maximum likelihood tree inferred by the signature code is used to estimate the most likely ancestral amino acids at branch points in the interior of the tree (Bhattacharya et al., 2007). For the branch point preceding each leaf node, the most likely amino acid is determined based on the most likely nucleotides at each position in the codon, which is translated to obtain the ancestral AA state of that leaf. A Fisher’s exact test contingency table is based on whether the amino acid changed away from or stayed the same as the ancestral state, and whether the neutralizing phenotype is resistant or sensitive. Full statistics and contingency tables are provided in Table S3, including detailed examples about how the contingency tables are constructed and their interpretation. As above, this phylogenetically corrected association test can also be used to analyze PNGS associations.

Several cutoffs were used to define relative sensitivity and resistance: IC_50_ titers being above (negative) or below (positive) the threshold of detection based on the highest concentration of Ab used, or partitioning the data about the median or the quartile responses. For a given amino acid at a given site, the results for phylogenetically corrected test with the lowest p-values are shown in Table S3 E-H, with the test performed indicated in the table. If there are ties, they are broken by presenting the undetected vs detected responses (called PosNeg) when they are available, or by presenting the median over the quartile breakdowns if the tie is just between those two cutoffs. We also present uncorrected associations for all amino acids in *positions of interest* with bNAb sensitivity, again with a q-value cutoff of 0.2 (Tables S3I–S3L). This enabled us to explore the potential of amino acids at these interesting positions to contribute to levels of bNAb sensitivity, with a less stringent test than required surviving a phylogenetic correction. Sites were *deemed of interest* for this extended exploration by being either located within epitope, or by being found to be significantly associated with IC_50_ titers using a phylogenetically corrected test for at least one bNAb in a class.

Throughout this study, signatures were generally defined using the Fisher’s exact test method described above, but we also explored using a non-parametric Wilcoxon rank sum test. In this case, the distributions of IC_50_ titers were compared when an amino acid or PNGS site was present or absent in a given position, in a phylogenetically corrected analysis. The Wilcoxon test was generally less sensitive than a Fisher’s test, however some CD4bs signatures were best defined using this test, and these results are provided in Table S3M.

##### Antibody references

Links between particular antibodies, references, and antibody provenance and relationships are provided in table format Table S1. Fourteen V3 glycan bNAbs were studied (Bonsignori et al., 2016, Garces et al., 2015, Julien et al., 2013, Kong et al., 2013, Mouquet et al., 2012, Pejchal et al., 2011, Trkola et al., 1996, Walker et al., 2011). Ten V2 bNAbs were analyzed (Bonsignori et al., 2011, Doria-Rose et al., 2014, Doria-Rose et al., 2016, McLellan et al., 2011, Sok et al., 2014, Walker et al., 2009, Walker et al., 2011). Twenty-six CD4bs bNAbs were studied (Bonsignori et al., 2017a, Burton et al., 1991, Corti et al., 2010, Diskin et al., 2011, Gao et al., 2014, Huang et al., 2016, Klein et al., 2012, Rudicell et al., 2014, Scheid et al., 2011, Wagh et al., 2016, Wu et al., 2010, Wu et al., 2011, Wu et al., 2015, Zhou et al., 2013, Zhou et al., 2015). These were grouped into 3 types (Zhou et al., 2015): VH1-2 restricted, VH1-46 restricted, and those with a CDR H3 dominant mode of binding (Table S1). The 4 MPER bNAbs studied were grouped by epitope, 2F5 or 4E10/10E8/DH511 (Buchacher et al., 1994, Huang et al., 2012, Nelson et al., 2007, Williams et al., 2017)

##### Phylogenetic trees

Maximum likelihood trees were generated based on amino acid sequences using PhyML (Guindon et al., 2010) using the HIVb model (Nickle et al., 2007) (https://www.hiv.lanl.gov/content/sequence/PHYML/interface.html), and represented using Rainbow Tree at the Los Alamos database (https://www.hiv.lanl.gov/content/sequence/RAINBOWTREE/rainbowtree.html), (Paradis et al., 2004).

##### Alignments

The signature analysis tool requires as input codon-aligned nucleotide alignments, which we generated using the Gene Cutter tool at https://www.hiv.lanl.gov/content/sequence/GENE_CUTTER/cutter.html, followed with hand editing. The complete dataset alignments for data sets 3 and 4 and TZM.bl neutralization assay IC_50_ data used in this study will be in the Special Interest Alignments page of the Los Alamos upon publication (https://www.hiv.lanl.gov/content/sequence/HIV/SI_alignments/datasets.html), and all of the neutralization data is publicly available through web-based *CATNAP* tool.

##### Sequence Representation

Amino acids single-letter codes are used throughout. Standard HXB2 numbers is used throughout. The Los Alamos database Analyze Align tool (https://www.hiv.lanl.gov/content/sequence/ANALYZEALIGN/analyze_align.html) was used to generate sequence LOGOS (Crooks et al., 2004). LOGOS represent the frequency of amino acids in the illustrations included here, the measure of interest for this study, in the M group dataset 4, or in the C clade dataset 3. During the course of this study we have built convenient features into the Analyze Align LOGO generation tool: (1) when an N is embedded in a glycosylation site motif Nx[ST], we replace N with the letter O in the LOGO figure, otherwise we leave it as an N, (2) a grey box is used to indicate gaps inserted to maintain the alignment, (3) specific color schemes (e.g. our red/blue sensitivity/resistance color scheme) are now available, and (4) the tool can now make LOGOs of discontinuous sites by utilizing HXB2 numbering.

##### Hypervariable region characterization

Hypervariable regions were characterized using the Los Alamos database *Variable Region Characteristics* tool: https://www.hiv.lanl.gov/content/sequence/VAR_REG_CHAR/index.html. The variable loops V1, V2, V4, V5 each have hypervariable regions that frequently mutate by insertion and deletion. V3 loops have low levels of mutation by insertion and deletion, thus this region is readily aligned and was not considered hypervariable. The boundaries of these regions are shown in Table S3 R. We systematically tested for correlations with each variable region characteristic (length, net charge, and number of PNGS) and Ab sensitivity for every Ab in each dataset, and calculated q-values to address multiple tests. If an Ab in a class had a q value of < 0.20 with a V-loop correlate, it was considered of interest, and all other Abs of that same class were tracked and included in Tables S3N–S3Q. The hypervariable nature of these regions leads to rapid changes in them within the course of a given infection, so one would expect markedly diminished phylogenetic correlation with these hypervariable loop characteristics across a population, and thus a phylogenetic correction is not appropriate for these analyses. For each bNAb, we tested for correlations between all variable region characteristics and bNAb sensitivity, both including and excluding censored data (Tables S3N–S3Q).

### Quantification and Statistical Analysis

#### Sequence Analyses

##### Signature Statistics

To address multiple tests false discovery rate q-values were calculated (Storey and Tibshirani, 2003). For all signature comparisons in Table S3, a q-value < 0.2 was required for inclusion. We built our own Fisher’s exact text code and q-value estimates into the signature analysis package (Bhattacharya et al., 2007). We use R (www.r-project.org/) to perform Wilcoxon rank sum comparisons of distributions, to perform Kendall’s tau (McLeod, 2011) to test for correlations, and to calculate q-values for addressing multiple tests when evaluating variable loop characteristics (Dabney and Storey, 2013). Heatmaps were generated using the Los Alamos HIV database tool (https://www.hiv.lanl.gov/content/sequence/HEATMAP/heatmap.html).

##### Machine learning predictions

We used the Python package scikit-learn for machine learning predictions of bNAb sensitivity (Pedregosa et al., 2011). We initially compared several machine learning strategies (Random Forest, Support Vector Machine, and Linear Discriminant Analysis) using the M group cross-validation scores; the C clade holdout group was not considered during this selection process. Random Forest (RF) (Breiman, 2001) strategies performed best, in particular the ExtraTreesRegressor and ExtraTreesClassifer methods (Geurts et al., 2006) gave the highest accuracy overall, and so were used here as a basis for comparing the accuracy of sequence-based filtering strategies for obtaining input features for neutralization predictions. The overall RF result is obtained by combining the results from each of the individual trees in the forest. For our RF experiments, we fixed the size of the ensemble to 250 trees but otherwise used default values of the scikit-learn parameters. In particular, the depth of the trees (*i.e.,* the number of branches) was adaptively determined using a bootstrap approach available in scikit-learn.

As mentioned in the text, the three pre-filtering strategies used were: mRMR, the full signature set (including outside epitope signatures, hypervariable loop characteristics and clade associations), and only signatures in the bNAb epitopes. Input data files for signature-based prefiltering were created with columns of data translated so that clades, and signature amino acids and PNGSs in a given position, were assigned a 1 if associated with sensitivity, -1 if associated with resistance, or 0 if not associated. Quantitative values for correlated variable loop characteristics were also included.

For each pre-filter strategy, we obtained predictions for two scenarios. First, we predicted IC_50_ titers for the 207 Env sequences from the M group dataset 4, using leave-one-out cross-validation. We decided to use leave-one-out cross validation because the datasets were small enough that we were not computationally constrained, and this approach minimizes bias in small data sets (Arlot and Celisse, 2010). Second, we trained the RF on the M group data from datasets 1,2 and 4, and evaluated the performance on a holdout C clade virus set from dataset 3. To maintain the independence of our holdout set, we used signature pre-filters that were defined only on the basis of the M group datasets 1, 2, and 4, and we excluded the 26 pseudoviruses from the 200 in the C clade set that were also found in M group data. Predictions were made for the 13 bNAbs that were available in both dataset 3 and 4, and both regression (IC_50_ titers; Table S4; Figure 4) and classification (positive versus negative; Table S5) prediction accuracies were assessed. We compared the accuracy of results using different strategies to pre-filter Env sequence alignment data.

For regression, the aim is to predict the potency, and here we used three measures of performance to assess the quality of these predictions. The most direct measure is the mean absolute value of the prediction error (MAE). We also used the R^2^ statistic (the coefficient of determination), whose variation is generally (but not strictly) bounded between zero and one, with larger values corresponding to better predictions. Finally, to assess the statistical significance of the predictions vis-a-vis a null hypothesis of no predictive power, we computed the p-value associated with a Kendall's tau test comparing the predictions to the true values. Ranked importance of different features from the RF analysis are provided in Table S6.

For classification, the goal is to predict a binary outcome of whether a bNAb will give detectable neutralization responses against a given sequence or not. The most intuitive measurement of performance is the accuracy, i.e., the fraction of sequences for which the prediction is correct. In some cases, however, simply predicting all positives or all negatives will give a very high accuracy score (e.g. 10E8 neutralizes at some level 98% of the viruses tested), so machine learning prediction is highly accurate, but it is not much better than just predicting that all Envs are positive.

We tested 3 comparisons of particular interest to highlight the importance of signatures in enabling accurate predictions. First, we used only signature sites that were in contact residues versus the complete signatures; the complete list was favored for regression predictions (see Results). Second, we compared using signature sites sequence features as inputs, to using the mRMR approach to filter out the 100 most informative sites (Hepler et al., 2014, Peng et al., 2005). As noted in the Results, complete signatures yielded the most accurate predictions for regression, but there was no clear preference for classification. Before switching to our own mRMR-RF code, to make sure our approach was at least comparable in prediction accuracy to the previously published IDEpi classification code (Hepler et al., 2014), we compared the prediction accuracy of the two methods using 10-fold cross-validation for M group analysis, and also comparing the accuracy for the C clade holdout. Our implementation of the mRMR-RF approach was generally comparable to IDEpi (Table S5), although for a small number of antibodies our error was substantially lower (e.g. VRC01 and 10E8). As a final comparison, because most published computational studies present only a very small number of amino acid signatures for each Ab, we sought to determine whether reducing the number of features to only the strongest features improved the scores, so we limited the Random Forest to include only the 3 most informative features. When comparing this restricted set to the full signature pattern, we found the restricted set not only did not improve classification or regression scores, it often made them much worse.

#### Vaccine Immune Response Comparisons

##### Analysis of SET vaccines neutralization data

Neutralization data were analyzed using the R package (www.r-project.org) and GraphPad Prism version 6.00 software (GraphPad Software, San Diego California USA, www.graphpad.com”).

We considered ID_50_ titers positive if they were at least 10 above background:

Cutoff 1: Response = Post, if Post > MuLV + 10; 10 otherwise, where ‘Post’ is post-vaccination sera ID50 (4 weeks-post last vaccination), ‘MuLV’ is the background level for an animal-matched MuLV negative control (4 weeks-post last vaccination) (Bricault et al, 2015), and “10” considered below the level of detection. We compared the statistical results presented in Figure 6 with the outcome using alternative cutoffs 2 and 3:
Cutoff 2: Response = Post - MuLV, if Post-MuLV > 10, 10 otherwise,
Cutoff 3: Response = Post, if Post >3^∗^MuLV, 10 otherwise,

and found the results obtained using Cutoff 2 and Cutoff 3 were consistent with the results obtained with the cutoff 1 when comparing vaccine groups, so Cutoff 1 is shown.

The breadth of neutralization response (detected vs not-detected) was assessed by counting for each animal the proportion of pseudoviruses with detectable neutralization and then applying the two-sided Wilcoxon rank-sum test to compare the differences in distributions of responses per animal between the 459C WT and the V2-SET vaccines.

The differences in the magnitude of responses between V2-SET vaccines and the 459C WT alone were assessed by a nonparametric permutation test following the strategy described in (Parrish et al., 2013). Briefly, this test compares the medians of responses elicited by the 459C WT and the given V2-SET vaccine in the observed data and in the 10,000 randomized sets of resampled data where the vaccine category is randomly reassigned between vaccinated animals. The fraction of occurrences of median differences in the randomized data that are equal to or less than that observed median differences in the actual data provides an estimate of the probability for observing the actual results by the chance alone.

### Data and Software Availability

In this study we have created a catalog of new signature sites, also including those that were defined previously, and created 3 web-based tools to facilitate future analyses: *GenSig* enables users to implement their own phylogenetically corrected signature analysis, *FilteredForests* enables machine learning predictions using either bNAb signature-based or mRMR prefilters, and we have automated neutralization signature predictions for new bNAb neutralization panels as they are incorporated into the Los Alamos HIV database *CATNAP* NAb interface (Yoon et al., 2015).

#### GenSig

We have developed a Signature Tool web interface, *GenSig*, at the Los Alamos HIV database: https://www.hiv.lanl.gov/content/sequence/GENETICSIGNATURES/gs.html. It can work on any phenotype file given in conjunction with a codon-aligned nucleotide alignment of a protein coding region of moderate size (<1000 gene sequences) -- the tool is not specific for HIV-1 and neutralization data. If, however, an input alignment is an HIV-1 gene alignment with the HXB2 reference sequence is included, the numbering of the output will be given according to HIV standardized HXB2 numbering.

#### CATNAP Enhancement

The HIV-1 pseudovirus sequence data for the viral panels and previously published GenBank accession numbers are all already available through the Los Alamos HIV Database *CATNAP* tool, which we maintain. All new neutralization data used in this study will be integrated into the *CATNAP* tool at the time of publication: https://www.hiv.lanl.gov/catnap. Since new HIV-1 bNAbs are continuously being added to the literature, and new neutralization panel data is regularly entered into the Los Alamos database *CATNAP* tool (Yoon et al., 2015) for comparative analysis (https://www.hiv.lanl.gov/components/sequence/HIV/neutralization/index.html), we have added an automated signature analysis feature to update signatures for new data as it accrues.

#### FilteredForests

A web interface to run our sequence-based prefilters for machine learning predictions of bNAb sensitivity automatically coupled to RF code from the Python scikit-learn package, (Pedregosa et al., 2011) is called *FilteredForests* code. One can generate their own signature-based prefilters or use mRMR (Peng et al., 2005) to generate from a sequence alignment input files for the RF machine learning codes ExtraTreesRegressor and ExtraTreesClassifer. This web interface is available at: https://www.hiv.lanl.gov/content/sequence/FLTFORESTS/fltforests.html

The code is available at:https://github.com/hivdb-lanl/FilteredForests

#### bNAb signature information access

To enable comparisons to sites of interest for particular bNAbs identified here, to sites identified in the previously published literature, all signatures identified in this study have been incorporated into the Los Alamos HIV Immunology Databases. The signature information will be included and accessible through three Los Alamos database tools: a simple spread sheet that is an overview of many of the key findings from the literature, that allows comparisons of findings for all sites (rows) in Env across many antibody studies, organized by paper and/or antibody (columns) (https://www.hiv.lanl.gov/content/immunology/neutralizing_ab_resources.html). The signatures will also be accessible through the relational database we have built for searching bNAb characteristics (Neutralizing Antibody Contexts and Features, (www.hiv.lanl.gov/components/sequence/HIV/featuredb/search/env_ab_search_pub.comp); and the Genome Browser, which allows users to interactively explore functional domains and sites relevant to antibodies across Env (www.hiv.lanl.gov/content/sequence/genome_browser/browser.html).”

## Supplemental Information

Document S1. Figures S1–S7 and Tables S1, S2, S4, S5, and S7

Table S3. All Signature Tables for All Antibodies and Datasets, Related to Figures 3 and 4 A-D. Summary tables organized by site, amino acids, and antibodies, and providing an overview of all signature sites found for a given antibody class, across all 4 datasets. Separate tabs are provided for each bNAb class studied (tabs A-D). Sites within hypervariable regions are excluded. The antibodies included in each of the four primary datasets are listed at the top. Sites are included as a signature if at least one antibody in one dataset had a phylogenetically corrected signature site with a q-value of < 0.2 (Table S3 tabs E-H) or if the signature site was in a contact residue. If either of these criteria was met, the site was deemed of interest, and all simple (without a phylogenetic correction) Fisher’s exact associations with a q-value < 0.2 were then included and tracked for that site. Complete details of statistical support for each signature are included in Table S3 tabs E-H (phylogenetic corrected signatures only, organized by antibody) and S3 tabs I-L (all simple associations for sites of interest, organized by site). Antibodies with significant associations after a phylogenetic correction (from Table S3E-H) are bold. The positions are based on HXB2 numbering. If the site is known to be in a contact site for an antibody in the class, based on structural studies, the position number is colored and bold. Amino acids significantly associated with bNAb resistance are colored red, those that are associated with sensitivity are blue. Glycosylation site patterns were tracked, and a PNGS motif is noted as “NxST”. Note that lack of an association in a particular dataset does not mean that the association in another is not valid for a given antibody, it may simply mean that a given dataset did not have enough power to resolve the association statistically. If a particular site was associated with bNAbs sensitivity/resistance in multiple datasets, for more than one bNAb in a class, or was located in a known contact residue, it was deemed likely to be robust as it was supported by several lines of evidence (HXB2 position numbers of such sites are highlighted in bold). These more robust associations were used as a basis for the signature profiles in Figure 3 in the main body of the text and for structural mapping. Contradictory signatures, associated with sensitivity to some antibodies in a class, but resistance to others, are highlighted in tan. Tabs: A.V3 bNAb signature summary. Contact residues are indicated in blue in the Position column. B. V2 bNAb signature summary. Contact residues are indicated in green in the Position column. C. CD4bs bNAb signature summary. The HXB2 positions of contact residues as described in the legend to Table S4C are indicated by lavender text. Most CD4bs bNAbs are VH1-2 or VH-46 using and are noted in black. The sites that were used for the illustration in the main paper in Figure 3 focus on VH1-2 or VH-46 usage CD4bs bNAb signatures. Antibody names in red include CDRH3 dominated antibodies (Zhou et al., 2015) CH103, HJ16, VRC13, VRC16, and IgG1b12, plus two VH1-2 antibodies, VRC03 and VRC06, that tended to track with CDRH3 bNAbs in terms of signature associations. These antibodies generally have less breadth and often have contradictory signatures relative to most VH1-2 or VH-46 using CD4bs bNAbs. D. **D.** MPER bNAb signature summary. Contact residues are indicated in the Position in dark brown for 2F5, light brown for 4E10/10E8/DH511. E-H. Phylogenetically corrected signatures, organized by antibody**.** These tables list the statistical support for all phylogenetically corrected amino acid signatures with q-values <0.2 for each antibody studied, organized by antibody class and antibody, providing details regarding signatures statistics. Separate tabs are provided for each bNAb class studied (S3 E-H). The phylogenetic correction compares to the neutralization phenotype the amino acids in sites that are unchanging to those that change between a taxon and its most recent ancestral node as estimated using a maximum likelihood tree. The column headings are as follows. The “*Table*” columns are T2 and T3, for Table 2 and 3. These tables are phylogenetically corrected signatures. A detailed example of how to read each kind of table is provided in each spreadsheet. If the signature analysis was testing for N-linked glycosylation sites rather than simple amino acids, it is indicated as a “glycan” table, e*.*g*.* T2glycan. The *Dataset* is either: the first (1) or second (2) completely independent M group datasets, the C clade dataset (3), or the larger M group data (4), shown in Figure S1. The *cutoff* is the cutoff used for the input phenotype that gave the highest degree of statistical support for a particular signature. *PosNeg* means the data was broken down between positive, i.e. a detected IC_50_, and negative, with IC_50_ above the threshold of detection. Data was also broken down by above or below the median titer, and upper and lower quartiles. The *HXB2 pos* is the position in the alignment based on HXB2 numbering. The *test AA* is the amino acid that was being evaluated in the position; only those with a q-value <0.2 are included. Also, we excluded a small number of cases when phylogenetic association were not also supported by a simple uncorrected association. If *blue*, its presence was associated with enhanced sensitivity, if *red*, with resistance. NxST is an abbreviation to refer to an intact N-linked glycosylation site motif. *Antibody* is the name of the bNAb. *P-value*, *q-value* and *Odds Ratio* are all summary statistics, that are based on the 2x2 contingency tables that are outlined as r1c1, r1c2, r2c1, r2c2, where r stands for row, c for column. See T2 and T3 examples of how to read the contingency tables for the two distinct types of corrections, change towards or away from a given AA. P-values are based on a 2-sided Fisher’s exact test, the q-values were based on all signature p-values. We also list ranked AAs, based on the most informative AAs for our machine learning implementation of *Regression* (predicting IC_50_ values from sequences, Table S4) and *Classification* (predicting positive/negative IC_50_ values from sequences, Table S5). These are listed by rank of importance, followed by the HXB2 position and the amino acid, or a dash if a deletion is important. The next columns show results from other signature analysis papers, including just the associations that were directly reported and readily retrieved from the primary publications. The association is listed alongside signature amino acid we have identified when possible. From West et al. (West et al., 2013) we report associations given as the antibody name, the amino acid association, and the position. Chuang et al. summarizes the published NEP predictions for the 10 highest rank scores (Chuang et al., 2013, Chuang et al., 2014). Hepler et al. (Hepler et al., 2014) associations are from the primary publication using IDEPI. Ferguson et al.’s results are listed as compressed sensing results (given as amino acid and position), ensemble support including mutual information (given as yes or no) and experimental support (given as yes or ND for not done). E. V3 bNAb phylogenetically corrected signatures and statistical support**.** V3 bNAbs contacts are highlighted in blue, based on two Env bound structures: PGT128 (301, 303, 304, 323-327, 332) (Pejchal et al., 2011) and PGT135 (295, 301, 330, 332, 339, 373, 384, 386, 389, 392, 409, 415, 417-419). The bNAb 2G12 is also included here, even though its epitope is very distinct from the other antibodies included this table. F. V2 bNAb phylogenetically corrected signatures and statistical support. V2 bNAbs contacts are highlighted in green, based on structural contacts for PG9 (McLellan et al., 2011) (contact signatures are: PNGS at N156-158, PNGS at N160-162, 165, 167-171, 173). G. CD4bs bNAb phylogenetically corrected signatures and statistical support. Representative CD4bs bNAbs contacts are highlighted in lavender and are based on an inclusive summary of structural contacts defined for CD4 and CD4bs bNAbs. These contact regions include the following HXB2 positions: V1 proximal: 97-99, 122-129, 196-198, 207, loop D: 275-283, 308, 318, CD4 binding loop: 364-374, beta20/21: 425-432, beta23: 455-459, V5 hypervariable region 460-465, beta 24: 466-477. Sites within the V5 hypervariable region are not included in the signature analysis, even though they can interact directly with the CD4bs bNAbs, due to alignment uncertainty. Contacts regions for CD4bs bNAbs and CD4 were defined based on the following data: CD4 contacts (Wu et al., 2011, Zhou et al., 2010): 124-127, 196-198, 279-283, 365-370, 374, 425-432, 455-461, 469-477; VRC01 contacts (Wu et al., 2011, Zhou et al., 2010): 97, 122, 276, 278-283, 365-368, 371, 427-430, 455-476; IgG1b12 contacts (Zhou et al., 2007): 267, 268, 280, 281, 364-373, 395, 397, 417-419, 430-432, 453-458; NIH45-46 contacts: 97-99, 102, 122-124, 127-128, 276, 427, 430-432, 455-480; 3BNC117 contacts (Scheid et al., 2016): 124, 198, 207, 275-276, 278-282, 308, 318, 365-368, 371, 428-430, 455-462, 469, 473. H**.** MPER bNAb phylogenetically corrected signatures and statistical support. 2F5 and other MPER antibodies bind to distinct regions. The 2F5 epitope is focused on the sites 662-668 (the HXB2 sequence ELDKWAS) (Ofek et al., 2004) and is highlighted in dark brown. 4E10 is focused on the sites 671-676 (NWFDIT) (Cardoso et al., 2005) and the broader more potent 10E8 extends further out, 671-683, NWFDISNWLWYIK with contacts including positions 671-673 and 676 (Huang et al., 2012). The DH511 lineage binds to an epitope similar to 10E8 (Williams et al., 2017). The 10E8/4E10/DH511 epitopes are highlighted in light brown. I-M. Amino acid associations with bNAb sensitivity in sites of interest, organized by site**.** Separate tabs are provided for each bNAb class studied (I-L). We include sites here after a hypothesis has been raised that a site is of interest: if a site is statistically significant after a phylogenetic correction, i*.*e*.* included in Table S4 for any antibody in a class, or if it is directly in a bNAb contact residue, it is considered of interest for the full bNAb class. Next, Fisher’s test for associations of all amino acids at that site with a q < 0.2 for all antibodies in that class are listed. “Table 1” (T1) is a contingency table for a simple Fisher’s exact test based on the amino acid under consideration in all of the sequences in the set and their IC_50_ breakdowns, with no phylogenetic correction applied. This table uses the same columns headings defined in Table S3 E-H, but the table rows are organized by HXB2 position instead of by antibody. An example of how to read the contingency table is provided in each data spreadsheet. We then list importance-ranked signatures based on our machine learning implementation for regression (levels of sensitivity, Table S4) and classification (positive/negative Table S5), followed by columns that show previously published signatures for antibodies in our study. The association is listed alongside signature sites we have identified, if the earlier finding is also supported by our analyses, or in a separate row if we did not find support for the reported association in our analyses. I. Simple signatures associated with V3 bNAb sensitivity in sites deemed of interest. Contact residues are highlighted in blue. J. Simple signatures associated with V2 bNAb sensitivity in sites deemed of interest. Contact residues are highlighted in green. K. Simple signatures associated with CD4bs bNAb sensitivity in sites deemed of interest. Contact residues are highlighted in lavender. L. Additional signatures associated with MPER bNAb sensitivity in sites deemed of interest. 2F5 Contact residues are highlighted in dark brown, other MPER antibody contacts in light brown. M. Results of applying a Wilcoxon test to CD4bs antibodies from dataset 4. Our signature bioinformatics tool provides an option to use a Wilcoxon rank sum test to compare the IC_50_ score distributions, in the presence or absence of a give amino acid at a given position, and we tested its performance for dataset 4. For most bNAb classes, this yielded fewer signatures and less significant results than the Fisher’s exact test for the same data, but CD4bs bNAbs had exceptions listed here. This table includes only cases for which the Wilcoxon yielded comparable or lower p-values than Fishers, and so adds signatures to part C. The number of pseudoviruses with (AA) and without (AA) the signature amino acid, and the median value of the IC_50_ data for that set of pseudoviruses, are noted for each antibody. N-R. Hypervariable region characteristic signatures. Excel spread sheet, supporting Figure 2B**.** The statistics of associations between of V1, V2, V4, and V5 hypervariable region characteristics and IC_50_ scores for each antibody organized by antibody class. Separate tabs are provided for each bNAb class studied (N-Q); and tab R provides a key showing the boundaries of hypervariable regions relative to HXB2. Our analyses considered characteristics of the full-length variable loops, the more narrowly defined hypervariable segment (in bold lettering) that cannot be reliably aligned, and the sum of behaviors across both V1 and V2; we included 10 regions in all, in our search for correlates with bNAb potency. The characteristics of combined V1 + V2 regions were often a stronger correlate of bNAb sensitivity than of either V1 or V2 considered in isolation. Only characteristics that had at least one association based on Kendall’s tau with a q-value < 0.2 are captured in this table; once that level of significance was found, the characteristic is considered of potential interest, and all associations between a characteristic and antibodies of the same class are shown. Dataset 3 (C clade) and dataset 4 (M group) are included here, as they are the largest datasets and best powered. If only the hypervariable region of a loop was used for the analysis, it is indicated by an “h”, for example V1 means the entire V1 loops was used, V1h means only the hypervariable region. If two highly related characteristics were identified as statistically of interest, like V1 and V1h, only the most significant relationship of the two was retained. The characteristics are: *Charge*, the net charge of the amino acids spanning the region considered (summing over each region such that an Arg, Lys and His contribute +1, Glu and Asp -1); *Length,* the number of amino acids in the region based on the HXB2 boundaries; and *Glycos,* the number of PNGSs within the boundaries of the region under consideration. Kendall’s tau was used to calculate p-values. N. V loop and hypervariable region characteristics associated with V3 bNAb sensitivity. Excluding negative IC_50_ responses enhanced correlations, so the impact of loop length on potency among just positive responders was more dramatic. This is likely because viruses are completely resistant when the PNGS at N332 is lost, regardless of loop characteristics. Thus, even viruses with favorable loop characteristics will be negative if the PNGS at N332 is absent, complicating resolution of other characteristics of importance. O. V loop and hypervariable region characteristics associated with V2 bNAb sensitivity. P. V loop and hypervariable region characteristics associated with CD4bs bNAb sensitivity. Q. V loop and hypervariable region characteristics associated with MPER bNAb sensitivity Increasing numbers of PNGS’s in the V1 loop correlated with enhanced sensitivity to 10E8, and with other MPER bNAbs to a lesser extent. This was the only case where increasing the size of the variable region was associated with increased bNAb sensitivity. R. Hypervariable region boundaries are relative to the HXB2 reference strain V loop sequences. Hypervariable regions are highlighted in bold and red and are subregions of the full variable region loops.

Table S6. Machine Learning Informative Feature Contributions, Related to Figure 4 This table presents ranked signature features that were among the top 10 most informative sites for different antibodies of different classes. It has four sections, one for each antibody class, and each section lists the most important signature features for Classification and Regression predictions for each antibody tested from dataset 4. On the right are summaries of the number of times recurrent features are found. Different shades highlight different V loop characteristics. Bold indicates sites that were repeatedly informative for a number of antibodies (at least 4/11 times for CD4bs bNAbs, 3/6 times for V2 bNAbs, 3/6 times for V3 bNAbs, and 2/3 for MPER bNAbs). Blue indicates signature positions within epitope contact regions, and black indicates positions outside the contact region. NxST N332 and NxST N160 indicate a PNGS. Clades were only rarely among the most informative data for prediction.

Document S2. Article plus Supplemental Information
