# HIV-1 Neutralizing Antibody Signatures and Application to Epitope-Targeted Vaccine Design

**DOI:** 10.1016/j.chom.2019.07.016

**Published:** 2019-08-14

**Authors:** Christine A. Bricault, Karina Yusim, Michael S. Seaman, Hyejin Yoon, James Theiler, Elena E. Giorgi, Kshitij Wagh, Maxwell Theiler, Peter Hraber, Jennifer P. Macke, Edward F. Kreider, Gerald H. Learn, Beatrice H. Hahn, Johannes F. Scheid, James M. Kovacs, Jennifer L. Shields, Christy L. Lavine, Fadi Ghantous, Michael Rist, Madeleine G. Bayne, George H. Neubauer, Katherine McMahan, Hanqin Peng, Coraline Chéneau, Jennifer J. Jones, Jie Zeng, Christina Ochsenbauer, Joseph P. Nkolola, Kathryn E. Stephenson, Bing Chen, S. Gnanakaran, Mattia Bonsignori, LaTonya D. Williams, Barton F. Haynes, Nicole Doria-Rose, John R. Mascola, David C. Montefiori, Dan H. Barouch, Bette Korber

## Main Text

(Cell Host & Microbe *25*, 59–72.e1–e8, January 9, 2019)

In Table S3 of the originally published version of this article, details regarding the statistical support for certain signatures were inadvertently omitted by the authors. An updated version of Table S3 is now provided online. The associated legend remains unchanged, and the updating of Table S3 does not impact any conclusions of the study.

