# Supplementary material for: HIV-1 Neutralizing Antibody Signatures and Application to Epitope-Targeted Vaccine Design
Source: Cell Host Microbe. 2019 Jan 9;25(1):59–72.e8. doi: 10.1016/j.chom.2018.12.001 (PMC6331341; doi:10.1016/j.chom.2018.12.001)
Supplement: Document S1. Figures S1–S7 and Tables S1, S2, S4, S5, and S7 [file mmc1.pdf]

Cell Host & Microbe, Volume *25*

Supplemental Information

HIV-1 Neutralizing Antibody Signatures

and Application to Epitope-Targeted Vaccine Design

Christine A. Bricault, Karina Yusim, Michael S. Seaman, Hyejin Yoon, James Theiler, Elena E. Giorgi, Kshitij Wagh, Maxwell Theiler, Peter Hraber, Jennifer P. Macke, Edward F. Kreider, Gerald H. Learn, Beatrice H. Hahn, Johannes F. Scheid, James M. Kovacs, Jennifer L. Shields, Christy L. Lavine, Fadi Ghantous, Michael Rist, Madeleine G. Bayne, George H. Neubauer, Katherine McMahan, Hanqin Peng, Coraline Chéneau, Jennifer J. Jones, Jie Zeng, Christina Ochsenbauer, Joseph P. Nkolola, Kathryn E. Stephenson, Bing Chen, S. Gnanakaran, Mattia Bonsignori, LaTonya D. Williams, Barton F. Haynes, Nicole Doria-Rose, John R. Mascola, David C. Montefiori, Dan H. Barouch, and Bette Korber

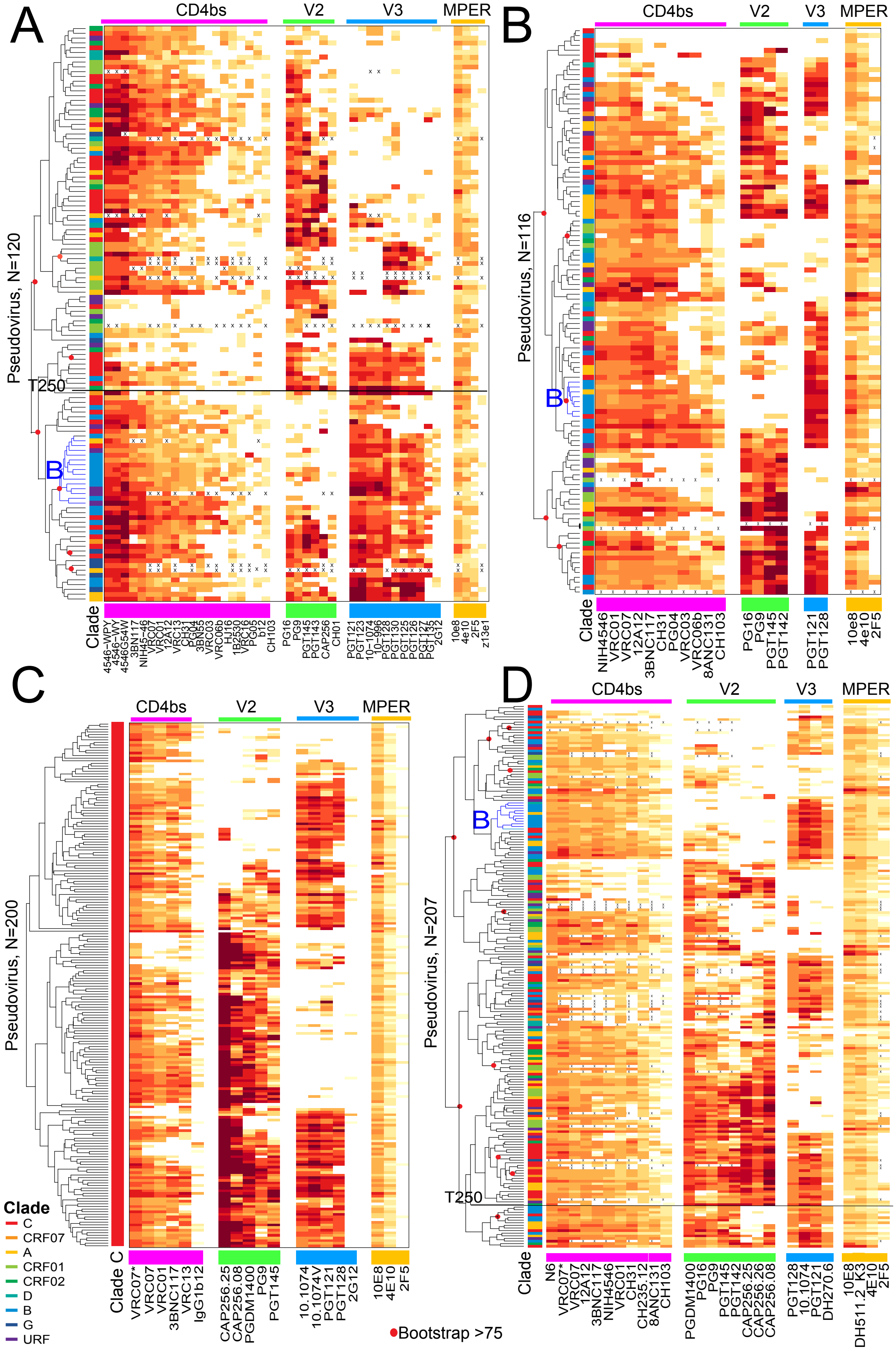

A
CD4bs
V2
V3
MPER
Pseudovirus, N=120
T250
B
Clade
B
CD4bs
V2
V3
MPER
Pseudovirus, N=116
B
Clade
NIH4546
VRC01
VRC07
12A12
3BNC117
CH31
PG04
VRC03
VRC06b
8ANC131
CH103
PG16
PG9
PGT145
PGT142
PGT121
PGT128
10e8
4e10
2F5
C
CD4bs
V2
V3
MPER
Pseudovirus, N=200
Clade C
VRC07*
VRC07
VRC01
3BNC117
VRC13
IgG1b12
CAP256.25
CAP256.08
PGDM1400
PG9
PGT145
10.1074
10.1074V
PGT121
PGT128
2G12
10E8
4E10
2F5
Clade
C
CRF07
A
CRF01
CRF02
D
B
G
URF
Bootstrap >75
D
CD4bs
V2
V3
MPER
B
Pseudovirus, N=207
T250
Clade
N6
VRC07*
VRC07
12A12
3BNC117
NIH4546
VRC01
CH31
CH235.12
8ANC131
CH103
PGDM1400
PG16
PG9
PGT145
PGT142
CAP256.25
CAP256.26
CAP256.08
PGT128
10.1074
PGT121
DH270.6
10E8
DH511.2_K3
4E10
2F5

Fig. S1. Heatmaps displaying IC_{50} values of bNAbs and pseudoviruses used in datasets 1-4, the foundational data for Figs. 1-3. Heatmaps displaying IC_{50} values of bNAbs and pseudoviruses used in datasets 1-4, the foundational data for Figs. 1-3. This figure illustrates the extent and nature of the data used for this study, and shared patterns of sensitivity and resistance across antibody classes. **Panels A-D correspond to datasets 1-4, respectively.** Antibodies are arranged by class, indicated across the top of each heat map (CD4bs, magenta; V2, green; V3, blue; and MPER, gold). Neutralization IC_{50} values ranging from low (10-50 ug/ml) to high potency (< 0.0001 ug-ml) are indicated by light yellow through deep red. White indicates an IC_{50} above the threshold of detection (generally 50 ug/ml, but occasionally when antibodies were in limited supply, lesser maximum concentrations were used). The pseudovirus clades are indicated by the colored bars on the left of each heatmap; panel C includes only C clade viruses. A small black "x" indicates no available data. Pseudoviruses are clustered according to like-behavior against the antibody panel according to the dendrogram on the left of each map. Clusters of Envs with bootstrap values >75% are rare, and are indicated as red dots on the dendrograms. There is a recurrent bootstrap-supported cluster enriched for B clade viruses, and indicated by a blue B in panels A, B, and D; the pattern associated with this cluster was V3 sensitivity and V2 bNAb resistance. In contrast, CRF01 viruses are resistant to most V3 bNAbs, and sensitive V2 bNAbs; the statistics of these patterns are explored in detail in later sections of this paper. The antibody 2G12, an older glycan bNAb with limited breadth (Trkola et al., 1996), was grouped with the V3 bNAbs because it includes the PNGS N322 glycan in its epitope; however, because it is so distinctive, it is not included in V3 bNAb summaries. Finally, the virus CRF02_T250.4 is highlighted in parts (A) and (D). T250.4 is extremely sensitive to V2 and V3 bNAbs, but resistant to CD4bs bNAbs). It has hypervariable region characteristics associated with high sensitivity to both V2 and V3 antibodies, so the V1 and V2 loops from CRF02_T250.4 were incorporated into our V2 and V3 SET vaccines (Fig. S6). CRF02_T250.4 is a CRF02 and so subtype A in Env (GenBank accession number EU513189). In panels C and D, VRC07* is an abbreviation for VRC07-523LS. Datasets 1 and 2 share many antibodies, but the pseudoviruses were selected to be completely independent with 120 and 116 viruses included, respectively. Datasets 3 and 4 are larger, with 200 and 207 pseudoviruses, but each contains subsets of viruses that overlap with the other sets. In dataset 4 (D) 208 pseudoviruses were tested, but the full Env gene sequence of one them, 426.c (McGuire et al., 2013) was uncertain, so only 207 viruses were included here. IC_{50} and IC_{80} values were both available, but as IC_{50} is typically a more sensitive indicator for defining signatures, due to having less censored data, it was used here and throughout the paper.

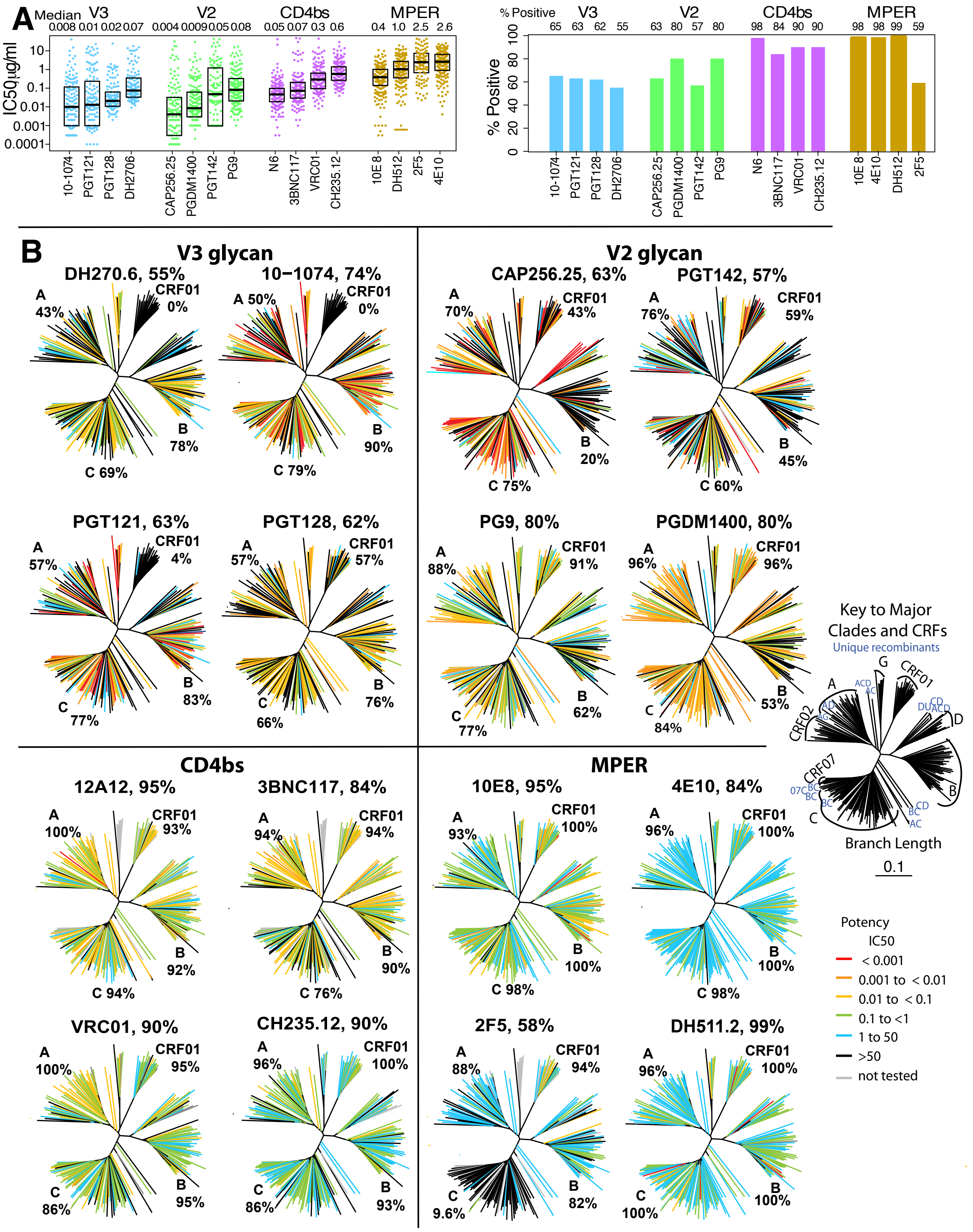
A
Median
V3
0.008 0.01 0.02 0.07
V2
0.004 0.009 0.05 0.08
CD4bs
0.05 0.07 0.3 0.6
MPER
0.4 1.0 2.5 2.6
IC50μg/ml
10
1
0.1
0.01
0.001
0.0001
10-1074
PGT121
PGT128
DH2706
CAP256.25
PGDM1400
PGT142
PG9
N6
3BNC117
VRC01
CH235.12
10E 8
DH512
2F5
4E10
% Positive
V3
65 63 62 55
V2
63 80 57 80
CD4bs
98 84 90 90
MPER
98 98 99 59
% Positive
0 20 40 60 80 100
10-1074
PGT121
PGT128
DH2706
CAP256.25
PGDM1400
PGT142
PG9
N6
3BNC117
VRC01
CH235.12
10E 8
4E10
DH512
2F5
B
V3 glycan
DH270.6, 55%
A 43%
CRF01 0%
B 78%
C 69%
10−1074, 74%
A 50%
CRF01 0%
B 90%
C 79%
PGT121, 63%
A 57%
CRF01 4%
B 83%
C 77%
PGT128, 62%
A 57%
CRF01 57%
B 76%
C 66%
V2 glycan
CAP256.25, 63%
A 70%
CRF01 43%
B 20%
C 75%
PGT142, 57%
A 76%
CRF01 59%
B 45%
C 60%
PG9, 80%
A 88%
CRF01 91%
B 62%
C 77%
PGDM1400, 80%
A 96%
CRF01 96%
B 53%
C 84%
Key to Major Clades and CRFs
Unique recombinants
G
CRF01
A
CRF02
D
CRF07
B
C
Branch Length
0.1
CD4bs
12A12, 95%
A 100%
CRF01 93%
B 92%
C 94%
3BNC117, 84%
A 94%
CRF01 94%
B 90%
C 76%
VRC01, 90%
A 100%
CRF01 95%
B 95%
C 86%
CH235.12, 90%
A 96%
CRF01 100%
B 93%
C 86%
MPER
10E8, 95%
A 93%
CRF01 100%
B 100%
C 98%
4E10, 84%
A 96%
CRF01 100%
B 100%
C 98%
2F5, 58%
A 88%
CRF01 94%
B 82%
C 9.6%
DH511.2, 99%
A 96%
CRF01 100%
B 100%
C 100%
Potency
IC50
< 0.001
0.001 to < 0.01
0.01 to < 0.1
0.1 to <1
1 to 50
>50
not tested

Fig. S2. A comparison of breadth and potency of bNAb classes using four representative bNAbs from each class, related to Figs. 1-2. The IC_{50} data for these figure was from the most comprehensive multi-clade dataset we studied, dataset 4. (**A) Overall breadth and potency.** The left hand figure illustrates bNAb potency against sensitive strains, and the right shows their breadth in terms of frequency of detectable IC_{50} neutralization responses. This IC_{50} data for this graph was from the most comprehensive multi-clade dataset we studied, dataset 4. (**B) Phylogenetic associations with potency for 4 representative antibodies from each of the 4 classes.** Major clades and circulating recombinant forms (CRFs) are indicated on the detailed black "key" maximum likelihood tree on the far right. CRF01 is common in Asia; it is an AE recombinant that is mostly E in Env. CRF07 is common in China, a BC recombinant, mostly C in Env. CRF02 is common in West Africa, an AG recombinant, mostly A in Env. Clade B dominates the north American and European epidemics, Clade C southern Africa and India, and A is common in Central Africa. Unique recombinants are common among natural isolates, and some are included in this panel; their parental clades are indicated in blue text in the "key" tree. Branch colors in the other 16 trees indicate IC_{50} values, the strength of antibody responses – from red (potent) to blue (weak); black is not detected (see key). The percentage of viruses with a detectable response for 4 major clades that are best sampled, A, B, C and CRF01, are indicated for each clade. 4 representative antibodies for each class are shown. The top left shows V3 glycan antibodies. CRF01 has extreme resistance to these bNAbs. PGT128 is the only one of these four bNAbs able to neutralize CRF01 viruses. A Fisher's exact test comparison of the fraction of detectable neutralized viruses in CRF01 versus other clades show this is highly significant for DH270.6 ($p= 4 \times 10^{-10}$), 10-1074 ($p= 9 \times 10^{-14}$), and PDT121 ($p=4 \times 10^{-11}$), but the effect was not significate for PGT128. The top right shows V2 glycan antibodies. Clade B is highly resistant to CAP256.VRC26 (called CAP256 here) lineage antibodies (Doria-Rose et al., 2015); relative clade B resistance was also found for other V2g bNAbs, including PG9 and PGDM1400, PGT142. The Fisher's exact test p-values for B clade versus others were CAP256.25 ($p = 6 \times 10^{-12}$), PG9 ($p = 0.003$), PGDM1400 ($p = 0.00001$), and PGT142 was not significantly different in the B clade, but this seemed to be due more to a loss of reactivity in other clades than a relative gain within the B clade. There was also a C clade effect found only for CAP256.VRC26 lineage antibodies, in that C clade viruses were more sensitive. CAP256.25 had Fisher's test p-value for positive/negative was p=0.007, odds ratio 2.3). The results were more dramatic for CAP256.08, ($p = 0.000095$, OR = 3.4), and also evident for CAP256.26 ($p=0.007$, OR = 2.8). The bottom left shows CD4bs antibodies. All CD4bs bNAbs are very broad, but there are two clade effects worth highlighting. Responses to clade A were more potent. There is also a general tendency for these antibodies to have limited breadth and potency against C clade; 3BNC117 is most impacted, but VRC01 is as well. A Fisher's exact test comparison of the fraction of detectable neutralized viruses among C clade and CRF07, which is mostly C clade in Env, shows a trend of reduced activity (Fisher's $p = 0.037$) for 3BNC117. But if the potency of 3BNC117 is compared against C and CRF07 viruses versus others, a significant difference is observed in level of response (Wilcoxon rank sum $p = 0.00003$), and VRC01 is also shows a trend for less potent responses ($p = 0.027$); these effects are exacerbated for IC80 scores (data not shown). On the other hand, N6 and VRC07 have greater potency and breadth overall, and their potency not particularly compromised among C clade viruses. The bottom right shows MPER antibodies. 2F5 has a strong clade effect, resistance in the C clade, not shared by other MPER bNAbs. Clade A has diminished sensitivity to MPER antibodies, in contrast to CD4bs bNAbs. 10E8, DH511.2, and 4E10 are all very broad; 10E8 is the most potent.

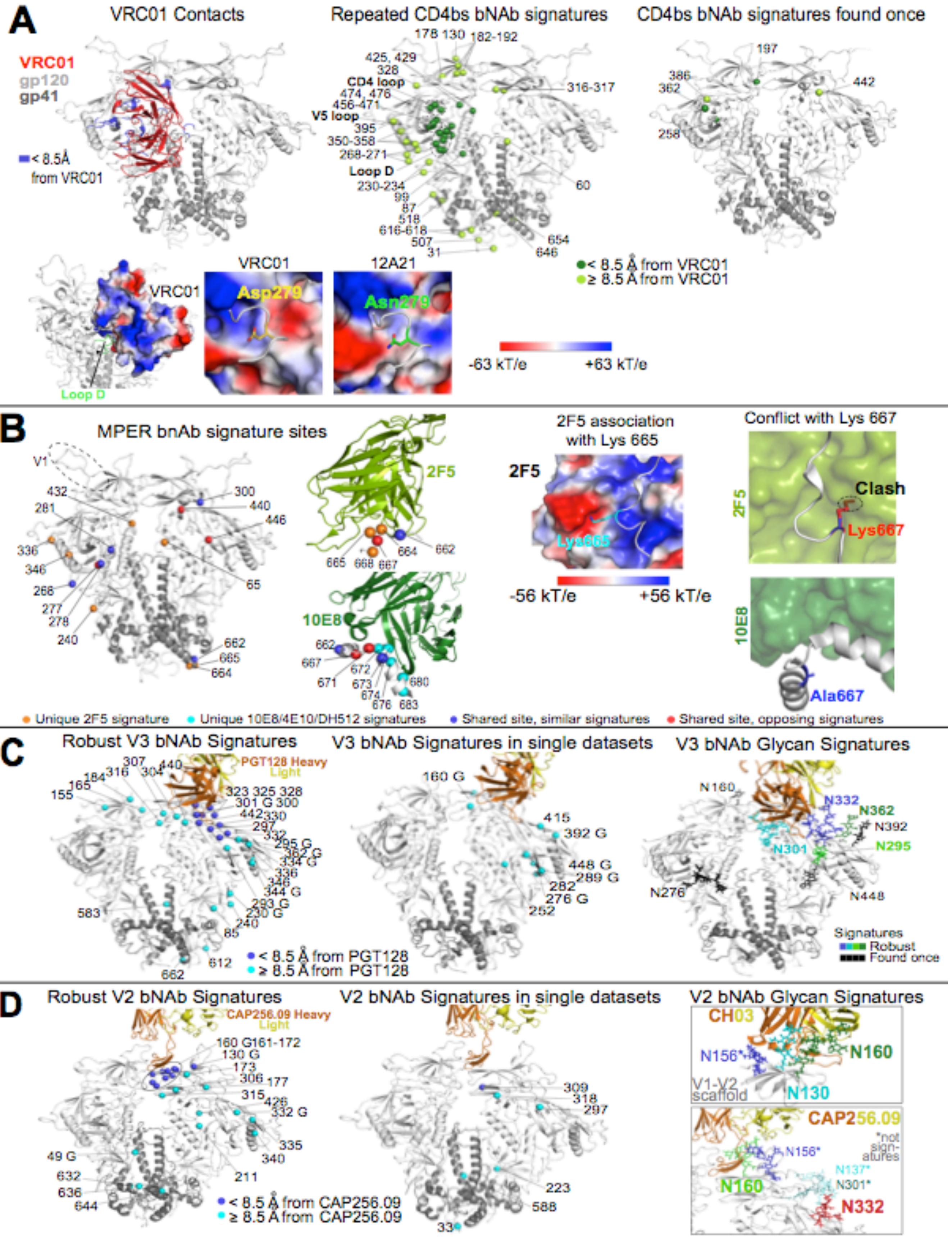
A
VRC01 Contacts
VRC01
gp120
gp41
< 8.5Å from VRC01
Repeated CD4bs bNAb signatures
178 130 182-192
425, 429
328
CD4 loop
474, 476
456-471
V5 loop
395
350-358
268-271
Loop D
230-234
99
87
518
616-618
507
31
316-317
60
654
646
CD4bs bNAb signatures found once
197
386
362
442
258
VRC01
Loop D
VRC01
12A21
Asp279
Asn279
< 8.5 Å from VRC01
≥ 8.5 Å from VRC01
-63 kT/e
+63 kT/e
B
MPER bnAb signature sites
V1
432
281
336
346
268
277
278
240
300
440
446
65
662
665
664
2F5
665 668 667
664
662
10E8
662
667
671
672
673
674
676
683
680
2F5 association with Lys 665
2F5
Lys665
-56 kT/e
+56 kT/e
Conflict with Lys 667
2F5
Clash
Lys667
10E8
Ala667
Unique 2F5 signature
Unique 10E8/4E10/DH512 signatures
Shared site, similar signatures
Shared site, opposing signatures
C
Robust V3 bNAb Signatures
PGT128 Heavy
Light
307
316
304
440
184
165
155
323 325 328
301 G 300
442
330
297
332
295 G
362 G
334 G
336
346
344 G
293 G
230 G
240
85
583
612
662
< 8.5 Å from PGT128
≥ 8.5 Å from PGT128
V3 bNAb Signatures in single datasets
160 G
415
392 G
448 G
289 G
282
276 G
252
V3 bNAb Glycan Signatures
N160
N332
N362
N392
N301
N295
N276
N448
Signatures
Robust
Found once
D
Robust V2 bNAb Signatures
CAP256.09 Heavy
Light
160 G161-172
130 G
173
306
177
315
426
332 G
335
340
211
49 G
632
636
644
< 8.5 Å from CAP256.09
≥ 8.5 Å from CAP256.09
V2 bNAb Signatures in single datasets
309
318
297
223
588
33
V2 bNAb Glycan Signatures
CH03
N156*
N160
V1-V2 scaffold
N130
CAP256.09
*not signatures
N156*
N137*
N301*
N160
N332

Fig. S3. Structural mapping and analyses of bNAb signatures, supporting Fig. 3 in the main text.

(A) CD4bs bNAb signatures. The top left panel shows co-crystal structure of VRC01 bound to Env trimer (PDB: 5FYJ, (Stewart-Jones et al., 2016)), with VRC01 contacts (<8.5Å) highlighted in blue. The top row center and right panels show signature sites (spheres) mapped on the Env crystal structure, color-coded according to their proximity to VRC01. The center panel shows the most robust signatures supported in multiple datasets, and the right shows signatures found in only one dataset. The panels in the bottom row show the interaction of site 279 with VRC01 and 12A21, a site of contradictory signatures – D279 was associated with sensitivity to VRC01 and resistance to 12A12 (from the same antibody lineage as 12A21). The left panel shows the location of Loop D and site 279 on the Env trimer with respect to VRC01 in a surface representation color-coded by vacuum electrostatic potential generated using Pymol (Version 1.8, Schrodinger LLC) with negatively/positively charged surfaces shown in red/blue respectively. The center and right panels are zoomed-in views of interaction of D279 with VRC01 and of N279 with 12A21 (PDB: 4JPW (Klein et al., 2013)). Negatively charged D279 interacts with a positively charged cavity on VRC01, potentially explaining why this amino acid is associated with sensitivity. However, for 12A21, N279 is closest to a negatively charged surface, which may be why D279 is not favored in antibodies of this lineage.

(B) MPER bNAb signatures. The structural mapping of signature sites (spheres) from Figure 3 on an Env trimer (left) and on MPER peptides from co-crystal structures with MPER bNAb Fabs (center left). Most of the signatures between 10E8, 4E10, and DH511 lineage antibodies were shared, and were distinct from 2F5 signatures. Thus, the signature sites are color-coded according to whether they were signatures for 2F5 (orange) or for 10E8/4E10/DH511 (cyan) exclusively. Signatures shared across all bNAbs are shown with blue or red spheres depending on whether AAs were concordantly or discordantly associated between 2F5 and other MPER bNAbs, respectively (Fig. 3D). C clade viruses are resistant to 2F5, likely in part because they often carry K667 which would cause unfavorable interactions with 2F5 (Figs. 1, S3). The center right panel shows 2F5 interaction with Lys at 665 (K665), a highly favored signature (Fig. 3). The side chain of K665 is proximal to a strongly negatively charged surface on 2F5, which can explain this preferred 2F5 signature. The right most panels show the position of signature site 667 with respect to 2F5 and 10E8, respectively. Amino acids like K667 and N667 are associated with 2F5 resistance, but are neutral for 10E8 (Table S3). The 2F5 co-crystal structure had A667 (a sensitivity signature) and to understand the impact of these other mutations, we explored modeling K667 (shown) and N667 (not shown) using the “mutagenesis” wizard in Pymol. These models indicate the most likely configuration of K667 could lead to a steric clash with the 2F5 fab (as seen by Lys side chain partially penetrating the translucent 2F5 volume), while A667 (blue) does not. While other clades generally carry Ala at 667, clade C does not, which may contribute to clade C’s resistance to 2F5. In contrast, the site at 667 is not proximal to 10E8 Fab (right panel), thus both K667 and N667 variants should be tolerated. Structures used are PDB: 1TJH (Ofek et al., 2004) for 2F5, and PDB: 4G6F (Huang et al., 2012) for 10E8.

(C) V3 bNAb signatures. Left and center panels show the amino acid signatures (spheres) mapped on an Env trimer co-crystal structure with PGT128 Fab (PDB: 5C7K) (Kong et al., 2015). Signatures are stratified according to whether they were found in multiple datasets (left) or in one dataset only (center), and according to whether or not they were within 8.5Å of the PGT128 Fab. Glycan signatures are indicated with a “G” next to them. Robust, multiple-dataset signatures were concentrated in the PGT128 contact regions as compared to the signatures found in only one dataset, but enrichment was not significant using a Fisher's exact test. The right panel shows the mapping of N-linked glycan signatures. All available glycans in the Env-PGT128 co-crystal structure that were signatures for V3 bNAbs are shown in “stick” representation; colored glycans are signatures in multiple datasets, while black glycans are signatures in only a single dataset. The glycans at positions 332 and 301 interact directly with the antibody. The glycans at positions 295 and 392 do not contact PGT128 directly, but are close to N332 (6-15Å minimum distance), and may impact glycan dynamics and processing of N332, thereby indirectly affecting binding. Other glycan signatures are far from PGT128 and key contact glycans (e.g. the glycan at N448, that is ~22-26Å away from PGT128 and N332) but may still subtly influence V3 bNAb sensitivity through an indirect mechanism. Distant glycan signatures were not supported in multiple datasets.

(D) V2 bNAb signatures. The left and center panels show V2 bNAb N-linked glycan signatures mapped on a structural model of CAP256-VRC26.09 bound to an Env trimer (Gorman et al., 2016); color-coding same as in (C). The right panel shows glycan signatures on V1/V2 scaffold bound to CH03 (PDB: 5ESV, Gorman et al., 2016) (top) and on Env-CAP256-VRC26.09 structural model (bottom). The glycans at positions N130, N156, and N160 interact with V2 bNAbs and PNGS’s at positions N130 and N160 are signatures. The PNGS at N156 is relatively conserved but not a signature and Envs that lack it are generally sensitive to V2 bNAbs. In the bottom panel, glycans N137 and N301 are not V2 bNAb signatures, but may mediate interactions between signature glycan at N332 and the core glycan at N156.

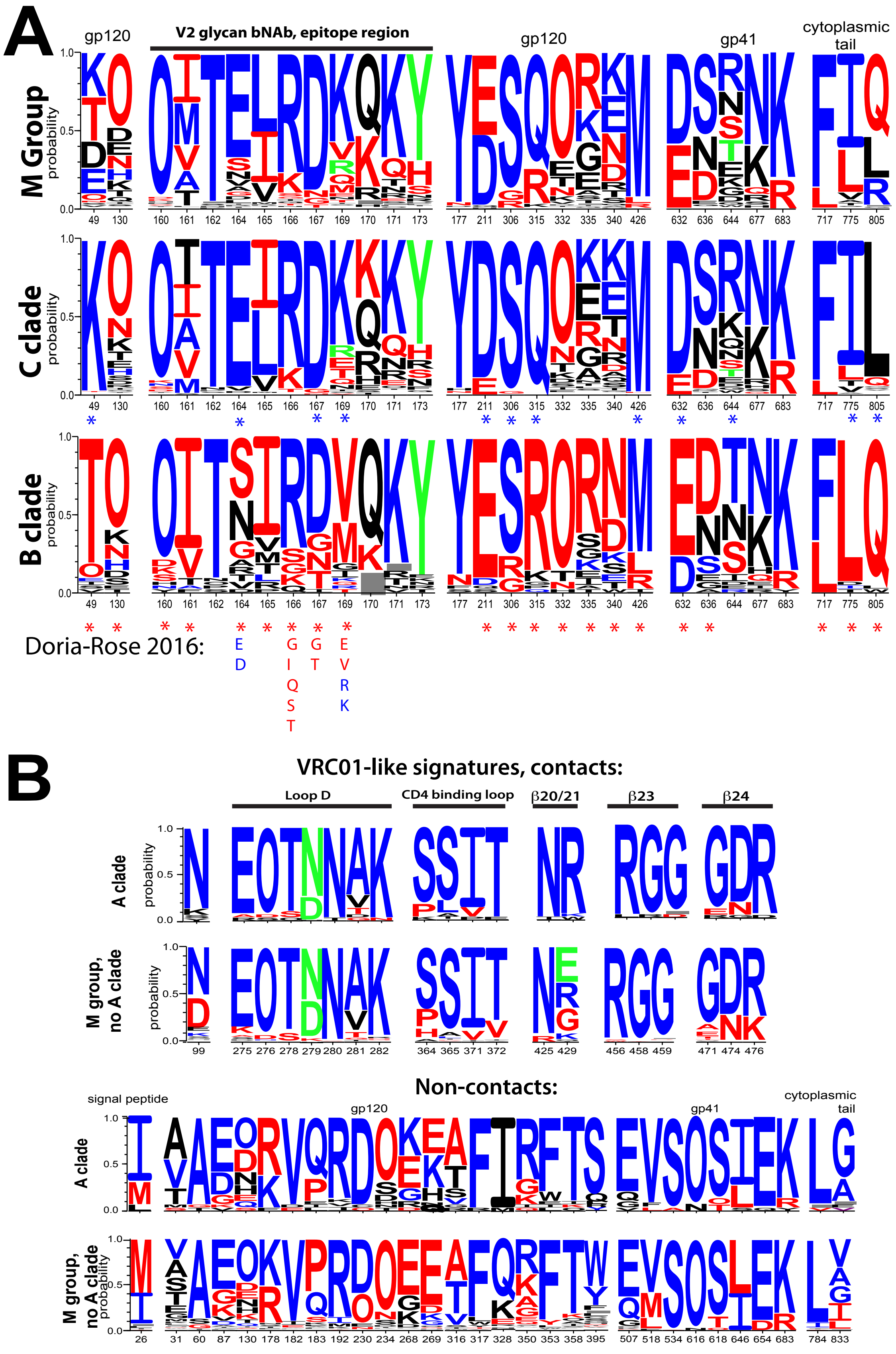
A
gp120
V2 glycan bNAb, epitope region
gp120
gp41
cytoplasmic tail
M Group
C clade
B clade
probability
1.0
0.5
0.0
49 130 160 161 162 164 165 166 167 169 170 171 173 177 211 306 315 332 335 340 426 632 636 644 677 683 717 775 805
Doria-Rose 2016:
E D
G I Q S T
G T
E V R K
B
VRC01-like signatures, contacts:
Loop D
CD4 binding loop
β20/21
β23
β24
A clade
M group, no A clade
99
275 276 278 279 280 281 282
364 365 371 372
425 429
456 458 459
471 474 476
Non-contacts:
signal peptide
gp120
gp41
cytoplasmic tail
26 31 60 87 130 178 182 183 192 230 234 268 269 316 317 328 350 353 358 395 507 518 534 616 618 646 654 683 784 833

Fig. S4. Signatures associated with clade sensitivity, supporting Figs. 2 and 3. (**A) Specific amino acids that may contribute to the clade preferences of V2 bNAbs.** V2 bNAbs signature frequencies displayed in LOGO plots based on the sequences included in dataset 4, for M-group (top), C clade (middle) and B clade (bottom). The height of the letter corresponds to it frequency. Blue indicates AAs associated with sensitivity, red with resistance, and green with sensitivity to some bNAbs, but resistance to others. (Doria-Rose et al., 2015) proposed that an enrichment for unfavorable signatures, or paucity of favorable signatures, may dictate the resistance of the B clade to the CAP256.VRC26 lineage antibodies, and identified four such signatures near the V2 region. These signatures (AA positions 164, 166, 167, and 169) are noted at the bottom of the figure, and our data support these findings. However, their observations were limited to the core epitope. We found an additional 17 signature sites (marked with red asterisks) where shifts in amino acid frequencies in the B clade (either resistance associated AAs more frequent or reduced frequencies of favorable AAs) may impact overall B clade resistance. CAP256.VRC26 lineage antibodies also have increased potency against C clade viruses and blue asterisks mark signatures that may impact this enhanced potency. Analysis in Table S5 indicates that CAP256.VRC26 antibodies all have striking preferences for K49, E164, and Q315 relative to other V2 bNAbs, so these sites are particularly good candidates for impacting the enhanced potency of the of CAP256.VRC26 bNAbs with C clade viruses. **(B) Signatures that may be associated with increased A clade sensitivity to CD4bs bNAbs.** The signatures associated with CD4bs resistance in contact residues are very rare in A clade relative to the rest of the M group (particularly in AA positions 99, 364, 372, 429, 471 and 475), and this same pattern is also evident in some on the non-contact residues (positions 26, 87, 183, 234, 268, 269, 350, 518, and 646). CD4bs antibodies with enhanced recognition of A clade viruses include: CH31, p=0.0000013; VRC01, p=0.000018; 8AN131, p=0.0004; CH235.12, p=0.00053; PG04, p=0.0011; 12A12, p=0.0028; 3BNC117, p=0.0017; N6, p = 0.04; NIH45.46, p=0.045 (p-values, Wilcoxon rank sum). VRC07, VRC06b, CH103, and VRC03 were not significantly more potent against A clade.

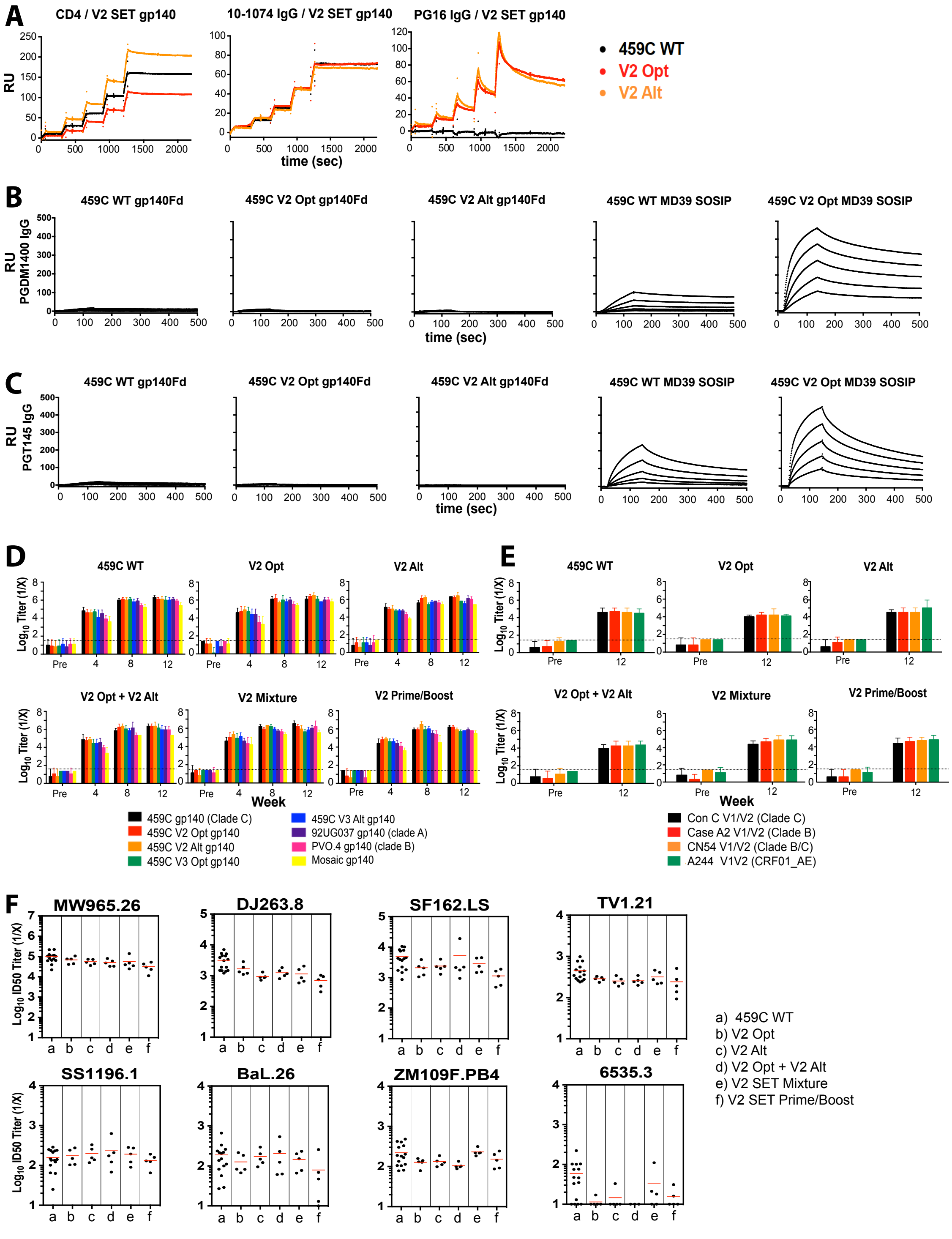
A
CD4 / V2 SET gp140
10-1074 IgG / V2 SET gp140
PG16 IgG / V2 SET gp140
RU
time (sec)
459C WT
V2 Opt
V2 Alt
B
459C WT gp140Fd
459C V2 Opt gp140Fd
459C V2 Alt gp140Fd
459C WT MD39 SOSIP
459C V2 Opt MD39 SOSIP
RU
PGDM1400 IgG
time (sec)
C
459C WT gp140Fd
459C V2 Opt gp140Fd
459C V2 Alt gp140Fd
459C WT MD39 SOSIP
459C V2 Opt MD39 SOSIP
RU
PGT145 IgG
time (sec)
D
459C WT
V2 Opt
V2 Alt
V2 Opt + V2 Alt
V2 Mixture
V2 Prime/Boost
Log10 Titer (1/X)
Pre
Week
459C gp140 (Clade C)
459C V2 Opt gp140
459C V2 Alt gp140
459C V3 Opt gp140
459C V3 Alt gp140
92UG037 gp140 (clade A)
PVO.4 gp140 (clade B)
Mosaic gp140
E
459C WT
V2 Opt
V2 Alt
V2 Opt + V2 Alt
V2 Mixture
V2 Prime/Boost
Log10 Titer (1/X)
Pre
Week
Con C V1/V2 (Clade C)
Case A2 V1/V2 (Clade B)
CN54 V1/V2 (Clade B/C)
A244 V1V2 (CRF01_AE)
F
MW965.26
DJ263.8
SF162.LS
TV1.21
SS1196.1
BaL.26
ZM109F.PB4
6535.3
Log10 ID50 Titer (1/X)
a b c d e f
a) 459C WT
b) V2 Opt
c) V2 Alt
d) V2 Opt + V2 Alt
e) V2 SET Mixture
f) V2 SET Prime/Boost

Fig. S5. Antigenicity of and Immunogenicity of V2-SET immunogens in support of Figure 5C, 6 and 7. (A-C) present surface plasmon resonance results. (A) Presentation of the CD4 binding site by soluble CD4, V3/glycan binding site by 10-1074 binding, V2/glycan binding site with PG16 binding to V2-SET gp140 foldon Envs. Sensorgram colors correspond to Env in the key. B-C. Presentation of the (B) PGDM1400 and (C) PGT145 epitopes. Gp140 foldon (gp140) or gp140 SOSIP (SOSIP) Envs were tested as denoted in the graph title. Sensorgrams are presented in black. RU, response units. **(D) Sera from V2-SET vaccinated guinea pigs tested in endpoint ELISAs against a panel of gp140 antigens.** Titles represent the vaccine given. Colors show coating Env as listed. V3-SET proteins are being further studied for 2nd generation vaccine design. (**E) Sera tested in endpoint ELISAs against V1/V2 gp70 scaffolds**. Colors correspond to scaffold origin. The horizontal dotted line indicates background and error bars indicate standard deviation for all endpoint ELISAs. **(F) Guinea pig sera obtained at week 12, tested against a multi-clade panel of tier 1A and 1B, A, B, and C clade neutralization-sensitive isolates in the TZM.bl neutralization assay.** Neutralization data for every data point are MuLV negative control background subtracted. Values less than 10 are set to 10. Horizontal red lines indicate mean titers. Vaccination regimens listed along the x-axis. The title refers to the tested pseudovirus, its tier and clade.

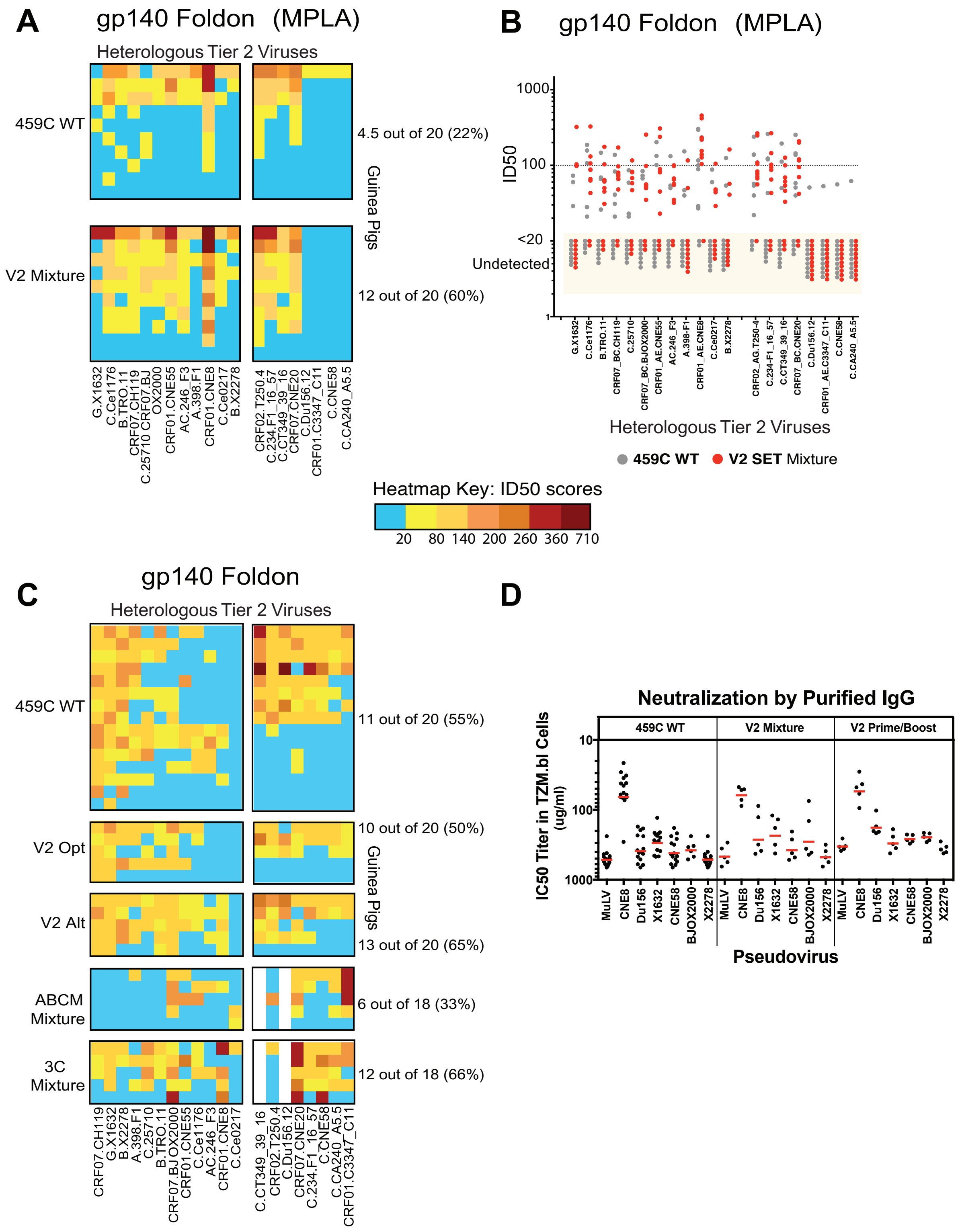
A
gp140 Foldon (MPLA)
Heterologous Tier 2 Viruses
459C WT
4.5 out of 20 (22%)
V2 Mixture
12 out of 20 (60%)
Guinea Pigs
G.X1632
C.Ce1176
B.TRO.11
CRF07.CH119
C.25710 CRF07.BJ
OX2000
CRF01.CNE55
AC.246_F3
A.398.F1
CRF01.CNE8
C.Ce0217
B.X2278
CRF02.T250.4
C.234.F1_16_57
C.CT349_39_16
CRF07.CNE20
C.Du156.12
CRF01.C3347_C11
C.CNE58
C.CA240_A5.5
B
gp140 Foldon (MPLA)
ID50
1000
100
<20
Undetected
1
Heterologous Tier 2 Viruses
459C WT
V2 SET Mixture
Heatmap Key: ID50 scores
20 80 140 200 260 360 710
C
gp140 Foldon
Heterologous Tier 2 Viruses
459C WT
11 out of 20 (55%)
V2 Opt
10 out of 20 (50%)
V2 Alt
13 out of 20 (65%)
ABCM Mixture
6 out of 18 (33%)
3C Mixture
12 out of 18 (66%)
Guinea Pigs
CRF07.CH119
G.X1632
B.X2278
A.398.F1
C.25710
B.TRO.11
CRF07.BJOX2000
CRF01.CNE55
C.Ce1176
AC.246_F3
CRF01.CNE8
C.Ce0217
C.CT349_39_16
CRF02.T250.4
C.Du156.12
CRF07.CNE20
C.234.F1_16_57
C.CNE58
C.CA240_A5.5
CRF01.C3347_C11
D
Neutralization by Purified IgG
IC50 Titer in TZM.bl Cells (ug/ml)
459C WT
V2 Mixture
V2 Prime/Boost
10
100
1000
MuLV
CNE8
Du156
X1632
CNE58
BJOX2000
X2278
Pseudovirus

Fig. S6. Robustness of the enhanced V2-SET vaccine neutralization response, in support of Fig. 6. (A) Heatmap comparisons of Tier 2 bNAbs elicited by 459C WT and V2-SET gp140 foldon vaccines, with an MPLA adjuvant. Guinea pigs were vaccinated intramuscularly at weeks 0, 8, and 24, with 100 µg of 459C WT, or with a total of 100 µg V2-SET proteins divided equally among the 3 Envs, formulated in MPLA. **(B) Comparisons of potency of the responses shown in part (A)**. The dotted line at 100 is added for visual emphasis. Dots in cream colored box are responses below the limit of detection for the assay ($ID_{50} < 20$). Colors represent vaccination regimens. (**C) Heatmap comparisons of tier 2 NAb responses to single components of the trivalent V2-SET vaccine, and two other polyvalent mixtures.** The polyvalent control vaccines included the following previously studied multivalent immunogens: The 3C mixture included 459C WT plus two additional WT clade C gp140s (Bricault et al., 2015), and the tetravalent A, B, and C clade with a Mosaic Env gp140 (ABCM Mixture) (Bricault et al., 2018). The 3C and ABCM Mixtures were analyzed against a panel of 9 C clade and 9 non-C clade pseudoviruses (18 of the original 20 were tested; Du156.12 and CT349_39_16 were not available for testing). Unlike the trivalent V2-SET vaccine, neither the 3C nor the ABCM cocktails afforded significantly enhanced tier 2 breadth as compared with 459C WT; this result did not change when the outliers (the animals with the lowest breadth of response) were removed. **(D) Purified polyclonal IgG from vaccinated guinea pigs evaluated against select tier 2 pseudoviruses and MuLV (the negative control)**. Pseudoviruses are shown alsong the x-axis. The vaccination regimen is depicted in the title. Horizontal red lines indicate mean titers. For the heatmaps in A and C, test columns represent the pseudoviruses noted below the maps, and rows correspond to a single animal grouped by vaccine regimen. Potency of ID_{50} responses are indicated in in the color key, with red to yellow most to least potent. Negative responses are shown in blue. Breadth of response for each vaccination regimen is shown to the right of the heat map as a median number of detected pseudoviruses per animal, out of the number of pseudoviruses tested, with the respective percentage in parentheses.

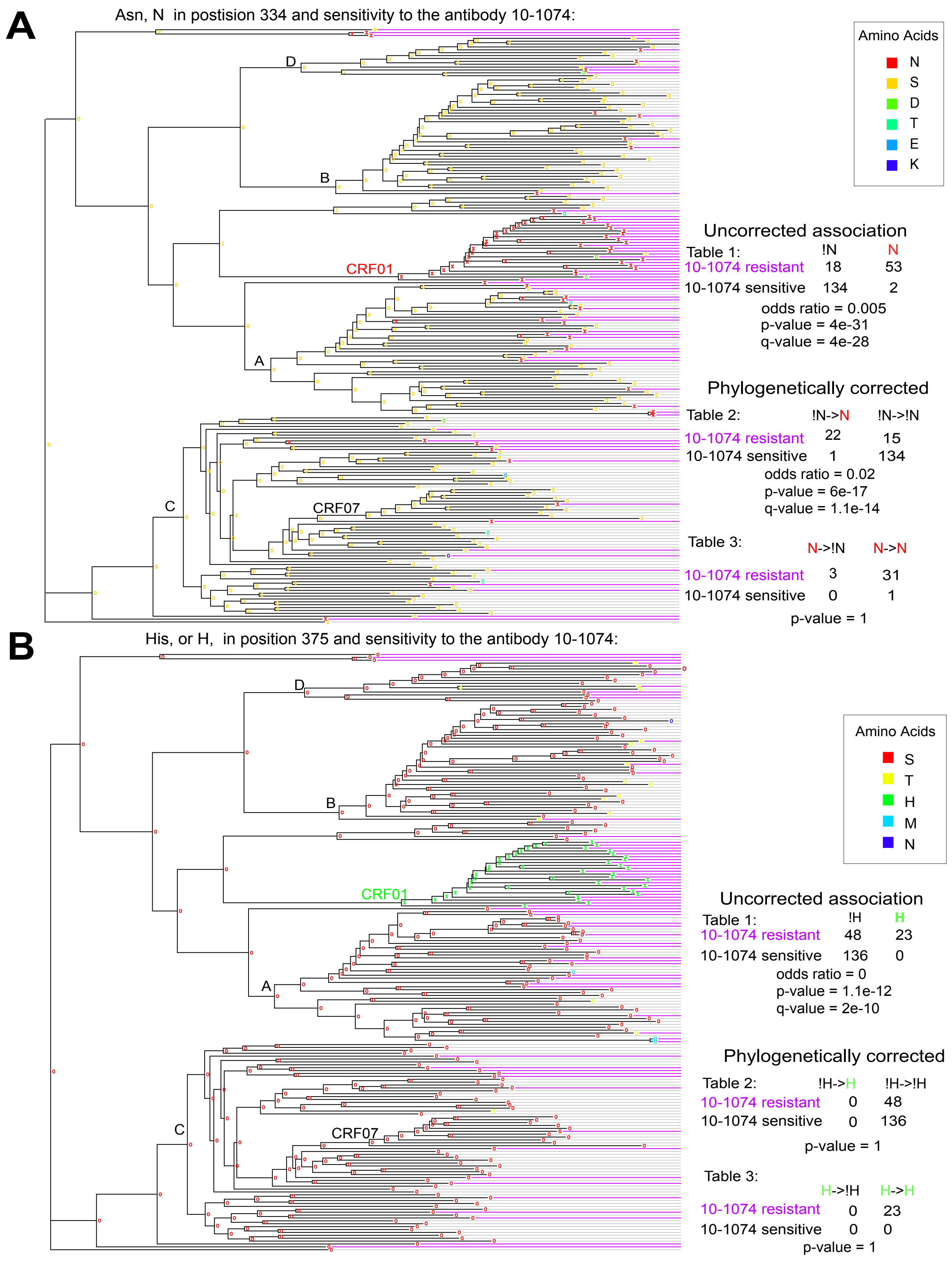
A
Asn, N in postision 334 and sensitivity to the antibody 10-1074:
D
B
CRF01
A
C
CRF07
Amino Acids
N
S
D
T
E
K
Uncorrected association
Table 1: !N N
10-1074 resistant 18 53
10-1074 sensitive 134 2
odds ratio = 0.005
p-value = 4e-31
q-value = 4e-28
Phylogenetically corrected
Table 2: !N->N !N->!N
10-1074 resistant 22 15
10-1074 sensitive 1 134
odds ratio = 0.02
p-value = 6e-17
q-value = 1.1e-14
Table 3: N->!N N->N
10-1074 resistant 3 31
10-1074 sensitive 0 1
p-value = 1
B
His, or H, in position 375 and sensitivity to the antibody 10-1074:
D
B
CRF01
A
C
CRF07
Amino Acids
S
T
H
M
N
Uncorrected association
Table 1: !H H
10-1074 resistant 48 23
10-1074 sensitive 136 0
odds ratio = 0
p-value = 1.1e-12
q-value = 2e-10
Phylogenetically corrected
Table 2: !H->H !H->!H
10-1074 resistant 0 48
10-1074 sensitive 0 136
p-value = 1
Table 3: H->!H H->H
10-1074 resistant 0 23
10-1074 sensitive 0 0
p-value = 1

Fig S7. Maximum likelihood trees with ancestral states and signature statistics illustrating the importance of a phylogenetic correction, supporting in Figure 3. (A) A phylogenetically supported relationship between N334 and 10-1074 resistance. Magenta bars mark resistant pseudotyped viral sequences (leaves), light grey mark sensitive. At the leaves, a red X indicates N334, the amino acid state that is being examined, and other amino acids are represented by other colors. The most likely amino acids in position 334 in the leaves and ancestral nodes are indicated by the color (key on the figure), based on the observed state for the leaves. Maximum likelihood provides an estimate of the most probable state of amino acid 334 given the phylogeny and the evolutionary model for the ancestral states. The number indicates the probability of the ancestral state to have been an Asn, N334 the test amino acid. 0-9 indicates a probability of 0-0.9, and X a probability approaching 1. N334 is almost always present in CRF01 (red X's), but it also recurs many times in scattered places throughout the tree. The simple Fisher's test in Table 1 shows it was present 55 times and only twice when N334 was present was the virus sensitive; this is highly significant. This correlation was also supported in the phylogenetic correction, where we asked if the ancestral node preceding the leaf was most likely not to have not been Asn (N) based on the phylogenetic reconstruction of the ancestral state, and for the end point taxa to have gained an Asn. This happened 23 times in the tree, summarized in Table 2, and 22/23 times that the pseudovirus acquired the N334 relative to its ancestral state, that virus was resistant to 10-1074. This association was also highly significant. Table 3 shows the association between 10-1074 resistance and the phylogenetic loss of N334, which was not significant. **(B) An illustration of the association between H375 and 10-1074 resistance.** In this case, H375 (indicated by green X's at the leaves) is only found in CRF01 and nowhere else in the entire tree. In other clades, the S375 is most prevalent (marked in red) but other amino acids are also found (indicated by other colors). Similar to (A), the ancestral nodes are color-coded by the maximum likelihood ancestral amino acid, with the number indicating the probability of it to be H375. In a simple Fisher's test, H375 is highly associated with 10-1074 resistance, as shown in Table 1. But there is only one branch in the tree with the mutational event resulting in a shift from S375 to H375, it is found in the long branch leading into the CRF01 clade. This long branch leading into CRF01 is indicative of the many other changes that distinguish CRF01 from other clades. So, we cannot be sure if H375 is driving the resistance of the CRF01 lineage, or if it is in linkage with one of the other positions enriched in CRF01 that drive the phenotypic effect, with H375 on its own not having a phenotypic impact on 10-1074 resistance. Thus, there is no phylogenetic support for the association with H375 and resistance, and so it is absent from Table S4. This does not prove or disprove that H375 impacts 10-1074 resistance; it might be a factor in the general resistance in the CRF01 clade but we have no other supporting evidence. In contrast, the recurrence of the N334 happens many times in the tree, and it has a strong association with resistance wherever it occurs, as shown in (A), making it a compelling candidate for being a key mutation underlying the CRF01 resistance to 10-1074, and also suggesting it is important in other clades.

Supplemental Tables

Ab class	Ab	Datasets	Donor	Donor Clade	Clonal Lineage	References
V3	10-1074	1 3 4	Donor 17	A		(Mouquet et al., 2012)
V3	10-1074V	3	Donor 17	A	10-1074	
V3	10-996	1	Donor 17	A		(Mouquet et al., 2012)
glycan	2G12	1				(Trkola et al., 1996)
V3	DH270.6	4	CH0848	C	DH270	(Bonsignori, 2017)
V3	DH270.5	4	CH0848	C	DH270	(Bonsignori, 2017)
V3	DH270.1	4	CH0848	C	DH270	(Bonsignori, 2017)
V3	PGT121	1 2 3 4	Donor 17	A	PGT121	(Garces et al., 2015; Julien et al., 2013; Mouquet et al., 2012; Walker et al., 2011)
V3	PGT123	1	Donor 17	A	PGT121	(Julien et al., 2013; Walker et al., 2011)
V3	PGT125	1	Donor 36	CRF02_AG	PGT128	(Walker et al., 2011)
V3	PGT126	1	Donor 36	CRF02_AG	PGT128	(Walker et al., 2011)
V3	PGT127	1	Donor 36	CRF02_AG	PGT128	(Pejchal et al., 2011; Walker et al., 2011)
V3	PGT128	1 3 4	Donor 36	CRF02_AG	PGT128	(Pejchal et al., 2011; Walker et al., 2011)
V3	PGT135	1	Donor 39	C		(Kong et al., 2013)
V2	CAP256.08	3 4	CAP256	C	VRC26	(Doria-Rose et al., 2015; Doria-Rose et al., 2014)
V2	CAP256.25	3 4	CAP256	C	VRC26	(Doria-Rose et al., 2015; Doria-Rose et al., 2014)
V2	CAP256.26	1 4	CAP256	C	VRC26	(Doria-Rose et al., 2015; Doria-Rose et al., 2014; Gorman et al., 2016)
V2	CH01	1	CH0219	A	CH01	(Bonsignori et al., 2011)
V2	PG9	1 2 3 4	Donor 24	A	PG9	(McLellan et al., 2011; Pejchal et al., 2010; Walker et al., 2009)
V2	PG16	1 2 4	Donor 24	A	PG9	(McLellan et al., 2011; Pejchal et al., 2010; Walker et al., 2009)
V2	PGDM1400	3 4	Donor 84	C		(Sok et al., 2014)

V2	PGT142	2	Donor 84	C	PGT145	(McLellan et al., 2011; Walker et al., 2011)
V2	PGT143	1	Donor 84	C	PGT145	(McLellan et al., 2011; Walker et al., 2011)
V2	PGT145	1 2 3 4	Donor 84	C	PGT145	(McLellan et al., 2011; Walker et al., 2011)
CD4bs	8ANC131	1 2	Patient 8, RU8	B		(Scheid et al., 2011; Zhou et al., 2015)
CD4bs	1B2530	1	Patient 1, RU1	B		(Scheid et al., 2011; Zhou et al., 2015)
CD4bs	CH235.12	4	CH0505	C		(Bonsignori et al., 2016)
CD4bs	12A12	1 2	Patient 12	CRF02_AG		(Scheid et al., 2011; Zhou et al., 2015)
CD4bs	3BC176	1				
CD4bs	3BNC117	1 2	Patient 3	B	3BNC117	Scheid et al., 2011; Zhou et al., 2015)
CD4bs	3BNC55	1	VC10042	B	3BNC117	(Scheid et al., 2011; Zhou et al., 2015)
CD4bs	CH31	1 2	CH0219	A	CH01	(Wu et al., 2011(Wu et al., 2011; Zhou et al., 2015)
CD4bs	NIH45-46	1 2	NIH45	B	VRC01	(Diskin et al., 2011; Scheid et al., 2011)
CD4bs	NIH45-46-WPY	1	NIH45	B	VRC01-modified	
CD4bs	NIH45-46-WY	1	NIH45	B	VRC01-modified	
CD4bs	NIH45-46-G54W	1	NIH45	B	VRC01-modified	(Diskin et al., 2011)
CD4bs	VRC01	1 2	NIH45	B	VRC01	(Wu et al., 2010; Wu et al., 2011; Zhou et al., 2010; Zhou et al., 2015)
CD4bs	VRC07	1 2	NIH45	B	VRC01	(Rudicell et al., 2014)
CD4bs	VRC07.523.LS	3 4	NIH45	B	VRC07-modified	(Rudicell et al., 2014)
CD4bs	VRC03	1 2	NIH45	B	VRC01	(Diskin et al., 2011; Wu et al., 2010; Wu et al., 2015; Wu et al., 2011)
CD4bs	N6	4	Z258	B		(Huang et al., 2016)
CD4bs	VRC06b	1 2	NIH45	B	VRC01	
CD4bs	VRC22	1				
CD4bs	PG04	1 2	Donor 74	A/D	PG04	(Wu et al., 2011; Zhou et al., 2015)
CD4bs	PG05	1	Donor 74	A/D		*

CD4bs	CH103	1 2	CH0505	C		(Gao et al., 2014; Liao et al., 2013)
CD4bs	HJ16	1	V13208	C		(Corti et al., 2010; Zhou et al., 2015)
CD4bs	VRC13	1	Donor 44	B		(Zhou et al., 2015)
CD4bs	VRC16	1	Donor C38	unk		(Zhou et al., 2015)
CD4bs	IgG1b12	1	Donor b	unk		(Burton et al., 1991; Zhou et al., 2015)
MPER	10E8	1 2	Donor N152	B		(Huang et al., 2012)
MPER	2F5	1 2				(Buchacher et al., 1994; Ofek et al., 2004)
MPER	4E10	1 2				(Buchacher et al., 1994; Cardoso et al., 2005)
MPER	Z13E1	1	FDA2	B		(Nelson et al., 2007; Pejchal et al., 2009)
MPER	DH511.2	4	CH0210	C	DH511	(Williams et al., 2017)
MPER	DH511.2_K3	4	CH0210	C	DH511	(Williams et al., 2017)

*PG05 is described in patent application WO 2012040562 A2. The patent indicates PG05 did not compete with other CD4bs antibodies, while PG04 does; however, PG05 is sold through Creative Biolabs as a CD4bs antibody. Given the uncertainty, we are including it with CD4bs set for completeness; it had poor neutralization breadth and gave no strong signatures so does not impact the overall CD4bs signature analysis.

Table S1. bNAbs included in this study, the foundation for Figs. 1-3. The columns indicate the antibody class, common name, the datasets that included the bNAb, the subject ID from whom it was isolated, the HIV-1 clade of the infecting strain, and the clonal lineage that gave rise to the antibody if reported, or a note if the antibody is a modification of a natural antibody, and selected references that describe the antibody isolation, structure, and/or contact residues. The type of CD4bs antibody as described by Zhou et al. (Zhou et al., 2015) is indicated by the color of the antibody name: VH1-46, brown; VH1-2, green; and CDR H3, red; these subdivisions of CD4bs bNAbs were used as breakdowns for integrating signature analysis across antibodies.

	A	B	C	D	G	01	02	07	p-value	tau
**%PNGS N332 dataset 4**	57	88	86	71	83	4	73	86	-	-
**%PNGS N322 database**	67	86	77	80	78	3	83	84	0.017	0.61
**% Neutralization positive DH270.6**	43	78	69	30	86	0	53	86	0.012	0.67
**% Neutralization positive PGT128**	57	76	66	50	71	56	33	71	0.039	0.52
**% Neutralization positive PGT121**	57	83	77	50	86	4	67	86	0.011	0.67
**% Neutralization positive 10-1074**	50	90	79	70	86	0	73	86	0.000001	0.88

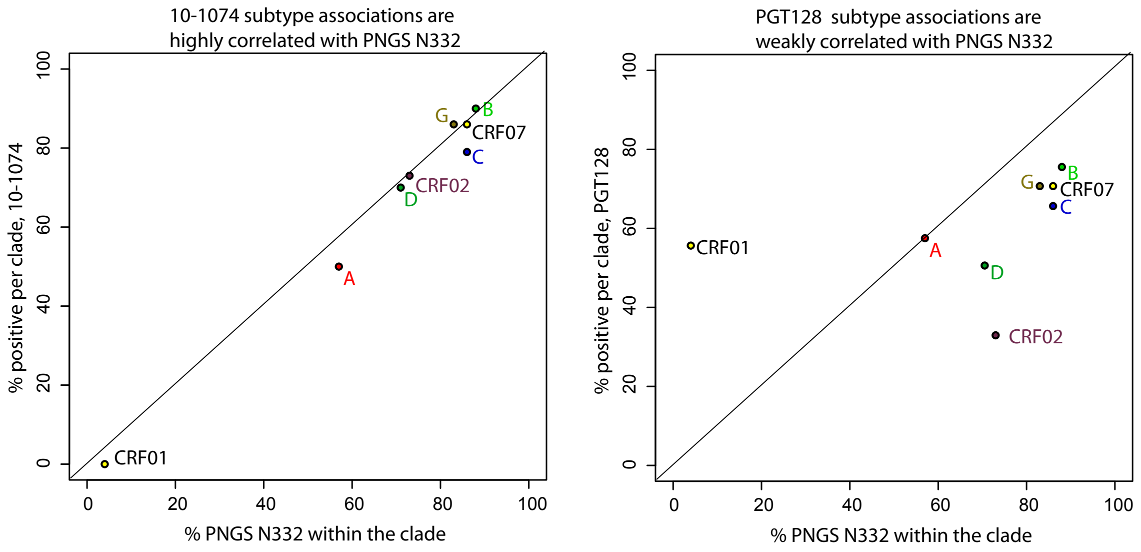

Table S2. N332 frequencies by subtype and recognition of V3 glycan antibodies, supporting Figs. 1-2. The first row shows the percentages of viruses that carry the PNGS N332 in each clade in dataset 4 (207 Envs). The second row is the percentage of the PNGS N332 found in the curated Los Alamos database M group alignment (with 5451 sequences, only one HIV sequence per person in the database, second row), showing that the frequency of the PNGS N332 in dataset 4 is reasonably representative of the larger database. The next 4 rows are the percentage of the viruses that are positive (IC_{50} of <50 ug/ml) for any one subtype for a given V3 bNAb. The lack of PNGS at N332 is very common in some clades, less common in others, and this is significantly correlated with the frequency with which a clade is recognized for each antibody studied. The loss of the PNGS at N332 is clearly not the only way to confer resistance, still it is highly predictive of the recognition within clades. The p-values are from Kendall's tau, and compare the frequencies of the PNGS N332 in dataset 4 to each of the other rows. Underneath the table are two plots to illustrate the relationship between V3 bNAb sensitivity within in a clade and the frequency of PNGS N332, showing the two antibodies with the strongest and weakest correlation. On the left is a plot of the V3 bNAb with the strongest correlation between PNGS N332 and sensitivity, 10-1074. The diagonal line indicates a perfect correlation. This illustrates 10-1074's has a strict dependence on PNGS N332 (p=0.000001, Kendall's tau). PGT128 is the least correlated of the V3 bNAbs (p = 0.039), and is plotted on the right. In the case of PGT128, there must be additional mutations aside from the loss of PNGS N332 commonly driving resistance, as most clades drop well below the diagonal. This is particularly evident in CRF02, a very common in West and Central Africa.

Table S3: All signature tables for all antibodies and datasets. These tables support Figs. 3 and 4.

A-D. Summary tables organized by site, amino acids, and antibodies, and providing an overview of all signature sites found for a given antibody class, across all 4 datasets. Separate tabs are provided for each bNAb class studied (tabs A-D). Sites within hypervariable regions are excluded. The antibodies included in each of the four primary datasets are listed at the top. Sites are included as a signature if at least one antibody in one dataset had a phylogenetically corrected signature site with a q-value of < 0.2 (Table S3 tabs E-H) or if the signature site was in a contact residue. If either of these criteria was met, the site was deemed of interest, and all simple (without a phylogenetic correction) Fisher's exact associations with a q-value < 0.2 were then included and tracked for that site. Complete details of statistical support for each signature are included in Table S3 tabs E-H (phylogenetic corrected signatures only, organized by antibody) and S3 tabs I-L (all simple associations for sites of interest, organized by site). Antibodies with significant associations after a phylogenetic correction (from Table S3 E-H) are bold. The positions are based on HXB2 numbering. If the site is known to be in a contact site for an antibody in the class, based on structural studies, the position number is colored and bold. Amino acids significantly associated with bNAb resistance are colored red, those that are associated with sensitivity are blue. Glycosylation site patterns were tracked, and a PNGS motif is noted as "NxST". Note that lack of an association in a particular dataset does not mean that the association in another is not valid for a given antibody, it may simply mean that a given dataset did not have enough power to resolve the association statistically. If a particular site was associated with bNAbs sensitivity/resistance in multiple datasets, for more than one bNAb in a class, or was located in a known contact residue, it was deemed likely to be robust as it was supported by several lines of evidence (HXB2 position numbers of such sites are highlighted in bold). These more robust associations were used as a basis for the signature profiles in Fig. 3 in the main body of the text and for structural mapping. Contradictory signatures, associated with sensitivity to some antibodies in a class, but resistance to others, are highlighted in tan. Tabs:

A. **V3 bNAb signature summary.** Contact residues are indicated in blue in the Position column

B. **V2 bNAb signature summary.** Contact residues are indicated in green in the Position column.

C. **CD4bs bNAb signature summary.** The HXB2 positions of contact residues as described in the legend to Table S4C are indicated by lavender text. Most CD4bs bNAbs are VH1-2 or VH-46 using and are noted in black. The sites that were used for the illustration in the main paper in Fig. 3 focus on VH1-2 or VH-46 usage CD4bs bNAb signatures. Antibody names in red include CDRH3 dominated antibodies (Zhou et al., 2015) CH103, HJ16, VRC13, VRC16, and IgG1b12, plus two VH1-2 antibodies, VRC03 and VRC06, that tended to track with CDRH3 bNAbs in terms of signature associations. These antibodies generally have less breadth and often have contradictory signatures relative to most VH1-2 or VH-46 using CD4bs bNAbs.

D. **D. MPER bNAb signature summary.** Contact residues are indicated in the Position in dark brown for 2F5, light brown for 4E10/10E8/DH511.

E-H. Phylogenetically corrected signatures, organized by antibody. These tables list the statistical support for all phylogenetically corrected amino acid signatures with q-values <0.2 for each antibody studied, organized by antibody class and antibody, providing details regarding signatures statistics. Separate tabs are provided for each bNAb class studied (S3 E-H). The phylogenetic correction compares to the neutralization phenotype the amino acids in sites that are unchanging to those that change between a taxon and its most recent ancestral node as estimated using a maximum likelihood tree. The column headings are as follows. The "*Table*" columns are T2 and T3, for Table 2 and 3. These tables are phylogenetically corrected signatures. A detailed example of how to read each kind of table is provided in each spreadsheet. If the signature analysis was testing for N-linked glycosylation sites rather than simple amino acids, it is indicated as a "glycan" table, *e.g.* T2glycan. The *Dataset* is either: the first (1) or second (2) completely independent M group datasets, the C clade dataset (3), or the larger M group data (4), shown in Fig. S1. The *cutoff* is the cutoff used for the input phenotype that gave the highest degree of statistical support for a particular signature. PosNeg means the data was broken down between positive, i.e. a detected IC_{50}, and negative, with IC_{50} above the threshold of detection. Data was also broken down by above or below the median titer, and upper and lower quartiles. The *HXB2 pos* is the position in the alignment based on HXB2 numbering. The *test AA* is the amino acid that was being evaluated in the position; only those with a q-value <0.2 are included. Also, we excluded a small number of cases when phylogenetic association were not also supported by a simple uncorrected association. If *blue*, its presence was associated with enhanced sensitivity, if *red*, with resistance. NxST is an abbreviation to refer to an intact N-linked glycosylation site motif. *Antibody* is the name of the bNAb. *P-value, q-value* and *Odds Ratio* are all summary statistics, that are based on the 2x2 contingency tables that are outlined as r1c1, r1c2, r2c1, r2c2, where r stands for row, c for column. See T2 and T3 examples of how to read the contingency tables for the two distinct types of corrections, change towards or away from a given

AA. P-values are based on a 2-sided Fisher's exact test, the q-values were based on all signature p-values. We also list ranked AAs, based on the most informative AAs for our machine learning implementation of *Regression* (predicting IC_{50} values from sequences, Table S4) and *Classification* (predicting positive/negative IC_{50} values from sequences, Table S5). These are listed by rank of importance, followed by the HXB2 position and the amino acid, or a dash if a deletion is important. The next columns show results from other signature analysis papers, including just the associations that were directly reported and readily retrieved from the primary publications. The association is listed alongside signature amino acid we have identified when possible. From West et al. (West et al., 2013) we report associations given as the antibody name, the amino acid association, and the position. Chuang et al. summarizes the published NEP predictions for the 10 highest rank scores (Chuang et al., 2013; Chuang et al., 2014). Hepler et al. (Hepler et al., 2014) associations are from the primary publication using IDEPI. Ferguson et al.'s results are listed as compressed sensing results (given as amino acid and position), ensemble support including mutual information (given as yes or no) and experimental support (given as yes or ND for not done).

- **E. V3 bNAb phylogenetically corrected signatures and statistical support.** V3 bNAbs contacts are highlighted in blue, based on two Env bound structures: PGT128 (301, 303, 304, 323-327, 332) (Pejchal et al., 2011) and PGT135 (295, 301, 330, 332, 339, 373, 384, 386, 389, 392, 409, 415, 417-419). The bNAb 2G12 is also included here, even though its epitope is very distinct from the other antibodies included this table.
- **F. V2 bNAb phylogenetically corrected signatures and statistical support.** V2 bNAbs contacts are highlighted in green, based on structural contacts for PG9 (McLellan et al., 2011) (contact signatures are: PNGS at N156-158, PNGS at N160-162, 165, 167-171, 173).
- **G. CD4bs bNAb phylogenetically corrected signatures and statistical support.** Representative CD4bs bNAbs contacts are highlighted in lavender and are based on an inclusive summary of structural contacts defined for CD4 and CD4bs bNAbs. These contact regions include the following HXB2 positions: V1 proximal: 97-99, 122-129, 196-198, 207, loop D: 275-283, 308, 318, CD4 binding loop: 364-374, beta20/21: 425-432, beta23: 455-459, V5 hypervariable region 460-465, beta 24: 466-477. Sites within the V5 hypervariable region are not included in the signature analysis, even though they can interact directly with the CD4bs bNAbs, due to alignment uncertainty. Contacts regions for CD4bs bNAbs and CD4 were defined based on the following data: CD4 contacts (Wu et al., 2011; Zhou et al., 2010): 124-127, 196-198, 279-283, 365-370, 374, 425-432, 455-461, 469-477; VRC01 contacts (Wu et al., 2011; Zhou et al., 2010): 97, 122, 276, 278-283, 365-368, 371, 427-430, 455-476; IgG1b12 contacts (Zhou et al., 2007): 267, 268, 280, 281, 364-373, 395, 397, 417-419, 430-432, 453-458; NIH45-46 contacts: 97-99, 102, 122-124, 127-128, 276, 427, 430-432, 455-480; 3BNC117 contacts (Scheid et al., 2016): 124, 198, 207, 275-276, 278-282, 308, 318, 365-368, 371, 428-430, 455-462, 469, 473.
- **H. MPER bNAb phylogenetically corrected signatures and statistical support.** 2F5 and other MPER antibodies bind to distinct regions. The 2F5 epitope is focused on the sites 662-668 (the HXB2 sequence ELDKWAS) and is highlighted in dark brown. 4E10 is focused on the sites 671-676 (NWFDIT) (Cardoso et al., 2005) and the broader more potent 10E8 extends further out, 671-683, NWFDISNWLWYIK with contacts including positions 671-673 and 676 (Huang et al., 2012). The DH511 lineage binds to an epitope similar to 10E8 (Williams et al., 2017). The 10E8/4E10/DH511 epitopes are highlighted in light brown.

I-M. Amino acid associations with bNAb sensitivity in sites of interest, organized by site. Separate tabs are provided for each bNAb class studied (I-L). We include sites here after a hypothesis has been raised that a site is of interest: if a site is statistically significant after a phylogenetic correction, *i.e.* included in Table S4 for any antibody in a class, or if it is directly in a bNAb contact residue, it is considered of interest for the full bNAb class. Next, Fisher's test for associations of all amino acids at that site with a $q < 0.2$ for all antibodies in that class are listed. "Table 1" (T1) is a contingency table for a simple Fisher's exact test based on the amino acid under consideration in all of the sequences in the set and their IC_{50} breakdowns, with no phylogenetic correction applied. This table uses the same columns headings defined in Table S3 E-H, but the table rows are organized by HXB2 position instead of by antibody. An example of how to read the contingency table is provided in each data spreadsheet. We then list importance-ranked signatures based on our machine learning implementation for regression (levels of sensitivity, Table S4) and classification (positive/negative Table S5), followed by columns that show previously published signatures for antibodies in our study. The association is listed alongside signature sites we have identified, if the earlier finding is also supported by our analyses, or in a separate row if we did not find support for the reported association in our analyses.

I. Simple signatures associated with V3 bNAb sensitivity in sites deemed of interest. Contact residues are highlighted in blue.

J. Simple signatures associated with V2 bNAb sensitivity in sites deemed of interest. Contact residues are highlighted in green.

K. Simple signatures associated with CD4bs bNAb sensitivity in sites deemed of interest. Contact residues are highlighted in lavender.

L. Additional signatures associated with MPER bNAb sensitivity in sites deemed of interest. 2F5 Contact residues are highlighted in dark brown, other MPER antibody contacts in light brown.

M. Results of applying a Wilcoxon test to CD4bs antibodies from dataset 4. Our signature bioinformatics tool provides an option to use a Wilcoxon rank sum test to compare the IC_{50} score distributions, in the presence or absence of a give amino acid at a given position, and we tested its performance for dataset 4. For most bNAb classes, this yielded fewer signatures and less significant results than the Fisher's exact test for the same data, but CD4bs bNAbs had exceptions listed here. This table includes only cases for which the Wilcoxon yielded comparable or lower p-values than Fishers, and so adds signatures to part C. The number of pseudoviruses with (AA) and without (AA) the signature amino acid, and the median value of the IC_{50} data for that set of pseudoviruses, are noted for each antibody.

N-R. Hypervariable region characteristic signatures. Excel spread sheet, supporting Fig. 2B. The statistics of associations between of V1, V2, V4, and V5 hypervariable region characteristics and IC_{50} scores for each antibody organized by antibody class. Separate tabs are provided for each bNAb class studied (N-Q); and tab R provides a key showing the boundaries of hypervariable regions relative to HXB2. Our analyses considered characteristics of the full-length variable loops, the more narrowly defined hypervariable segment (in bold lettering) that cannot be reliably aligned, and the sum of behaviors across both V1 and V2; we included 10 regions in all, in our search for correlates with bNAb potency. The characteristics of combined V1 + V2 regions were often a stronger correlate of bNAb sensitivity than of either V1 or V2 considered in isolation. Only characteristics that had at least one association based on Kendall's tau with a q-value < 0.2 are captured in this table; once that level of significance was found, the characteristic is considered of potential interest, and all associations between a characteristic and antibodies of the same class are shown. Dataset 3 (C clade) and dataset 4 (M group) are included here, as they are the largest datasets and best powered. If only the hypervariable region of a loop was used for the analysis, it is indicated by an "h", for example V1 means the entire V1 loops was used, V1h means only the hypervariable region. If two highly related characteristics were identified as statistically of interest, like V1 and V1h, only the most significant relationship of the two was retained. The characteristics are: *Charge*, the net charge of the amino acids spanning the region considered (summing over each region such that an Arg, Lys and His contribute +1, Glu and Asp -1); *Length,* the number of amino acids in the region based on the HXB2 boundaries; and *Glycos,* the number of PNGSs within the boundaries of the region under consideration. Kendall's tau was used to calculate p-values.

N. V loop and hypervariable region characteristics associated with V3 bNAb sensitivity. Excluding negative IC_{50} responses enhanced correlations, so the impact of loop length on potency among just positive responders was more dramatic. This is likely because viruses are completely resistant when the PNGS at N332 is lost, regardless of loop characteristics. Thus, even viruses with favorable loop characteristics will be negative if the PNGS at N332 is absent, complicating resolution of other characteristics of importance.

O. V loop and hypervariable region characteristics associated with V2 bNAb sensitivity.

P. V loop and hypervariable region characteristics associated with CD4bs bNAb sensitivity.

Q. V loop and hypervariable region characteristics associated with MPER bNAb sensitivity Increasing numbers of PNGS's in the V1 loop correlated with enhanced sensitivity to 10E8, and with other MPER bNAbs to a lesser extent. This was the only case where increasing the size of the variable region was associated with increased bNAb sensitivity.

R. Hypervariable region boundaries are relative to the HXB2 reference strain V loop sequences. Hypervariable regions are highlighted in bold and red and are subregions of the full variable region loops.

Regression Predictions			MAE		R^2		p-value	
Class	Antibody	Features	Xval	Hold	Xval	Hold	Xval	Hold
V2	CAP256-VRC26.25	AllSig	1.35	**1.55**	**0.47**	**0.36**	7.50E-27	9.80E-20
V2	CAP256-VRC26.25	Contact	1.46	1.64	0.37	0.27	4.90E-20	1.80E-16
V2	CAP256-VRC26.25	mRMR 3	**1.32**	1.71	0.46	0.27	2.40E-20	5.30E-16
V2	PG9	AllSig	**0.77**	**0.85**	**0.53**	**0.51**	3.50E-27	5.60E-24
V2	PG9	Contact	0.87	1.01	0.4	0.34	7.80E-19	4.00E-16
V2	PG9	mRMR 86	0.87	1.02	0.39	0.31	1.20E-17	4.10E-12
V2	PGDM1400	AllSig	**0.88**	**1.06**	**0.48**	**0.49**	8.80E-25	1.60E-22
V2	PGDM1400	Contact	1.05	1.19	0.34	0.38	7.40E-17	1.40E-15
V2	PGDM1400	mRMR 5	1.05	1.12	0.32	0.36	2.50E-08	9.30E-13
V2	PGT145	AllSig	**1.28**	1.18	**0.27**	0.25	4.90E-14	2.50E-10
V2	PGT145	Contact	1.39	1.33	0.17	0.18	5.00E-09	3.20E-08
V2	PGT145	mRMR 6	1.34	**1.07**	0.18	**0.28**	9.40E-07	1.20E-08
V3	10-1074	AllSig	**0.79**	**0.73**	**0.71**	**0.69**	1.80E-38	1.00E-28
V3	10-1074	Contact	0.8	0.84	**0.71**	0.63	1.50E-33	8.50E-23
V3	10-1074	mRMR 99	0.89	0.82	0.64	0.64	2.80E-29	1.00E-25
V3	PGT121	AllSig	1.14	1.09	0.48	**0.43**	1.60E-28	6.30E-20
V3	PGT121	Contact	1.13	1.37	0.47	0.21	6.10E-25	1.50E-09
V3	PGT121	mRMR 77	**1.12**	**1.08**	**0.50**	0.41	3.00E-28	3.70E-19
V3	PGT128	AllSig	1.11	1.17	**0.37**	**0.39**	6.20E-19	3.10E-17
V3	PGT128	Contact	1.09	1.22	0.36	0.28	1.70E-17	1.50E-12
V3	PGT128	mRMR 15	**1.08**	**1.05**	0.32	0.33	3.30E-16	2.30E-15
CD4bs	3BNC117	AllSig	**0.73**	**0.9**	**0.48**	**0.25**	1.70E-19	2.10E-09
CD4bs	3BNC117	Contact	0.79	1.03	0.35	0.1	1.10E-14	1.80E-04
CD4bs	3BNC117	mRMR 96	0.84	0.94	0.29	0.19	4.20E-12	3.20E-07
CD4bs	VRC01	AllSig	**0.59**	0.74	**0.33**	0.22	1.80E-12	7.00E-10
CD4bs	VRC01	Contact	0.67	0.78	0.2	0.16	8.70E-05	3.80E-05
CD4bs	VRC01	mRMR 94	0.66	**0.72**	0.19	**0.26**	7.50E-06	8.50E-11
CD4bs	VRC07	AllSig	**0.55**	**0.7**	**0.43**	**0.25**	6.40E-14	2.80E-10
CD4bs	VRC07	Contact	0.67	0.76	0.22	0.21	4.30E-03	7.40E-04
CD4bs	VRC07	mRMR 10	0.62	0.76	0.22	0.16	8.40E-01	4.60E-07
CD4bs	VRC07-523-LS	AllSig	**0.63**	**0.66**	**0.35**	**0.1**	1.10E-11	1.50E-06
CD4bs	VRC07-523-LS	Contact	0.76	0.72	0.13	0.1	3.60E-01	4.80E-02
CD4bs	VRC07-523-LS	mRMR 8	0.66	0.69	**0.35**	0.06	4.60E-04	5.60E-04
MPER	10E8	AllSig	0.62	**0.48**	0.15	0.12	6.70E-08	1.00E-03
MPER	10E8	Contact	**0.6**	0.5	**0.18**	**0.15**	9.40E-04	1.20E-04
MPER	10E8	mRMR 24	0.65	0.54	0.07	0.03	2.80E-04	9.10E-01
MPER	4E10	AllSig	**0.53**	**0.59**	**0.13**	0.08	6.90E-05	1.30E-04
MPER	4E10	Contact	**0.53**	0.65	0.08	**0.11**	5.00E-02	1.90E-05
MPER	4E10	mRMR 41	0.55	0.74	0.03	0	4.90E-03	3.20E-01

	R^2 Xval p-value	R^2 Holdout p-value	R^2 Xval: Mean, Median (interquartile range)	R^2 Holdout: Mean, Median (interquartile range)
AllSig			0.39, 0.43 (0.33-0.48)	0.32, 0.25, (0.22-0.43)
Contact	0.003	0.003	0.31, 0.34 (0.18-0.37)	0.24, 0.27 (0.15-0.28)
mRMR	0.002	0.003	0.30, 0.32 (0.19-0.39)	0.25, 0.27 (0.16-0.33

	MAE Xval p-value	MAE Holdout p-value	MAE Xval: Mean, Median (interquartile range)	MAE Holdout: Mean, Median (interquartile range)
AllSig			0.84, 0.77 (0.62-1.11)	0.90, 0.85 (0.70-1.09)
Contact	0.007	0.0008	0.91, 0.80 (0.67-1.09)	1.00, 1.01 (0.76-1.22)
mRMR	0.009	0.05	0.90, 0.87 (0.66-1.08)	0.94, 0.94 (0.74-1.07)

Table S4. Machine learning regression prediction statistical details, supporting Figure 4 in the main text. We compared three strategies for machine learning input data filtering. The full set of genetic signatures for each class of antibody was used as input for the “AllSig” predictions. “Contact” predictions used only amino acid signatures inside the contact region. We also used mRMR (Peng et al., 2005) to pick the top informative 100 features (amino acids in particular positions associated with the neutralization sensitivity), with the most informative sites are listed in the footnotes. In this table, the number that follows the feature selection method mRMR is the number of features for which cross-validation produced the highest prediction score, testing up to 100 features. To compare these feature selection methods we used the ExtraTreesRegressor Random Forest method implemented in the scikit-learn package or predictions (Geurts et al., 2006). “Xval” columns refer to the global M group data (dataset 4) being evaluated for Random Forest predictions using leave-one-out cross validation. “Hold” refers to the C clade independent holdout set predictions evaluated using a model trained on dataset 4. R^2, the coefficient of determination, was calculated by comparing RF predictions to experimentally observed values. Mean Absolute Error (MAE) was also calculated to measure prediction accuracy; the lower the number, the better the prediction. The p-values on the right are based on a non-parametric Kendall’s correlation statistic. The best prediction method for each antibody is bold in the MAE column, and for both the cross-validation and the holdout set, using the full set of signatures gave the best predictions overall (see text for statistical summary). The mean, median, and interquartile range of the R^2 statistic across all antibodies is given for the different classes of input, for the cross validation and holdout, and the p-values for a paired Wilcoxon comparing either all signature sites to contacts-only, or all signature sites to mRMR. The p-values and summary statistics indicate using all signatures as a pre-filter generally gave higher accuracy when considered across all antibodies than using only contact signatures, or mRMR.

***REGRESSION footnotes:**

CAP256-VRC26.25	3/3	V169**K** R315**Q** R166**R**
PG9	10/86	O160**O** K171**K** t138d V169**K** q170k -132k**-** 189h**-** F717**F** r633r o750h
PGDM1400	5/5	O160**O** v169e q170- l619l -787d**K**
PGT145	6/6	O160**O** G167**D** v169e o332o m426r l134t
10-1074	10/99	O332**O** N300**N** n325k -363ap -363a**S** e824g V833**V** l755i K362**O** t341a
PGT121	10/77	O332**O** N300**N** I148- 363a**S** V255**V** N325**D** S334**S** S143- O301**O** v255i
PGT128	10/15	O332**O** O295**O** i320- T297**T** O301**O** I165**I** k151q H330**H** S334**S** R304**R**
3BNC117	10/96	G459**G** G471**G** q442o r853a M026**M** n462d R456**R** q621d -363a**S** k033d
VRC01	10/94	R456**R** 012a**-** a060g -397c**-** g471i q805r I371**I** d279**D** T450**T** -363ah
VRC07	10/10	R456**R** g471i G459**G** **-**132qd N355- O276**O** -363b**-** f353y r456s N280**N**
VRC07-523-LS	8/8	R456**R** g471i G459**G** N355- O276**O** r456s N280**N** Q258**Q**
10E8	10/24	t676s E268**E** r308h n671t l134s e351k e403l p369v -189h**-** i182e
4E10	10/41	k231e n325n o406a L025**L** s411n k155t e409d e464e t188q -189en
2F5	3/3	A667A K665**K** E662**E**

***Footnotes:** For each antibody, the number following mRMR in the table indicates the number of features corresponding to the N best features according to the random forest feature importance score, out of the M features that were chosen by mRMR, where M is chosen by yielding the best cross-validation score. N/M is shown for each antibody in the footnotes, followed by up to 10 of the N best features for each bNAb ordered by rank of importance. The feature naming convention is: Letter-Number-Letter, where Number is the HXB2 site number, the first Letter is the HXB2 residue at that site, and the last Letter is the signature residue. O stands for a PNGS. For features for which the HXB2 residue is "-" (a dash inserted to maintain the alignment), we use the format of Number-Letter-Letter, where now Number-Letter is the site number (e.g., 363a) which is the first "-" after HXB2 site 363. The last letter is the residue at that site in the sequence that is the one important for predictions. If the letter is uppercase and bold, it is associated with increased sensitivity, lower case plain text, resistance.

Class	Antibody	Fxn Positive M group	Fxn Positive C clade	Features	Accuracy Xval	Accuracy Hout	Neg-accuracy Xval	Neg-accuracy Hout	Pos-accuray Xval	Pos-accuray Hout	MCC Xval	MCC Hout	p-value Xval	p-value Hout
V2	PG9	0.8	0.71	AllSig	0.87	**0.82**	0.49	0.39	0.97	0.99	0.55	**0.54**	1.50E-11	1.70E-11
				Contact	0.87	0.8	0.54	0.43	0.96	0.95	0.56	0.47	4.50E-12	4.50E-09
				mRMR	0.88	0.79	0.56	0.33	0.96	0.98	**0.58**	0.44	6.30E-13	6.00E-08
				IDEpi	**0.89**	0.80					**0.58**	0.48		
V2	CAP256-VRC26.25	0.63	0.72	AllSig	0.76	**0.75**	0.64	0.23	0.84	0.95	0.49	0.27	4.70E-12	8.90E-04
				Contact	0.73	0.73	0.64	0.38	0.78	0.87	0.42	**0.28**	2.90E-09	5.20E-04
				mRMR	**0.8**	0.72	0.6	0	0.92	0.99	**0.57**	-0.05	4.80E-16	1
				IDEpi	0.78	0.72					0.52	-0.05		
V2	PGDM1400	0.8	0.74	AllSig	**0.89**	0.86	0.51	0.44	0.99	1	0.63	0.61	1.90E-15	4.20E-14
				Contact	0.86	0.84	0.46	0.44	0.95	0.98	0.49	0.56	5.90E-10	6.90E-12
				mRMR	0.9	**0.88**	0.59	0.6	0.98	0.98	**0.65**	**0.69**	1.40E-16	1.90E-17
				IDEpi	0.87	0.84					0.5	0.56		
V2	PGT145	0.75	0.71	AllSig	0.81	0.82	0.44	0.43	0.93	0.98	0.42	0.55	5.90E-08	7.50E-12
				Contact	0.81	0.79	0.5	0.45	0.91	0.93	0.45	0.46	4.90E-09	1.20E-08
				mRMR	**0.84**	**0.83**	0.5	0.41	0.95	1	**0.53**	**0.57**	9.00E-12	2.10E-13
				IDEpi	0.82	0.80					0.43	0.47		
V3	10-1074	0.66	0.63	AllSig	0.92	**0.95**	0.85	0.89	0.96	0.99	0.83	**0.9**	1.80E-34	1.30E-36
				Contact	**0.93**	0.92	0.87	0.86	0.96	0.95	**0.84**	0.83	1.20E-35	1.60E-29
				mRMR	**0.93**	0.93	0.85	0.84	0.97	0.98	**0.84**	0.85	1.50E-35	6.80E-32
				IDEpi	0.92	0.92					**0.84**	0.84		
V3	PGT121	0.63	0.69	AllSig	0.79	0.8	0.64	0.66	0.87	0.87	0.53	0.53	2.70E-14	9.00E-12
				Contact	**0.83**	0.73	0.74	0.53	0.88	0.82	**0.62**	0.36	3.70E-19	6.20E-06
				mRMR	0.82	0.79	0.68	0.58	0.9	0.88	0.61	0.49	2.20E-18	4.20E-10
				IDEpi	0.81	**0.83**					0.59	**0.60**		
V3	PGT128	0.62	0.54	AllSig	**0.78**	**0.79**	0.68	0.67	0.84	0.88	**0.53**	**0.57**	4.50E-14	3.70E-14
				Contact	0.74	0.76	0.59	0.66	0.84	0.85	0.45	0.52	3.30E-10	4.10E-12
				mRMR	0.76	0.75	0.63	0.54	0.84	0.93	0.48	0.52	7.60E-12	5.70E-12
				IDEpi	0.70	0.73					0.39	0.45		
CD4bs	VRC01	0.9	0.79	AllSig	**0.93**	0.82	0.4	0.35	0.99	0.94	**0.54**	0.37	9.60E-08	1.70E-05
				Contact	0.92	**0.83**	0.4	0.43	0.98	0.93	0.48	**0.43**	9.40E-07	5.10E-07
				mRMR	**0.93**	**o.83**	0.3	0.3	1	0.98	0.53	0.41	4.90E-07	2.60E-06
				IDEpi	0.74	**0.83**					0.26	0.39		
CD4bs	3BNC117	0.85	0.8	AllSig	**0.9**	0.83	0.46	0.51	0.97	0.91	**0.54**	0.45	2.70E-09	8.30E-08
				Contact	0.88	0.78	0.46	0.57	0.95	0.83	0.48	0.38	6.80E-08	3.60E-06
				mRMR	0.88	**0.87**	0.32	0.49	0.99	0.96	0.47	**0.54**	6.60E-07	4.80E-10
				IDEpi	0.86	0.83					0.22	0.37		
CD4bs	VRC07	0.93	0.87	AllSig	**0.95**	**0.88**	0.46	0.41	0.99	0.95	**0.56**	**0.4**	1.10E-06	2.10E-05
				Contact	0.94	**0.88**	0.31	0.41	0.99	0.95	0.43	**0.4**	2.40E-04	2.10E-05
				mRMR	0.94	**0.88**	0.23	0.14	1	0.99	0.47	0.27	3.00E-04	6.60E-03
				IDEpi	0.87	0.85					0.15	0.2		
CD4bs	VRC07-523-LS	0.96	0.95	AllSig	0.96	0.94	0.5	0.38	0.98	0.97	0.52	0.34	4.00E-05	3.30E-03
				Contact	0.97	**0.96**	0.38	0.38	0.99	0.99	0.52	**0.45**	1.80E-04	6.30E-04
				mRMR	0.97	0.95	0.38	0.25	1	0.99	**0.6**	0.33	4.60E-05	1.10E-02
				IDEpi	**0.98**	**0.96**					0.4	**0.45**		
MPER	10E8	0.98	0.98	AllSig	0.98	0.97	0.4	0.25	1	0.98	0.51	0.23	1.40E-03	9.00E-02
				Contact	**0.99**	**0.98**	0.6	0.25	1	0.99	**0.66**	**0.34**	2.70E-05	4.60E-02
				mRMR	0.97	**0.98**	0	0	0.99	1	0.02	0	1	1
				IDEpi	0.88	0.94					0.04	0.28		
MPER	4E10	0.98	0.93	AllSig	**0.98**	0.92	0.25	0	1	0.99	**0.34**	-0.02	3.80E-02	1
				Contact	**0.98**	0.93	0.25	0	1	1	**0.34**	0	3.80E-02	1
				mRMR	**0.98**	**0.94**	0	0.08	1	1	0	**0.28**	1	6.90E-02
				IDEpi	0.94	0.92					0	-0.02		

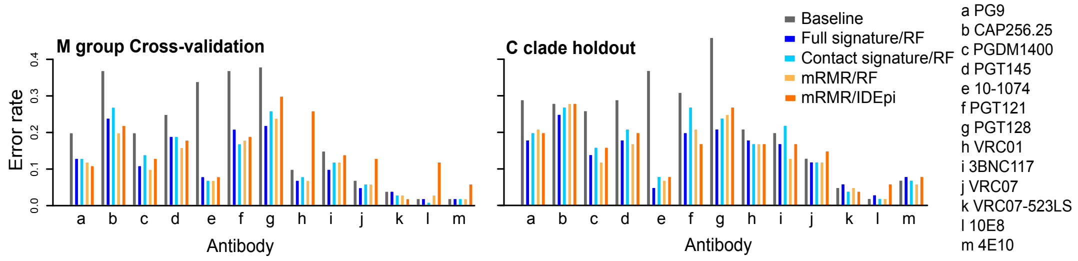

Table S5. Machine learning classification prediction statistics, comparing strategies for input feature selection, supporting Figure 4 in the main text. (A) Random Forest predictions of positive/negative IC_{50} values (above or below the threshold of detection at the highest concentration of antibody used). The global M group dataset 4 was evaluated for accuracy of predictions using leave-one-out cross-validation (the "Xval" columns). Then the M group data was used as a training set and the accuracy of predictions evaluated using the holdout set from C clade (the "Hout" columns). The fraction of true positives for each antibody in the M group data and the C clade hold out set is provided. It is considered the null or "default" model; for example, if 63% of Envs were sensitive to an antibody, and you guessed a new set of Envs was all sensitive, by "default" you would be correct 63% of the time. We also present the Matthews Correlation Coefficient (MCC) (ranging from 0 to 1, 0 being a no better than random, 1 a perfect prediction), and a Fisher's exact p-value, the level of significance indicating if the prediction is better than a default prediction based on true positives. As an example, for 10-1074, 63% of the C clade viruses are sensitive and the full signature machine learning predictions called 95% of these correctly as positive/negative. This high accuracy gave an MCC of 0.90 and a very low p-value. In contrast, for an antibody like 10E8, almost all Envs are sensitive to the antibody (98%), so the accuracy of the prediction is very high (97%), but this is essentially no better than just guessing all Envs are sensitive, so the MCC is 0.23 and the p-value it not significant. We also provide a summary of the accuracy of the positive calls and the negative calls independently; we have a low frequency of false positives and we have a higher frequency of false negatives. This is an expected outcome as the antibodies studied here are broadly reactive, i.e. there are many more positive than negative pseudoviruses for each antibody studied and the RF is optimized to achieve overall accuracy. It may be possible to tune this outcome by weighting the classes differently (e.g., with more weight on the negative class to improve the accuracy of the negative calls), if this was important for future studies, but this would come at a cost of lower overall accuracy.

As with regression, we varied the input filters using the same RF machine learning strategy. "AllSig" indicates all signatures were used in the pre-filter, "Contact" indicates only contact signatures were used, and "mRMR" indicates a standard mRMR pre-filter was used. We also ran IDEpi on our data to compare our results using mRMR to ensure we were getting reasonable results relative to the existing literature (Hepler et al., 2014) . We evaluated leave-10-out and leave-50-out using IDEpi; we present leave-50-out, as this was most predictive. The bold values indicate the best score for each antibody. Our implementation of mRMR gave scores that were generally comparable to IDEpi. The overall accuracies and MCC scores were generally roughly comparable, and unlike regression predictions, no particular input filter was favored. The features from the alignments that had the highest importance are listed in the footnote, see Table S4 above for the key.

***CLASSIFICATION footnotes:** organized following the footnotes in Table S8:

CAP256-VRC26.25	3/3	R315**Q** V169**K** R166**R**
PG9	10/11	O160**O** t138d K171**K** -353b**-** -132k**-** F717**F** -787b**S** h330y N677**N** s440q
PGDM1400	9/9	O160**O** v169e -787e**G** l619l q170- s668s m426r s440q -787d**K**
PGT145	10/96	O160**O** G167**D** g167g s446i r166g 189gn v169e t402w 132f**-** 031cn
10-1074	1/1	O332**O**
PGT121	10/92	O332**O** s334o n325k N325**D** n340r e620n S334**S** 363ap O301**O** V255**V**
PGT128	10/18	O332**O** O295**O** i320- T297**T** O301**O** H330**H** I165**I** q442e R304**R** k151q
3BNC117	2/2	G459**G** R456**R**
VRC01	1/1	R456**R**
VRC07	10/69	R456**R** G459**G** g471i -132qd N280**N** r304k 363b**-** N355- q352i -787bi
VRC07-523-LS	3/3	r456s g471i G459**G**
10E8	1/1	n671t
4E10	1/1	f673l
2F5	3/3	K665**K** A667**A** E662**E**

Underneath the table is an illustration of the error based on the data in the table. For all bNAbs, frequencies of errors for classification predictions are shown. The grey bar on the left of each set is the baseline null model that predicts everyone is positive, and the error is then just the frequency of viruses that were actually negative. Different machine learning strategies are compared for their impact on classification accuracy for calling Env sensitivity to a given antibody – the lower the bar, the more accurate the predictor. The 3 different input filters we tried were generally better than baseline (lower than the grey bar), and similar to IDEpi predictions, with a few exceptions. Machine learning classification predictions for V3 glycan antibodies may be particularly helpful. For bNAbs that have almost complete breadth naturally (e.g. VRC07-523-LS, 10E8, and 4E10), the simple baseline of "assuming positive" performs extremely well and machine learning predictions do not add much value. The 13 antibodies included in this figure were those that had data available in both the training set (data set 4) and the holdout set (data set 3).

Table S6. Machine learning informative feature contributions. Supporting Fig. 4. This table presents ranked signature features that were among the top 10 most informative sites for different antibodies of different classes. It has four sections, one for each antibody class, and each section lists the most important signature features for Classification and Regression predictions for each antibody tested from dataset 4. On the right are summaries of the number of times recurrent features are found. Different shades highlight different V loop characteristics. Bold indicates sites that were repeatedly informative for a number of antibodies (at least 4/11 times for CD4bs bNAbs, 3/6 times for V2 bNAbs, 3/6 times for V3 bNAbs, and 2/3 for MPER bNAbs). Blue indicates signature positions within epitope contact regions, black are positions outside the contact region. NxST N332 and NxST N160 indicate a PNGS. Clades were only rarely among the most informative data for prediction.

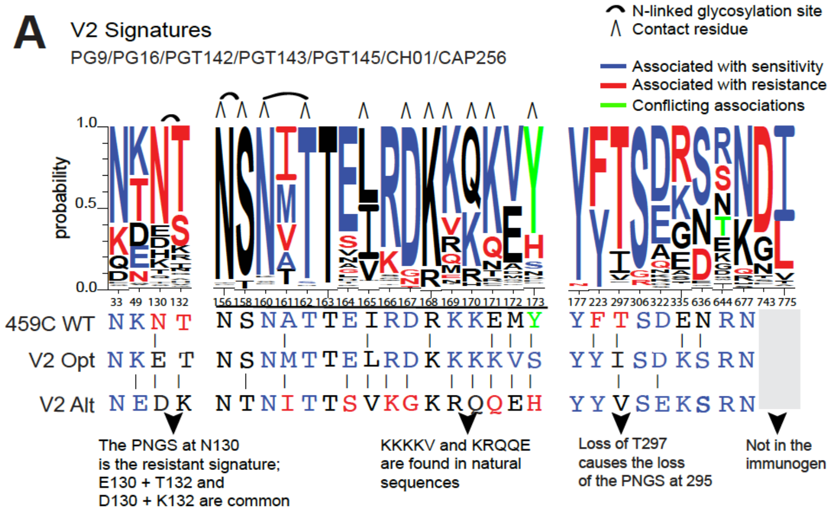

B

Sequence Name | **Sequence Alignment** | **End Position**

```
HXB2 position                      49
C.459             GNLWVTVYYGVPVWREAKTTLFCASDAKAYDREVHNVWATHACVPTDPNPQEIVLENVTENFNMWKNDMVDQ   72
V2glycan.opt      ------------------------------------------------------------------------   72
V2glycan.alt      -----------------E------------------------------------------------------   72

HXB2 position                                130                                       160
C.459             MHEDIISLWDQSLKPCVKLTPLCVTLNCTNVTSSAANVTSNVTNDANNASNANGRNVINEDMQNCSFNATTE  144
V2glycan.opt      --------------------------E--AFNSSSHTNSSIAMQEMK................N----M---  128
V2glycan.alt      --------------------------D-KAFNSSSHTNSSIAMQEMK................N-T--I--S  128
CRF02.T250_4                                DCQAFNSSSHTNSSIAMQEMK ---------------NCSFNVTTE

HXB2 position              169                 190                                223
C.459             IRDRKKEMYALFYKLDIVPLDGEKSDNRYRLINCNTSTLTQACPKVSFDPIPIHYCTPAGFAILKCNNKTFN  216
V2glycan.opt      L--K--KVS-------------..NKNGRQ------------------------------Y-----------  198
V2glycan.alt      VKGKRQQEH-------------..NKNGRQ------------------------------Y-----------  198
CRF02.T250_4      LRDKKKKEYSFFYKTDIEQI..NKNGRQ

HXB2 position                                                                     297
C.459             GTGPCNNVSTVQCTHGIKPVVSTQLLLNGSLAEEDIIIRSENLTNNAKTIIVHLNESVEIVCTRPNNNTRKS  288
V2glycan.opt      --------------------------------------------------------------I---------  270
V2glycan.alt      --------------------------------------------------------------V---------  270

HXB2 position                  321           335
C.459             IRIGPGQTFYANNDIIGDIRQAHCNISEEKWNNTLHRVWKKLVEHFPNKTTIRFDRHSGGDLEITTHSFNCG  360
V2glycan.opt      ---------------------------K--------------------------------------------  342
V2glycan.alt      -------------E-------------K--------------------------------------------  342

C.459             GEFFYCNTSGLFNITYNSNYTYNDTKHNGTKVITLPCRIKQIINMWQEVGRAMYAPPIAGNITCTSNITGLL  432
V2glycan.opt      ------------------------------------------------------------------------  414
V2glycan.alt      ------------------------------------------------------------------------  414

C.459             LTRDGGNNSTETETFRPGGGDMRDNWRSELYKYKVVEIKPLGIAPTGAKRRVVEREKRAVGIGAVFLGFLGA  504
V2glycan.opt      ------------------------------------------------------------------------  486
V2glycan.alt      ------------------------------------------------------------------------  486

C.459             AGSTMGAASITLTVQARQLLSGIVQQQSNLLKAIEAQQHLLQLTVWGIKQLQTRVLAIERYLKDQQLLGLWG  576
V2glycan.opt      ------------------------------------------------------------------------  558
V2glycan.alt      ------------------------------------------------------------------------  558

HXB2 position                                          636
C.459             CSAKLICTTAVPWNSSWSNKSETEIWNNMTWMQWDREINNYTNTIYRLLEESQNQQEKNENDLLALDKWNSL  648
V2glycan.opt      --------------------------------------S---------------------------------  630
V2glycan.alt      --------------------------------------S---------------------------------  630

C.459             WDWFGISNWLWYIR                                                            662
V2glycan.opt      --------------                                                            644
V2glycan.alt      --------------                                                            644
```

Table S7. Signature vaccine design based on dataset 1 and dataset 2, providing background detail in support of Figure 5. (A) The modifications made to 459C WT to create the trivalent vaccine designs for V2 Opt and V2 Alt immunogens. The signatures used for vaccine design were generated at a time when only datasets 1 and 2 were available and the antibodies informing the signature at that time are listed at the top. The contact region for each class of antibody is underlined and contact residues and PNGS are highlighted (see the key, top right). Red amino acids were associated with resistance, blue with sensitivity, green with either sensitivity or resistance depending on which antibody was assessed in the set, and black had no statistical support for an association. The size of each letter in the LOGO corresponds to the frequency of that AA in the M group panel data and we attempted to capture common AAs in signature sites at a global level. In both Figures A and B, 459C WT is shown under the LOGO in the same signature positions, AAs are colored in blue, red, green, or black according to their neutralization associations at the population level, and changes made in the Opt or Alt designs are indicated by vertical lines. Comments related to decisions about specific modifications are included; in general, we tried to represent relative diversity within the epitope (the underlined region), including AAs associated with resistance, favoring more common AAs, and grouping neighboring AAs to yield common motifs. Outside of the epitope we favored sensitivity, but if this could be achieved with inclusion of distinct variants that were either both sensitive or neutral signatures, we included the variant in the Alt versions to better represent natural HIV-1 diversity. **(B) Precise modifications introduced into the V2 SET vaccine mapped on to protein sequences.** 459C WT (GenBank accession JN681242) baseline gp140 protein sequence, with modifications introduced based on part (A). The CRF02.T250_4 (GenBank accession EU513189) V1 and V2 regions replaced those of 459C WT and these regions are shown in green. CRF02.T250_4 was exquisitely sensitive to both V2 and V3 bNAbs and had desirable loop characteristics for both classes of antibodies (though it was resistant to CD4bs bNAbs) (Fig. S1). Our intent was to enhance V2 responses, but thought that V3 epitope accessibility in the SET Alt and Opt designs might also be improved by using a hybrid of 459C WT with CRF02.T250_4 V1 and V2 regions. The periods (.) indicate gaps to maintain the alignment; T250 is shorter in these regions than 459C. The dashes (-) indicate identity with 459C WT. Key HXB2 position numbers are indicated above the alignment.
