# Supplementary material for: HIV-1 Neutralizing Antibody Signatures and Application to Epitope-Targeted Vaccine Design
Source: Cell Host Microbe. 2019 Jan 9;25(1):59–72.e8. doi: 10.1016/j.chom.2018.12.001 (PMC6331341; doi:10.1016/j.chom.2018.12.001)
Supplement: Document S2. Article plus Supplemental Information [file mmc4.pdf]

Article

Cell Host & Microbe

HIV-1 Neutralizing Antibody Signatures and Application to Epitope-Targeted Vaccine Design

Graphical Abstract

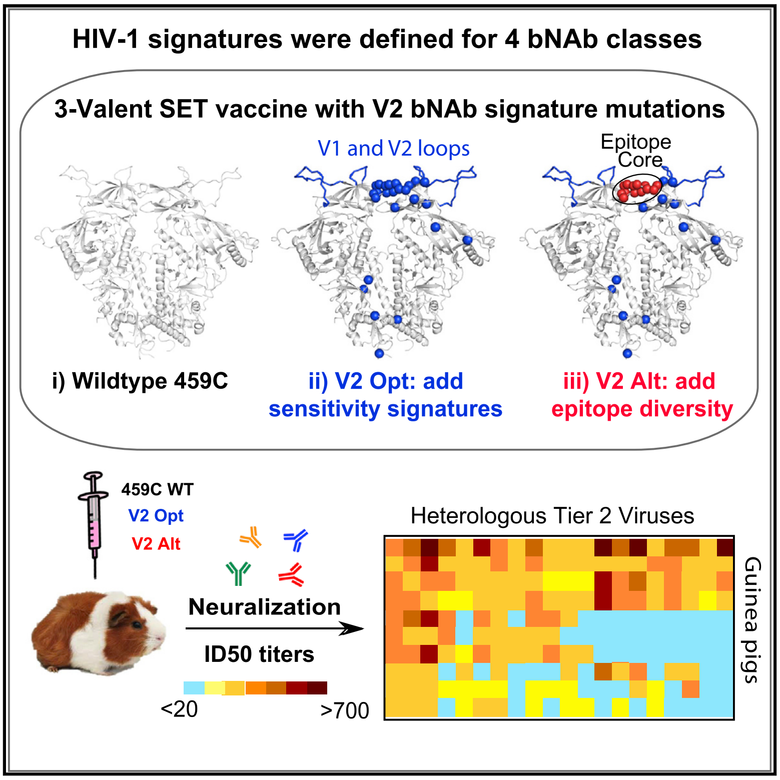

Authors

Christine A. Bricault, Karina Yusim, Michael S. Seaman, ..., David C. Montefiori, Dan H. Barouch, Bette Korber

Correspondence

dbarouch@bidmc.harvard.edu (D.H.B.), btk@lanl.gov (B.K.)

In Brief

HIV-1 Env amino acid signatures associated with sensitivity to broadly neutralizing antibodies were systematically defined from large neutralization panels. V2 signatures were incorporated in a trivalent vaccine to enhance epitope exposure and to include common epitope variants, resulting in increased neutralization breadth against heterologous viruses in a guinea pig model.

Highlights

- HIV-1 bNAb sensitivity signatures from 4 large virus panels mapped across 4 Ab classes
- Non-contact hypervariable region characteristics are critical for bNAb sensitivity
- HIV-1 Env 459C used alone as a vaccine can elicit modest tier 2 NAbs in guinea pigs
- V2 bNAb signature-guided modifications in 459C enhanced neutralization breadth

Bricault et al., 2019, Cell Host & Microbe *25*, 59–72
January 9, 2019 © 2018 The Author(s). Published by Elsevier Inc.
https://doi.org/10.1016/j.chom.2018.12.001

CellPress

HIV-1 Neutralizing Antibody Signatures and Application to Epitope-Targeted Vaccine Design

Christine A. Bricault,[1,17] Karina Yusim,[2,3,17] Michael S. Seaman,[1] Hyejin Yoon,[2] James Theiler,[2,3] Elena E. Giorgi,[2,3] Kshitij Wagh,[2,3] Maxwell Theiler,[2] Peter Hraber,[2] Jennifer P. Macke,[2] Edward F. Kreider,[4] Gerald H. Learn,[4] Beatrice H. Hahn,[4] Johannes F. Scheid,[5,6] James M. Kovacs,[7,8,9] Jennifer L. Shields,[1] Christy L. Lavine,[1] Fadi Ghantous,[1] Michael Rist,[1] Madeleine G. Bayne,[1] George H. Neubauer,[1] Katherine McMahan,[1] Hanqin Peng,[7,8] Coraline Chéneau,[1] Jennifer J. Jones,[10] Jie Zeng,[10] Christina Ochsenbauer,[10] Joseph P. Nkolola,[1] Kathryn E. Stephenson,[1,11] Bing Chen,[7,8] S. Gnanakaran,[2,3] Mattia Bonsignori,[12,13] LaTonya D. Williams,[12] Barton F. Haynes,[12,13,14] Nicole Doria-Rose,[15] John R. Mascola,[15] David C. Montefiori,[12,16] Dan H. Barouch,[1,11,18,19,*] and Bette Korber[2,3,18,*]
[1]Center for Virology and Vaccine Research, Beth Israel Deaconess Medical Center, Boston, MA 02215, USA
[2]Los Alamos National Laboratory, Los Alamos, NM 87545, USA
[3]New Mexico Consortium, Los Alamos, NM 87545, USA
[4]Departments of Medicine and Microbiology, Perelman School of Medicine, University of Pennsylvania, Philadelphia, PA 19104, USA
[5]Massachusetts General Hospital, Boston, MA 02114, USA
[6]Harvard Medical School, Boston, MA 02114, USA
[7]Division of Molecular Medicine, Children's Hospital, Boston, MA 02115, USA
[8]Department of Pediatrics, Harvard Medical School, Boston, MA 02115, USA
[9]Departments of Chemistry and Biochemistry, University of Colorado, Colorado Springs, CO 80918, USA
[10]Department of Medicine and CFAR, University of Alabama at Birmingham, Birmingham, AL 35294, USA
[11]Ragon Institute of Massachusetts General Hospital, MIT, and Harvard, Boston, MA 02114, USA
[12]Duke Human Vaccine Institute, Duke University School of Medicine, Durham, NC 27710, USA
[13]Department of Medicine, Duke University School of Medicine, Durham, NC 27710, USA
[14]Department of Immunology, Duke University School of Medicine, Durham, NC 27710, USA
[15]Vaccine Research Center, National Institute of Allergy and Infectious Diseases, National Institutes of Health, Bethesda, MD 20814, USA
[16]Department of Surgery, Duke University School of Medicine, Durham, NC 27710, USA
[17]These authors contributed equally
[18]These authors contributed equally
[19]Lead Contact
*Correspondence: dbarouch@bidmc.harvard.edu (D.H.B.), btk@lanl.gov (B.K.)
https://doi.org/10.1016/j.chom.2018.12.001

SUMMARY

Eliciting HIV-1-specific broadly neutralizing antibodies (bNAbs) remains a challenge for vaccine development, and the potential of passively delivered bNAbs for prophylaxis and therapeutics is being explored. We used neutralization data from four large virus panels to comprehensively map viral signatures associated with bNAb sensitivity, including amino acids, hypervariable region characteristics, and clade effects across four different classes of bNAbs. The bNAb signatures defined for the variable loop 2 (V2) epitope region of HIV-1 Env were then employed to inform immunogen design in a proof-of-concept exploration of signature-based epitope targeted (SET) vaccines. V2 bNAb signature-guided mutations were introduced into Env 459C to create a trivalent vaccine, and immunization of guinea pigs with V2-SET vaccines resulted in increased breadth of NAb responses compared with Env 459C alone. These data demonstrate that bNAb signatures can be utilized to engineer HIV-1 Env vaccine immunogens capable of eliciting antibody responses with greater neutralization breadth.

INTRODUCTION

Vaccine induction of broadly neutralizing antibodies (bNAbs) against diverse global tier 2 HIV-1 strains remains an unsolved challenge for the HIV-1 vaccine field. There has been some progress in animal models (Escolano et al., 2016; Saunders et al., 2017), but human trials have yet to elicit bNAbs, although NAbs with varying levels of breadth arise during natural infection (Hraber et al., 2014b). bNAbs typically develop slowly during chronic infection as the virus diversifies under immune pressure and B cell lineages adapt to the evolving virus (Bonsignori et al., 2017b; Doria-Rose et al., 2014; Liao et al., 2013; Wu et al., 2015). bNAb breadth and potency are evaluated using large panels of HIV-1 Envelope (Env) pseudoviruses that sample global HIV-1 diversity (Hraber et al., 2014a) or the C clade diversity of Southern Africa (Rademeyer et al., 2016).

We used data from 4 large neutralization panels for a more comprehensive mapping of viral signatures associated with bNAb sensitivity than undertaken previously (Chuang et al., 2013; Evans et al., 2014; Ferguson et al., 2013; West et al., 2013). Signature sites were identified using a strategy that incorporates a phylogenetic correction (Gnanakaran et al., 2010) for amino acids (AAs) and potential N-linked glycosylation sites (PNGSs) (Crispin and Doores, 2015), and we also explored the impacts of hypervariable region characteristics

 © 2018 The Author(s). Published by Elsevier Inc.
This is an open access article under the CC BY license (http://creativecommons.org/licenses/by/4.0/).

and clades. Recurrent signature patterns were found among bNAbs with shared specificities (Burton and Hangartner, 2016).

We next used variable V2 apex (V2) epitope bNAb signatures to inform HIV-1 Env immunogen design in a proof-of-concept exploration of an approach we call signature-based epitope targeted (SET) vaccines. Other vaccine design strategies include engaging bNAb germline precursors (Steichen et al., 2016), using polyvalent sets to capture diversity (Korber et al., 2017), lineage-based designs (Bonsignori et al., 2017b), and engineered native-like Envs (e.g., SOSIPs) (Sanders et al., 2013; Steichen et al., 2016). SOSIP vaccines elicit robust autologous NAbs that have limited breadth in rabbits and non-human primates (NHPs) (Pauthner et al., 2017; Sanders et al., 2015).

Our SET vaccine design started with the Env 459C (Bricault et al., 2015), as 459C alone elicited modest neutralization of some tier 2 heterologous strains in guinea pigs. V2-SET immunogens are a trivalent combination of 459C wild-type (WT) plus two additional proteins designed by modifying 459C to include V2 bNAb signatures intended to both enhance V2 epitope exposure and include relevant variation. V2-SET vaccines expressed as either gp140 SOSIP trimers or foldon trimers elicited increased NAb breadth compared to 459C alone in guinea pigs, suggesting the potential utility of bNAb signatures in vaccine design.

RESULTS

Neutralization Data

Four datasets measuring the sensitivity of bNAbs against panels of HIV-1 Envs were analyzed. Three panels sampled global viral diversity (Hraber et al., 2014a), and the other sampled only C clade, which dominates in Southern Africa (Rademeyer et al., 2016). Table S1 summarizes bNAb dataset inclusion, relationships, and provenance. bNAbs are grouped by epitope class: V2, V3 glycan (V3), CD4 binding site (CD4bs), and membrane proximal external region (MPER) (Burton and Mascola, 2015). V2 and V3 bNAbs often have great potency but limited breadth, CD4bs have expanded breadth, and MPER has high breadth but low potency (Figures 1, S1, and S2). Heatmaps displaying inhibitory concentrations of 50% (IC_{50}) data illustrate shared pattern sensitivity across bNAb classes (Figures 1 and S1), enabling the definition of common bNAb class signatures.

Neutralization Sensitivity and HIV-1 Clades

bNAb sensitivity patterns are associated with HIV-1 clades, which have geographic associations (Korber et al., 2009). The V2 bNAb lineage CAP256.VRC26 is less potent against clade B viruses (Doria-Rose et al., 2016); diminished clade B potency extends across the V2 bNAb class (Figures 1, 2A, and S2). CD4bs bNAbs are more sensitive to clade A viruses, and some bNAbs are less sensitive to C clade, in particular 3BNC117 and VRC01 (Figures 1, 2A, and S2). MPER bNAbs are less potent against clade A. Circulating Recombinant Form 01 (CRF01) viruses, common in Southeast Asia, are extremely resistant to V3 bNAbs (Figures 1 and S2), which is driven by a PNGS shift from positions N332 to N334 found in 96% of CRF01 viruses (Figure 1; Table S2). The N332 glycan directly contacts V3 bNAbs (Kong et al., 2013) and is essential for some, although PGT128 can tolerate its loss (Figure 1). CRF01 was already highly diverse in central Africa when a founder seeded the Thai epidemic (Korber et al., 2000); African CRF01 viruses also lack the PNGS at N332. PNGS N332 frequencies vary between clades, correlating with overall clade sensitivity to V3 bNAbs (Table S2); e.g., ~40% of A clade viruses are resistant to most V3 bNAbs, and 43% lack the PNGS N332 (Figure 2A; Table S2). The N332 PNGS is under-represented among transmitted C clade viruses (Rademeyer et al., 2016), which may impact the efficacy of V3 bNAbs in southern Africa.

Amino Acid Signatures

Signature patterns were identified by testing each AA and PNGS across Env alignments for associations with sensitivity to each bNAb in each of the 4 neutralization panels (Figure S1), based on associations with either potency or detectable neutralization (Tables S3A–S3D; Figure 3). Key signature sites were initially identified using a method that corrects for phylogenetic artifacts (Gnanakaran et al., 2010) (Tables S3E–S3H). Once a site was deemed of interest by this stringent criterion, any significant associations within these key sites were listed (Tables S3I–S3L).

CD4bs bNAbs

While many CD4bs signatures are in contacts (Figures 3 and S3; Table S3) and known to influence sensitivity (Gao et al., 2014; Lynch et al., 2015), most are outside of contact surfaces. We tested two CD4bs resistance signatures directly by introducing them into the CH505 transmitted-founder (TF) backbone. The G458Y signature mutation conferred complete resistance (IC_{50} > 25 μg/mL) to VRC01 and 3BNC117, and both can neutralize the CH505 TF (IC_{50} of 0.14 and 0.03 μg/mL, respectively). The non-contact T234N signature substitution introduced a PNGS at N234 that increased resistance to CD4bs bNAbs 5- to 7-fold, and IC_{50} titers went from 0.36 to 1.82 μg/mL for CH235 and from 0.12 to 0.87 μg/mL for VRC01.

MPER bNAbs

As their breadth is so great, most 10E8, 4E10, and DH511 signatures are associated with potency, except for rare mutations associated with complete resistance: W672L, F673L, and W680G (Figures 3 and S3; Table S3). The 10E8-specific resistance signature N671T accounts for the increased breadth of DH511 lineage bNAbs over 10E8 (Williams et al., 2017). The MPER epitope signature K683R is also associated with resistance to CD4bs and V2 bNAbs (Figure 3; Table S3), consistent with MPER changes impacting overall neutralization sensitivity (Bradley et al., 2016). Clade C resistance to 2F5 may be explained by clade C not having an otherwise conserved Ala, A667 (Figure S3).

V3 bNAbs

Glycans that interact directly with V3 bNAbs (Behrens et al., 2016) and are positive signatures for neutralization are at positions N332, N301, and N295 (Figures 3 and S3; Table S3). Similar to CD4bs and MPER bNAbs, V3 bNAb sensitivity signatures in the epitope were relatively conserved, with positive signatures as the most common variant (Figure 3). In contrast, V3 bNAb signature sites between positions 336–442 were extremely variable and so may contribute to more nuanced levels of potency. In

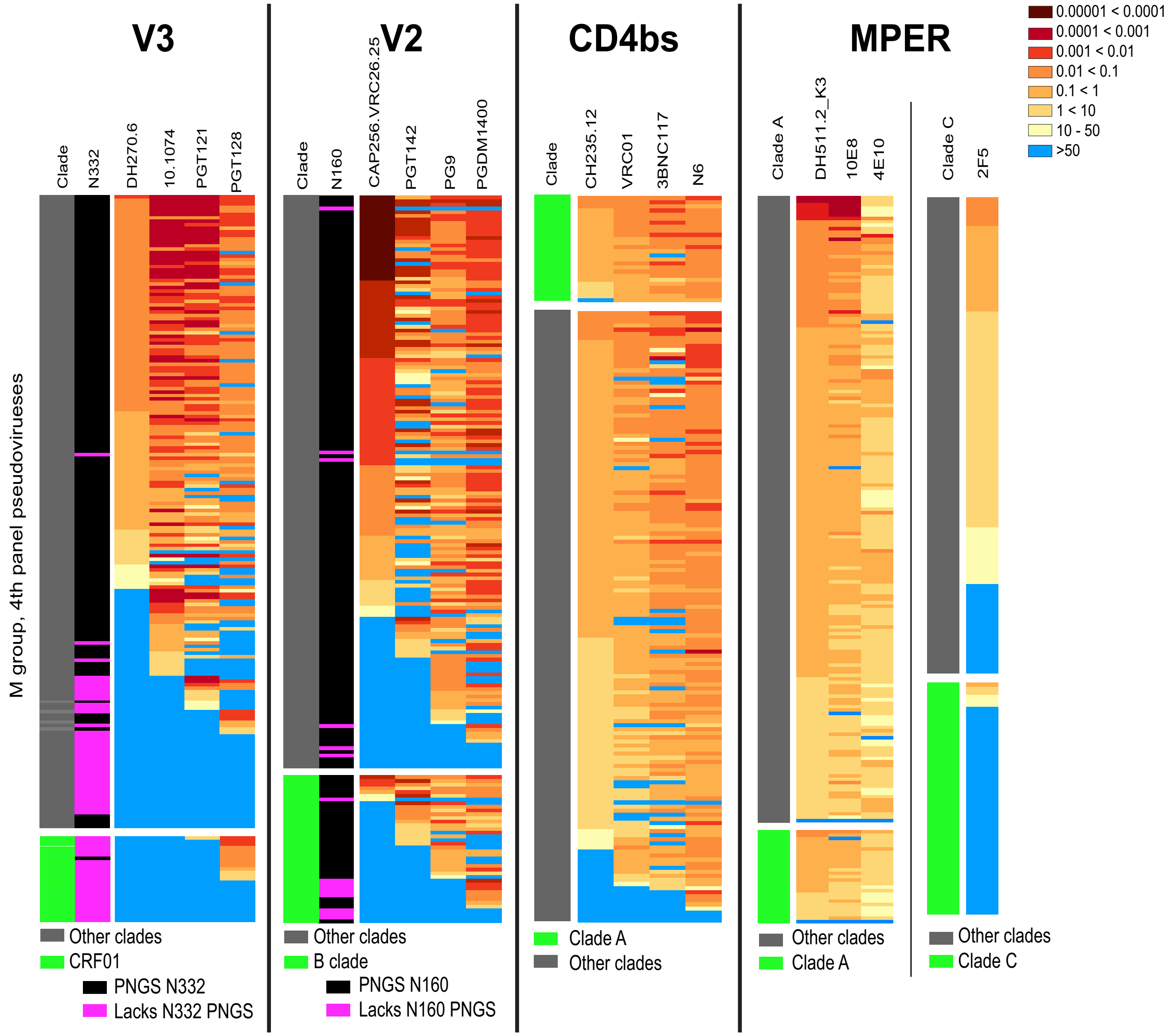

Figure 1. Heatmaps Showing IC_{50} Neutralization Titers for Dataset 4

Darker red hues indicate more potent neutralization and blue indicates undetected responses. Rows represent pseudoviruses, ordered differently in each panel to highlight commonalities in neutralization profiles across bNAbs in each class. The clade with the strongest clade effect is separated and indicated in green to the left. Key PNGSs are indicated by magenta. Among MPER bNAbs 2F5 is considered separately as it has a unique epitope.

the GDIR contact motif (Sok et al., 2016) only D325 is a signature, because the other 3 positions are nearly invariant.

V2 bNAbs

V2 bNAbs contact glycans at positions N156 and N160. The PNGS N160 is critical for many V2 bNAbs, with a few exceptions that may be enabled by nearby glycans acting in compensatory role (Figure 1; Table S3) (McLellan et al., 2011). In contrast, 11 viruses of the 380 unique viruses in the combined datasets 3 and 4 lacked the PNGS at N156, and these 11 viruses had very similar distributions of IC_{50} titers to viruses that had the PNGS site for all V2 bNAbs tested. PNGSs at positions N130 and N332 are associated with V2 bNAb resistance. The glycan at N130 is near the CAP256.VRC26 contact surface (Figure S3) and it interacts with CH03 (Gorman et al., 2016), but glycans at N130 and N332 may also act indirectly through glycan dynamics (Stewart-Jones et al., 2016) or carbohydrate processing (Behrens et al., 2016). Many V2 bNAb contact signatures have been studied: K169 and K171 loss, and Q170K, confers resistance, while E/D164 increases CAP256.VRC26.25 sensitivity (Doria-Rose et al., 2012, 2016).

Evolutionary Counter-pressure

Some resistance signatures come with a fitness cost (Lynch et al., 2015). Also, some signatures are associated with resistance or

CellPress

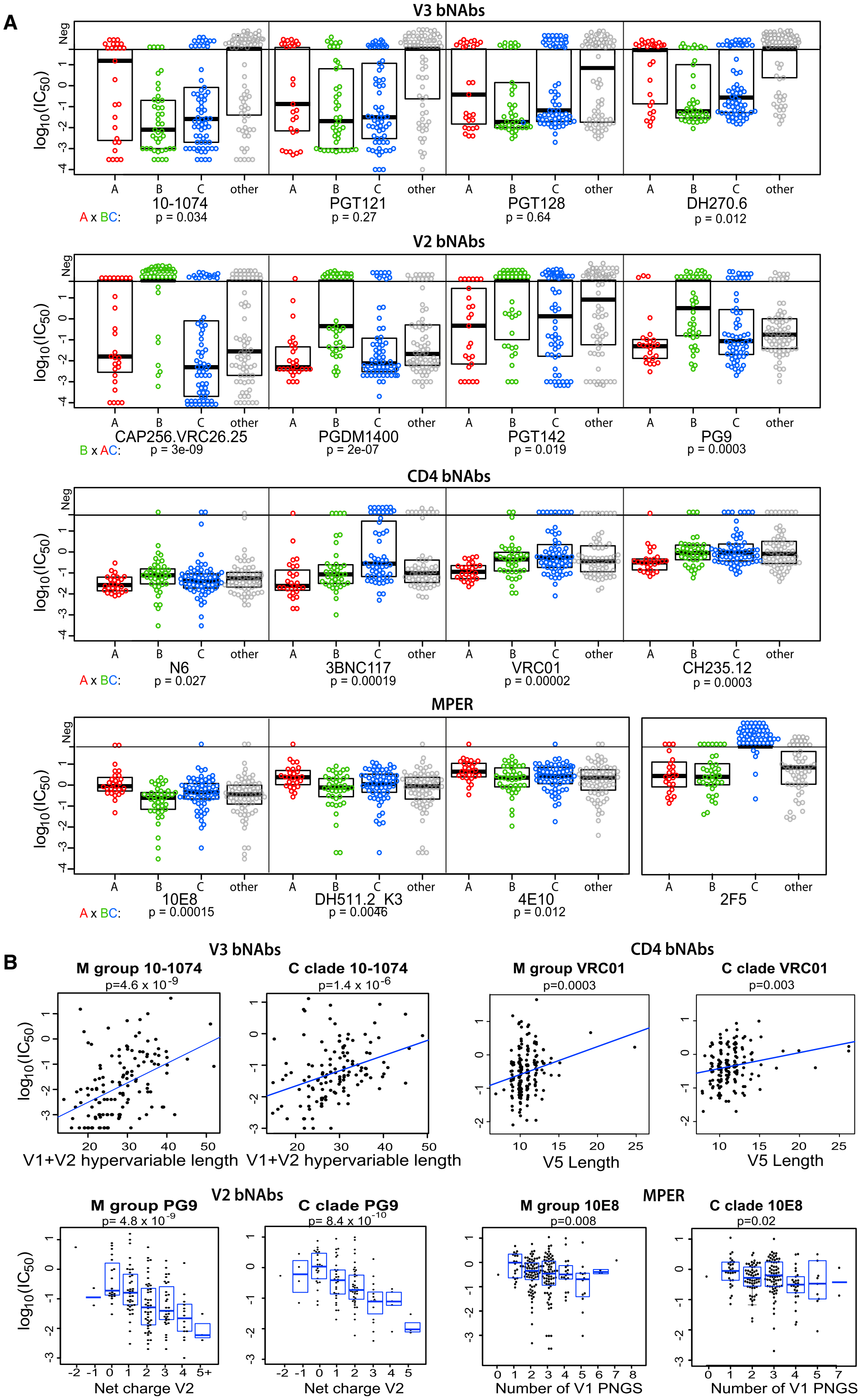

(legend on next page)

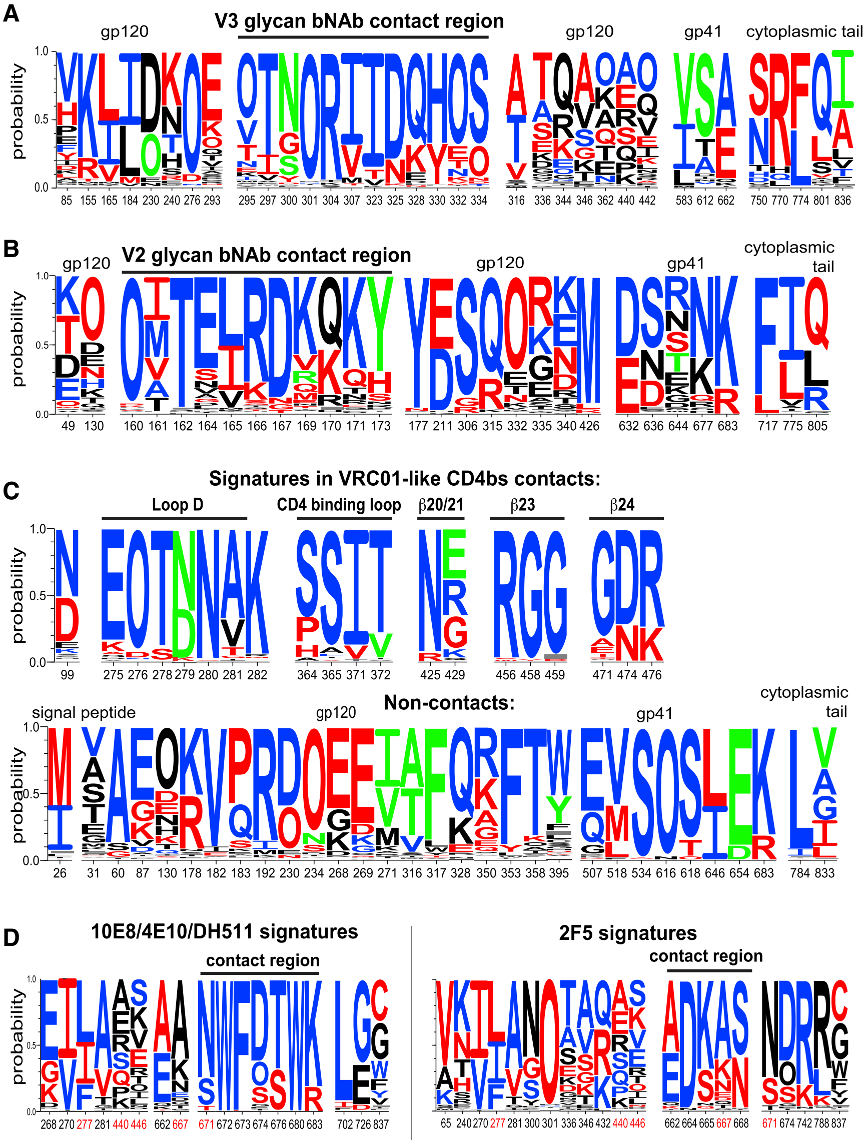

Figure 3. Sequence LOGOs of AA Signatures by Antibody Class

This figure highlights the more robust signature sites in that they were supported by multiple lines of evidence—they either had phylogenetically corrected associations supported by at least 2 datasets, were a signature site in a contact residue, or both. Not all bNAbs in a class are associated with every signature. Complete lists with detailed statistics are provided in Table S3. Letter height represents AA frequencies in dataset 4. “O” represents an Asn in a PNGS motif. AAs associated with resistance and sensitivity are red and blue, respectively. AAs shown in green differ for different bNAbs within the class. (A) V3 bNAbs, (B) V2, (C) VH1-2 and VH1-46 CD4bs, and (D) MPER, with 10E8/4E10/DH511 on the left, 2F5 on the right, and red HXB2 position numbers highlighting opposing signatures between the two.

sensitivity depending on the bNAb class (Figure 3; Table S3), including the PNGS at N130, associated with V2 bNAb resistance and CD4bs VRC03 sensitivity; the PNGS at N332, associated with V3 bNAb sensitivity and V2 bNAb resistance; and L165, associated with V2 bNAb sensitivity and V3 bNAb resistance (Figure 3; Table S3). Antibodies within a class can also have contradictory signatures. Some MPER signature AAs have opposing associations for 2F5 versus 4E10/10E8/DH511 (Figures 3 and S3; Table S3). A negatively charged D279 is associated with VRC01 sensitivity and with 12A12 resistance, likely due to the local charge in the bNAb paratopes (Figure S3) (Klein et al., 2013). CD4bs signatures for CDRH3 bNAbs (Zhou et al., 2015) often had opposing signatures relative to VH1-2 or VH1-46 bNAbs (Table S3).

Clade Sensitivity

Signature sites offer hypotheses to explain bNAb clade sensitivities. Four contact site candidates were proposed to contribute to the reduced reactivity of CAP256.VRC26 bNAbs with B clade viruses (Doria-Rose et al., 2016). We found an additional 17 signatures that may limit V2 bNAb potency against B clade viruses (Figure S4A). In contrast, CAP256.VRC26 bNAbs are most potent against clade C viruses and 12 signatures may be relevant. CD4bs antibodies have enhanced potency against A clade viruses, and resistance signatures are relatively rare in A clade (Figure S4B); in contrast, 3BNC117 and VRC01 have reduced breadth and potency against C clade viruses (Figure 2).

Hypervariable Loops and Neutralization Sensitivity

HIV-1 Env hypervariable regions evolve rapidly *in vivo* by insertions and deletions, giving rise to extreme length variation and making alignments spanning these regions unreliable. These changes can mediate bNAb escape (Bonsignori et al., 2016; Gao et al., 2014; Korber et al., 2017). We use alignment-independent characteristics of these regions (length, net charge, and

Figure 2. Env Characteristics Associated with bNAb Class Sensitivity

(A) Clade associations. Circles illustrate IC_{50} titers from dataset 4, highlighting the 3 best represented clades: A in red, B in green, and C and CRF07 (which is clade C in Env) in blue. All others are gray. Boxplots show medians and quartiles. Patterns of relative clade sensitivity are consistent across bNAb classes. The p values are based on two-sided Wilcoxon tests comparing the most distinctive clade among A, B, and C to the other two clades. Points above the horizontal line were above the threshold of detection. The bolus of negative points in the “other” group for V3 bNAbs is primarily CRF01.

(B) Examples of hypervariable loop characteristic correlations with bNAb sensitivity, including one for each bNAb class (complete associations are in Tables S3N–S3Q). M-group and clade C data are from datasets 4 and 3, respectively. The p values are based on Kendall’s tau.

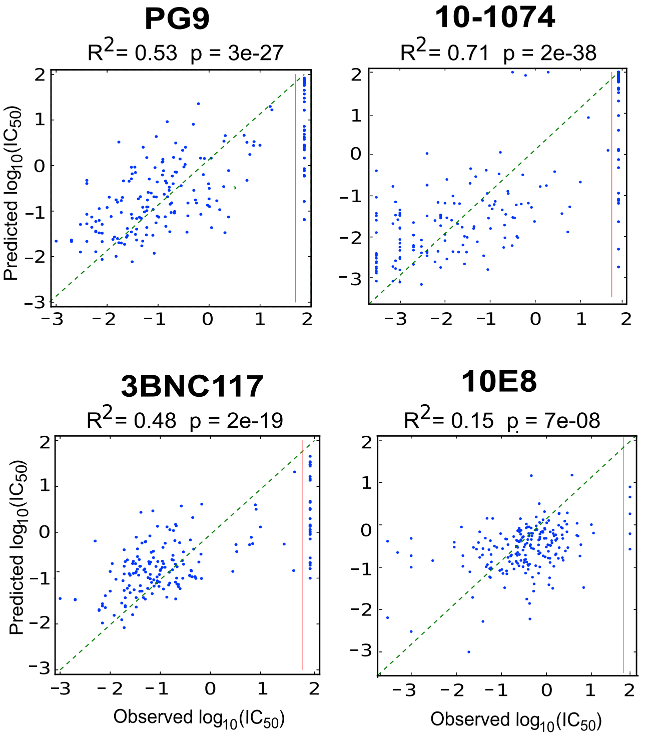

Figure 4. Random Forest Signature-Based Prediction Accuracy
Leave-one-out cross-validation regression predictions using dataset 4 for one bNAb from each bNAb class. R^2 is the standard coefficient of determination; the p value is from Kendall's tau. The red line marks the threshold of detection. R^2 values remained significant when negative points were excluded: PG9, $R^2 = 0.35$, $p = 4 \times 10^{-14}$; 10–1074, $R^2 = 0.27$, $p = 2 \times 10^{-9}$; 3BNC117, $R^2 = 0.30$, $p = 3 \times 10^{-9}$; and 10E8, $R^2 = 0.12$, $p = 9 \times 10^{-7}$.

number of PNGSs) to identify patterns associated with bNAb resistance profiles. The strongest of these associations are included in Tables S3M–S3P, and examples are shown in Figure 2B.

Combined V1+V2 length was the strongest correlate with V3 bNAb neutralization resistance—so insertions in either or both regions reduce sensitivity—followed by the number of PNGSs in V1+V2 and V1 length (Figure 2B; Table S3). V1 length variation played a critical role in the development of V3 bNAb DH270 lineage (Bonsignori et al., 2017a). A glycan in hypervariable V1 contacts PGT121-family bNAbs, enabling interactions when the key N332 glycan is absent. However, when N332 glycan is present, consistent with our signature predictions, removing this glycan enhances PGT121-family sensitivity (Garces et al., 2015).

The strongest variable region correlation with V2 bNAb sensitivity was V2 loop net-positive charge (Figure 2B; Table S6). The effect remained strong when only the V2 hypervariable region (positions 185–190) was considered. V2 bNAbs have long anionic CDRH3 loops (Doria-Rose et al., 2012; McLellan et al., 2011), which may drive the preference for positive charge. Also, V1+V2 hypervariable region length was inversely correlated with V2 bNAb sensitivity (Table S3).

Paradoxically, although CD4bs bNAbs can have great breadth, they bind near the V5 region which is subject to extreme length variation. Both V5 length and the number of PNGSs within V5 are associated with reduced CD4bs bNAb potency (Figure 2B; Table S3). CD4bs bNAbs can be selected to tolerate changes in V5 length after they arise as escape mutations *in vivo* (Fera et al., 2014). Long V1 and V2 regions also correlate with CD4bs bNAb resistance (Table S3) and mediate *in vivo* escape (Gao et al., 2014). Hence, long V1 and V2 regions are associated with relative resistance to 3 major classes of bNAbs: CD4bs, V2, and V3. At the population level, there are evolutionary counter-pressures against larger loops, as smaller hypervariable regions tend to be selected at transmission (Derdeyn et al., 2004). Unexpectedly, increasing numbers of PNGSs in V1 correlated with enhanced sensitivity to MPER bNAbs, particularly 10E8 (Figure 2B; Table S3).

Machine Learning Predictions of Env Neutralization

We next explored using bNAb signatures for sequence-based machine learning predictions of bNAb sensitivity. Using a Random Forest (RF) for IC_{50} regression predictions, we compared prediction accuracies for 3 prefiltering strategies: (1) a standard prefilter, minimal-redundancy-maximal-relevance (mRMR) (Peng et al., 2005); (2) the full bNAb signatures for each antibody class, including AA, PNGS, clade, and variable loop characteristics; and (3) only signatures in contact sites. We evaluated predictions using leave-one-out cross validation for dataset 4, as well as for an independent C clade holdout set. The accuracy using the full signature was superior to mRMR (p = 0.003, paired Wilcoxon, C clade holdout comparison), and to the contact-region-only signature (also p = 0.003) (Table S4); examples are shown in Figure 4. We also tested positive/negative classification predictions using our prefilter strategies and RF, comparing them to a published method called IDEpi (Hepler et al., 2014); the methods were comparable (Table S5). Hypervariable region characteristics were consistently among the most important factors for predicting IC_{50} titers (Table S6).

V2-SET Vaccines

Immunogen Design and Expression

We next utilized V2 bNAb signatures to inform vaccine design, intending to increase V2 epitope exposure and represent relevant natural diversity within the epitope. V2-SET trivalent vaccines included 459C WT, which alone induced low-level neutralization of some tier 2 viruses plus two complementary immunogens, called Optimal (Opt) and Alternative (Alt) (Figures 5A and 5B; Table S7). Opt introduced V2 bNAb virus sensitivity signatures into the 459C WT backbone, to enhance epitope expression, exposure, affinity, or relevant carbohydrate processing (Figure 5A). Alt incorporated V2 bNAb sensitivity signatures outside of the contact region; however, within the epitope it captured natural diversity in V2 signature sites—including globally common AAs complementary to those found in 459C WT and Opt—even if associated with relative resistance (Figure 5B). V2-SET immunogens also incorporated modified hypervariable regions with characteristics favoring V2 bNAb sensitivity, including short V1 and V2 hypervariable regions with a positively charged V2 region (Figure 2B; Tables S3N–S3R). The WT Env T250_4 met these criteria and was also highly sensitive to *both* V2 and V3 bNAbs (Figure S1). Hypothesizing T250_4 hypervariable regions might improve polyclonal

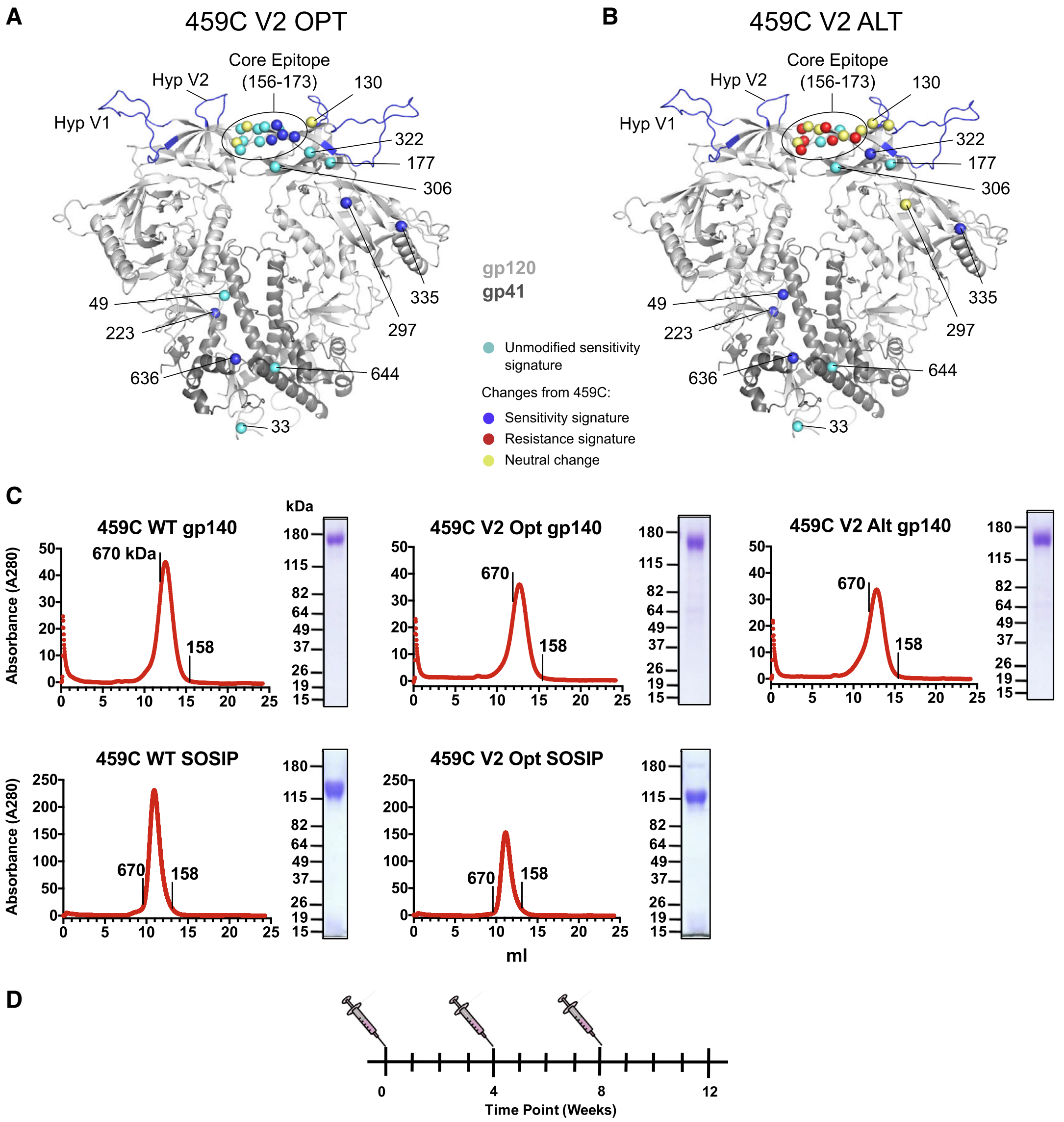

Vaccine	Prime	Boost 1	Boost 2	n
**459C WT**	459C WT gp140	459C WT gp140	459C WT gp140	15
**V2 Opt**	V2 Opt gp140	V2 Opt gp140	V2 Opt gp140	5
**V2 Alt**	V2 Alt gp140	V2 Alt gp140	V2 Alt gp140	5
**V2 Opt+V2 Alt**	V2 Opt+V2 Alt gp140	V2 Opt+V2 Alt gp140	V2 Opt+V2 Alt gp140	5
**V2 Mixture**	459C WT+V2 Opt+V2 Alt gp140	459C WT+V2 Opt+V2 Alt gp140	459C WT+V2 Opt+V2 Alt gp140	5
**V2 Prime/Boost**	V2 Opt gp140	WT+V2 Alt gp140	WT+V2 Alt gp140	5
**459C WT SOSIP**	459C WT SOSIP	459C WT SOSIP	459C WT SOSIP	5
**V2 Mixture SOSIP**	459C WT+V2 Opt SOSIP+V2 Alt gp140	459C WT+V2 Opt SOSIP+V2 Alt gp140	459C WT+V2 Opt SOSIP+V2 Alt gp140	5
**ABCM Mixture**	92UG+PVO.4+C97+Mos gp140	92UG+PVO.4+C97+Mos gp140	92UG+PVO.4+C97+Mos gp140	5
**3C Mixture**	459C WT+C97+405C gp140	459C WT+C97+405C gp140	459C WT+C97+405C gp140	5

Figure 5. V2-SET Vaccine Design and Production

(A and B) Structural mapping (PDB: 5FYJ) of mutations introduced into 459C WT (Table S7) to create (A) Opt and (B) Alt V2-SET vaccine constructs. Spheres are color-coded to indicate AA modifications associated with sensitivity or resistance. Opt constructs uniformly carry sensitivity signatures. Alt constructs carry mutations that enhance sensitivity outside the core epitope, but in the epitope introduce signature mutations complementary to 459C WT and Opt to capture epitope diversity.

(C) Gel filtration chromatography traces of gp140 V2-SET immunogens run on a Superose 6 column (foldon) or Superdex 200 column (SOSIP). Coomassie stained SDS-PAGE of purified Envs are next to each trace with molecular weight standards noted.

(D) Guinea pig vaccination regimens. Animals were vaccinated intramuscularly in the quadriceps with 100 μg total immunogen at weeks 0, 4, and 8; n is the group size.

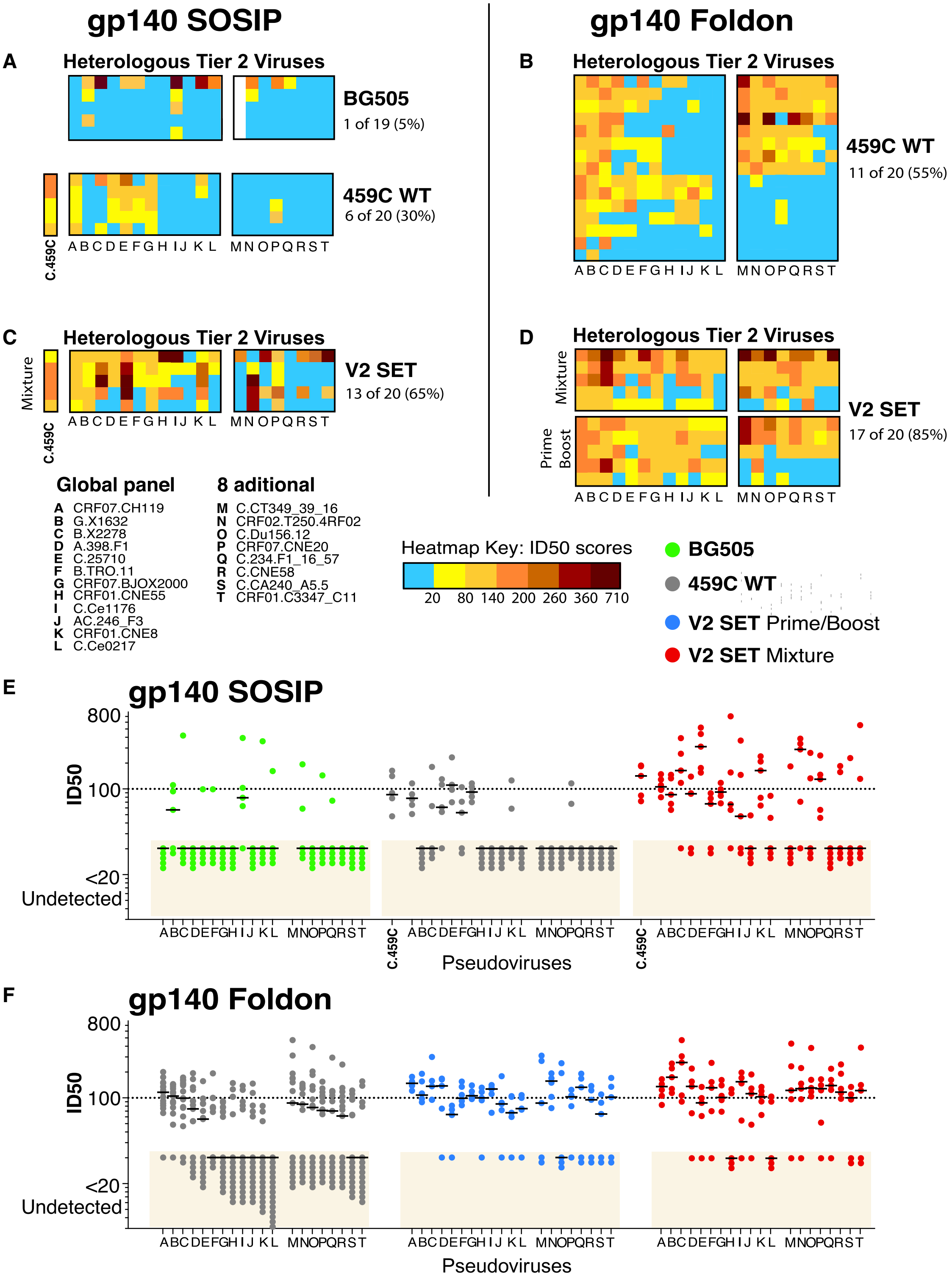

Figure 6. V2-SET Vaccines Improve the Breadth and the Magnitude of Tier 2 NAbs Compared to BG505 and 459C WT

(A–D) Heatmaps of neutralizing responses comparing groups of guinea pigs vaccinated with 459C WT, V2-SET, and BG505 vaccines. Monovalent vaccines: (A) BG505 and 459C WT SOSIP and (B) 459C WT gp140 foldon. Trivalent vaccines: (C) V2-SET SOSIP and (D) gp140 foldon vaccines delivered as either a mixture or prime boost. Columns represent tier 2 pseudoviruses (see key), ordered by sensitivity. Rows represent guinea pigs, organized by vaccine group. The potency of ID_{50} responses increases from yellow to dark red, below threshold responses are blue. To compare the breadth of response between different vaccine regimens, the median number of detectable responses is reported for each vaccination regimen to the right of the heatmaps, and detectible responses per animal were

(legend continued on next page)

responses to both bNAb classes, we used T250_4's V1 and V2 regions in our V2-SET constructs (Table S7).

The V2-SET gp140s were produced in 293T cells by transient transfection as either SOSIP or foldon immunogens. The gp140 foldon contains a C-terminal T4-fibritin trimerization domain following the MPER and is not cleaved by furin (Frey et al., 2008). The gp140 SOSIP is a native-like trimer truncated before the MPER and cleaved by furin (Steichen et al., 2016). Each purified Env protein ran as a single symmetrical peak by size exclusion chromatography, and as a single band on SDS-PAGE (Figure 5C). We could not produce a stable V2 Alt SOSIP.

The antigenic properties of the V2-SET foldon immunogens were probed using surface plasmon resonance (Tables S5A–S5C). Soluble CD4 (Kwong et al., 1998) and V3 bNAb 10–1074 both bound to all three. V2 bNAb PG16 bound Opt and Alt immunogens more robustly than 459C WT, consistent with increased V2 exposure. Trimer-specific V2 bNAbs PGT145 and PGDM1400 bound robustly to SOSIP but not to foldon gp140s, consistent with SOSIP gp140s being native-like (Sanders et al., 2013; Steichen et al., 2016).

Immunogenicity of V2-SET Env Vaccines

Guinea pigs were immunized three times at monthly intervals (Figure 5D), intramuscularly in the quadriceps, with a total of 100 μg Env (divided equally among immunogens in cocktails) formulated with CpG/Emulsigen adjuvant. Animals were bled 4 weeks after each vaccination, with peak immunogenicity at week 12. Binding responses were assessed by ELISA using the immunogens and a small panel of additional Envs expressed as gp140s, and V1/V2 gp70 scaffolds (Kayman et al., 1994) (Figures S5D–S5E). All vaccination regimens elicited comparable high magnitude binding responses with similar kinetics. Furthermore, all vaccines elicited tier-1 NAb responses against easy-to-neutralize viruses (Figure S5F).

Trivalent V2-SET vaccines augmented the magnitude and breadth of neutralization against 20 tier 2 pseudoviruses (the standard global panel of 12 [deCamp et al., 2014], plus 8 additional) compared with 459C WT alone, using either the gp140 SOSIP or gp140 foldon vaccine platforms (Figure 6). Using the gp140 foldon platform, the V2-SET Mixture (the 3 vaccine components co-delivered) and V2-SET Prime/Boost (V2 Opt prime and V2 Alt+459C WT boost) both improved the magnitude of tier 2 NAbs responses compared to 459C WT alone ($p = 0.006$ and $p = 0.008$, respectively, using a non-parametric permutation test). A median of 85% of the heterologous tier 2 viruses were neutralized in the V2-SET groups, compared to 55% in the 459C WT alone group ($p = 0.004$, two-sided Wilcoxon rank sum) (Figure 6B). Using the SOSIP platform the V2-SET Mixture similarly improved neutralization potency compared to SOSIP 459C WT alone ($p = 0.008$), and a median of 65% of the heterologous tier 2 viruses tested were neutralized in the V2-SET group, compared to 30% for the 459C WT group ($p = 0.008$) (Figure 6A). In contrast, the BG505 SOSIP gp140 induced a median of 5% the heterologous tier 2 viruses tested, although one animal had more breadth (Figure 6C).

We next explored the generalizability these findings. First, tier 2 NAb responses with the V2-SET vaccine were similarly enhanced compared to 459C WT using a different adjuvant, MPLA (Nkolola et al., 2014b) (Figures S6A and S6B). Second, while V2 Opt and Alt delivered alone were immunogenic, they did not enhance neutralization breadth like the trivalent combination (Figure S6C). Third, the tier 2 NAb responses were mediated by purified IgG (Figure S7D). Finally, other previously studied multivalent Env cocktails involving natural sequence immunogens, a trivalent clade C vaccine (Bricault et al., 2015) and a tetravalent multiclade vaccine (Bricault et al., 2018), did not significantly enhance tier 2 breadth over 459C WT (Figure S6C). Together, these data suggest that the improved NAb breadth induced with the trivalent V2-SET vaccine was generalizable, dependent on the SET bioinformatic design, and could not be achieved by simple mixtures of WT Env immunogens.

We also assessed post-vaccination antibody responses for binding to linear peptides spanning Env (Stephenson et al., 2015). Despite similar overall ELISA titers (Figure S5), V2-SET vaccines binding responses to linear V3 peptides were markedly lower than 459C WT, suggesting they were redirected away from non-neutralizing linear V3 epitopes (Figure 7A). To assess whether the improved V2-SET tier 2 NAb responses resulted from V2-specific conformational antibodies, we constructed pseudoviruses that resulted in the loss of the PNGS at position N160—a critical sensitivity signature for V2 bNAbs. The N160-PNGS-loss mutation pseudoviruses showed increased neutralization sensitivity to 459C WT vaccine-elicited NAbs, suggesting that a region partly shielded by the N160 glycan was targeted, but they also abrogated the enhancement in NAb potency achieved with V2-SET vaccine compared to the 459C WT vaccine (Figure 7B), suggesting that the improved the performance of the V2-SET vaccine over 459C WT depended on the PNGS at N160.

DISCUSSION

We defined HIV-1 Env bNAb signatures using neutralization data from four large virus panels to provide an unprecedented level of bNAb signature mapping—including hundreds of AA and PNGS signatures, as well as critical hypervariable region characteristics—for over 50 NAbs. The accuracy of Env sequence-based machine learning predictions of IC_{50} titers were generally improved by focusing on relevant signatures and hypervariable

compared by a two-sided Wilcoxon test. BG505 and 459C WT SOSIP vaccines were comparable ($p = 0.13$), and V2-SET SOSIP vaccine responses were broader than either BG505 ($p = 0.02$) or 459C WT ($p = 0.01$). Responses elicited by the V2-SET foldon vaccine were broader than responses elicited by the 459C WT foldon vaccine ($p = 0.006$).

(E and F) Magnitudes of tier 2 NAb responses to SOSIP and foldon vaccine groups, respectively. Responses that were at least 10 above the background are considered positive and are shown; the dotted line at an ID_{50} titer of 100 is added for visual emphasis. Colors represent the vaccine groups (see key). Horizontal black lines are the median response to each pseudovirus. Response magnitudes were compared with a nonparametric permutation test (STAR Methods). For SOSIP vaccines, there was no statistical difference in potency between BG505 and 459C WT vaccine groups ($p = 0.06$), but V2-SET responses were more potent than both BG505 ($p = 0.007$) and 459C WT ($p = 0.008$). For gp140 foldon delivery, V2-SET responses were more potent than 459C WT ($p = 0.002$).

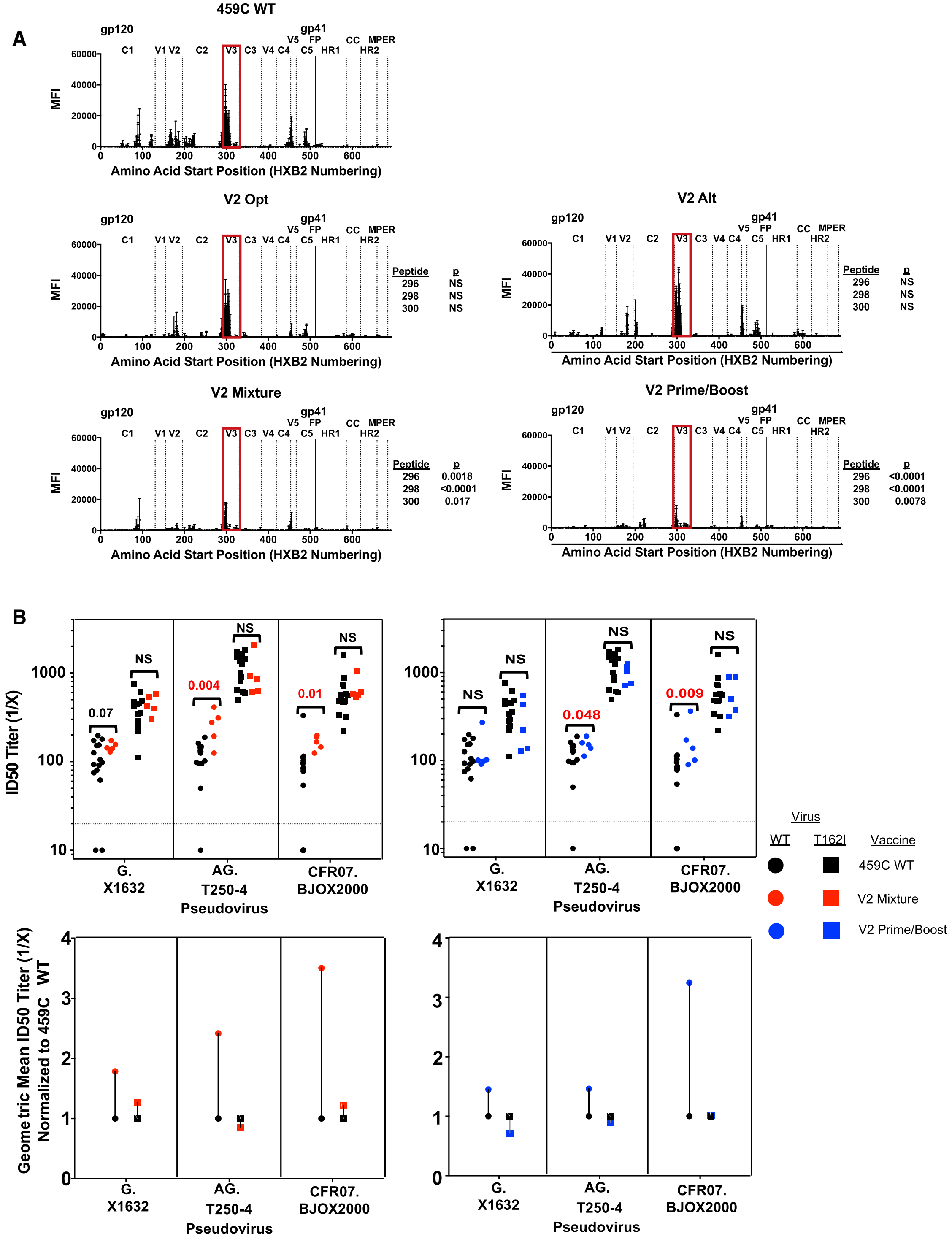

Figure 7. Mapping of Antibody Responses Elicited by V2-SET Vaccine

(A) Magnitude and position of binding antibody responses from guinea pig sera to linear 15-mer peptides on peptide microarrays from each gp140 foldon vaccine group. Each dot represents an average MFI (mean fluorescence intensity) per peptide that is positive for antibody binding within each vaccination group, standard deviation shown. Env regions are delineated by vertical lines, the V3 loop highlighted in red. Statistical differences for binding responses to peptides with starting positions in V3 as compared to 459C WT are shown. The p values are based on a Wilcoxon one-sided test; NS means not significant.

(B) Neutralizing titers against select pseudoviruses with a N160-dependent enhancement of V2-SET responses over 459C WT. ID_{50} titers in guinea pigs vaccinated with 459C WT and V2-SET vaccines against the native pseudoviruses are shown by dots, and against the N160 glycan deletion mutant (T162I)

(legend continued on next page)

region characteristics that were consistently highly ranked as key features for accurate predictions; thus supporting the consideration of these characteristics in vaccine design. Such machine-learning-based predictions of bNAb sensitivity levels across populations of Env sequences may ultimately be useful for modeling the relative sensitivity of a set of bNAbs across a regional epidemic targeted for treatment and for interpreting results of bNAb-based prevention and therapeutic clinical studies. Our data also highlight the importance of HIV-1 clades for both bNAb passive infusion studies and vaccine studies.

We also developed a signature-based approach to Env immunogen design using the V2 bNAb signature patterns to inform the design of a trivalent V2-SET vaccine. Induction of heterologous tier 2 NAbs has proven to be a major challenge for the HIV-1 vaccine field, and to date, the breadth of tier 2 NAbs induced by vaccines has been modest in both small and large animal models. The native-like BG505 SOSIP trimer induces potent autologous NAbs but with minimal tier 2 NAb breadth (Pauthner et al., 2017; Sanders et al., 2015). The trivalent V2-SET vaccine induced greater breadth of tier 2 NAb responses than the 459C SOSIP trimer alone, and both proved superior to the BG505 SOSIP trimer. The improved NAb breadth using V2-SET antigens was reproducible in guinea pig vaccination studies and generalizable using two common HIV-1 Env trimer platforms (SOSIP and foldon gp140s) and two adjuvants (CpG/Emulsigen and MPLA). Moreover, the enhanced NAb breadth was not observed with two cocktails of natural sequence HIV-1 Env immunogens. Although the magnitude of the tier 2 NAb responses remained low to moderate, these data demonstrate the proof-of-concept that bNAb signatures can contribute to the design of next-generation HIV-1 Env immunogens.

STAR★METHODS

Detailed methods are provided in the online version of this paper and include the following:

- KEY RESOURCES TABLE
- CONTACT FOR REAGENT AND RESOURCE SHARING
- EXPERIMENTAL MODEL AND SUBJECT DETAILS
  - Human Subjects
  - Cell Lines
  - Guinea Pig Vaccinations
- METHOD DETAILS
  - Experimental Methods for Vaccine Evaluation
  - Sequence and Signature Analysis
- QUANTIFICATION AND STATISTICAL ANALYSIS
  - Sequence Analyses
  - Vaccine Immune Response Comparisons
- DATA AND SOFTWARE AVAILABILITY
  - GenSig
  - CATNAP Enhancement
  - FilteredForests
  - bNAb signature information access

SUPPLEMENTAL INFORMATION

Supplemental Information includes seven figures and seven tables and can be found with this article online at https://doi.org/10.1016/j.chom.2018.12.001.

ACKNOWLEDGMENTS

Supported by the Bill & Melinda Gates Foundation (OPP1032144, OPP1040741, OPP1032144, and OPP1169339), the NIH (AI096040, AI100645, A1095985, AI124377, AI126603, AI128751, and AI129797), and the Ragon Institute of MGH, MIT, and Harvard. We have no financial conflicts of interest. We thank Robert Bailer and Mark Louder, VRC, NIAID, for contributing large panel neutralization data. We thank Mark Connors, Lynn Morris, Penny Moore, Michel Nussenzweig, Michael Farzan, and Matthew Gardner for availability of antibodies and reagents; the HVTN Laboratory Program and Leonidas Stamatatos for envelope sequences; Nicholas Provine, Zi Han Kang, Alex Badamchi-Zadeh, Pablo Penaloza-MacMaster, Rafael Larocca, Sophia Rits-Vollach, Sandra Vertentes, and Helen DeCosta for generous assistance and advice; and Kelli Green and Hongmei Gao for their organizational efforts.

AUTHOR CONTRIBUTIONS

Conceptualization, B.K., C.A.B., D.H.B., and D.C.M.; Software, H.Y., J.T., B.K., M.T., K.W., and P.H.; Writing, C.A.B., K.Y., B.K., and D.H.B.; Review & Editing, J.T., G.H.L., B.H.H., K.W., J.P.M., E.F.K., M.S.S., D.C.M., B.F.H., and J.R.M.; Analysis, B.K., J.T., K.Y., E.E.G., K.W., M.T., P.H., C.A.B., D.H.B., and M.S.S.; Supervision, D.C.M., B.K., B.H.H., S.G., D.H.B., M.S.S., B.C., C.O., and K.E.S.; Bioinformatic Methodology, B.K., D.C.M., K.Y., and E.F.K.; Data Curation, J.P.M., K.Y., and G.H.L.; Immunological and Biochemical Assays, C.A.B., M.S.S., J.M.K., J.L.S., C.L.L., F.G., M.R., M.G.B., G.H.N., K.M., J.J.J., J.Z., C.O., H.P., C.C., J.P.N., K.E.S., and L.D.W.; Visualization, B.K., K.W., K.Y., and J.T.; Resources, N.D.-R., J.R.M., M.S.S., D.C.M., J.F.S., M.B., L.D.W., and B.F.H.; Funding Acquisition, D.C.M., D.H.B., B.F.H., and B.K.

DECLARATION OF INTERESTS

The authors declare no competing interests. A provisional patent application for the vaccine technology has been filed by C.A.B., K.Y., D.H.B., and B.K.

Received: April 14, 2018
Revised: July 6, 2018
Accepted: November 14, 2018
Published: January 9, 2019

REFERENCES

Arlot, S., and Celisse, A. (2010). A survey of cross-validation procedures for model selection. Statist. Surv. *4*, 40–79.

Behrens, A.J., Vasiljevic, S., Pritchard, L.K., Harvey, D.J., Andev, R.S., Krumm, S.A., Struwe, W.B., Cupo, A., Kumar, A., Zitzmann, N., et al. (2016). Composition and antigenic effects of individual glycan sites of a trimeric HIV-1 envelope glycoprotein. Cell Rep. *14*, 2695–2706.

Bhattacharya, T., Daniels, M., Heckerman, D., Foley, B., Frahm, N., Kadie, C., Carlson, J., Yusim, K., McMahon, B., Gaschen, B., et al. (2007). Founder effects in the assessment of HIV polymorphisms and HLA allele associations. Science *315*, 1583–1586.

Bonsignori, M., Hwang, K.K., Chen, X., Tsao, C.Y., Morris, L., Gray, E., Marshall, D.J., Crump, J.A., Kapiga, S.H., Sam, N.E., et al. (2011). Analysis of a clonal lineage of HIV-1 envelope V2/V3 conformational epitope-specific

pseudoviruses by squares. Colors represent the particular gp140 foldon vaccination regimen: black is 459C WT, red is V2-SET mixture, and blue is V2-SET prime/boost. The top plots show ID_{50} titers for each guinea pig. The dotted line at 20 marks the limit of detection. The bottom plots show the geometric means of the NAb titers from the top plot (over the animals vaccinated by the same vaccine and tested on the same pseudovirus as in the top plot), normalized to 459C WT. The p values from Wilcoxon pairwise comparisons are shown in red. NS, not significant.

broadly neutralizing antibodies and their inferred unmutated common ancestors. J. Virol. *85*, 9998–10009.

Bonsignori, M., Kreider, E.F., Fera, D., Meyerhoff, R.R., Bradley, T., Wiehe, K., Alam, S.M., Aussedat, B., Walkowicz, W.E., Hwang, K.K., et al. (2017a). Staged induction of HIV-1 glycan-dependent broadly neutralizing antibodies. Sci. Transl. Med. *9*, https://doi.org/10.1126/scitranslmed.aai7514.

Bonsignori, M., Liao, H.X., Gao, F., Williams, W.B., Alam, S.M., Montefiori, D.C., and Haynes, B.F. (2017b). Antibody-virus co-evolution in HIV infection: paths for HIV vaccine development. Immunol. Rev. *275*, 145–160.

Bonsignori, M., Zhou, T., Sheng, Z., Chen, L., Gao, F., Joyce, M.G., Ozorowski, G., Chuang, G.Y., Schramm, C.A., Wiehe, K., et al. (2016). Maturation pathway from germline to broad HIV-1 neutralizer of a CD4-mimic antibody. Cell *165*, 449–463.

Bradley, T., Trama, A., Tumba, N., Gray, E., Lu, X., Madani, N., Jahanbakhsh, F., Eaton, A., Xia, S.M., Parks, R., et al. (2016). Amino acid changes in the HIV-1 gp41 membrane proximal region control virus neutralization sensitivity. EBioMedicine *12*, 196–207.

Breiman, L. (2001). Random forests. Mach. Learn. *45*, 5–32.

Bricault, C.A., Kovacs, J.M., Badamchi-Zadeh, A., McKee, K., Shields, J.L., Gunn, B.M., Neubauer, G.H., Ghantous, F., Jennings, J., Gillis, L., et al. (2018). Neutralizing antibody responses following long-term vaccination with HIV-1 Env gp140 in guinea pigs. J. Virol. *92*, https://doi.org/10.1128/JVI.00369-18.

Bricault, C.A., Kovacs, J.M., Nkolola, J.P., Yusim, K., Giorgi, E.E., Shields, J.L., Perry, J., Lavine, C.L., Cheung, A., Ellingson-Strouss, K., et al. (2015). A multivalent clade C HIV-1 Env trimer cocktail elicits a higher magnitude of neutralizing antibodies than any individual component. J. Virol. *89*, 2507–2519.

Buchacher, A., Predl, R., Strutzenberger, K., Steinfellner, W., Trkola, A., Purtscher, M., Gruber, G., Tauer, C., Steindl, F., Jungbauer, A., et al. (1994). Generation of human monoclonal antibodies against HIV-1 proteins; electrofusion and Epstein-Barr virus transformation for peripheral blood lymphocyte immortalization. AIDS Res. Hum. Retrovir. *10*, 359–369.

Burton, D.R., Barbas, C.F., 3rd, Persson, M.A., Koenig, S., Chanock, R.M., and Lerner, R.A. (1991). A large array of human monoclonal antibodies to type 1 human immunodeficiency virus from combinatorial libraries of asymptomatic seropositive individuals. Proc. Natl. Acad. Sci. USA *88*, 10134–10137.

Burton, D.R., and Hangartner, L. (2016). Broadly neutralizing antibodies to HIV and their role in vaccine design. Annu. Rev. Immunol. *34*, 635–659.

Burton, D.R., and Mascola, J.R. (2015). Antibody responses to envelope glycoproteins in HIV-1 infection. Nat. Immunol. *16*, 571–576.

Cardoso, R.M., Zwick, M.B., Stanfield, R.L., Kunert, R., Binley, J.M., Katinger, H., Burton, D.R., and Wilson, I.A. (2005). Broadly neutralizing anti-HIV antibody 4E10 recognizes a helical conformation of a highly conserved fusion-associated motif in gp41. Immunity *22*, 163–173.

Chuang, G.Y., Acharya, P., Schmidt, S.D., Yang, Y., Louder, M.K., Zhou, T., Kwon, Y.D., Pancera, M., Bailer, R.T., Doria-Rose, N.A., et al. (2013). Residue-level prediction of HIV-1 antibody epitopes based on neutralization of diverse viral strains. J. Virol. *87*, 10047–10058.

Chuang, G.Y., Liou, D., Kwong, P.D., and Georgiev, I.S. (2014). NEP: web server for epitope prediction based on antibody neutralization of viral strains with diverse sequences. Nucleic Acids Res. *42*, W64–W71.

Corti, D., Langedijk, J.P., Hinz, A., Seaman, M.S., Vanzetta, F., Fernandez-Rodriguez, B.M., Silacci, C., Pinna, D., Jarrossay, D., Balla-Jhagjhoorsingh, S., et al. (2010). Analysis of memory B cell responses and isolation of novel monoclonal antibodies with neutralizing breadth from HIV-1-infected individuals. PLoS One *5*, e8805.

Crispin, M., and Doores, K.J. (2015). Targeting host-derived glycans on enveloped viruses for antibody-based vaccine design. Curr. Opin. Virol. *11*, 63–69.

Crooks, G.E., Hon, G., Chandonia, J.M., and Brenner, S.E. (2004). WebLogo: a sequence logo generator. Genome Res. *14*, 1188–1190.

Dabney, A., and Storey, J.D. (2013). Q-value estimation for false discovery rate control. R package version 2.14.0. http://github.com/jdstorey/qvalue.

deCamp, A., Hraber, P., Bailer, R.T., Seaman, M.S., Ochsenbauer, C., Kappes, J., Gottardo, R., Edlefsen, P., Self, S., Tang, H., et al. (2014). Global panel of HIV-1 Env reference strains for standardized assessments of vaccine-elicited neutralizing antibodies. J. Virol. *88*, 2489–2507.

Derdeyn, C.A., Decker, J.M., Bibollet-Ruche, F., Mokili, J.L., Muldoon, M., Denham, S.A., Heil, M.L., Kasolo, F., Musonda, R., and Hahn, B.H. (2004). Envelope-constrained neutralization-sensitive HIV-1 after heterosexual transmission. Science *303*, 2019–2022.

Diskin, R., Scheid, J.F., Marcovecchio, P.M., West, A.P., Jr., Klein, F., Gao, H., Gnanapragasam, P.N., Abadir, A., Seaman, M.S., Nussenzweig, M.C., et al. (2011). Increasing the potency and breadth of an HIV antibody by using structure-based rational design. Science *334*, 1289–1293.

Doria-Rose, N.A., Bhiman, J.N., Roark, R.S., Schramm, C.A., Gorman, J., Chuang, G.Y., Pancera, M., Cale, E.M., Ernandes, M.J., Louder, M.K., et al. (2016). New member of the V1V2-directed CAP256-VRC26 lineage that shows increased breadth and exceptional potency. J. Virol. *90*, 76–91.

Doria-Rose, N.A., Georgiev, I., O'Dell, S., Chuang, G.Y., Staupe, R.P., McLellan, J.S., Gorman, J., Pancera, M., Bonsignori, M., Haynes, B.F., et al. (2012). A short segment of the HIV-1 gp120 V1/V2 region is a major determinant of resistance to V1/V2 neutralizing antibodies. J. Virol. *86*, 8319–8323.

Doria-Rose, N.A., Schramm, C.A., Gorman, J., Moore, P.L., Bhiman, J.N., DeKosky, B.J., Ernandes, M.J., Georgiev, I.S., Kim, H.J., Pancera, M., et al. (2014). Developmental pathway for potent V1V2-directed HIV-neutralizing antibodies. Nature *509*, 55–62.

Escolano, A., Steichen, J.M., Dosenovic, P., Kulp, D.W., Golijanin, J., Sok, D., Freund, N.T., Gitlin, A.D., Oliveira, T., Araki, T., et al. (2016). Sequential immunization elicits broadly neutralizing anti-HIV-1 antibodies in Ig knockin mice. Cell *166*, 1445–1458.e12.

Evans, M.C., Phung, P., Paquet, A.C., Parikh, A., Petropoulos, C.J., Wrin, T., and Haddad, M. (2014). Predicting HIV-1 broadly neutralizing antibody epitope networks using neutralization titers and a novel computational method. BMC Bioinformatics *15*, 77.

Fera, D., Schmidt, A.G., Haynes, B.F., Gao, F., Liao, H.X., Kepler, T.B., and Harrison, S.C. (2014). Affinity maturation in an HIV broadly neutralizing B-cell lineage through reorientation of variable domains. Proc. Natl. Acad Sci. USA *111*, 10275–10280.

Ferguson, A.L., Falkowska, E., Walker, L.M., Seaman, M.S., Burton, D.R., and Chakraborty, A.K. (2013). Computational prediction of broadly neutralizing HIV-1 antibody epitopes from neutralization activity data. PLoS One *8*, e80562.

Freeman, M.M., Seaman, M.S., Rits-Volloch, S., Hong, X., Kao, C.Y., Ho, D.D., and Chen, B. (2010). Crystal structure of HIV-1 primary receptor CD4 in complex with a potent antiviral antibody. Structure *18*, 1632–1641.

Frey, G., Peng, H., Rits-Volloch, S., Morelli, M., Cheng, Y., and Chen, B. (2008). A fusion-intermediate state of HIV-1 gp41 targeted by broadly neutralizing antibodies. Proc. Natl. Acad. Sci. USA *105*, 3739–3744.

Gao, F., Bonsignori, M., Liao, H.X., Kumar, A., Xia, S.M., Lu, X., Cai, F., Hwang, K.K., Song, H., Zhou, T., et al. (2014). Cooperation of B cell lineages in induction of HIV-1-broadly neutralizing antibodies. Cell *158*, 481–491.

Garces, F., Lee, J.H., de Val, N., Torrents de la Pena, A.T., Kong, L., Puchades, C., Hua, Y., Stanfield, R.L., Burton, D.R., Moore, J.P., et al. (2015). Affinity maturation of a potent family of HIV antibodies is primarily focused on accommodating or avoiding glycans. Immunity *43*, 1053–1063.

Geurts, P., Ernst, D., and Wehenkel, L. (2006). Extremely randomized trees. Mach. Learn. *63*, 3–42.

Gnanakaran, S., Daniels, M.G., Bhattacharya, T., Lapedes, A.S., Sethi, A., Li, M., Tang, H., Greene, K., Gao, H., Haynes, B.F., et al. (2010). Genetic signatures in the envelope glycoproteins of HIV-1 that associate with broadly neutralizing antibodies. PLoS Comp. Biol. *6*, e1000955.

Gorman, J., Soto, C., Yang, M.M., Davenport, T.M., Guttman, M., Bailer, R.T., Chambers, M., Chuang, G.Y., DeKosky, B.J., Doria-Rose, N.A., et al. (2016). Structures of HIV-1 Env V1V2 with broadly neutralizing antibodies reveal commonalities that enable vaccine design. Nat. Struct. Mol. Biol. *23*, 81–90.

Guindon, S., Dufayard, J.F., Lefort, V., Anisimova, M., Hordijk, W., and Gascuel, O. (2010). New algorithms and methods to estimate maximum-likelihood phylogenies: assessing the performance of PhyML 3.0. Syst. Biol. *59*, 307–321.

Hepler, N.L., Scheffler, K., Weaver, S., Murrell, B., Richman, D.D., Burton, D.R., Poignard, P., Smith, D.M., and Kosakovsky Pond, S.L. (2014). IDEPI: rapid prediction of HIV-1 antibody epitopes and other phenotypic features from sequence data using a flexible machine learning platform. PLoS Comput. Biol. *10*, e1003842.

Hraber, P., Korber, B.T., Lapedes, A.S., Bailer, R.T., Seaman, M.S., Gao, H., Greene, K.M., McCutchan, F., Williamson, C., Kim, J.H., et al. (2014a). Impact of clade, geography, and age of the epidemic on HIV-1 neutralization by antibodies. J. Virol. *88*, 12623–12643.

Hraber, P., Seaman, M.S., Bailer, R.T., Mascola, J.R., Montefiori, D.C., and Korber, B.T. (2014b). Prevalence of broadly neutralizing antibody responses during chronic HIV-1 infection. AIDS *28*, 163–169.

Huang, J., Kang, B.H., Ishida, E., Zhou, T., Griesman, T., Sheng, Z., Wu, F., Doria-Rose, N.A., Zhang, B., McKee, K., et al. (2016). Identification of a CD4-Binding-Site antibody to HIV that evolved near-pan neutralization breadth. Immunity *45*, 1108–1121.

Huang, J., Ofek, G., Laub, L., Louder, M.K., Doria-Rose, N.A., Longo, N.S., Imamichi, H., Bailer, R.T., Chakrabarti, B., Sharma, S.K., et al. (2012). Broad and potent neutralization of HIV-1 by a gp41-specific human antibody. Nature *491*, 406–412.

Julien, J.P., Sok, D., Khayat, R., Lee, J.H., Doores, K.J., Walker, L.M., Ramos, A., Diwanji, D.C., Pejchal, R., Cupo, A., et al. (2013). Broadly neutralizing antibody PGT121 allosterically modulates CD4 binding via recognition of the HIV-1 gp120 V3 base and multiple surrounding glycans. PLoS Pathog. *9*, e1003342.

Kayman, S.C., Wu, Z., Revesz, K., Chen, H., Kopelman, R., and Pinter, A. (1994). Presentation of native epitopes in the V1/V2 and V3 regions of human immunodeficiency virus type 1 gp120 by fusion glycoproteins containing isolated gp120 domains. J. Virol. *68*, 400–410.

Klein, F., Diskin, R., Scheid, J.F., Gaebler, C., Mouquet, H., Georgiev, I.S., Pancera, M., Zhou, T., Incesu, R.B., Fu, B.Z., et al. (2013). Somatic mutations of the immunoglobulin framework are generally required for broad and potent HIV-1 neutralization. Cell *153*, 126–138.

Klein, F., Gaebler, C., Mouquet, H., Sather, D.N., Lehmann, C., Scheid, J.F., Kraft, Z., Liu, Y., Pietzsch, J., Hurley, A., et al. (2012). Broad neutralization by a combination of antibodies recognizing the CD4 binding site and a new conformational epitope on the HIV-1 envelope protein. J. Exp. Med. *209*, 1469–1479.

Kong, L., Lee, J.H., Doores, K.J., Murin, C.D., Julien, J.P., McBride, R., Liu, Y., Marozsan, A., Cupo, A., Klasse, P.J., et al. (2013). Supersite of immune vulnerability on the glycosylated face of HIV-1 envelope glycoprotein gp120. Nat. Struct. Mol. Biol. *20*, 796–803.

Korber, B., Hraber, P., Wagh, K., and Hahn, B.H. (2017). Polyvalent vaccine approaches to combat HIV-1 diversity. Immunol. Rev. *275*, 230–244.

Korber, B., Muldoon, M., Theiler, J., Gao, F., Gupta, R., Lapedes, A., Hahn, B.H., Wolinsky, S., and Bhattacharya, T. (2000). Timing the ancestor of the HIV-1 pandemic strains. Science *288*, 1789–1796.

Korber, B.T., Letvin, N.L., and Haynes, B.F. (2009). T-cell vaccine strategies for human immunodeficiency virus, the virus with a thousand faces. J. Virol. *83*, 8300–8314.

Kwong, P.D., Wyatt, R., Robinson, J., Sweet, R.W., Sodroski, J., and Hendrickson, W.A. (1998). Structure of an HIV gp120 envelope glycoprotein in complex with the CD4 receptor and a neutralizing human antibody. Nature *393*, 648–659.

Li, M., Gao, F., Mascola, J.R., Stamatatos, L., Polonis, V.R., Koutsoukos, M., Voss, G., Goepfert, P., Gilbert, P., Greene, K.M., et al. (2005). Human immunodeficiency virus type 1 env clones from acute and early subtype B infections for standardized assessments of vaccine-elicited neutralizing antibodies. J. Virol. *79*, 10108–10125.

Liao, H.X., Bonsignori, M., Alam, S.M., McLellan, J.S., Tomaras, G.D., Moody, M.A., Kozink, D.M., Hwang, K.K., Chen, X., Tsao, C.Y., et al. (2013). Vaccine induction of antibodies against a structurally heterogeneous site of immune pressure within HIV-1 envelope protein variable regions 1 and 2. Immunity *38*, 176–186.

Lynch, R.M., Wong, P., Tran, L., O'Dell, S., Nason, M.C., Li, Y., Wu, X., and Mascola, J.R. (2015). HIV-1 fitness cost associated with escape from the VRC01 class of CD4 binding site neutralizing antibodies. J. Virol. *89*, 4201–4213.

McLellan, J.S., Pancera, M., Carrico, C., Gorman, J., Julien, J.P., Khayat, R., Louder, R., Pejchal, R., Sastry, M., Dai, K., et al. (2011). Structure of HIV-1 gp120 V1/V2 domain with broadly neutralizing antibody PG9. Nature *480*, 336–343.

McLeod, A.I. (2011). Kendall: Kendall Rank Correlation and Mann-Kendall Trend Test. https://cran.r-project.org/web/packages/Kendall/Kendall.pdf.

Mouquet, H., Scharf, L., Euler, Z., Liu, Y., Eden, C., Scheid, J.F., Halper-Stromberg, A., Gnanapragasam, P.N., Spencer, D.I., Seaman, M.S., et al. (2012). Complex-type N-glycan recognition by potent broadly neutralizing HIV antibodies. Proc. Natl. Acad. Sci. USA *109*, E3268–E3277.

Nelson, J.D., Brunel, F.M., Jensen, R., Crooks, E.T., Cardoso, R.M., Wang, M., Hessell, A., Wilson, I.A., Binley, J.M., Dawson, P.E., et al. (2007). An affinity-enhanced neutralizing antibody against the membrane-proximal external region of human immunodeficiency virus type 1 gp41 recognizes an epitope between those of 2F5 and 4E10. J. Virol. *81*, 4033–4043.

Nickle, D.C., Heath, L., Jensen, M.A., Gilbert, P.B., Mullins, J.I., and Kosakovsky Pond, S.L. (2007). HIV-specific probabilistic models of protein evolution. PLoS One *2*, e503.

Nkolola, J.P., Bricault, C.A., Cheung, A., Shields, J., Perry, J., Kovacs, J.M., Giorgi, E., van Winsen, M., Apetri, A., Brinkman-van der Linden, E.C.M., et al. (2014a). Characterization and immunogenicity of a novel mosaic M HIV-1 gp140 trimer. J. Virol. *88*, 9538–9552.

Nkolola, J.P., Cheung, A., Perry, J.R., Carter, D., Reed, S., Schuitemaker, H., Pau, M.G., Seaman, M.S., Chen, B., and Barouch, D.H. (2014b). Comparison of multiple adjuvants on the stability and immunogenicity of a clade C HIV-1 gp140 trimer. Vaccine *32*, 2109–2116.

Nkolola, J.P., Peng, H., Settembre, E.C., Freeman, M., Grandpre, L.E., Devoy, C., Lynch, D.M., La Porte, A., Simmons, N.L., Bradley, R., et al. (2010). Breadth of neutralizing antibodies elicited by stable, homogeneous clade A and clade C HIV-1 gp140 envelope trimers in guinea pigs. J. Virol. *84*, 3270–3279.

Ofek, G., Tang, M., Sambor, A., Katinger, H., Mascola, J.R., Wyatt, R., and Kwong, P.D. (2004). Structure and mechanistic analysis of the anti-human immunodeficiency virus type 1 antibody 2F5 in complex with its gp41 epitope. J. Virol. *78*, 10724–10737.

Paradis, E., Claude, J., and Strimmer, K. (2004). APE: analyses of phylogenetics and evolution in R language. Bioinformatics *20*, 289–290.

Parrish, N.F., Gao, F., Li, H., Giorgi, E.E., Barbian, H.J., Parrish, E.H., Zajic, L., Iyer, S.S., Decker, J.M., Kumar, A., et al. (2013). Phenotypic properties of transmitted founder HIV-1. Proc. Natl. Acad. Sci. USA *110*, 6626–6633.

Pauthner, M., Havenar-Daughton, C., Sok, D., Nkolola, J.P., Bastidas, R., Boopathy, A.V., Carnathan, D.G., Chandrashekar, A., Cirelli, K.M., Cottrell, C.A., et al. (2017). Elicitation of robust tier 2 neutralizing antibody responses in nonhuman primates by HIV envelope trimer immunization using optimized approaches. Immunity *46*, 1073–1088.e6.

Pedregosa, F., Varoquaux, G., Gramfort, A., Michel, V., Thirion, B., Grisel, O., Blondel, M., Prettenhofer, P., Weiss, R., Dubourg, V., et al. (2011). Scikit-learn: machine learning in Python. J. Mach. Learn. Res. *12*, 2825–2830.

Pejchal, R., Doores, K.J., Walker, L.M., Khayat, R., Huang, P.-S., Wang, S.-K., Stanfield, R.L., Julien, J.-P., Ramos, A., Crispin, M., et al. (2011). A potent and broad neutralizing antibody recognizes and penetrates the HIV glycan shield. Science *334*, 1097–1103.

Peng, H., Long, F., and Ding, C. (2005). Feature selection based on mutual information: criteria of max-dependency, max-relevance, and min-redundancy. IEEE Trans. Pattern Anal. Mach. Intell. *27*, 1226–1238.

Rademeyer, C., Korber, B., Seaman, M.S., Giorgi, E.E., Thebus, R., Robles, A., Sheward, D.J., Wagh, K., Garrity, J., Carey, B.R., et al. (2016). Features of recently transmitted HIV-1 Clade C viruses that impact antibody recognition: implications for active and passive immunization. PLoS Pathog. *12*, e1005742.

Rudicell, R.S., Kwon, Y.D., Ko, S.-Y., Pegu, A., Louder, M.K., Georgiev, I.S., Wu, X., Zhu, J., Boyington, J.C., Chen, X., et al. (2014). Enhanced potency

of a broadly neutralizing HIV-1 antibody in vitro improves protection against lentiviral infection in vivo. J. Virol. *88*, 12669–12682.

Sanders, R.W., Derking, R., Cupo, A., Julien, J.P., Yasmeen, A., de Val, N., Kim, H.J., Blattner, C., de la Pena, A.T., Korzun, J., et al. (2013). A next-generation cleaved, soluble HIV-1 Env trimer, BG505 SOSIP. 664 gp140, expresses multiple epitopes for broadly neutralizing but not non-neutralizing antibodies,. PLoS Pathog. *9*, e1003618.

Sanders, R.W., van Gils, M.J., Derking, R., Sok, D., Ketas, T.J., Burger, J.A., Ozorowski, G., Cupo, A., Simonich, C., Goo, L., et al. (2015). HIV-1 vaccines. HIV-1 neutralizing antibodies induced by native-like envelope trimers. Science *349*, aac4223.

Sarzotti-Kelsoe, M., Bailer, R.T., Turk, E., Lin, C.L., Bilska, M., Greene, K.M., Gao, H., Todd, C.A., Ozaki, D.A., Seaman, M.S., et al. (2014). Optimization and validation of the TZM-bl assay for standardized assessments of neutralizing antibodies against HIV-1. J. Immunol. Methods *409*, 131–146.

Saunders, K.O., Verkoczy, L.K., Jiang, C., Zhang, J., Parks, R., Chen, H., Housman, M., Bouton-Verville, H., Shen, X., Trama, A.M., et al. (2017). Vaccine induction of heterologous Tier 2 HIV-1 neutralizing antibodies in animal models. Cell Rep. *21*, 3681–3690.

Scheid, J.F., Horwitz, J.A., Bar-On, Y., Kreider, E.F., Lu, C.L., Lorenzi, J.C., Feldmann, A., Braunschweig, M., Nogueira, L., Oliveira, T., et al. (2016). HIV-1 antibody 3BNC117 suppresses viral rebound in humans during treatment interruption. Nature *535*, 556–560.

Scheid, J.F., Mouquet, H., Ueberheide, B., Diskin, R., Klein, F., Oliveira, T.Y.K., Pietzsch, J., Fenyo, D., Abadir, A., Velinzon, K., et al. (2011). Sequence and structural convergence of broad and potent HIV antibodies that mimic CD4 binding. Science *333*, 1633–1637.

Sok, D., Pauthner, M., Briney, B., Lee, J.H., Saye-Francisco, K.L., Hsueh, J., Ramos, A., Le, K.M., Jones, M., Jardine, J.G., et al. (2016). A prominent site of antibody vulnerability on HIV envelope incorporates a motif associated with CCR5 binding and its camouflaging glycans. Immunity *45*, 31–45.

Sok, D., van Gils, M.J., Pauthner, M., Julien, J.P., Saye-Francisco, K.L., Hsueh, J., Briney, B., Lee, J.H., Le, K.M., Lee, P.S., et al. (2014). Recombinant HIV envelope trimer selects for quaternary-dependent antibodies targeting the trimer apex. Proc. Natl. Acad. Sci. USA *111*, 17624–17629.

Steichen, J.M., Kulp, D.W., Tokatlian, T., Escolano, A., Dosenovic, P., Stanfield, R.L., McCoy, L.E., Ozorowski, G., Hu, X., Kalyuzhniy, O., et al. (2016). HIV vaccine design to target germline precursors of glycan-dependent broadly neutralizing antibodies. Immunity *45*, 483–496.

Stephenson, K.E., Neubauer, G.H., Reimer, U., Pawlowski, N., Knaute, T., Zerweck, J., Korber, B.T., and Barouch, D.H. (2015). Quantification of the epitope diversity of HIV-1-specific binding antibodies by peptide microarrays for global HIV-1 vaccine development. J. Immunol. Methods *416*, 105–123.

Stewart-Jones, G.B., Soto, C., Lemmin, T., Chuang, G.Y., Druz, A., Kong, R., Thomas, P.V., Wagh, K., Zhou, T., Behrens, A.J., et al. (2016). Trimeric HIV-1-Env structures define glycan shields from clades A, B, and G. Cell *165*, 813–826.

Storey, J.D., and Tibshirani, R. (2003). Statistical significance for genomewide studies. Proc. Natl. Acad. Sci. USA *100*, 9440–9445.

Trkola, A., Purtscher, M., Muster, T., Ballaun, C., Buchacher, A., Sullivan, N., Srinivasan, K., Sodroski, J., Moore, J.P., and Katinger, H. (1996). Human monoclonal antibody 2G12 defines a distinctive neutralization epitope on the gp120 glycoprotein of human immunodeficiency virus type 1. J. Virol. *70*, 1100–1108.

Wagh, K., Bhattacharya, T., Williamson, C., Robles, A., Bayne, M., Garrity, J., Rist, M., Rademeyer, C., Yoon, H., Lapedes, A., et al. (2016). Optimal combinations of broadly neutralizing antibodies for prevention and treatment of HIV-1 Clade C infection. PLoS Pathog. *12*, e1005520.

Walker, L.M., Huber, M., Doores, K.J., Falkowska, E., Pejchal, R., Julien, J.P., Wang, S.K., Ramos, A., Chan-Hui, P.Y., Moyle, M., et al. (2011). Broad neutralization coverage of HIV by multiple highly potent antibodies. Nature *477*, 466–470.

Walker, L.M., Phogat, S.K., Chan-Hui, P.Y., Wagner, D., Phung, P., Goss, J.L., Wrin, T., Simek, M.D., Fling, S., Mitcham, J.L., et al. (2009). Broad and potent neutralizing antibodies from an African donor reveal a new HIV-1 vaccine target. Science *326*, 285–289.

West, A.P., Jr., Scharf, L., Horwitz, J., Klein, F., Nussenzweig, M.C., and Bjorkman, P.J. (2013). Computational analysis of anti-HIV-1 antibody neutralization panel data to identify potential functional epitope residues. Proc. Natl. Acad. Sci. USA *110*, 10598–10603.

Williams, L.D., Ofek, G., Schätzle, S., McDaniel, J.R., Lu, X., Nicely, N.I., Wu, L., Lougheed, C.S., Bradley, T., Louder, M.K., et al. (2017). Potent and broad HIV neutralizing antibodies in memory B cells and plasma. Sci. Immunol. *2*, https://doi.org/10.1126/sciimmunol.aal2200.

Wu, X., Yang, Z.Y., Li, Y., Hogerkorp, C.M., Schief, W.R., Seaman, M.S., Zhou, T., Schmidt, S.D., Wu, L., Xu, L., et al. (2010). Rational design of envelope identifies broadly neutralizing human monoclonal antibodies to HIV-1. Science *329*, 856–861.

Wu, X., Zhang, Z., Schramm, C.A., Joyce, M.G., Kwon, Y.D., Zhou, T., Sheng, Z., Zhang, B., O'Dell, S., McKee, K., et al. (2015). Maturation and diversity of the VRC01-antibody lineage over 15 years of chronic HIV-1 infection. Cell *161*, 470–485.

Wu, X., Zhou, T., Zhu, J., Zhang, B., Georgiev, I., Wang, C., Chen, X., Longo, N.S., Louder, M., McKee, K., et al. (2011). Focused evolution of HIV-1 neutralizing antibodies revealed by structures and deep sequencing. Science *333*, 1593–1602.

Yoon, H., Macke, J., West, A.P., Jr., Foley, B., Bjorkman, P.J., Korber, B., and Yusim, K. (2015). CATNAP: a tool to compile, analyze and tally neutralizing antibody panels. Nucleic Acids Res. *43*, W213–W219.

Zhou, T., Xu, L., Dey, B., Hessell, A.J., Van Ryk, D., Xiang, S.H., Yang, X., Zhang, M.Y., Zwick, M.B., Arthos, J., et al. (2007). Structural definition of a conserved neutralization epitope on HIV-1 gp120. Nature *445*, 732–737.

Zhou, T., Georgiev, I., Wu, X., Yang, Z.Y., Dai, K., Finzi, A., Kwon, Y.D., Scheid, J.F., Shi, W., Xu, L., et al. (2010). Structural basis for broad and potent neutralization of HIV-1 by antibody VRC01. Science *329*, 811–817.

Zhou, T., Lynch, R.M., Chen, L., Acharya, P., Wu, X., Doria-Rose, N.A., Joyce, M.G., Lingwood, D., Soto, C., Bailer, R.T., et al. (2015). Structural repertoire of HIV-1-neutralizing antibodies targeting the CD4 Supersite in 14 donors. Cell *161*, 1280–1292.

Zhou, T., Zhu, J., Wu, X., Moquin, S., Zhang, B., Acharya, P., Georgiev, I.S., Altae-Tran, H.R., Chuang, G.Y., Joyce, M.G., et al. (2013). Multidonor analysis reveals structural elements, genetic determinants, and maturation pathway for HIV-1 neutralization by VRC01-class antibodies. Immunity *39*, 245–258.

STAR★METHODS

KEY RESOURCES TABLE

REAGENT or RESOURCE	SOURCE	IDENTIFIER
Antibodies		
10-1074	Laboratory of Michel Nussenzweig, Rockefeller University	RRID: AB_2491062; Mouquet et al. (2012)
PG16	Polymun	RRID: AB_2491031; Cat#AB016
PGT145	Catalent	RRID: AB_2491054
PGDM1400	Catalent	Sok et al. (2014)
HRP-conjugated goat anti-guinea pig secondary antibody	Jackson ImmunoResearch Laboratories	Cat#106-035-003
Alexa Fluor 647-conjugated AffiniPure Goat Anti-Guinea Pig IgG (H+L)	Jackson ImmunoResearch Laboratories	Cat#706-605-148
Bacterial and Virus Strains		
X1632 T162I pseudovirus	Laboratory of Christina Ochsenbauer, University of Alabama Birmingham	N/A
T250-4 T162I pseudovirus	Laboratory of Christina Ochsenbauer, University of Alabama Birmingham	N/A
BJOX2000 T162I pseudovirus	Laboratory of Christina Ochsenbauer, University of Alabama Birmingham	N/A
Chemicals, Peptides, and Recombinant Proteins		
HIV-1 gp70 V1/V2 (ConC)	Immune Technology Corp	Cat#IT-001-213p
HIV-1 gp70 V1/V2 (Case A2)	Immune Technology Corp	Cat#IT-001-214p
HIV-1 gp70 V1/V2 (CN54)	Immune Technology Corp	Cat#IT-001-211p
HIV-1 gp70 V1/V2 (A244)	Immune Technology Corp	Cat#IT-001-212p
HBS-EP	GE Healthcare	Cat#BR100188
Soluble CD4	Laboratory of Bing Chen, Children's Hospital Boston	Freeman et al. (2010)
Amine Coupling Kit	GE Healthcare	Cat#BR100050
Pierce Recombinant Protein A	ThermoScientific	Cat#21184
Emulsigen	MVP Adjuvants	N/A
Monophosphoryl Lipid A from S. minnesota R595	InvivoGen	Cat#vac-mpla
SuperBlock T20 (TBS) Blocking Buffer	Thermo Scientific	Cat#37536
High-Capacity Protein A Agarose	Thermo Scientific	Cat# 89948
459C WT gp140 foldon	Bricault et al. (2015)	N/A
459C V2 Opt gp140 foldon	This paper	N/A
459C V2 Alt gp140 foldon	This paper	N/A
459C WT SOSIP	This paper	N/A
459C V2 Opt SOSIP	This paper	N/A
C97ZA012 gp140 foldon	Nkolola et al., (2010)	N/A
405C gp140 foldon	Bricault et al., (2015)	N/A
92UG037 gp140 foldon	Nkolola et al., (2010)	N/A
PVO.4 gp140 foldon	Li et al., (2005)	Accession number: AY835444
Mosaic gp140 foldon	Nkolola et al., (2014a)	N/A

(*Continued on next page*)

Continued		
REAGENT or RESOURCE	SOURCE	IDENTIFIER
Deposited Data		
Neutralizing antibody signatures will be deposited in the Los Alamos HIV database and accessible through the Genome Browser, the Neutralizing Antibody relational database, and the Env annotation tables.	Los Alamos HIV Database	https://www.hiv.lanl.gov/content/immunology/neutralizing_ab_resources.html, www.hiv.lanl.gov/components/sequence/HIV/featuredb/search/env_ab_search_pub.comp, www.hiv.lanl.gov/content/sequence/genome_browser/browser.html
Experimental Models: Cell Lines		
Human: 293T	ATCC	ATCC CRL-3216
Experimental Models: Organisms/Strains		
Hartley guinea pigs: Outbred	Elm Hill Labs	N/A
Oligonucleotides		
CpG: 5'-TCGTCGTTGTCGTTTTGTCGTT-3'	Midland Reagent Company	N/A
Recombinant DNA		
GeneArt	Life Technologies	Cat#817003DE
Software and Algorithms		
Softmax Pro-4.7.1	Molecular Devices	https://www.moleculardevices.com/systems/microplate-readers/softmax-pro-7-software
GenePix Pro 7 software	Molecular Devices	https://www.moleculardevices.com/en/asset/br/data-sheets/genepix-pro-software-datasheet-v7-rev-b
GenePix Array List	Stephenson et al., (2015)	N/A
GenSig, a signature analysis web interface	Los Alamos HIV Database	https://www.hiv.lanl.gov/content/sequence/GENETICSIGNATURES/gs.html
CATNAP, Neutralization data resource	Los Alamos HIV Database	https://www.hiv.lanl.gov/components/sequence/HIV/neutralization/
Filtered Forests	Los Alamos HIV Database, github	https://www.hiv.lanl.gov/content/sequence/FLTFORESTS/fltforests.html, https://github.com/hivdb-lanl/FilteredForests
Other		
RepliTope Antigen Collection HIV Ultra slides	JPT Peptide Technologies GmbH	Cat#RT-HD_HIV
CM5 Chips	GE Healthcare	Cat#BR100012

CONTACT FOR REAGENT AND RESOURCE SHARING

Further information and requests for resources and reagents should be directed to and will be fulfilled by the Lead Contact, Dan Barouch (dbarouch@bidmc.harvard.edu).

EXPERIMENTAL MODEL AND SUBJECT DETAILS

Human Subjects

The HIV-1 bNAbs used in this study were all isolated in the context of other studies (Table S1). The Env pseudoviruses are all part of widely used standard panels. Human specimens used to derive these reagents are de-identified and considered exempt by the Duke University IRB, and the exemption approved by the Los Alamos National Lab IRB.

Cell Lines

Human endothelial kidney 293T cells (ATCC) were used for transient transfection of HIV-1 Env expressing plasmids and stably transfected human endothelial kidney 293T cells (Codex Biosolutions) were utilized for the production of HIV-1 Env gp140 and SOSIP immunogens.

Guinea Pig Vaccinations

Healthy, outbred, research-naïve, female Hartley guinea pigs (bred at and purchased from Elm Hill) at between 350 and 500 grams and about 1 to 2 months of age were used for vaccination studies and housed at the Animal Research Facility of Beth Israel Deaconess Medical Center under approved Institutional Animal Care and Use Committee (IACUC) protocols. Animals were co-housed 2 to 5 animals per cage, based on animal weight. All animals were naïve at the initiation of the study. Guinea pigs (5-15/group) were immunized with Env gp140 immunogens intramuscularly in the quadriceps bilaterally at 4-week intervals (weeks 0, 4, 8) for a total of 3 injections. Vaccine formulations for each guinea pig consisted of a total of 100μg of immunogen per injection formulated in 15% Emulsigen (vol/vol) oil-in-water emulsion (MVP Laboratories) and 50 μg CpG (Midland Reagent Company) or 10 μg Monophosphoryl lipid A (MPLA) (InvivoGen) as adjuvants. We also tested the V2-SET immunogen sequences in the context of the gp140 MD39 SOSIP constructs (Steichen et al., 2016), using a lengthened schedule of vaccinations at weeks 0, 8, and 24. Serum samples were obtained from the vena cava of anesthetized animals four weeks after each immunization as well as prior to vaccination for week 0, naïve sera.

METHOD DETAILS

Experimental Methods for Vaccine Evaluation

Plasmids, Cell Lines, Protein Production, and Antibodies

Our baseline immunogen was the C clade Env 459C, initially selected because it elicited tier 1B NAb responses (Bricault et al., 2015), and subsequently found to induce low levels of select tier 2 NAbs upon evaluation of larger tier 2 pseudovirus panels. The codon-optimized synthetic genes of the V2-SET HIV-1 Env gp140 foldon (gp140) and gp140 MD39 SOSIP (SOSIP) immunogens were produced by GeneArt (Life Technologies). All gp140 constructs contained a consensus leader signal sequence peptide, as well as a C-terminal foldon trimerization tag followed by a His-tag as described previously (Frey et al., 2008; Nkolola et al., 2010). Large scale production of HIV-1 Env gp140 foldon and SOSIPs were produced as described previously (Nkolola et al., 2010, 2014a; Steichen et al., 2016). Of note, the gp140 SOSIP immunogens were cleaved by furin and the gp140 foldon immunogens were not. Soluble two-domain CD4 was produced as described previously (Freeman et al., 2010). 10-1074 was generously provided by Michel Nussenzweig (Rockefeller University, New York, NY). PG16 was purchased from Polymun Scientific, PGT145 and PGDM1400 from Catalent, gp70 V1/V2 HIV-1 envelope scaffolds including ConC, Case A2, CN54, and A244 V1/V2 from Immune Technology Corp.

Surface Plasmon Resonance Binding Analysis

SPR experiments were conducted on a Biacore 3000 (GE Healthcare) at 25°C utilizing HBS-EP [10 mM Hepes (pH 7.4), 150 mM NaCl, 3 mM EDTA, 0.005% P20] (GE Healthcare) as the running buffer. Immobilization of CD4 (~1,000 response units (RU)) or protein A (ThermoScientific) to CM5 chips was performed following the standard amine coupling procedure as recommended by the manufacturer (GE Healthcare). Select protein-protein interactions were analyzed using single-cycle kinetics consisting of four cycles of a 1-min association phase and a 4-min dissociation phase without regeneration between injections, followed by an additional cycle of a 1-min association phase and a 15-min dissociation phase, at a flow rate of 50 μL/min. Immobilized IgGs were captured at about 500 RU for 10-1074 and about 3,000 RU for PG16. Soluble gp140 foldon was then passed over the surface at increasing concentrations from 62.5 nM-1,000 nM. Regeneration was conducted with 35 mM NaOH, 1.3 M NaCl (pH 12) at 100 μL/min followed by 5-min equilibration in the HBS-EP buffer. For experiments run with PGDM1400 and PGT145 IgG, immobilized PGDM1400 and PGT145 IgGs were captured at between 150-200 RU. Binding experiments were conducted with a flow rate of 50 μl/min with a 2-minute associate phase and a 5-minute dissociation phase. Soluble gp140 foldon or gp140 SOSIP were then passed over the surface at increasing concentrations from 31.25 nM-500 nM. Regeneration was conducted with one injection (3 seconds) of 35 mM sodium hydroxide, 1.3 M sodium chloride at 100ul/min followed by a 3-minute equilibration phase in HBS-EP. Identical injections over blank surfaces were subtracted from the binding data for analysis. All samples were run in duplicate and yielded similar kinetic results. Single curves of the duplicates are shown in all figures.

Endpoint ELISAs

Serum binding antibodies against gp140 foldon and V1/V2 scaffolds were measured by endpoint enzyme-linked immunosorbant assays (ELISAs) as described previously (Nkolola et al., 2010). Briefly, ELISA plates (Thermo Scientific) were coated with individual gp140s or V1/V2 scaffolds (Immune Technology) and incubated overnight. Guinea pig sera were then added in serial dilutions and later detected with an HRP-conjugated goat anti-guinea pig secondary antibody (Jackson ImmunoResearch Laboratories). Plates were developed and read using the Spectramax Plus ELISA plate reader (Molecular Devices) and Softmax Pro-4.7.1 software. End-point titers were considered positive at the highest dilution that maintained an absorbance >2-fold above background values.

Peptide Microarrays

RepliTope Antigen Collection HIV Ultra slides (JPT Peptide Technologies GmbH) arrays were generated, conducted, and analyzed using methods as described previously (Stephenson et al., 2015). These slides contain linear 15-mer peptides designed utilizing the HIV-1 global sequence database to provide coverage of HIV-1 global sequences (Stephenson et al., 2015). Briefly, microarray slides were incubated with guinea pig sera diluted 1/200 in SuperBlock T20 (TBS) Blocking Buffer (Thermo Scientific). Binding antibody responses were detected with Alexa Fluor 647-conjugated AffiniPure Goat Anti-Guinea Pig IgG (H+L) (Jackson ImmunoResearch Laboratories). All batches of slides were run in parallel with a control slide incubated with the secondary antibody only for background subtraction. Slides were scanned with a GenePix 4300A scanner (Molecular Devices) and analyzed with GenePix Pro 7 software and

GenePix Array List (Stephenson et al., 2015). The threshold values for positivity were calculated as the point at which the chance that signal is noise as low as possible ($P<10^{-16}$). The peak positive antibody binding responses to linear V3 Env peptides were further analyzed comparing the 459C WT and the V2-SET vaccines. Peptides with the highest magnitude binding responses were analyzed comparing geometric means over animals separately against each 15-mer peptide start position. Geometric means were calculated for each vaccination group resulting in a single point per vaccine per peptide sequence.

TZM.bl Neutralization Assay for Vaccine Sera

All IC_{50} data for the large neutralization panels were obtained using the validated luciferase-based TZM.bl assay (Sarzotti-Kelsoe et al., 2014); most antibodies to a maximum concentration of 50 μg/ml. For vaccine responses, 20 tier 2 pseudoviruses were used in the TZM.bl neutralization assay: the standardized global panel of 1HIV-1 reference strains independently selected to be representative of larger global panels (deCamp et al., 2014) and a panel of 8 additional tier 2 pseudoviruses selected because they were relatively sensitive to human sera (falling in the top quartile of geometric mean serological reactivity of the tier 2 panel), were sensitive to the V2 bNAb monoclonals (Yoon et al., 2015), and were relatively close in sequence to the V2-SET vaccines in the neutralization signature positions. The 8 additional pseudoviruses were added as an *a priori* attempt to increase the chances of getting a positive signal, but when tested were found to be very comparable in sensitivity to the global panel. The rationally selected tier 2 pseudoviruses included clade C (Du156.12, CT349_39_16, 234_F1_15_57, CNE58, and CA240_A5.5), CRF 02_AG (T250_4), CRF 07_BC (CNE20), and CRF 01_AE (C3347_C11) strains. For purification of guinea pig polyclonal IgG from sera, High-Capacity Protein A Agarose (Thermo Scientific) was utilized following manufacturer's instructions. After purification by protein A, polyclonal IgG samples were buffer exchanged into 1X phosphate buffered saline, pH 7.4 (Gibco) utilizing a EMD Millipore Amicon Ultra-15 Centrifugal Filter Unit (Millipore) at 4°C. Mutant pseudoviruses were generated with point mutations in V2/glycans to map NAb responses targeting these epitopes. Point mutations aiming to abrogate V2 antibody neutralization were selected to minimize disruptions in the virus backbone by representing mutations that occur commonly in nature. A T162I mutation was introduced into X1632, T250-4, and BJOX2000 to knock out the glycan at position N160; this mutations is relatively common among natural isolates.

Sequence and Signature Analysis

Signature analysis

To systematically identify sites of interest, we used our phylogenetically corrected approach (Gnanakaran et al., 2010) to minimize false positives due to lineage effects (Bhattacharya et al., 2007), and q-values to constrain false positives due to multiple testing (Storey and Tibshirani, 2003). These sites are summarized by antibody in Tables S3A–S3D, and population frequencies of signatures are displayed as LOGOs in Figure 3. Statistical details for all phylogenetically corrected signatures that met the statistical cutoff ($q \leq 0.2$) are provided in a summary table organized by antibody (Tables S3E–S3H). To be more inclusive, within each bNAb class, for sites that have either have a phylogenetically corrected signature for any bNAb within that class or are in the epitope binding region for a crystalized representative of the class, we also list in Tables S3I–S3L all AA and PNGS associations with a q-value <0.2, without the constraint of a phylogenetic correction, organized by Env position (Tables S3I–S3L). For comparison, published bNAb signatures from previous studies (Chuang et al., 2014; Ferguson et al., 2013; West et al., 2013) are also included in Tables S3E–S3L). This comparison shows that our analysis provides more detailed mapping of sites that may be relevant to the overall bNAb sensitivity than has been previously assembled. Because the bNAb field is advancing rapidly and new data are continuously accruing, we have also integrated our signature code into the CATNAP bioinformatics tool into the Los Alamos HIV Immunology Database (Yoon et al., 2015), allowing signature analysis to be conducted on-the-fly as new bNAb data is entered into the database.

Phylogenetically corrected signature methods were described in detail in earlier publications (Bhattacharya et al., 2007; Gnanakaran et al., 2010). Briefly, for a simple uncorrected test, a 2 x 2 contingency table is generated where the data is divided about a phenotypic cutoff (*e.g.* "high" or "low" IC_{50} values split about the median) and whether or not a sequence has a given amino acid at a given position, and a Fisher's exact test is used to assess statistical significance of each such contingency table. All amino acids are tested in all positions, and a false discovery rate (FDR) adjusted q-value (Storey and Tibshirani, 2003) with a threshold of <0.2 used to define sites of interest, to be inclusive but still limit false negatives. This simple test can also be used to test associations with PNGS.

Even with FDR, without a phylogenetic correction simple signatures can yield an extreme over-abundance of apparent results, and many associations will not be causative, but can be carried along by genetic linkage to a site where the variation has direct consequences. An example illustrating how can happen is provided in Figure S7. In this example, the CRF01 clade is highly resistant to V3 bNAbs, and this is likely to be primarily driven the loss of the critical PNGS at N332 throughout the entire clade. But given the lack of reactivity for V3 bNAbs among CRF01 sequences, any amino acid highly enriched among CRF01 sequences will be associated with V3 bNAb resistance, yet most are likely to not be causative. An association that is still statistically supported after a phylogenetic correction, which requires that the correlation between the amino acid and the phenotype recur in sequences in dispersed locations throughout the tree, is more likely to reveal direct causative associations with the phenotype.

For a phylogenetically corrected test, a maximum likelihood tree inferred by the signature code is used to estimate the most likely ancestral amino acids at branch points in the interior of the tree (Bhattacharya et al., 2007). For the branch point preceding each leaf node, the most likely amino acid is determined based on the most likely nucleotides at each position in the codon, which is translated to obtain the ancestral AA state of that leaf. A Fisher's exact test contingency table is based on whether the amino acid changed away from or stayed the same as the ancestral state, and whether the neutralizing phenotype is resistant or sensitive. Full statistics and

contingency tables are provided in Table S3, including detailed examples about how the contingency tables are constructed and their interpretation. As above, this phylogenetically corrected association test can also be used to analyze PNGS associations.

Several cutoffs were used to define relative sensitivity and resistance: IC_{50} titers being above (negative) or below (positive) the threshold of detection based on the highest concentration of Ab used, or partitioning the data about the median or the quartile responses. For a given amino acid at a given site, the results for phylogenetically corrected test with the lowest p-values are shown in Table S3 E-H, with the test performed indicated in the table. If there are ties, they are broken by presenting the undetected vs detected responses (called PosNeg) when they are available, or by presenting the median over the quartile breakdowns if the tie is just between those two cutoffs. We also present uncorrected associations for all amino acids in *positions of interest* with bNAb sensitivity, again with a q-value cutoff of 0.2 (Tables S3I–S3L). This enabled us to explore the potential of amino acids at these interesting positions to contribute to levels of bNAb sensitivity, with a less stringent test than required surviving a phylogenetic correction. Sites were *deemed of interest* for this extended exploration by being either located within epitope, or by being found to be significantly associated with IC_{50} titers using a phylogenetically corrected test for at least one bNAb in a class.

Throughout this study, signatures were generally defined using the Fisher's exact test method described above, but we also explored using a non-parametric Wilcoxon rank sum test. In this case, the distributions of IC_{50} titers were compared when an amino acid or PNGS site was present or absent in a given position, in a phylogenetically corrected analysis. The Wilcoxon test was generally less sensitive than a Fisher's test, however some CD4bs signatures were best defined using this test, and these results are provided in Table S3M.

Antibody references

Links between particular antibodies, references, and antibody provenance and relationships are provided in table format Table S1. Fourteen V3 glycan bNAbs were studied (Bonsignori et al., 2016; Garces et al., 2015; Julien et al., 2013; Kong et al., 2013; Mouquet et al., 2012; Pejchal et al., 2011; Trkola et al., 1996; Walker et al., 2011). Ten V2 bNAbs were analyzed (Bonsignori et al., 2011; Doria-Rose et al., 2014, 2016; McLellan et al., 2011; Sok et al., 2014; Walker et al., 2009, 2011). Twenty-six CD4bs bNAbs were studied (Bonsignori et al., 2017a; Burton et al., 1991; Corti et al., 2010; Diskin et al., 2011; Gao et al., 2014; Huang et al., 2016; Klein et al., 2012; Rudicell et al., 2014; Scheid et al., 2011; Wagh et al., 2016; Wu et al., 2010, 2011, 2015; Zhou et al., 2013, 2015). These were grouped into 3 types (Zhou et al., 2015): VH1-2 restricted, VH1-46 restricted, and those with a CDR H3 dominant mode of binding (Table S1). The 4 MPER bNAbs studied were grouped by epitope, 2F5 or 4E10/10E8/DH511 (Buchacher et al., 1994; Huang et al., 2012; Nelson et al., 2007; Williams et al., 2017)

Phylogenetic trees

Maximum likelihood trees were generated based on amino acid sequences using PhyML (Guindon et al., 2010) using the HIVb model (Nickle et al., 2007) (https://www.hiv.lanl.gov/content/sequence/PHYML/interface.html), and represented using Rainbow Tree at the Los Alamos database (https://www.hiv.lanl.gov/content/sequence/RAINBOWTREE/rainbowtree.html), (Paradis et al., 2004).

Alignments

The signature analysis tool requires as input codon-aligned nucleotide alignments, which we generated using the Gene Cutter tool at https://www.hiv.lanl.gov/content/sequence/GENE_CUTTER/cutter.html, followed with hand editing. The complete dataset alignments for data sets 3 and 4 and TZM.bl neutralization assay IC_{50} data used in this study will be in the Special Interest Alignments page of the Los Alamos upon publication (https://www.hiv.lanl.gov/content/sequence/HIV/SI_alignments/datasets.html), and all of the neutralization data is publicly available through web-based *CATNAP* tool.

Sequence Representation

Amino acids single-letter codes are used throughout. Standard HXB2 numbers is used throughout. The Los Alamos database Analyze Align tool (https://www.hiv.lanl.gov/content/sequence/ANALYZEALIGN/analyze_align.html) was used to generate sequence LOGOS (Crooks et al., 2004). LOGOS represent the frequency of amino acids in the illustrations included here, the measure of interest for this study, in the M group dataset 4, or in the C clade dataset 3. During the course of this study we have built convenient features into the Analyze Align LOGO generation tool: (1) when an N is embedded in a glycosylation site motif Nx[ST], we replace N with the letter O in the LOGO figure, otherwise we leave it as an N, (2) a grey box is used to indicate gaps inserted to maintain the alignment, (3) specific color schemes (e.g. our red/blue sensitivity/resistance color scheme) are now available, and (4) the tool can now make LOGOs of discontinuous sites by utilizing HXB2 numbering.

Hypervariable region characterization

Hypervariable regions were characterized using the Los Alamos database *Variable Region Characteristics* tool: https://www.hiv.lanl.gov/content/sequence/VAR_REG_CHAR/index.html. The variable loops V1, V2, V4, V5 each have hypervariable regions that frequently mutate by insertion and deletion. V3 loops have low levels of mutation by insertion and deletion, thus this region is readily aligned and was not considered hypervariable. The boundaries of these regions are shown in Table S3 R. We systematically tested for correlations with each variable region characteristic (length, net charge, and number of PNGS) and Ab sensitivity for every Ab in each dataset, and calculated q-values to address multiple tests. If an Ab in a class had a q value of < 0.20 with a V-loop correlate, it was considered of interest, and all other Abs of that same class were tracked and included in Tables S3N–S3Q. The hypervariable nature of these regions leads to rapid changes in them within the course of a given infection, so one would expect markedly diminished phylogenetic correlation with these hypervariable loop characteristics across a population, and thus a phylogenetic correction is not appropriate for these analyses. For each bNAb, we tested for correlations between all variable region characteristics and bNAb sensitivity, both including and excluding censored data (Tables S3N–S3Q).

QUANTIFICATION AND STATISTICAL ANALYSIS

Sequence Analyses

Signature Statistics

To address multiple tests false discovery rate q-values were calculated (Storey and Tibshirani, 2003). For all signature comparisons in Table S3, a q-value $\leq$ 0.2 was required for inclusion. We built our own Fisher's exact text code and q-value estimates into the signature analysis package (Bhattacharya et al., 2007). We use R (www.r-project.org/) to perform Wilcoxon rank sum comparisons of distributions, to perform Kendall's tau (McLeod, 2011) to test for correlations, and to calculate q-values for addressing multiple tests when evaluating variable loop characteristics (Dabney and Storey, 2013). Heatmaps were generated using the Los Alamos HIV database tool (https://www.hiv.lanl.gov/content/sequence/HEATMAP/heatmap.html).

Machine learning predictions

We used the Python package scikit-learn for machine learning predictions of bNAb sensitivity (Pedregosa et al., 2011). We initially compared several machine learning strategies (Random Forest, Support Vector Machine, and Linear Discriminant Analysis) using the M group cross-validation scores; the C clade holdout group was not considered during this selection process. Random Forest (RF) (Breiman, 2001) strategies performed best, in particular the ExtraTreesRegressor and ExtraTreesClassifer methods (Geurts et al., 2006) gave the highest accuracy overall, and so were used here as a basis for comparing the accuracy of sequence-based filtering strategies for obtaining input features for neutralization predictions. The overall RF result is obtained by combining the results from each of the individual trees in the forest. For our RF experiments, we fixed the size of the ensemble to 250 trees but otherwise used default values of the scikit-learn parameters. In particular, the depth of the trees (*i.e.,* the number of branches) was adaptively determined using a bootstrap approach available in scikit-learn.

As mentioned in the text, the three pre-filtering strategies used were: mRMR, the full signature set (including outside epitope signatures, hypervariable loop characteristics and clade associations), and only signatures in the bNAb epitopes. Input data files for signature-based prefiltering were created with columns of data translated so that clades, and signature amino acids and PNGSs in a given position, were assigned a 1 if associated with sensitivity, -1 if associated with resistance, or 0 if not associated. Quantitative values for correlated variable loop characteristics were also included.

For each pre-filter strategy, we obtained predictions for two scenarios. First, we predicted IC_{50} titers for the 207 Env sequences from the M group dataset 4, using leave-one-out cross-validation. We decided to use leave-one-out cross validation because the datasets were small enough that we were not computationally constrained, and this approach minimizes bias in small data sets (Arlot and Celisse, 2010). Second, we trained the RF on the M group data from datasets 1,2 and 4, and evaluated the performance on a holdout C clade virus set from dataset 3. To maintain the independence of our holdout set, we used signature pre-filters that were defined only on the basis of the M group datasets 1, 2, and 4, and we excluded the 26 pseudoviruses from the 200 in the C clade set that were also found in M group data. Predictions were made for the 13 bNAbs that were available in both dataset 3 and 4, and both regression (IC_{50} titers; Table S4; Figure 4) and classification (positive versus negative; Table S5) prediction accuracies were assessed. We compared the accuracy of results using different strategies to pre-filter Env sequence alignment data.

For regression, the aim is to predict the potency, and here we used three measures of performance to assess the quality of these predictions. The most direct measure is the mean absolute value of the prediction error (MAE). We also used the R^2 statistic (the coefficient of determination), whose variation is generally (but not strictly) bounded between zero and one, with larger values corresponding to better predictions. Finally, to assess the statistical significance of the predictions vis-a-vis a null hypothesis of no predictive power, we computed the p-value associated with a Kendall's tau test comparing the predictions to the true values. Ranked importance of different features from the RF analysis are provided in Table S6.

For classification, the goal is to predict a binary outcome of whether a bNAb will give detectable neutralization responses against a given sequence or not. The most intuitive measurement of performance is the accuracy, i.e., the fraction of sequences for which the prediction is correct. In some cases, however, simply predicting all positives or all negatives will give a very high accuracy score (e.g. 10E8 neutralizes at some level 98% of the viruses tested), so machine learning prediction is highly accurate, but it is not much better than just predicting that all Envs are positive.

We tested 3 comparisons of particular interest to highlight the importance of signatures in enabling accurate predictions. First, we used only signature sites that were in contact residues versus the complete signatures; the complete list was favored for regression predictions (see Results). Second, we compared using signature sites sequence features as inputs, to using the mRMR approach to filter out the 100 most informative sites (Hepler et al., 2014; Peng et al., 2005). As noted in the Results, complete signatures yielded the most accurate predictions for regression, but there was no clear preference for classification. Before switching to our own mRMR-RF code, to make sure our approach was at least comparable in prediction accuracy to the previously published IDEpi classification code (Hepler et al., 2014), we compared the prediction accuracy of the two methods using 10-fold cross-validation for M group analysis, and also comparing the accuracy for the C clade holdout. Our implementation of the mRMR-RF approach was generally comparable to IDEpi (Table S5), although for a small number of antibodies our error was substantially lower (e.g. VRC01 and 10E8). As a final comparison, because most published computational studies present only a very small number of amino acid signatures for each Ab, we sought to determine whether reducing the number of features to only the strongest features improved the scores, so we limited the Random Forest to include only the 3 most informative features. When comparing this restricted set to the full signature pattern, we found the restricted set not only did not improve classification or regression scores, it often made them much worse.

Vaccine Immune Response Comparisons

Analysis of SET vaccines neutralization data

Neutralization data were analyzed using the R package (www.r-project.org) and GraphPad Prism version 6.00 software (GraphPad Software, San Diego California USA, www.graphpad.com").

We considered ID_{50} titers positive if they were at least 10 above background:

> Cutoff 1: Response = Post, if Post > MuLV + 10; 10 otherwise, where 'Post' is post-vaccination sera ID50 (4 weeks-post last vaccination), 'MuLV' is the background level for an animal-matched MuLV negative control (4 weeks-post last vaccination) (Bricault et al, 2015), and "10" considered below the level of detection. We compared the statistical results presented in Figure 6 with the outcome using alternative cutoffs 2 and 3:
> Cutoff 2: Response = Post - MuLV, if Post-MuLV > 10, 10 otherwise,
> Cutoff 3: Response = Post, if Post >3*MuLV, 10 otherwise,

and found the results obtained using Cutoff 2 and Cutoff 3 were consistent with the results obtained with the cutoff 1 when comparing vaccine groups, so Cutoff 1 is shown.

The breadth of neutralization response (detected vs not-detected) was assessed by counting for each animal the proportion of pseudoviruses with detectable neutralization and then applying the two-sided Wilcoxon rank-sum test to compare the differences in distributions of responses per animal between the 459C WT and the V2-SET vaccines.

The differences in the magnitude of responses between V2-SET vaccines and the 459C WT alone were assessed by a nonparametric permutation test following the strategy described in (Parrish et al., 2013). Briefly, this test compares the medians of responses elicited by the 459C WT and the given V2-SET vaccine in the observed data and in the 10,000 randomized sets of resampled data where the vaccine category is randomly reassigned between vaccinated animals. The fraction of occurrences of median differences in the randomized data that are equal to or less than that observed median differences in the actual data provides an estimate of the probability for observing the actual results by the chance alone.

DATA AND SOFTWARE AVAILABILITY

In this study we have created a catalog of new signature sites, also including those that were defined previously, and created 3 web-based tools to facilitate future analyses: *GenSig* enables users to implement their own phylogenetically corrected signature analysis, *FilteredForests* enables machine learning predictions using either bNAb signature-based or mRMR prefilters, and we have automated neutralization signature predictions for new bNAb neutralization panels as they are incorporated into the Los Alamos HIV database *CATNAP* NAb interface (Yoon et al., 2015).

GenSig

We have developed a Signature Tool web interface, *GenSig*, at the Los Alamos HIV database: https://www.hiv.lanl.gov/content/sequence/GENETICSIGNATURES/gs.html. It can work on any phenotype file given in conjunction with a codon-aligned nucleotide alignment of a protein coding region of moderate size (<1000 gene sequences) – the tool is not specific for HIV-1 and neutralization data. If, however, an input alignment is an HIV-1 gene alignment with the HXB2 reference sequence is included, the numbering of the output will be given according to HIV standardized HXB2 numbering.

CATNAP Enhancement

The HIV-1 pseudovirus sequence data for the viral panels and previously published GenBank accession numbers are all already available through the Los Alamos HIV Database *CATNAP* tool, which we maintain. All new neutralization data used in this study will be integrated into the *CATNAP* tool at the time of publication: https://www.hiv.lanl.gov/catnap. Since new HIV-1 bNAbs are continuously being added to the literature, and new neutralization panel data is regularly entered into the Los Alamos database *CATNAP* tool (Yoon et al., 2015) for comparative analysis (https://www.hiv.lanl.gov/components/sequence/HIV/neutralization/index.html), we have added an automated signature analysis feature to update signatures for new data as it accrues.

FilteredForests

A web interface to run our sequence-based prefilters for machine learning predictions of bNAb sensitivity automatically coupled to RF code from the Python scikit-learn package, (Pedregosa et al., 2011) is called *FilteredForests* code. One can generate their own signature-based prefilters or use mRMR (Peng et al., 2005) to generate from a sequence alignment input files for the RF machine learning codes ExtraTreesRegressor and ExtraTreesClassifer. This web interface is available at: https://www.hiv.lanl.gov/content/sequence/FLTFORESTS/fltforests.html

The code is available at:https://github.com/hivdb-lanl/FilteredForests

bNAb signature information access

To enable comparisons to sites of interest for particular bNAbs identified here, to sites identified in the previously published literature, all signatures identified in this study have been incorporated into the Los Alamos HIV Immunology Databases. The signature

information will be included and accessible through three Los Alamos database tools: a simple spread sheet that is an overview of many of the key findings from the literature, that allows comparisons of findings for all sites (rows) in Env across many antibody studies, organized by paper and/or antibody (columns) (https://www.hiv.lanl.gov/content/immunology/neutralizing_ab_resources.html). The signatures will also be accessible through the relational database we have built for searching bNAb characteristics (Neutralizing Antibody Contexts and Features, (www.hiv.lanl.gov/components/sequence/HIV/featuredb/search/env_ab_search_pub.comp); and the Genome Browser, which allows users to interactively explore functional domains and sites relevant to antibodies across Env (www.hiv.lanl.gov/content/sequence/genome_browser/browser.html).''

Cell Host & Microbe, Volume *25*

Supplemental Information

HIV-1 Neutralizing Antibody Signatures

and Application to Epitope-Targeted Vaccine Design

Christine A. Bricault, Karina Yusim, Michael S. Seaman, Hyejin Yoon, James Theiler, Elena E. Giorgi, Kshitij Wagh, Maxwell Theiler, Peter Hraber, Jennifer P. Macke, Edward F. Kreider, Gerald H. Learn, Beatrice H. Hahn, Johannes F. Scheid, James M. Kovacs, Jennifer L. Shields, Christy L. Lavine, Fadi Ghantous, Michael Rist, Madeleine G. Bayne, George H. Neubauer, Katherine McMahan, Hanqin Peng, Coraline Chéneau, Jennifer J. Jones, Jie Zeng, Christina Ochsenbauer, Joseph P. Nkolola, Kathryn E. Stephenson, Bing Chen, S. Gnanakaran, Mattia Bonsignori, LaTonya D. Williams, Barton F. Haynes, Nicole Doria-Rose, John R. Mascola, David C. Montefiori, Dan H. Barouch, and Bette Korber

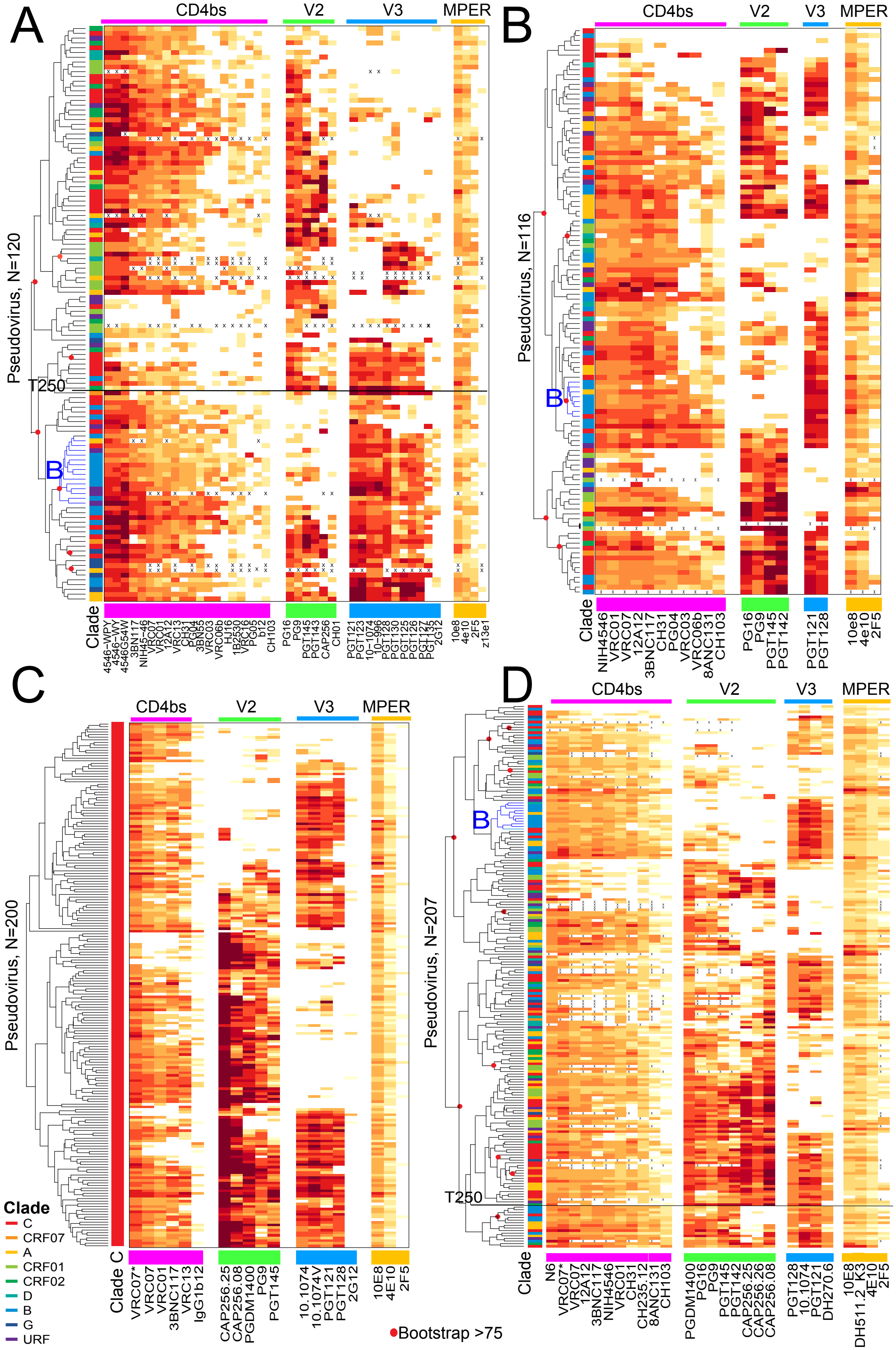
A
CD4bs
V2
V3
MPER
Pseudovirus, N=120
T250
B
Clade
4546-WPY
4546-WY
4546G54W
3BN117
NIH45-46
VRC07
VRC01
12A12
VRC13
CH31
PG04
3BN55
VRC03
VRC06b
HJ16
1B2530
VRC16
PG05
b12
CH103
PG16
PG9
PGT145
PGT143
CAP256
CH01
PGT121
PGT123
10-1074
10-996
PGT128
PGT130
PGT125
PGT126
PGT127
PGT135
2G12
10e8
4e10
2F5
z13e1
B
Pseudovirus, N=116
NIH4546
VRC01
VRC07
12A12
3BNC117
CH31
PG04
VRC03
VRC06b
8ANC131
CH103
PG16
PG9
PGT145
PGT142
PGT121
PGT128
10e8
4e10
2F5
C
Pseudovirus, N=200
Clade C
VRC07*
VRC07
VRC01
3BNC117
VRC13
IgG1b12
CAP256.25
CAP256.08
PGDM1400
PG9
PGT145
10.1074
10.1074V
PGT121
PGT128
2G12
10E8
4E10
2F5
Clade
C
CRF07
A
CRF01
CRF02
D
B
G
URF
Bootstrap >75
D
Pseudovirus, N=207
T250
N6
VRC07*
VRC07
12A12
3BNC117
NIH4546
VRC01
CH31
CH235.12
8ANC131
CH103
PGDM1400
PG16
PG9
PGT145
PGT142
CAP256.25
CAP256.26
CAP256.08
PGT128
10.1074
PGT121
DH270.6
10E8
DH511.2_K3
4E10
2F5

Fig. S1. Heatmaps displaying IC_{50} values of bNAbs and pseudoviruses used in datasets 1-4, the foundational data for Figs. 1-3. Heatmaps displaying IC_{50} values of bNAbs and pseudoviruses used in datasets 1-4, the foundational data for Figs. 1-3. This figure illustrates the extent and nature of the data used for this study, and shared patterns of sensitivity and resistance across antibody classes. **Panels A-D correspond to datasets 1-4, respectively.** Antibodies are arranged by class, indicated across the top of each heat map (CD4bs, magenta; V2, green; V3, blue; and MPER, gold). Neutralization IC_{50} values ranging from low (10-50 ug/ml) to high potency (< 0.0001 ug-ml) are indicated by light yellow through deep red. White indicates an IC_{50} above the threshold of detection (generally 50 ug/ml, but occasionally when antibodies were in limited supply, lesser maximum concentrations were used). The pseudovirus clades are indicated by the colored bars on the left of each heatmap; panel C includes only C clade viruses. A small black "x" indicates no available data. Pseudoviruses are clustered according to like-behavior against the antibody panel according to the dendrogram on the left of each map. Clusters of Envs with bootstrap values >75% are rare, and are indicated as red dots on the dendrograms. There is a recurrent bootstrap-supported cluster enriched for B clade viruses, and indicated by a blue B in panels A, B, and D; the pattern associated with this cluster was V3 sensitivity and V2 bNAb resistance. In contrast, CRF01 viruses are resistant to most V3 bNAbs, and sensitive V2 bNAbs; the statistics of these patterns are explored in detail in later sections of this paper. The antibody 2G12, an older glycan bNAb with limited breadth (Trkola et al., 1996), was grouped with the V3 bNAbs because it includes the PNGS N322 glycan in its epitope; however, because it is so distinctive, it is not included in V3 bNAb summaries. Finally, the virus CRF02_T250.4 is highlighted in parts (A) and (D). T250.4 is extremely sensitive to V2 and V3 bNAbs, but resistant to CD4bs bNAbs). It has hypervariable region characteristics associated with high sensitivity to both V2 and V3 antibodies, so the V1 and V2 loops from CRF02_T250.4 were incorporated into our V2 and V3 SET vaccines (Fig. S6). CRF02_T250.4 is a CRF02 and so subtype A in Env (GenBank accession number EU513189). In panels C and D, VRC07* is an abbreviation for VRC07-523LS. Datasets 1 and 2 share many antibodies, but the pseudoviruses were selected to be completely independent with 120 and 116 viruses included, respectively. Datasets 3 and 4 are larger, with 200 and 207 pseudoviruses, but each contains subsets of viruses that overlap with the other sets. In dataset 4 (D) 208 pseudoviruses were tested, but the full Env gene sequence of one them, 426.c (McGuire et al., 2013) was uncertain, so only 207 viruses were included here. IC_{50} and IC_{80} values were both available, but as IC_{50} is typically a more sensitive indicator for defining signatures, due to having less censored data, it was used here and throughout the paper.

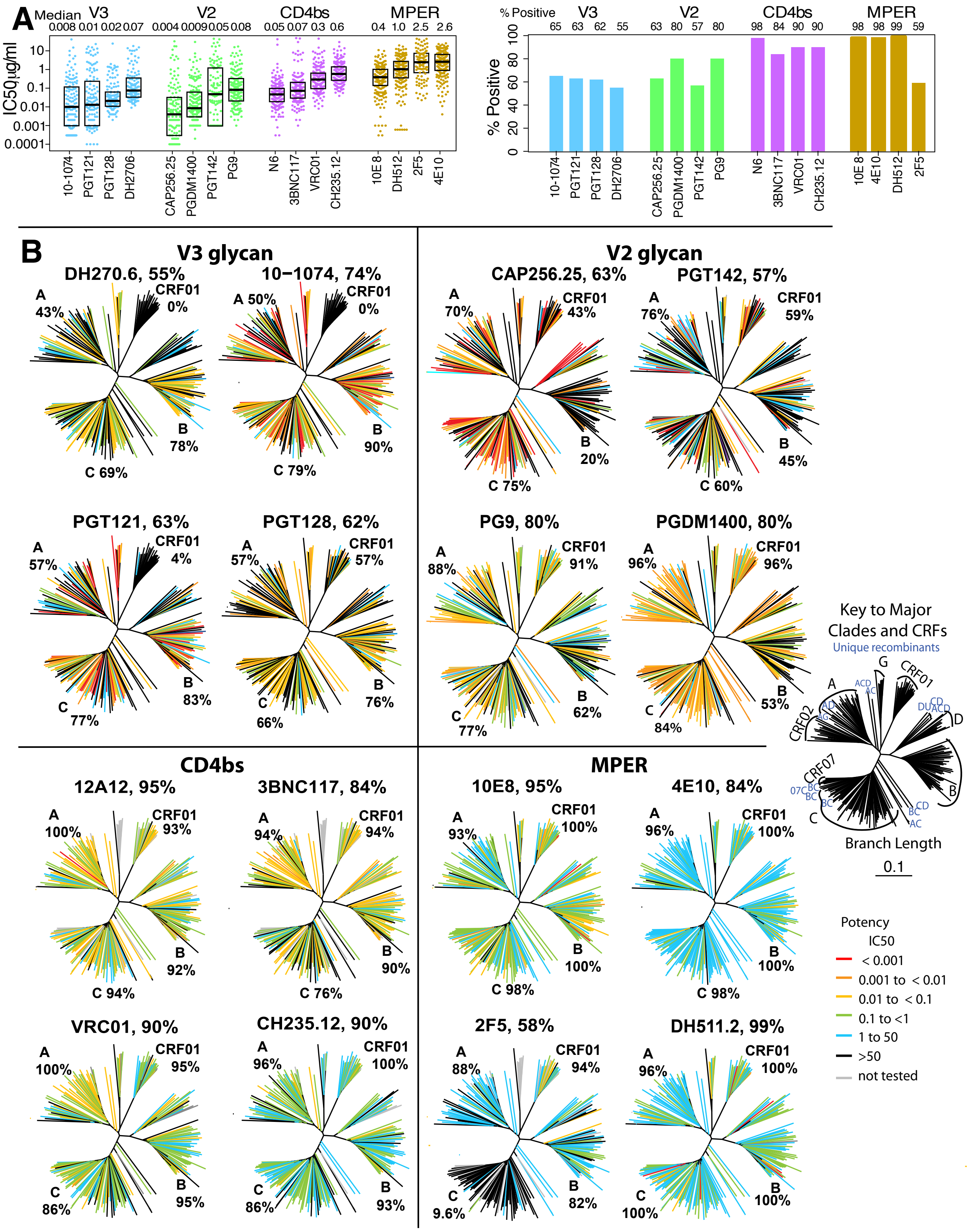
A
Median V3 V2 CD4bs MPER
0.008 0.01 0.02 0.07 0.004 0.009 0.05 0.08 0.05 0.07 0.3 0.6 0.4 1.0 2.5 2.6
IC50μg/ml
10-1074 PGT121 PGT128 DH2706 CAP256.25 PGDM1400 PGT142 PG9 N6 3BNC117 VRC01 CH235.12 10E8 DH512 2F5 4E10
% Positive V3 V2 CD4bs MPER
65 63 62 55 63 80 57 80 98 84 90 90 98 98 99 59
% Positive
10-1074 PGT121 PGT128 DH2706 CAP256.25 PGDM1400 PGT142 PG9 N6 3BNC117 VRC01 CH235.12 10E8 4E10 DH512 2F5
B
V3 glycan
DH270.6, 55%
10−1074, 74%
PGT121, 63%
PGT128, 62%
V2 glycan
CAP256.25, 63%
PGT142, 57%
PG9, 80%
PGDM1400, 80%
CD4bs
12A12, 95%
3BNC117, 84%
VRC01, 90%
CH235.12, 90%
MPER
10E8, 95%
4E10, 84%
2F5, 58%
DH511.2, 99%
Key to Major Clades and CRFs
Unique recombinants
Branch Length
0.1
Potency
IC50
< 0.001
0.001 to < 0.01
0.01 to < 0.1
0.1 to <1
1 to 50
>50
not tested

Fig. S2. A comparison of breadth and potency of bNAb classes using four representative bNAbs from each class, related to Figs. 1-2. The IC_{50} data for these figure was from the most comprehensive multi-clade dataset we studied, dataset 4. (**A) Overall breadth and potency.** The left hand figure illustrates bNAb potency against sensitive strains, and the right shows their breadth in terms of frequency of detectable IC_{50} neutralization responses. This IC_{50} data for this graph was from the most comprehensive multi-clade dataset we studied, dataset 4. (**B) Phylogenetic associations with potency for 4 representative antibodies from each of the 4 classes.** Major clades and circulating recombinant forms (CRFs) are indicated on the detailed black "key" maximum likelihood tree on the far right. CRF01 is common in Asia; it is an AE recombinant that is mostly E in Env. CRF07 is common in China, a BC recombinant, mostly C in Env. CRF02 is common in West Africa, an AG recombinant, mostly A in Env. Clade B dominates the north American and European epidemics, Clade C southern Africa and India, and A is common in Central Africa. Unique recombinants are common among natural isolates, and some are included in this panel; their parental clades are indicated in blue text in the "key" tree. Branch colors in the other 16 trees indicate IC_{50} values, the strength of antibody responses – from red (potent) to blue (weak); black is not detected (see key). The percentage of viruses with a detectable response for 4 major clades that are best sampled, A, B, C and CRF01, are indicated for each clade. 4 representative antibodies for each class are shown. The top left shows V3 glycan antibodies. CRF01 has extreme resistance to these bNAbs. PGT128 is the only one of these four bNAbs able to neutralize CRF01 viruses. A Fisher's exact test comparison of the fraction of detectable neutralized viruses in CRF01 versus other clades show this is highly significant for DH270.6 ($p= 4 \times 10^{-10}$), 10-1074 ($p= 9 \times 10^{-14}$), and PDT121 ($p=4 \times 10^{-11}$), but the effect was not significate for PGT128. The top right shows V2 glycan antibodies. Clade B is highly resistant to CAP256.VRC26 (called CAP256 here) lineage antibodies (Doria-Rose et al., 2015); relative clade B resistance was also found for other V2g bNAbs, including PG9 and PGDM1400, PGT142. The Fisher's exact test p-values for B clade versus others were CAP256.25 ($p = 6 \times 10^{-12}$), PG9 ($p = 0.003$), PGDM1400 ($p = 0.00001$), and PGT142 was not significantly different in the B clade, but this seemed to be due more to a loss of reactivity in other clades than a relative gain within the B clade. There was also a C clade effect found only for CAP256.VRC26 lineage antibodies, in that C clade viruses were more sensitive. CAP256.25 had Fisher's test p-value for positive/negative was $p=0.007$, odds ratio 2.3). The results were more dramatic for CAP256.08, ($p = 0.000095$, OR = 3.4), and also evident for CAP256.26 ($p=0.007$, OR = 2.8). The bottom left shows CD4bs antibodies. All CD4bs bNAbs are very broad, but there are two clade effects worth highlighting. Responses to clade A were more potent. There is also a general tendency for these antibodies to have limited breadth and potency against C clade; 3BNC117 is most impacted, but VRC01 is as well. A Fisher's exact test comparison of the fraction of detectable neutralized viruses among C clade and CRF07, which is mostly C clade in Env, shows a trend of reduced activity (Fisher's $p = 0.037$) for 3BNC117. But if the potency of 3BNC117 is compared against C and CRF07 viruses versus others, a significant difference is observed in level of response (Wilcoxon rank sum $p = 0.00003$), and VRC01 is also shows a trend for less potent responses ($p = 0.027$); these effects are exacerbated for IC80 scores (data not shown). On the other hand, N6 and VRC07 have greater potency and breadth overall, and their potency not particularly compromised among C clade viruses. The bottom right shows MPER antibodies. 2F5 has a strong clade effect, resistance in the C clade, not shared by other MPER bNAbs. Clade A has diminished sensitivity to MPER antibodies, in contrast to CD4bs bNAbs. 10E8, DH511.2, and 4E10 are all very broad; 10E8 is the most potent.

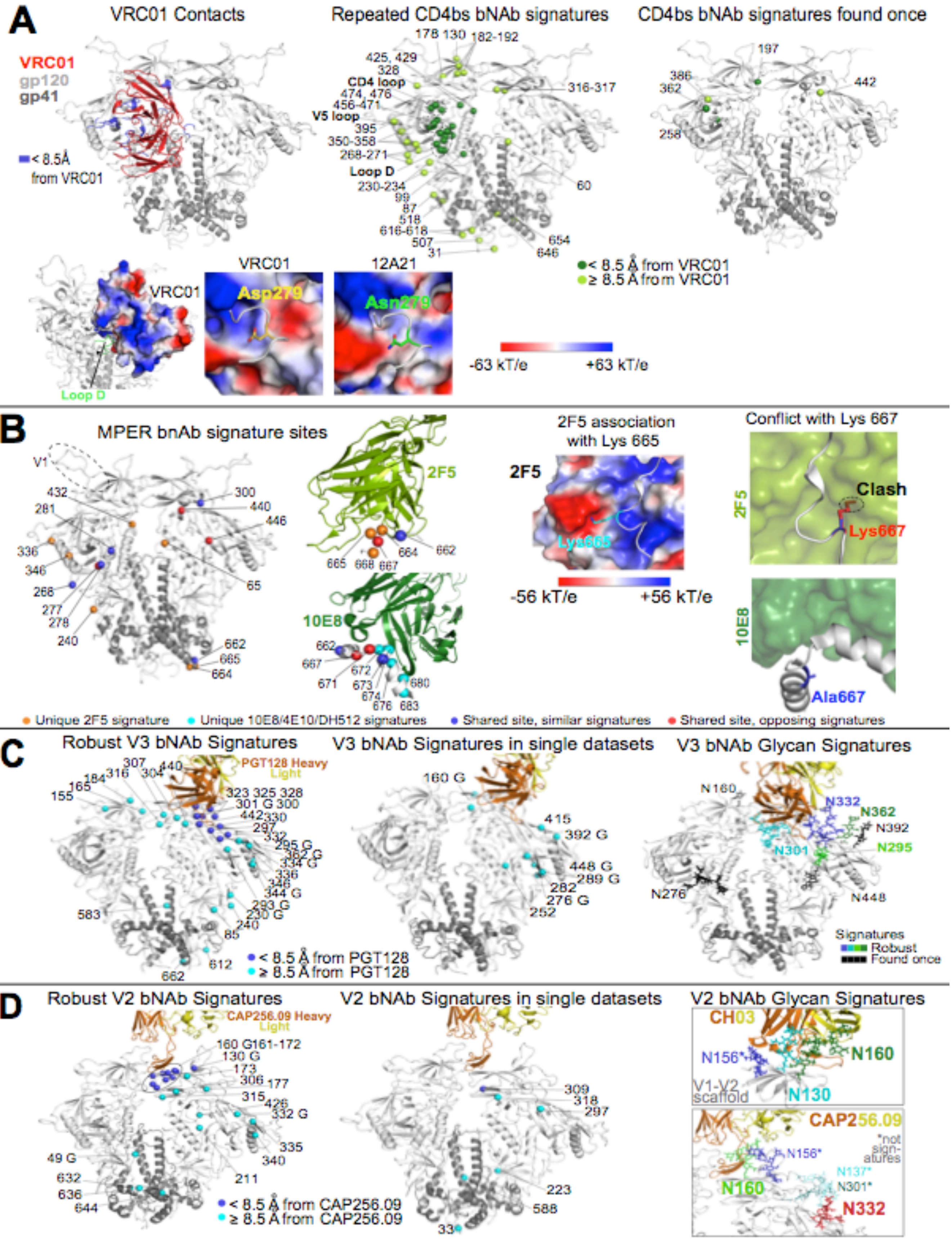
A
VRC01 Contacts
Repeated CD4bs bNAb signatures
CD4bs bNAb signatures found once
VRC01
gp120
gp41
< 8.5Å from VRC01
178 130 182-192
425, 429
328
CD4 loop
474, 476
456-471
V5 loop
395
350-358
268-271
Loop D
230-234
99
87
518
616-618
507
31
316-317
60
654
646
197
386
362
442
258
< 8.5 Å from VRC01
≥ 8.5 Å from VRC01
VRC01
12A21
Asp279
Asn279
Loop D
-63 kT/e
+63 kT/e
B
MPER bnAb signature sites
V1
432
281
336
346
268
277
278
240
300
440
446
65
662
665
664
2F5
665 668 667
664
662
10E8
662
667
671
672
673
674
676
680
683
2F5 association with Lys 665
2F5
Lys665
-56 kT/e
+56 kT/e
Conflict with Lys 667
Clash
Lys667
Ala667
Unique 2F5 signature
Unique 10E8/4E10/DH512 signatures
Shared site, similar signatures
Shared site, opposing signatures
C
Robust V3 bNAb Signatures
V3 bNAb Signatures in single datasets
V3 bNAb Glycan Signatures
PGT128 Heavy
Light
307
184 316
165
155
304 440
323 325 328
301 G 300
442 330
297
332
295 G
362 G
334 G
336
346
344 G
293 G
230 G
240
85
583
612
662
< 8.5 Å from PGT128
≥ 8.5 Å from PGT128
160 G
415
392 G
448 G
289 G
282
276 G
252
N160
N332
N362
N392
N301
N295
N276
N448
Signatures
Robust
Found once
D
Robust V2 bNAb Signatures
V2 bNAb Signatures in single datasets
V2 bNAb Glycan Signatures
CAP256.09 Heavy
Light
160 G161-172
130 G
173
306
177
315
426
332 G
335
340
211
49 G
632
636
644
< 8.5 Å from CAP256.09
≥ 8.5 Å from CAP256.09
309
318
297
223
588
33
CH03
N156*
N160
V1-V2 scaffold
N130
CAP256.09
*not signatures
N156*
N137*
N301*
N160
N332

Fig. S3. Structural mapping and analyses of bNAb signatures, supporting Fig. 3 in the main text.

(A) CD4bs bNAb signatures. The top left panel shows co-crystal structure of VRC01 bound to Env trimer (PDB: 5FYJ, (Stewart-Jones et al., 2016)), with VRC01 contacts (<8.5Å) highlighted in blue. The top row center and right panels show signature sites (spheres) mapped on the Env crystal structure, color-coded according to their proximity to VRC01. The center panel shows the most robust signatures supported in multiple datasets, and the right shows signatures found in only one dataset. The panels in the bottom row show the interaction of site 279 with VRC01 and 12A21, a site of contradictory signatures – D279 was associated with sensitivity to VRC01 and resistance to 12A12 (from the same antibody lineage as 12A21). The left panel shows the location of Loop D and site 279 on the Env trimer with respect to VRC01 in a surface representation color-coded by vacuum electrostatic potential generated using Pymol (Version 1.8, Schrodinger LLC) with negatively/positively charged surfaces shown in red/blue respectively. The center and right panels are zoomed-in views of interaction of D279 with VRC01 and of N279 with 12A21 (PDB: 4JPW (Klein et al., 2013)). Negatively charged D279 interacts with a positively charged cavity on VRC01, potentially explaining why this amino acid is associated with sensitivity. However, for 12A21, N279 is closest to a negatively charged surface, which may be why D279 is not favored in antibodies of this lineage.

(B) MPER bNAb signatures. The structural mapping of signature sites (spheres) from Figure 3 on an Env trimer (left) and on MPER peptides from co-crystal structures with MPER bNAb Fabs (center left). Most of the signatures between 10E8, 4E10, and DH511 lineage antibodies were shared, and were distinct from 2F5 signatures. Thus, the signature sites are color-coded according to whether they were signatures for 2F5 (orange) or for 10E8/4E10/DH511 (cyan) exclusively. Signatures shared across all bNAbs are shown with blue or red spheres depending on whether AAs were concordantly or discordantly associated between 2F5 and other MPER bNAbs, respectively (Fig. 3D). C clade viruses are resistant to 2F5, likely in part because they often carry K667 which would cause unfavorable interactions with 2F5 (Figs. 1, S3). The center right panel shows 2F5 interaction with Lys at 665 (K665), a highly favored signature (Fig. 3). The side chain of K665 is proximal to a strongly negatively charged surface on 2F5, which can explain this preferred 2F5 signature. The right most panels show the position of signature site 667 with respect to 2F5 and 10E8, respectively. Amino acids like K667 and N667 are associated with 2F5 resistance, but are neutral for 10E8 (Table S3). The 2F5 co-crystal structure had A667 (a sensitivity signature) and to understand the impact of these other mutations, we explored modeling K667 (shown) and N667 (not shown) using the "mutagenesis" wizard in Pymol. These models indicate the most likely configuration of K667 could lead to a steric clash with the 2F5 fab (as seen by Lys side chain partially penetrating the translucent 2F5 volume), while A667 (blue) does not. While other clades generally carry Ala at 667, clade C does not, which may contribute to clade C's resistance to 2F5. In contrast, the site at 667 is not proximal to 10E8 Fab (right panel), thus both K667 and N667 variants should be tolerated. Structures used are PDB: 1TJH (Ofek et al., 2004) for 2F5, and PDB: 4G6F (Huang et al., 2012) for 10E8.

(C) V3 bNAb signatures. Left and center panels show the amino acid signatures (spheres) mapped on an Env trimer co-crystal structure with PGT128 Fab (PDB: 5C7K) (Kong et al., 2015). Signatures are stratified according to whether they were found in multiple datasets (left) or in one dataset only (center), and according to whether or not they were within 8.5Å of the PGT128 Fab. Glycan signatures are indicated with a "G" next to them. Robust, multiple-dataset signatures were concentrated in the PGT128 contact regions as compared to the signatures found in only one dataset, but enrichment was not significant using a Fisher's exact test. The right panel shows the mapping of N-linked glycan signatures. All available glycans in the Env-PGT128 co-crystal structure that were signatures for V3 bNAbs are shown in "stick" representation; colored glycans are signatures in multiple datasets, while black glycans are signatures in only a single dataset. The glycans at positions 332 and 301 interact directly with the antibody. The glycans at positions 295 and 392 do not contact PGT128 directly, but are close to N332 (6-15Å minimum distance), and may impact glycan dynamics and processing of N332, thereby indirectly affecting binding. Other glycan signatures are far from PGT128 and key contact glycans (e.g. the glycan at N448, that is ~22-26Å away from PGT128 and N332) but may still subtly influence V3 bNAb sensitivity through an indirect mechanism. Distant glycan signatures were not supported in multiple datasets.

(D) V2 bNAb signatures. The left and center panels show V2 bNAb N-linked glycan signatures mapped on a structural model of CAP256-VRC26.09 bound to an Env trimer (Gorman et al., 2016); color-coding same as in (C). The right panel shows glycan signatures on V1/V2 scaffold bound to CH03 (PDB: 5ESV, Gorman et al., 2016) (top) and on Env-CAP256-VRC26.09 structural model (bottom). The glycans at positions N130, N156, and N160 interact with V2 bNAbs and PNGS's at positions N130 and N160 are signatures. The PNGS at N156 is relatively conserved but not a signature and Envs that lack it are generally sensitive to V2 bNAbs. In the bottom panel, glycans N137 and N301 are not V2 bNAb signatures, but may mediate interactions between signature glycan at N332 and the core glycan at N156.

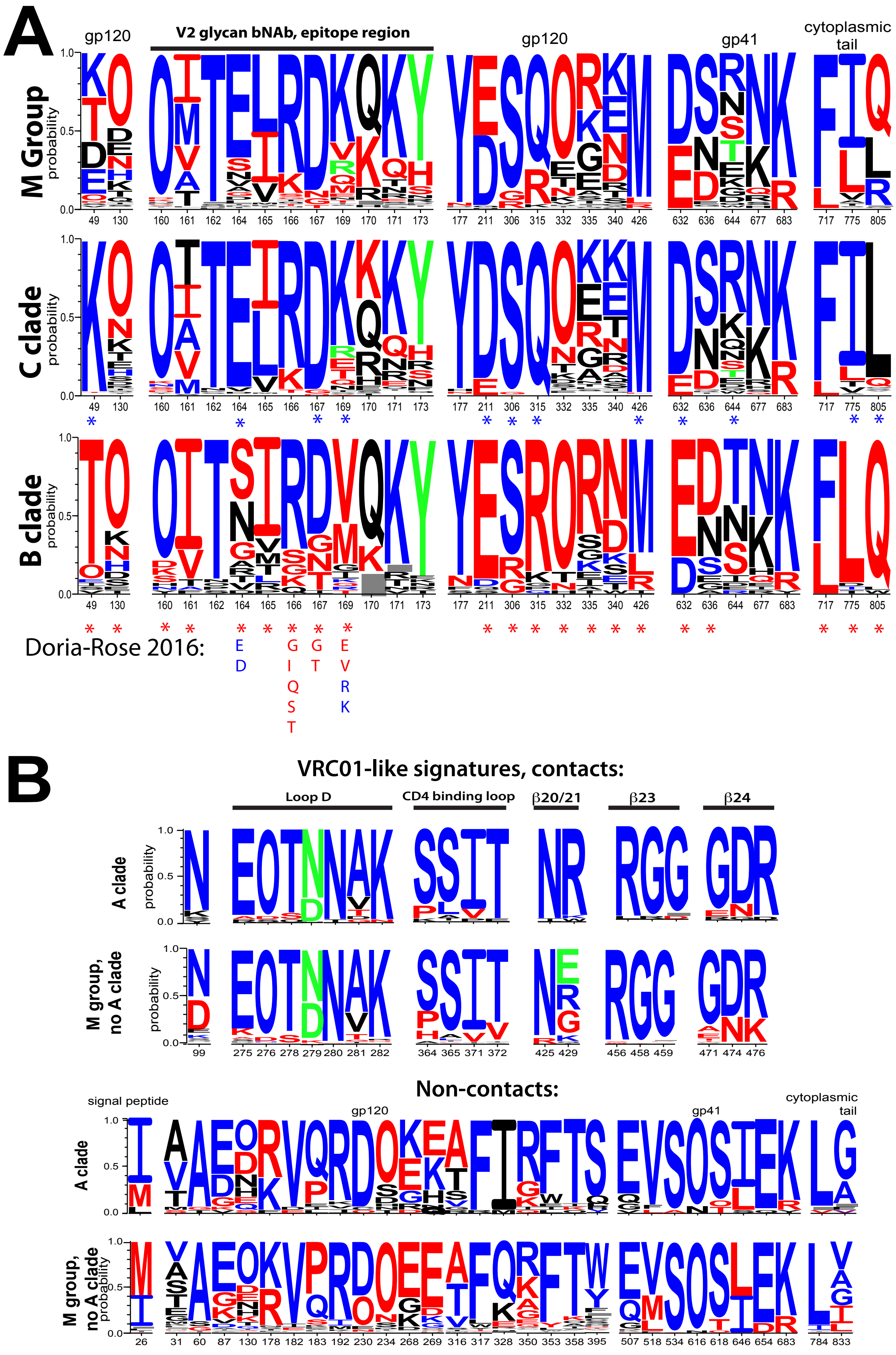
A
gp120
V2 glycan bNAb, epitope region
gp120
gp41
cytoplasmic tail
M Group
C clade
B clade
probability
Doria-Rose 2016:
B
VRC01-like signatures, contacts:
Loop D
CD4 binding loop
β20/21
β23
β24
A clade
M group, no A clade
Non-contacts:
signal peptide
gp120
gp41
cytoplasmic tail

Fig. S4. Signatures associated with clade sensitivity, supporting Figs. 2 and 3. (**A) Specific amino acids that may contribute to the clade preferences of V2 bNAbs.** V2 bNAbs signature frequencies displayed in LOGO plots based on the sequences included in dataset 4, for M-group (top), C clade (middle) and B clade (bottom). The height of the letter corresponds to it frequency. Blue indicates AAs associated with sensitivity, red with resistance, and green with sensitivity to some bNAbs, but resistance to others. (Doria-Rose et al., 2015) proposed that an enrichment for unfavorable signatures, or paucity of favorable signatures, may dictate the resistance of the B clade to the CAP256.VRC26 lineage antibodies, and identified four such signatures near the V2 region. These signatures (AA positions 164, 166, 167, and 169) are noted at the bottom of the figure, and our data support these findings. However, their observations were limited to the core epitope. We found an additional 17 signature sites (marked with red asterisks) where shifts in amino acid frequencies in the B clade (either resistance associated AAs more frequent or reduced frequencies of favorable AAs) may impact overall B clade resistance. CAP256.VRC26 lineage antibodies also have increased potency against C clade viruses and blue asterisks mark signatures that may impact this enhanced potency. Analysis in Table S5 indicates that CAP256.VRC26 antibodies all have striking preferences for K49, E164, and Q315 relative to other V2 bNAbs, so these sites are particularly good candidates for impacting the enhanced potency of the of CAP256.VRC26 bNAbs with C clade viruses. **(B) Signatures that may be associated with increased A clade sensitivity to CD4bs bNAbs.** The signatures associated with CD4bs resistance in contact residues are very rare in A clade relative to the rest of the M group (particularly in AA positions 99, 364, 372, 429, 471 and 475), and this same pattern is also evident in some on the non-contact residues (positions 26, 87, 183, 234, 268, 269, 350, 518, and 646). CD4bs antibodies with enhanced recognition of A clade viruses include: CH31, p=0.0000013; VRC01, p=0.000018; 8AN131, p=0.0004; CH235.12, p=0.00053; PG04, p=0.0011; 12A12, p=0.0028; 3BNC117, p=0.0017; N6, p = 0.04; NIH45.46, p=0.045 (p-values, Wilcoxon rank sum). VRC07, VRC06b, CH103, and VRC03 were not significantly more potent against A clade.

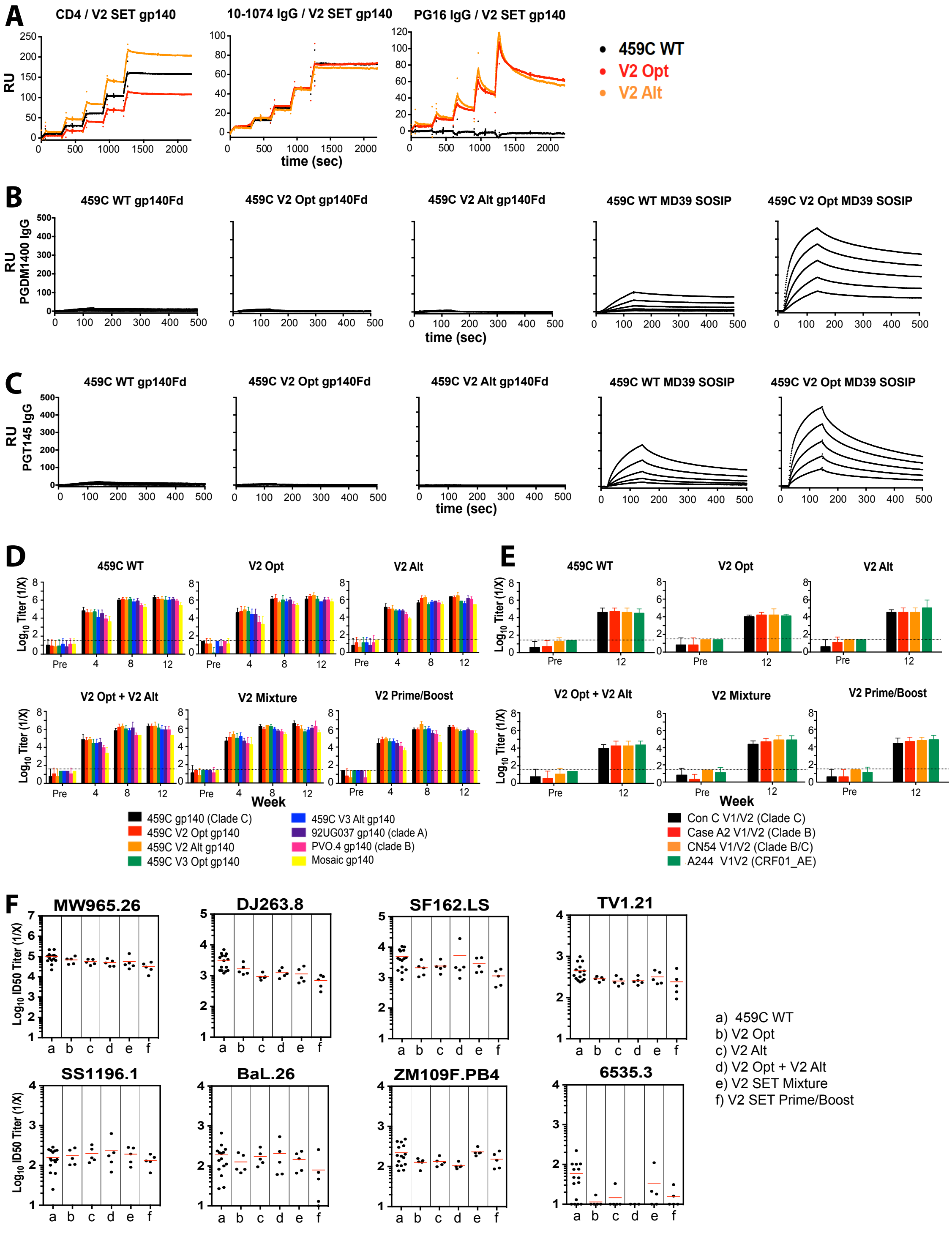
A
CD4 / V2 SET gp140
10-1074 IgG / V2 SET gp140
PG16 IgG / V2 SET gp140
RU
time (sec)
459C WT
V2 Opt
V2 Alt
B
459C WT gp140Fd
459C V2 Opt gp140Fd
459C V2 Alt gp140Fd
459C WT MD39 SOSIP
459C V2 Opt MD39 SOSIP
RU
PGDM1400 IgG
time (sec)
C
459C WT gp140Fd
459C V2 Opt gp140Fd
459C V2 Alt gp140Fd
459C WT MD39 SOSIP
459C V2 Opt MD39 SOSIP
RU
PGT145 IgG
time (sec)
D
459C WT
V2 Opt
V2 Alt
V2 Opt + V2 Alt
V2 Mixture
V2 Prime/Boost
Log10 Titer (1/X)
Pre
Week
459C gp140 (Clade C)
459C V2 Opt gp140
459C V2 Alt gp140
459C V3 Opt gp140
459C V3 Alt gp140
92UG037 gp140 (clade A)
PVO.4 gp140 (clade B)
Mosaic gp140
E
459C WT
V2 Opt
V2 Alt
V2 Opt + V2 Alt
V2 Mixture
V2 Prime/Boost
Log10 Titer (1/X)
Pre
Week
Con C V1/V2 (Clade C)
Case A2 V1/V2 (Clade B)
CN54 V1/V2 (Clade B/C)
A244 V1V2 (CRF01_AE)
F
MW965.26
DJ263.8
SF162.LS
TV1.21
SS1196.1
BaL.26
ZM109F.PB4
6535.3
Log10 ID50 Titer (1/X)
a) 459C WT
b) V2 Opt
c) V2 Alt
d) V2 Opt + V2 Alt
e) V2 SET Mixture
f) V2 SET Prime/Boost

Fig. S5. Antigenicity of and Immunogenicity of V2-SET immunogens in support of Figure 5C, 6 and 7. (A-C) present surface plasmon resonance results. (A) Presentation of the CD4 binding site by soluble CD4, V3/glycan binding site by 10-1074 binding, V2/glycan binding site with PG16 binding to V2-SET gp140 foldon Envs. Sensorgram colors correspond to Env in the key. B-C. Presentation of the (B) PGDM1400 and (C) PGT145 epitopes. Gp140 foldon (gp140) or gp140 SOSIP (SOSIP) Envs were tested as denoted in the graph title. Sensorgrams are presented in black. RU, response units. **(D) Sera from V2-SET vaccinated guinea pigs tested in endpoint ELISAs against a panel of gp140 antigens.** Titles represent the vaccine given. Colors show coating Env as listed. V3-SET proteins are being further studied for 2nd generation vaccine design. (**E) Sera tested in endpoint ELISAs against V1/V2 gp70 scaffolds**. Colors correspond to scaffold origin. The horizontal dotted line indicates background and error bars indicate standard deviation for all endpoint ELISAs. **(F) Guinea pig sera obtained at week 12, tested against a multi-clade panel of tier 1A and 1B, A, B, and C clade neutralization-sensitive isolates in the TZM.bl neutralization assay.** Neutralization data for every data point are MuLV negative control background subtracted. Values less than 10 are set to 10. Horizontal red lines indicate mean titers. Vaccination regimens listed along the x-axis. The title refers to the tested pseudovirus, its tier and clade.

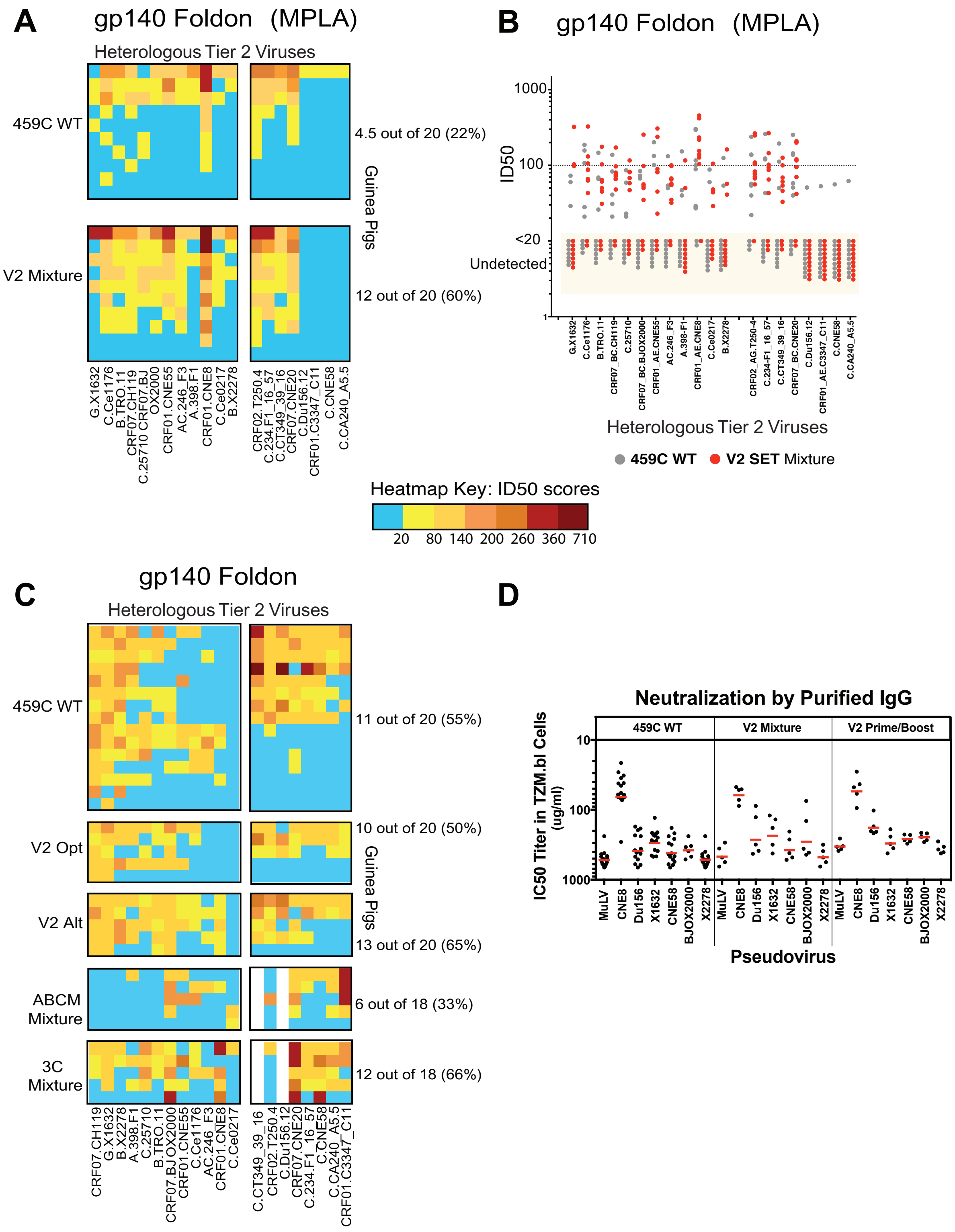
A
gp140 Foldon (MPLA)
Heterologous Tier 2 Viruses
459C WT
4.5 out of 20 (22%)
V2 Mixture
12 out of 20 (60%)
Guinea Pigs
G.X1632
C.Ce1176
B.TRO.11
CRF07.CH119
C.25710 CRF07.BJ
OX2000
CRF01.CNE55
AC.246_F3
A.398.F1
CRF01.CNE8
C.Ce0217
B.X2278
CRF02.T250.4
C.234.F1_16_57
C.CT349_39_16
CRF07.CNE20
C.Du156.12
CRF01.C3347_C11
C.CNE58
C.CA240_A5.5
B
gp140 Foldon (MPLA)
1000
100
ID50
<20
Undetected
1
Heterologous Tier 2 Viruses
459C WT
V2 SET Mixture
Heatmap Key: ID50 scores
20 80 140 200 260 360 710
C
gp140 Foldon
Heterologous Tier 2 Viruses
459C WT
11 out of 20 (55%)
V2 Opt
10 out of 20 (50%)
V2 Alt
13 out of 20 (65%)
Guinea Pigs
ABCM Mixture
6 out of 18 (33%)
3C Mixture
12 out of 18 (66%)
CRF07.CH119
G.X1632
B.X2278
A.398.F1
C.25710
B.TRO.11
CRF07.BJOX2000
CRF01.CNE55
C.Ce1176
AC.246_F3
CRF01.CNE8
C.Ce0217
C.CT349_39_16
CRF02.T250.4
C.Du156.12
CRF07.CNE20
C.234.F1_16_57
C.CNE58
C.CA240_A5.5
CRF01.C3347_C11
D
Neutralization by Purified IgG
459C WT
V2 Mixture
V2 Prime/Boost
IC50 Titer in TZM.bl Cells (ug/ml)
10
100
1000
MuLV
CNE8
Du156
X1632
CNE58
BJOX2000
X2278
Pseudovirus

Fig. S6. Robustness of the enhanced V2-SET vaccine neutralization response, in support of Fig. 6. (A) Heatmap comparisons of Tier 2 bNAbs elicited by 459C WT and V2-SET gp140 foldon vaccines, with an MPLA adjuvant. Guinea pigs were vaccinated intramuscularly at weeks 0, 8, and 24, with 100 µg of 459C WT, or with a total of 100 µg V2-SET proteins divided equally among the 3 Envs, formulated in MPLA. **(B) Comparisons of potency of the responses shown in part (A)**. The dotted line at 100 is added for visual emphasis. Dots in cream colored box are responses below the limit of detection for the assay ($ID_{50} < 20$). Colors represent vaccination regimens. (**C) Heatmap comparisons of tier 2 NAb responses to single components of the trivalent V2-SET vaccine, and two other polyvalent mixtures.** The polyvalent control vaccines included the following previously studied multivalent immunogens: The 3C mixture included 459C WT plus two additional WT clade C gp140s (Bricault et al., 2015), and the tetravalent A, B, and C clade with a Mosaic Env gp140 (ABCM Mixture) (Bricault et al., 2018). The 3C and ABCM Mixtures were analyzed against a panel of 9 C clade and 9 non-C clade pseudoviruses (18 of the original 20 were tested; Du156.12 and CT349_39_16 were not available for testing). Unlike the trivalent V2-SET vaccine, neither the 3C nor the ABCM cocktails afforded significantly enhanced tier 2 breadth as compared with 459C WT; this result did not change when the outliers (the animals with the lowest breadth of response) were removed. **(D) Purified polyclonal IgG from vaccinated guinea pigs evaluated against select tier 2 pseudoviruses and MuLV (the negative control)**. Pseudoviruses are shown alsong the x-axis. The vaccination regimen is depicted in the title. Horizontal red lines indicate mean titers. For the heatmaps in A and C, test columns represent the pseudoviruses noted below the maps, and rows correspond to a single animal grouped by vaccine regimen. Potency of ID_{50} responses are indicated in in the color key, with red to yellow most to least potent. Negative responses are shown in blue. Breadth of response for each vaccination regimen is shown to the right of the heat map as a median number of detected pseudoviruses per animal, out of the number of pseudoviruses tested, with the respective percentage in parentheses.

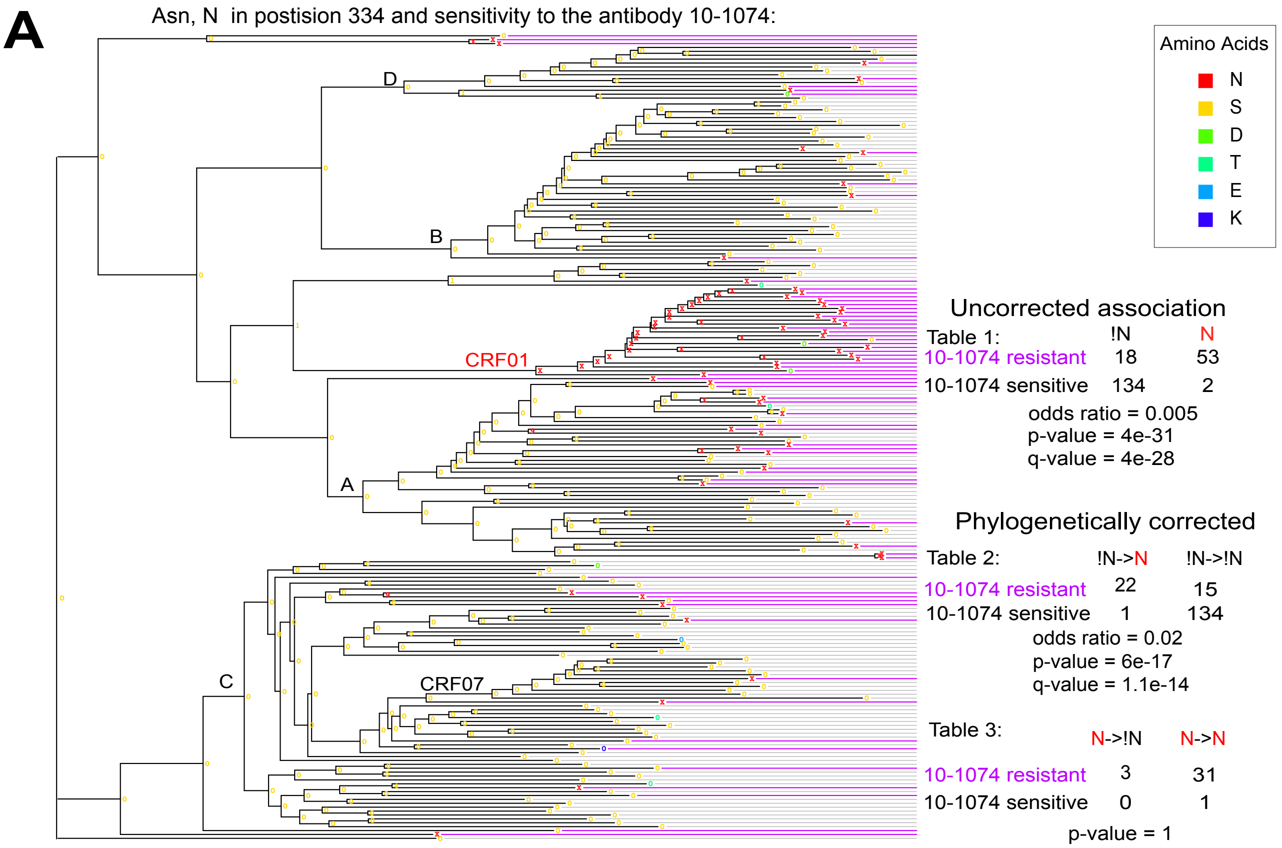
A
Asn, N in postision 334 and sensitivity to the antibody 10-1074:
D
B
CRF01
A
C
CRF07
Amino Acids
N
S
D
T
E
K
Uncorrected association
Table 1: !N N
10-1074 resistant 18 53
10-1074 sensitive 134 2
odds ratio = 0.005
p-value = 4e-31
q-value = 4e-28
Phylogenetically corrected
Table 2: !N->N !N->!N
10-1074 resistant 22 15
10-1074 sensitive 1 134
odds ratio = 0.02
p-value = 6e-17
q-value = 1.1e-14
Table 3: N->!N N->N
10-1074 resistant 3 31
10-1074 sensitive 0 1
p-value = 1

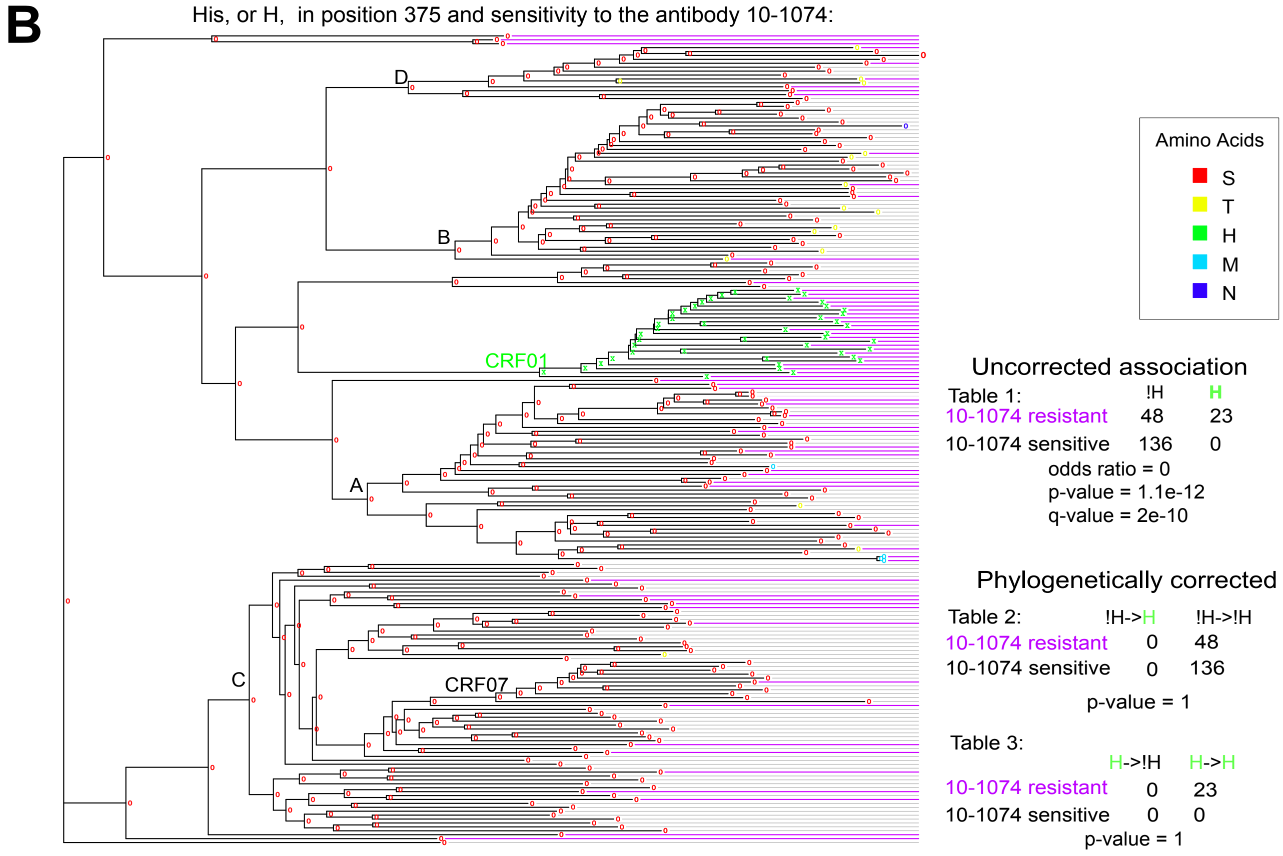
B
His, or H, in position 375 and sensitivity to the antibody 10-1074:
D
B
CRF01
A
C
CRF07
Amino Acids
S
T
H
M
N
Uncorrected association
Table 1: !H H
10-1074 resistant 48 23
10-1074 sensitive 136 0
odds ratio = 0
p-value = 1.1e-12
q-value = 2e-10
Phylogenetically corrected
Table 2: !H->H !H->!H
10-1074 resistant 0 48
10-1074 sensitive 0 136
p-value = 1
Table 3: H->!H H->H
10-1074 resistant 0 23
10-1074 sensitive 0 0
p-value = 1

Fig S7. Maximum likelihood trees with ancestral states and signature statistics illustrating the importance of a phylogenetic correction, supporting in Figure 3. (A) A phylogenetically supported relationship between N334 and 10-1074 resistance. Magenta bars mark resistant pseudotyped viral sequences (leaves), light grey mark sensitive. At the leaves, a red X indicates N334, the amino acid state that is being examined, and other amino acids are represented by other colors. The most likely amino acids in position 334 in the leaves and ancestral nodes are indicated by the color (key on the figure), based on the observed state for the leaves. Maximum likelihood provides an estimate of the most probable state of amino acid 334 given the phylogeny and the evolutionary model for the ancestral states. The number indicates the probability of the ancestral state to have been an Asn, N334 the test amino acid. 0-9 indicates a probability of 0-0.9, and X a probability approaching 1. N334 is almost always present in CRF01 (red X's), but it also recurs many times in scattered places throughout the tree. The simple Fisher's test in Table 1 shows it was present 55 times and only twice when N334 was present was the virus sensitive; this is highly significant. This correlation was also supported in the phylogenetic correction, where we asked if the ancestral node preceding the leaf was most likely not to have not been Asn (N) based on the phylogenetic reconstruction of the ancestral state, and for the end point taxa to have gained an Asn. This happened 23 times in the tree, summarized in Table 2, and 22/23 times that the pseudovirus acquired the N334 relative to its ancestral state, that virus was resistant to 10-1074. This association was also highly significant. Table 3 shows the association between 10-1074 resistance and the phylogenetic loss of N334, which was not significant. **(B) An illustration of the association between H375 and 10-1074 resistance.** In this case, H375 (indicated by green X's at the leaves) is only found in CRF01 and nowhere else in the entire tree. In other clades, the S375 is most prevalent (marked in red) but other amino acids are also found (indicated by other colors). Similar to (A), the ancestral nodes are color-coded by the maximum likelihood ancestral amino acid, with the number indicating the probability of it to be H375. In a simple Fisher's test, H375 is highly associated with 10-1074 resistance, as shown in Table 1. But there is only one branch in the tree with the mutational event resulting in a shift from S375 to H375, it is found in the long branch leading into the CRF01 clade. This long branch leading into CRF01 is indicative of the many other changes that distinguish CRF01 from other clades. So, we cannot be sure if H375 is driving the resistance of the CRF01 lineage, or if it is in linkage with one of the other positions enriched in CRF01 that drive the phenotypic effect, with H375 on its own not having a phenotypic impact on 10-1074 resistance. Thus, there is no phylogenetic support for the association with H375 and resistance, and so it is absent from Table S4. This does not prove or disprove that H375 impacts 10-1074 resistance; it might be a factor in the general resistance in the CRF01 clade but we have no other supporting evidence. In contrast, the recurrence of the N334 happens many times in the tree, and it has a strong association with resistance wherever it occurs, as shown in (A), making it a compelling candidate for being a key mutation underlying the CRF01 resistance to 10-1074, and also suggesting it is important in other clades.

Supplemental Tables

Ab class	Ab	Datasets	Donor	Donor Clade	Clonal Lineage	References
V3	10-1074	1 3 4	Donor 17	A		(Mouquet et al., 2012)
V3	10-1074V	3	Donor 17	A	10-1074	
V3	10-996	1	Donor 17	A		(Mouquet et al., 2012)
glycan	2G12	1				(Trkola et al., 1996)
V3	DH270.6	4	CH0848	C	DH270	(Bonsignori, 2017)
V3	DH270.5	4	CH0848	C	DH270	(Bonsignori, 2017)
V3	DH270.1	4	CH0848	C	DH270	(Bonsignori, 2017)
V3	PGT121	1 2 3 4	Donor 17	A	PGT121	(Garces et al., 2015; Julien et al., 2013; Mouquet et al., 2012; Walker et al., 2011)
V3	PGT123	1	Donor 17	A	PGT121	(Julien et al., 2013; Walker et al., 2011)
V3	PGT125	1	Donor 36	CRF02_AG	PGT128	(Walker et al., 2011)
V3	PGT126	1	Donor 36	CRF02_AG	PGT128	(Walker et al., 2011)
V3	PGT127	1	Donor 36	CRF02_AG	PGT128	(Pejchal et al., 2011; Walker et al., 2011)
V3	PGT128	1 3 4	Donor 36	CRF02_AG	PGT128	(Pejchal et al., 2011; Walker et al., 2011)
V3	PGT135	1	Donor 39	C		(Kong et al., 2013)
V2	CAP256.08	3 4	CAP256	C	VRC26	(Doria-Rose et al., 2015; Doria-Rose et al., 2014)
V2	CAP256.25	3 4	CAP256	C	VRC26	(Doria-Rose et al., 2015; Doria-Rose et al., 2014)
V2	CAP256.26	1 4	CAP256	C	VRC26	(Doria-Rose et al., 2015; Doria-Rose et al., 2014; Gorman et al., 2016)
V2	CH01	1	CH0219	A	CH01	(Bonsignori et al., 2011)
V2	PG9	1 2 3 4	Donor 24	A	PG9	(McLellan et al., 2011; Pejchal et al., 2010; Walker et al., 2009)
V2	PG16	1 2 4	Donor 24	A	PG9	(McLellan et al., 2011; Pejchal et al., 2010; Walker et al., 2009)
V2	PGDM1400	3 4	Donor 84	C		(Sok et al., 2014)

V2	PGT142	2	Donor 84	C	PGT145	(McLellan et al., 2011; Walker et al., 2011)
V2	PGT143	1	Donor 84	C	PGT145	(McLellan et al., 2011; Walker et al., 2011)
V2	PGT145	1 2 3 4	Donor 84	C	PGT145	(McLellan et al., 2011; Walker et al., 2011)
CD4bs	8ANC131	1 2	Patient 8, RU8	B		(Scheid et al., 2011; Zhou et al., 2015)
CD4bs	1B2530	1	Patient 1, RU1	B		(Scheid et al., 2011; Zhou et al., 2015)
CD4bs	CH235.12	4	CH0505	C		(Bonsignori et al., 2016)
CD4bs	12A12	1 2	Patient 12	CRF02_AG		(Scheid et al., 2011; Zhou et al., 2015)
CD4bs	3BC176	1				
CD4bs	3BNC117	1 2	Patient 3	B	3BNC117	Scheid et al., 2011; Zhou et al., 2015)
CD4bs	3BNC55	1	VC10042	B	3BNC117	(Scheid et al., 2011; Zhou et al., 2015)
CD4bs	CH31	1 2	CH0219	A	CH01	(Wu et al., 2011(Wu et al., 2011; Zhou et al., 2015)
CD4bs	NIH45-46	1 2	NIH45	B	VRC01	(Diskin et al., 2011; Scheid et al., 2011)
CD4bs	NIH45-46-WPY	1	NIH45	B	VRC01-modified	
CD4bs	NIH45-46-WY	1	NIH45	B	VRC01-modified	
CD4bs	NIH45-46-G54W	1	NIH45	B	VRC01-modified	(Diskin et al., 2011)
CD4bs	VRC01	1 2	NIH45	B	VRC01	(Wu et al., 2010; Wu et al., 2011; Zhou et al., 2010; Zhou et al., 2015)
CD4bs	VRC07	1 2	NIH45	B	VRC01	(Rudicell et al., 2014)
CD4bs	VRC07.523.LS	3 4	NIH45	B	VRC07-modified	(Rudicell et al., 2014)
CD4bs	VRC03	1 2	NIH45	B	VRC01	(Diskin et al., 2011; Wu et al., 2010; Wu et al., 2015; Wu et al., 2011)
CD4bs	N6	4	Z258	B		(Huang et al., 2016)
CD4bs	VRC06b	1 2	NIH45	B	VRC01	
CD4bs	VRC22	1				
CD4bs	PG04	1 2	Donor 74	A/D	PG04	(Wu et al., 2011; Zhou et al., 2015)
CD4bs	PG05	1	Donor 74	A/D		*

CD4bs	CH103	1 2	CH0505	C		(Gao et al., 2014; Liao et al., 2013)
CD4bs	HJ16	1	V13208	C		(Corti et al., 2010; Zhou et al., 2015)
CD4bs	VRC13	1	Donor 44	B		(Zhou et al., 2015)
CD4bs	VRC16	1	Donor C38	unk		(Zhou et al., 2015)
CD4bs	IgG1b12	1	Donor b	unk		(Burton et al., 1991; Zhou et al., 2015)
MPER	10E8	1 2	Donor N152	B		(Huang et al., 2012)
MPER	2F5	1 2				(Buchacher et al., 1994; Ofek et al., 2004)
MPER	4E10	1 2				(Buchacher et al., 1994; Cardoso et al., 2005)
MPER	Z13E1	1	FDA2	B		(Nelson et al., 2007; Pejchal et al., 2009)
MPER	DH511.2	4	CH0210	C	DH511	(Williams et al., 2017)
MPER	DH511.2_K3	4	CH0210	C	DH511	(Williams et al., 2017)

*PG05 is described in patent application WO 2012040562 A2. The patent indicates PG05 did not compete with other CD4bs antibodies, while PG04 does; however, PG05 is sold through Creative Biolabs as a CD4bs antibody. Given the uncertainty, we are including it with CD4bs set for completeness; it had poor neutralization breadth and gave no strong signatures so does not impact the overall CD4bs signature analysis.

Table S1. bNAbs included in this study, the foundation for Figs. 1-3. The columns indicate the antibody class, common name, the datasets that included the bNAb, the subject ID from whom it was isolated, the HIV-1 clade of the infecting strain, and the clonal lineage that gave rise to the antibody if reported, or a note if the antibody is a modification of a natural antibody, and selected references that describe the antibody isolation, structure, and/or contact residues. The type of CD4bs antibody as described by Zhou et al. (Zhou et al., 2015) is indicated by the color of the antibody name: VH1-46, brown; VH1-2, green; and CDR H3, red; these subdivisions of CD4bs bNAbs were used as breakdowns for integrating signature analysis across antibodies.

	A	B	C	D	G	01	02	07	p-value	tau
**%PNGS N332 dataset 4**	57	88	86	71	83	4	73	86	-	-
**%PNGS N322 database**	67	86	77	80	78	3	83	84	0.017	0.61
**% Neutralization positive DH270.6**	43	78	69	30	86	0	53	86	0.012	0.67
**% Neutralization positive PGT128**	57	76	66	50	71	56	33	71	0.039	0.52
**% Neutralization positive PGT121**	57	83	77	50	86	4	67	86	0.011	0.67
**% Neutralization positive 10-1074**	50	90	79	70	86	0	73	86	0.000001	0.88

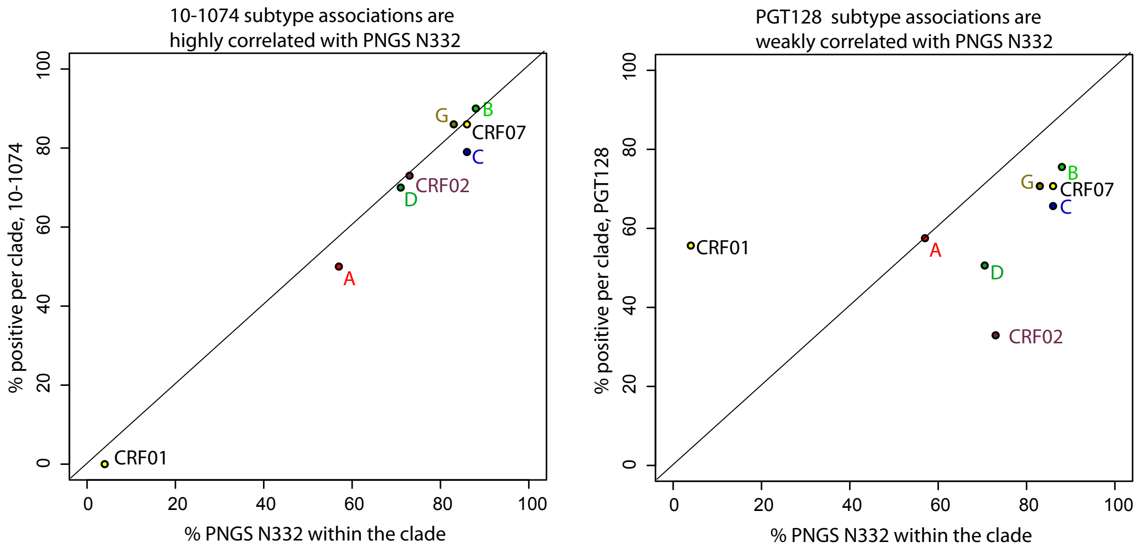

Table S2. N332 frequencies by subtype and recognition of V3 glycan antibodies, supporting Figs. 1-2. The first row shows the percentages of viruses that carry the PNGS N332 in each clade in dataset 4 (207 Envs). The second row is the percentage of the PNGS N332 found in the curated Los Alamos database M group alignment (with 5451 sequences, only one HIV sequence per person in the database, second row), showing that the frequency of the PNGS N332 in dataset 4 is reasonably representative of the larger database. The next 4 rows are the percentage of the viruses that are positive (IC$_{50}$ of <50 ug/ml) for any one subtype for a given V3 bNAb. The lack of PNGS at N332 is very common in some clades, less common in others, and this is significantly correlated with the frequency with which a clade is recognized for each antibody studied. The loss of the PNGS at N332 is clearly not the only way to confer resistance, still it is highly predictive of the recognition within clades. The p-values are from Kendall's tau, and compare the frequencies of the PNGS N332 in dataset 4 to each of the other rows. Underneath the table are two plots to illustrate the relationship between V3 bNAb sensitivity within in a clade and the frequency of PNGS N332, showing the two antibodies with the strongest and weakest correlation. On the left is a plot of the V3 bNAb with the strongest correlation between PNGS N332 and sensitivity, 10-1074. The diagonal line indicates a perfect correlation. This illustrates 10-1074's has a strict dependence on PNGS N332 (p=0.000001, Kendall's tau). PGT128 is the least correlated of the V3 bNAbs (p = 0.039), and is plotted on the right. In the case of PGT128, there must be additional mutations aside from the loss of PNGS N332 commonly driving resistance, as most clades drop well below the diagonal. This is particularly evident in CRF02, a very common in West and Central Africa.

Table S3: All signature tables for all antibodies and datasets. These tables support Figs. 3 and 4.

A-D. Summary tables organized by site, amino acids, and antibodies, and providing an overview of all signature sites found for a given antibody class, across all 4 datasets. Separate tabs are provided for each bNAb class studied (tabs A-D). Sites within hypervariable regions are excluded. The antibodies included in each of the four primary datasets are listed at the top. Sites are included as a signature if at least one antibody in one dataset had a phylogenetically corrected signature site with a q-value of < 0.2 (Table S3 tabs E-H) or if the signature site was in a contact residue. If either of these criteria was met, the site was deemed of interest, and all simple (without a phylogenetic correction) Fisher's exact associations with a q-value < 0.2 were then included and tracked for that site. Complete details of statistical support for each signature are included in Table S3 tabs E-H (phylogenetic corrected signatures only, organized by antibody) and S3 tabs I-L (all simple associations for sites of interest, organized by site). Antibodies with significant associations after a phylogenetic correction (from Table S3 E-H) are bold. The positions are based on HXB2 numbering. If the site is known to be in a contact site for an antibody in the class, based on structural studies, the position number is colored and bold. Amino acids significantly associated with bNAb resistance are colored red, those that are associated with sensitivity are blue. Glycosylation site patterns were tracked, and a PNGS motif is noted as "NxST". Note that lack of an association in a particular dataset does not mean that the association in another is not valid for a given antibody, it may simply mean that a given dataset did not have enough power to resolve the association statistically. If a particular site was associated with bNAbs sensitivity/resistance in multiple datasets, for more than one bNAb in a class, or was located in a known contact residue, it was deemed likely to be robust as it was supported by several lines of evidence (HXB2 position numbers of such sites are highlighted in bold). These more robust associations were used as a basis for the signature profiles in Fig. 3 in the main body of the text and for structural mapping. Contradictory signatures, associated with sensitivity to some antibodies in a class, but resistance to others, are highlighted in tan. Tabs:

A. **V3 bNAb signature summary.** Contact residues are indicated in blue in the Position column
B. **V2 bNAb signature summary.** Contact residues are indicated in green in the Position column.
C. **CD4bs bNAb signature summary.** The HXB2 positions of contact residues as described in the legend to Table S4C are indicated by lavender text. Most CD4bs bNAbs are VH1-2 or VH-46 using and are noted in black. The sites that were used for the illustration in the main paper in Fig. 3 focus on VH1-2 or VH-46 usage CD4bs bNAb signatures. Antibody names in red include CDRH3 dominated antibodies (Zhou et al., 2015) CH103, HJ16, VRC13, VRC16, and IgG1b12, plus two VH1-2 antibodies, VRC03 and VRC06, that tended to track with CDRH3 bNAbs in terms of signature associations. These antibodies generally have less breadth and often have contradictory signatures relative to most VH1-2 or VH-46 using CD4bs bNAbs.
D. **D. MPER bNAb signature summary.** Contact residues are indicated in the Position in dark brown for 2F5, light brown for 4E10/10E8/DH511.

E-H. Phylogenetically corrected signatures, organized by antibody. These tables list the statistical support for all phylogenetically corrected amino acid signatures with q-values <0.2 for each antibody studied, organized by antibody class and antibody, providing details regarding signatures statistics. Separate tabs are provided for each bNAb class studied (S3 E-H). The phylogenetic correction compares to the neutralization phenotype the amino acids in sites that are unchanging to those that change between a taxon and its most recent ancestral node as estimated using a maximum likelihood tree. The column headings are as follows. The "*Table*" columns are T2 and T3, for Table 2 and 3. These tables are phylogenetically corrected signatures. A detailed example of how to read each kind of table is provided in each spreadsheet. If the signature analysis was testing for N-linked glycosylation sites rather than simple amino acids, it is indicated as a "glycan" table, *e.g.* T2glycan. The *Dataset* is either: the first (1) or second (2) completely independent M group datasets, the C clade dataset (3), or the larger M group data (4), shown in Fig. S1. The *cutoff* is the cutoff used for the input phenotype that gave the highest degree of statistical support for a particular signature. PosNeg means the data was broken down between positive, i.e. a detected IC_{50}, and negative, with IC_{50} above the threshold of detection. Data was also broken down by above or below the median titer, and upper and lower quartiles. The *HXB2 pos* is the position in the alignment based on HXB2 numbering. The *test AA* is the amino acid that was being evaluated in the position; only those with a q-value <0.2 are included. Also, we excluded a small number of cases when phylogenetic association were not also supported by a simple uncorrected association. If *blue*, its presence was associated with enhanced sensitivity, if *red*, with resistance. NxST is an abbreviation to refer to an intact N-linked glycosylation site motif. *Antibody* is the name of the bNAb. *P-value, q-value* and *Odds Ratio* are all summary statistics, that are based on the 2x2 contingency tables that are outlined as r1c1, r1c2, r2c1, r2c2, where r stands for row, c for column. See T2 and T3 examples of how to read the contingency tables for the two distinct types of corrections, change towards or away from a given

AA. P-values are based on a 2-sided Fisher's exact test, the q-values were based on all signature p-values. We also list ranked AAs, based on the most informative AAs for our machine learning implementation of *Regression* (predicting IC_{50} values from sequences, Table S4) and *Classification* (predicting positive/negative IC_{50} values from sequences, Table S5). These are listed by rank of importance, followed by the HXB2 position and the amino acid, or a dash if a deletion is important. The next columns show results from other signature analysis papers, including just the associations that were directly reported and readily retrieved from the primary publications. The association is listed alongside signature amino acid we have identified when possible. From West et al. (West et al., 2013) we report associations given as the antibody name, the amino acid association, and the position. Chuang et al. summarizes the published NEP predictions for the 10 highest rank scores (Chuang et al., 2013; Chuang et al., 2014). Hepler et al. (Hepler et al., 2014) associations are from the primary publication using IDEPI. Ferguson et al.'s results are listed as compressed sensing results (given as amino acid and position), ensemble support including mutual information (given as yes or no) and experimental support (given as yes or ND for not done).

E. **V3 bNAb phylogenetically corrected signatures and statistical support.** V3 bNAbs contacts are highlighted in blue, based on two Env bound structures: PGT128 (301, 303, 304, 323-327, 332) (Pejchal et al., 2011) and PGT135 (295, 301, 330, 332, 339, 373, 384, 386, 389, 392, 409, 415, 417-419). The bNAb 2G12 is also included here, even though its epitope is very distinct from the other antibodies included this table.

F. **V2 bNAb phylogenetically corrected signatures and statistical support.** V2 bNAbs contacts are highlighted in green, based on structural contacts for PG9 (McLellan et al., 2011) (contact signatures are: PNGS at N156-158, PNGS at N160-162, 165, 167-171, 173).

G. **CD4bs bNAb phylogenetically corrected signatures and statistical support.** Representative CD4bs bNAbs contacts are highlighted in lavender and are based on an inclusive summary of structural contacts defined for CD4 and CD4bs bNAbs. These contact regions include the following HXB2 positions: V1 proximal: 97-99, 122-129, 196-198, 207, loop D: 275-283, 308, 318, CD4 binding loop: 364-374, beta20/21: 425-432, beta23: 455-459, V5 hypervariable region 460-465, beta 24: 466-477. Sites within the V5 hypervariable region are not included in the signature analysis, even though they can interact directly with the CD4bs bNAbs, due to alignment uncertainty. Contacts regions for CD4bs bNAbs and CD4 were defined based on the following data: CD4 contacts (Wu et al., 2011; Zhou et al., 2010): 124-127, 196-198, 279-283, 365-370, 374, 425-432, 455-461, 469-477; VRC01 contacts (Wu et al., 2011; Zhou et al., 2010): 97, 122, 276, 278-283, 365-368, 371, 427-430, 455-476; IgG1b12 contacts (Zhou et al., 2007): 267, 268, 280, 281, 364-373, 395, 397, 417-419, 430-432, 453-458; NIH45-46 contacts: 97-99, 102, 122-124, 127-128, 276, 427, 430-432, 455-480; 3BNC117 contacts (Scheid et al., 2016): 124, 198, 207, 275-276, 278-282, 308, 318, 365-368, 371, 428-430, 455-462, 469, 473.

H. **MPER bNAb phylogenetically corrected signatures and statistical support.** 2F5 and other MPER antibodies bind to distinct regions. The 2F5 epitope is focused on the sites 662-668 (the HXB2 sequence ELDKWAS) and is highlighted in dark brown. 4E10 is focused on the sites 671-676 (NWFDIT) (Cardoso et al., 2005) and the broader more potent 10E8 extends further out, 671-683, NWFDISNWLWYIK with contacts including positions 671-673 and 676 (Huang et al., 2012). The DH511 lineage binds to an epitope similar to 10E8 (Williams et al., 2017). The 10E8/4E10/DH511 epitopes are highlighted in light brown.

I-M. Amino acid associations with bNAb sensitivity in sites of interest, organized by site. Separate tabs are provided for each bNAb class studied (I-L). We include sites here after a hypothesis has been raised that a site is of interest: if a site is statistically significant after a phylogenetic correction, *i.e.* included in Table S4 for any antibody in a class, or if it is directly in a bNAb contact residue, it is considered of interest for the full bNAb class. Next, Fisher's test for associations of all amino acids at that site with a $q < 0.2$ for all antibodies in that class are listed. "Table 1" (T1) is a contingency table for a simple Fisher's exact test based on the amino acid under consideration in all of the sequences in the set and their IC_{50} breakdowns, with no phylogenetic correction applied. This table uses the same columns headings defined in Table S3 E-H, but the table rows are organized by HXB2 position instead of by antibody. An example of how to read the contingency table is provided in each data spreadsheet. We then list importance-ranked signatures based on our machine learning implementation for regression (levels of sensitivity, Table S4) and classification (positive/negative Table S5), followed by columns that show previously published signatures for antibodies in our study. The association is listed alongside signature sites we have identified, if the earlier finding is also supported by our analyses, or in a separate row if we did not find support for the reported association in our analyses.

I. **Simple signatures associated with V3 bNAb sensitivity in sites deemed of interest.** Contact residues are highlighted in blue.

J. **Simple signatures associated with V2 bNAb sensitivity in sites deemed of interest.** Contact residues are highlighted in green.

K. **Simple signatures associated with CD4bs bNAb sensitivity in sites deemed of interest.** Contact residues are highlighted in lavender.

L. **Additional signatures associated with MPER bNAb sensitivity in sites deemed of interest.** 2F5 Contact residues are highlighted in dark brown, other MPER antibody contacts in light brown.

M. **Results of applying a Wilcoxon test to CD4bs antibodies from dataset 4**. Our signature bioinformatics tool provides an option to use a Wilcoxon rank sum test to compare the IC_{50} score distributions, in the presence or absence of a give amino acid at a given position, and we tested its performance for dataset 4. For most bNAb classes, this yielded fewer signatures and less significant results than the Fisher's exact test for the same data, but CD4bs bNAbs had exceptions listed here. This table includes only cases for which the Wilcoxon yielded comparable or lower p-values than Fishers, and so adds signatures to part C. The number of pseudoviruses with (AA) and without (AA) the signature amino acid, and the median value of the IC_{50} data for that set of pseudoviruses, are noted for each antibody.

N-R. Hypervariable region characteristic signatures. Excel spread sheet, supporting Fig. 2B. The statistics of associations between of V1, V2, V4, and V5 hypervariable region characteristics and IC_{50} scores for each antibody organized by antibody class. Separate tabs are provided for each bNAb class studied (N-Q); and tab R provides a key showing the boundaries of hypervariable regions relative to HXB2. Our analyses considered characteristics of the full-length variable loops, the more narrowly defined hypervariable segment (in bold lettering) that cannot be reliably aligned, and the sum of behaviors across both V1 and V2; we included 10 regions in all, in our search for correlates with bNAb potency. The characteristics of combined V1 + V2 regions were often a stronger correlate of bNAb sensitivity than of either V1 or V2 considered in isolation. Only characteristics that had at least one association based on Kendall's tau with a q-value < 0.2 are captured in this table; once that level of significance was found, the characteristic is considered of potential interest, and all associations between a characteristic and antibodies of the same class are shown. Dataset 3 (C clade) and dataset 4 (M group) are included here, as they are the largest datasets and best powered. If only the hypervariable region of a loop was used for the analysis, it is indicated by an "h", for example V1 means the entire V1 loops was used, V1h means only the hypervariable region. If two highly related characteristics were identified as statistically of interest, like V1 and V1h, only the most significant relationship of the two was retained. The characteristics are: *Charge*, the net charge of the amino acids spanning the region considered (summing over each region such that an Arg, Lys and His contribute +1, Glu and Asp -1); *Length,* the number of amino acids in the region based on the HXB2 boundaries; and *Glycos,* the number of PNGSs within the boundaries of the region under consideration. Kendall's tau was used to calculate p-values.

N. **V loop and hypervariable region characteristics associated with V3 bNAb sensitivity.** Excluding negative IC_{50} responses enhanced correlations, so the impact of loop length on potency among just positive responders was more dramatic. This is likely because viruses are completely resistant when the PNGS at N332 is lost, regardless of loop characteristics. Thus, even viruses with favorable loop characteristics will be negative if the PNGS at N332 is absent, complicating resolution of other characteristics of importance.

O. **V loop and hypervariable region characteristics associated with V2 bNAb sensitivity.**

P. **V loop and hypervariable region characteristics associated with CD4bs bNAb sensitivity.**

Q. **V loop and hypervariable region characteristics associated with MPER bNAb sensitivity** Increasing numbers of PNGS's in the V1 loop correlated with enhanced sensitivity to 10E8, and with other MPER bNAbs to a lesser extent. This was the only case where increasing the size of the variable region was associated with increased bNAb sensitivity.

R. **Hypervariable region boundaries are relative to the HXB2 reference strain V loop sequences.** Hypervariable regions are highlighted in bold and red and are subregions of the full variable region loops.

Regression Predictions			MAE		R^2		p-value	
Class	Antibody	Features	Xval	Hold	Xval	Hold	Xval	Hold
V2	CAP256-VRC26.25	AllSig	1.35	**1.55**	**0.47**	**0.36**	7.50E-27	9.80E-20
V2	CAP256-VRC26.25	Contact	1.46	1.64	0.37	0.27	4.90E-20	1.80E-16
V2	CAP256-VRC26.25	mRMR 3	**1.32**	1.71	0.46	0.27	2.40E-20	5.30E-16
V2	PG9	AllSig	**0.77**	**0.85**	**0.53**	**0.51**	3.50E-27	5.60E-24
V2	PG9	Contact	0.87	1.01	0.4	0.34	7.80E-19	4.00E-16
V2	PG9	mRMR 86	0.87	1.02	0.39	0.31	1.20E-17	4.10E-12
V2	PGDM1400	AllSig	**0.88**	**1.06**	**0.48**	**0.49**	8.80E-25	1.60E-22
V2	PGDM1400	Contact	1.05	1.19	0.34	0.38	7.40E-17	1.40E-15
V2	PGDM1400	mRMR 5	1.05	1.12	0.32	0.36	2.50E-08	9.30E-13
V2	PGT145	AllSig	**1.28**	1.18	**0.27**	0.25	4.90E-14	2.50E-10
V2	PGT145	Contact	1.39	1.33	0.17	0.18	5.00E-09	3.20E-08
V2	PGT145	mRMR 6	1.34	**1.07**	0.18	**0.28**	9.40E-07	1.20E-08
V3	10-1074	AllSig	**0.79**	**0.73**	**0.71**	**0.69**	1.80E-38	1.00E-28
V3	10-1074	Contact	0.8	0.84	**0.71**	0.63	1.50E-33	8.50E-23
V3	10-1074	mRMR 99	0.89	0.82	0.64	0.64	2.80E-29	1.00E-25
V3	PGT121	AllSig	1.14	1.09	0.48	**0.43**	1.60E-28	6.30E-20
V3	PGT121	Contact	1.13	1.37	0.47	0.21	6.10E-25	1.50E-09
V3	PGT121	mRMR 77	**1.12**	**1.08**	**0.50**	0.41	3.00E-28	3.70E-19
V3	PGT128	AllSig	1.11	1.17	**0.37**	**0.39**	6.20E-19	3.10E-17
V3	PGT128	Contact	1.09	1.22	0.36	0.28	1.70E-17	1.50E-12
V3	PGT128	mRMR 15	**1.08**	**1.05**	0.32	0.33	3.30E-16	2.30E-15
CD4bs	3BNC117	AllSig	**0.73**	**0.9**	**0.48**	**0.25**	1.70E-19	2.10E-09
CD4bs	3BNC117	Contact	0.79	1.03	0.35	0.1	1.10E-14	1.80E-04
CD4bs	3BNC117	mRMR 96	0.84	0.94	0.29	0.19	4.20E-12	3.20E-07
CD4bs	VRC01	AllSig	**0.59**	0.74	**0.33**	0.22	1.80E-12	7.00E-10
CD4bs	VRC01	Contact	0.67	0.78	0.2	0.16	8.70E-05	3.80E-05
CD4bs	VRC01	mRMR 94	0.66	**0.72**	0.19	**0.26**	7.50E-06	8.50E-11
CD4bs	VRC07	AllSig	**0.55**	**0.7**	**0.43**	**0.25**	6.40E-14	2.80E-10
CD4bs	VRC07	Contact	0.67	0.76	0.22	0.21	4.30E-03	7.40E-04
CD4bs	VRC07	mRMR 10	0.62	0.76	0.22	0.16	8.40E-01	4.60E-07
CD4bs	VRC07-523-LS	AllSig	**0.63**	**0.66**	**0.35**	**0.1**	1.10E-11	1.50E-06
CD4bs	VRC07-523-LS	Contact	0.76	0.72	0.13	0.1	3.60E-01	4.80E-02
CD4bs	VRC07-523-LS	mRMR 8	0.66	0.69	**0.35**	0.06	4.60E-04	5.60E-04
MPER	10E8	AllSig	0.62	**0.48**	0.15	0.12	6.70E-08	1.00E-03
MPER	10E8	Contact	**0.6**	0.5	**0.18**	**0.15**	9.40E-04	1.20E-04
MPER	10E8	mRMR 24	0.65	0.54	0.07	0.03	2.80E-04	9.10E-01
MPER	4E10	AllSig	**0.53**	**0.59**	**0.13**	0.08	6.90E-05	1.30E-04
MPER	4E10	Contact	**0.53**	0.65	0.08	**0.11**	5.00E-02	1.90E-05
MPER	4E10	mRMR 41	0.55	0.74	0.03	0	4.90E-03	3.20E-01

	R^2 Xval p-value	R^2 Holdout p-value	R^2 Xval: Mean, Median (interquartile range)	R^2 Holdout: Mean, Median (interquartile range)
AllSig			0.39, 0.43 (0.33-0.48)	0.32, 0.25, (0.22-0.43)
Contact	0.003	0.003	0.31, 0.34 (0.18-0.37)	0.24, 0.27 (0.15-0.28)
mRMR	0.002	0.003	0.30, 0.32 (0.19-0.39)	0.25, 0.27 (0.16-0.33

	MAE Xval p-value	MAE Holdout p-value	MAE Xval: Mean, Median (interquartile range)	MAE Holdout: Mean, Median (interquartile range)
AllSig			0.84, 0.77 (0.62-1.11)	0.90, 0.85 (0.70-1.09)
Contact	0.007	0.0008	0.91, 0.80 (0.67-1.09)	1.00, 1.01 (0.76-1.22)
mRMR	0.009	0.05	0.90, 0.87 (0.66-1.08)	0.94, 0.94 (0.74-1.07)

Table S4. Machine learning regression prediction statistical details, supporting Figure 4 in the main text. We compared three strategies for machine learning input data filtering. The full set of genetic signatures for each class of antibody was used as input for the "AllSig" predictions. "Contact" predictions used only amino acid signatures inside the contact region. We also used mRMR (Peng et al., 2005) to pick the top informative 100 features (amino acids in particular positions associated with the neutralization sensitivity), with the most informative sites are listed in the footnotes. In this table, the number that follows the feature selection method mRMR is the number of features for which cross-validation produced the highest prediction score, testing up to 100 features. To compare these feature selection methods we used the ExtraTreesRegressor Random Forest method implemented in the scikit-learn package or predictions (Geurts et al., 2006). "Xval" columns refer to the global M group data (dataset 4) being evaluated for Random Forest predictions using leave-one-out cross validation. "Hold" refers to the C clade independent holdout set predictions evaluated using a model trained on dataset 4. R^2, the coefficient of determination, was calculated by comparing RF predictions to experimentally observed values. Mean Absolute Error (MAE) was also calculated to measure prediction accuracy; the lower the number, the better the prediction. The p-values on the right are based on a non-parametric Kendall's correlation statistic. The best prediction method for each antibody is bold in the MAE column, and for both the cross-validation and the holdout set, using the full set of signatures gave the best predictions overall (see text for statistical summary). The mean, median, and interquartile range of the R^2 statistic across all antibodies is given for the different classes of input, for the cross validation and holdout, and the p-values for a paired Wilcoxon comparing either all signature sites to contacts-only, or all signature sites to mRMR. The p-values and summary statistics indicate using all signatures as a pre-filter generally gave higher accuracy when considered across all antibodies than using only contact signatures, or mRMR.

***REGRESSION footnotes:**

CAP256-VRC26.25	3/3	V169**K** R315**Q** R166**R**
PG9	10/86	O160**O** K171**K** t138d V169**K** q170k -132k**-** 189h**-** F717**F** r633r o750h
PGDM1400	5/5	O160**O** v169e q170- l619l -787d**K**
PGT145	6/6	O160**O** G167**D** v169e o332o m426r l134t
10-1074	10/99	O332**O** N300**N** n325k -363ap -363a**S** e824g V833**V** l755i K362**O** t341a
PGT121	10/77	O332**O** N300**N** I148- 363a**S** V255**V** N325**D** S334**S** S143- O301**O** v255i
PGT128	10/15	O332**O** O295**O** i320- T297**T** O301**O** I165**I** k151q H330**H** S334**S** R304**R**
3BNC117	10/96	G459**G** G471**G** q442o r853a M026**M** n462d R456**R** q621d -363a**S** k033d
VRC01	10/94	R456**R** 012a**-** a060g -397c**-** g471i q805r I371**I** d279**D** T450**T** -363ah
VRC07	10/10	R456**R** g471i G459**G** **-**132qd N355- O276**O** -363b**-** f353y r456s N280**N**
VRC07-523-LS	8/8	R456**R** g471i G459**G** N355- O276**O** r456s N280**N** Q258**Q**
10E8	10/24	t676s E268**E** r308h n671t l134s e351k e403l p369v -189h**-** i182e
4E10	10/41	k231e n325n o406a L025**L** s411n k155t e409d e464e t188q -189en
2F5	3/3	A667A K665**K** E662**E**

***Footnotes:** For each antibody, the number following mRMR in the table indicates the number of features corresponding to the N best features according to the random forest feature importance score, out of the M features that were chosen by mRMR, where M is chosen by yielding the best cross-validation score. N/M is shown for each antibody in the footnotes, followed by up to 10 of the N best features for each bNAb ordered by rank of importance. The feature naming convention is: Letter-Number-Letter, where Number is the HXB2 site number, the first Letter is the HXB2 residue at that site, and the last Letter is the signature residue. O stands for a PNGS. For features for which the HXB2 residue is "-" (a dash inserted to maintain the alignment), we use the format of Number-Letter-Letter, where now Number-Letter is the site number (e.g., 363a) which is the first "-" after HXB2 site 363. The last letter is the residue at that site in the sequence that is the one important for predictions. If the letter is uppercase and bold, it is associated with increased sensitivity, lower case plain text, resistance.

Class	Antibody	Fxn Positive		Features	Accuracy		Neg-accuracy		Pos-accuray		MCC		p-value	
		M group	C clade		Xval	Hout	Xval	Hout	Xval	Hout	Xval	Hout	Xval	Hout
V2	PG9	0.8	0.71	AllSig	0.87	**0.82**	0.49	0.39	0.97	0.99	0.55	**0.54**	1.50E-11	1.70E-11
				Contact	0.87	0.8	0.54	0.43	0.96	0.95	0.56	0.47	4.50E-12	4.50E-09
				mRMR	0.88	0.79	0.56	0.33	0.96	0.98	**0.58**	0.44	6.30E-13	6.00E-08
				IDEpi	**0.89**	0.80					**0.58**	0.48		
V2	CAP256-VRC26.25	0.63	0.72	AllSig	0.76	**0.75**	0.64	0.23	0.84	0.95	0.49	0.27	4.70E-12	8.90E-04
				Contact	0.73	0.73	0.64	0.38	0.78	0.87	0.42	**0.28**	2.90E-09	5.20E-04
				mRMR	**0.8**	0.72	0.6	0	0.92	0.99	**0.57**	-0.05	4.80E-16	1
				IDEpi	0.78	0.72					0.52	-0.05		
V2	PGDM1400	0.8	0.74	AllSig	**0.89**	0.86	0.51	0.44	0.99	1	0.63	0.61	1.90E-15	4.20E-14
				Contact	0.86	0.84	0.46	0.44	0.95	0.98	0.49	0.56	5.90E-10	6.90E-12
				mRMR	0.9	**0.88**	0.59	0.6	0.98	0.98	**0.65**	**0.69**	1.40E-16	1.90E-17
				IDEpi	0.87	0.84					0.5	0.56		
V2	PGT145	0.75	0.71	AllSig	0.81	0.82	0.44	0.43	0.93	0.98	0.42	0.55	5.90E-08	7.50E-12
				Contact	0.81	0.79	0.5	0.45	0.91	0.93	0.45	0.46	4.90E-09	1.20E-08
				mRMR	**0.84**	**0.83**	0.5	0.41	0.95	1	**0.53**	**0.57**	9.00E-12	2.10E-13
				IDEpi	0.82	0.80					0.43	0.47		
V3	10-1074	0.66	0.63	AllSig	0.92	**0.95**	0.85	0.89	0.96	0.99	0.83	**0.9**	1.80E-34	1.30E-36
				Contact	**0.93**	0.92	0.87	0.86	0.96	0.95	**0.84**	0.83	1.20E-35	1.60E-29
				mRMR	**0.93**	0.93	0.85	0.84	0.97	0.98	**0.84**	0.85	1.50E-35	6.80E-32
				IDEpi	0.92	0.92					**0.84**	0.84		
V3	PGT121	0.63	0.69	AllSig	0.79	0.8	0.64	0.66	0.87	0.87	0.53	0.53	2.70E-14	9.00E-12
				Contact	**0.83**	0.73	0.74	0.53	0.88	0.82	**0.62**	0.36	3.70E-19	6.20E-06
				mRMR	0.82	0.79	0.68	0.58	0.9	0.88	0.61	0.49	2.20E-18	4.20E-10
				IDEpi	0.81	**0.83**					0.59	**0.60**		
V3	PGT128	0.62	0.54	AllSig	**0.78**	**0.79**	0.68	0.67	0.84	0.88	**0.53**	**0.57**	4.50E-14	3.70E-14
				Contact	0.74	0.76	0.59	0.66	0.84	0.85	0.45	0.52	3.30E-10	4.10E-12
				mRMR	0.76	0.75	0.63	0.54	0.84	0.93	0.48	0.52	7.60E-12	5.70E-12
				IDEpi	0.70	0.73					0.39	0.45		
CD4bs	VRC01	0.9	0.79	AllSig	**0.93**	0.82	0.4	0.35	0.99	0.94	**0.54**	0.37	9.60E-08	1.70E-05
				Contact	0.92	**0.83**	0.4	0.43	0.98	0.93	0.48	**0.43**	9.40E-07	5.10E-07
				mRMR	**0.93**	**o.83**	0.3	0.3	1	0.98	0.53	0.41	4.90E-07	2.60E-06
				IDEpi	0.74	**0.83**					0.26	0.39		
CD4bs	3BNC117	0.85	0.8	AllSig	**0.9**	0.83	0.46	0.51	0.97	0.91	**0.54**	0.45	2.70E-09	8.30E-08
				Contact	0.88	0.78	0.46	0.57	0.95	0.83	0.48	0.38	6.80E-08	3.60E-06
				mRMR	0.88	**0.87**	0.32	0.49	0.99	0.96	0.47	**0.54**	6.60E-07	4.80E-10
				IDEpi	0.86	0.83					0.22	0.37		
CD4bs	VRC07	0.93	0.87	AllSig	**0.95**	**0.88**	0.46	0.41	0.99	0.95	**0.56**	**0.4**	1.10E-06	2.10E-05
				Contact	0.94	**0.88**	0.31	0.41	0.99	0.95	0.43	**0.4**	2.40E-04	2.10E-05
				mRMR	0.94	**0.88**	0.23	0.14	1	0.99	0.47	0.27	3.00E-04	6.60E-03
				IDEpi	0.87	0.85					0.15	0.2		
CD4bs	VRC07-523-LS	0.96	0.95	AllSig	0.96	0.94	0.5	0.38	0.98	0.97	0.52	0.34	4.00E-05	3.30E-03
				Contact	0.97	**0.96**	0.38	0.38	0.99	0.99	0.52	**0.45**	1.80E-04	6.30E-04
				mRMR	0.97	0.95	0.38	0.25	1	0.99	**0.6**	0.33	4.60E-05	1.10E-02
				IDEpi	**0.98**	**0.96**					0.4	**0.45**		
MPER	10E8	0.98	0.98	AllSig	0.98	0.97	0.4	0.25	1	0.98	0.51	0.23	1.40E-03	9.00E-02
				Contact	**0.99**	**0.98**	0.6	0.25	1	0.99	**0.66**	**0.34**	2.70E-05	4.60E-02
				mRMR	0.97	**0.98**	0	0	0.99	1	0.02	0	1	1
				IDEpi	0.88	0.94					0.04	0.28		
MPER	4E10	0.98	0.93	AllSig	**0.98**	0.92	0.25	0	1	0.99	**0.34**	-0.02	3.80E-02	1
				Contact	**0.98**	0.93	0.25	0	1	1	**0.34**	0	3.80E-02	1
				mRMR	**0.98**	**0.94**	0	0.08	1	1	0	**0.28**	1	6.90E-02
				IDEpi	0.94	0.92					0	-0.02		

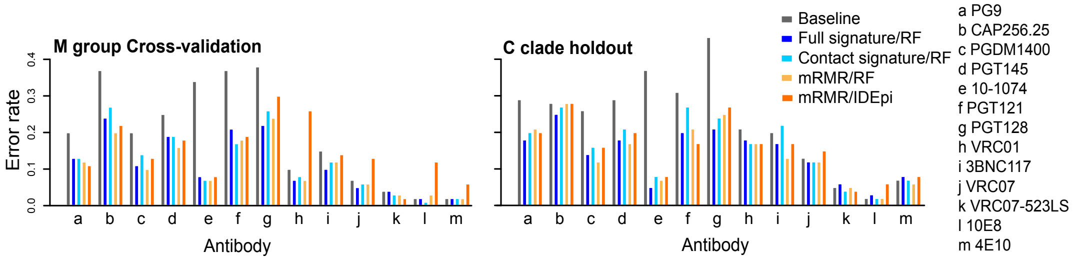

Table S5. Machine learning classification prediction statistics, comparing strategies for input feature selection, supporting Figure 4 in the main text. (A) Random Forest predictions of positive/negative IC_{50} values (above or below the threshold of detection at the highest concentration of antibody used). The global M group dataset 4 was evaluated for accuracy of predictions using leave-one-out cross-validation (the "Xval" columns). Then the M group data was used as a training set and the accuracy of predictions evaluated using the holdout set from C clade (the "Hout" columns). The fraction of true positives for each antibody in the M group data and the C clade hold out set is provided. It is considered the null or "default" model; for example, if 63% of Envs were sensitive to an antibody, and you guessed a new set of Envs was all sensitive, by "default" you would be correct 63% of the time. We also present the Matthews Correlation Coefficient (MCC) (ranging from 0 to 1, 0 being a no better than random, 1 a perfect prediction), and a Fisher's exact p-value, the level of significance indicating if the prediction is better than a default prediction based on true positives. As an example, for 10-1074, 63% of the C clade viruses are sensitive and the full signature machine learning predictions called 95% of these correctly as positive/negative. This high accuracy gave an MCC of 0.90 and a very low p-value. In contrast, for an antibody like 10E8, almost all Envs are sensitive to the antibody (98%), so the accuracy of the prediction is very high (97%), but this is essentially no better than just guessing all Envs are sensitive, so the MCC is 0.23 and the p-value it not significant. We also provide a summary of the accuracy of the positive calls and the negative calls independently; we have a low frequency of false positives and we have a higher frequency of false negatives. This is an expected outcome as the antibodies studied here are broadly reactive, i.e. there are many more positive than negative pseudoviruses for each antibody studied and the RF is optimized to achieve overall accuracy. It may be possible to tune this outcome by weighting the classes differently (e.g., with more weight on the negative class to improve the accuracy of the negative calls), if this was important for future studies, but this would come at a cost of lower overall accuracy.

As with regression, we varied the input filters using the same RF machine learning strategy. "AllSig" indicates all signatures were used in the pre-filter, "Contact" indicates only contact signatures were used, and "mRMR" indicates a standard mRMR pre-filter was used. We also ran IDEpi on our data to compare our results using mRMR to ensure we were getting reasonable results relative to the existing literature (Hepler et al., 2014) . We evaluated leave-10-out and leave-50-out using IDEpi; we present leave-50-out, as this was most predictive. The bold values indicate the best score for each antibody. Our implementation of mRMR gave scores that were generally comparable to IDEpi. The overall accuracies and MCC scores were generally roughly comparable, and unlike regression predictions, no particular input filter was favored. The features from the alignments that had the highest importance are listed in the footnote, see Table S4 above for the key.

***CLASSIFICATION footnotes:** organized following the footnotes in Table S8:

CAP256-VRC26.25	3/3	R315**Q** V169**K** R166**R**
PG9	10/11	O160**O** t138d K171**K** -353b**-** -132k**-** F717**F** -787b**S** h330y N677**N** s440q
PGDM1400	9/9	O160**O** v169e -787e**G** l619l q170- s668s m426r s440q -787d**K**
PGT145	10/96	O160**O** G167**D** g167g s446i r166g 189gn v169e t402w 132f**-** 031cn
10-1074	1/1	O332**O**
PGT121	10/92	O332**O** s334o n325k N325**D** n340r e620n S334**S** 363ap O301**O** V255**V**
PGT128	10/18	O332**O** O295**O** i320- T297**T** O301**O** H330**H** I165**I** q442e R304**R** k151q
3BNC117	2/2	G459**G** R456**R**
VRC01	1/1	R456**R**
VRC07	10/69	R456**R** G459**G** g471i -132qd N280**N** r304k 363b**-** N355- q352i -787bi
VRC07-523-LS	3/3	r456s g471i G459**G**
10E8	1/1	n671t
4E10	1/1	f673l
2F5	3/3	K665**K** A667**A** E662**E**

Underneath the table is an illustration of the error based on the data in the table. For all bNAbs, frequencies of errors for classification predictions are shown. The grey bar on the left of each set is the baseline null model that predicts everyone is positive, and the error is then just the frequency of viruses that were actually negative. Different machine learning strategies are compared for their impact on classification accuracy for calling Env sensitivity to a given antibody – the lower the bar, the more accurate the predictor. The 3 different input filters we tried were generally better than baseline (lower than the grey bar), and similar to IDEpi predictions, with a few exceptions. Machine learning classification predictions for V3 glycan antibodies may be particularly helpful. For bNAbs that have almost complete breadth naturally (e.g. VRC07-523-LS, 10E8, and 4E10), the simple baseline of "assuming positive" performs extremely well and machine learning predictions do not add much value. The 13 antibodies included in this figure were those that had data available in both the training set (data set 4) and the holdout set (data set 3).

Table S6. Machine learning informative feature contributions. Supporting Fig. 4. This table presents ranked signature features that were among the top 10 most informative sites for different antibodies of different classes. It has four sections, one for each antibody class, and each section lists the most important signature features for Classification and Regression predictions for each antibody tested from dataset 4. On the right are summaries of the number of times recurrent features are found. Different shades highlight different V loop characteristics. Bold indicates sites that were repeatedly informative for a number of antibodies (at least 4/11 times for CD4bs bNAbs, 3/6 times for V2 bNAbs, 3/6 times for V3 bNAbs, and 2/3 for MPER bNAbs). Blue indicates signature positions within epitope contact regions, black are positions outside the contact region. NxST N332 and NxST N160 indicate a PNGS. Clades were only rarely among the most informative data for prediction.

A

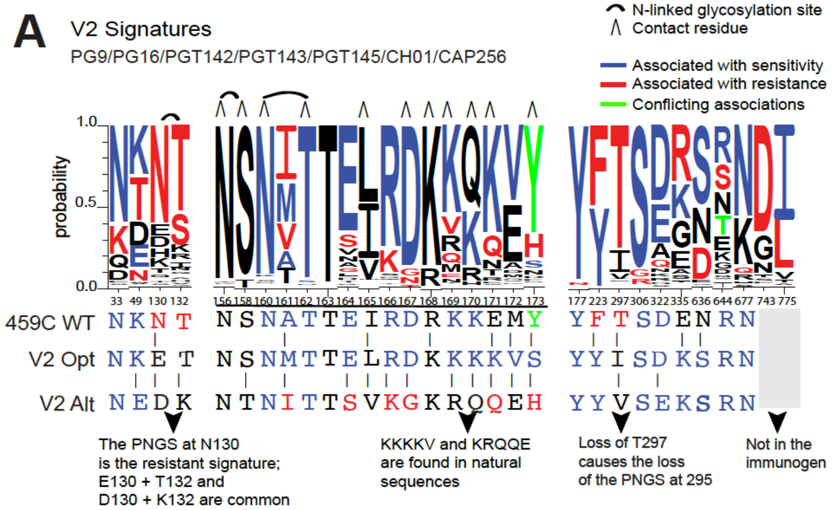

B

```
Sequence Name     Sequence Alignment                                                          End Position

HXB2 position                      49
C.459             GNLWVTVYYGVPVWREAKTTLFCASDAKAYDREVHNVWATHACVPTDPNPQEIVLENVTENFNMWKNDMVDQ   72
V2glycan.opt      ------------------------------------------------------------------------   72
V2glycan.alt      -----------------E------------------------------------------------------   72

HXB2 position                               130                                     160
C.459             MHEDIISLWDQSLKPCVKLTPLCVTLNCTNVTSSAANVTSNVTNDANNASNANGRNVINEDMQNCSFNATTE  144
V2glycan.opt      --------------------------E--AFNSSSHTNSSIAMQEMK................N----M---  128
V2glycan.alt      --------------------------D-KAFNSSSHTNSSIAMQEMK................N-T--I--S  128
CRF02.T250_4                                DCQAFNSSSHTNSSIAMQEMK ---------------NCSFNVTTE

HXB2 position           169                   190                              223
C.459             IRDRKKEMYALFYKLDIVPLDGEKSDNRYRLINCNTSTLTQACPKVSFDPIPIHYCTPAGFAILKCNNKTFN  216
V2glycan.opt      L--K--KVS-----------..NKNGRQ--------------------------------Y-----------  198
V2glycan.alt      VKGKRQQEH-----------..NKNGRQ--------------------------------Y-----------  198
CRF02.T250_4      LRDKKKKEYSFFYKTDIEQI..NKNGRQ

HXB2 position                                                                    297
C.459             GTGPCNNVSTVQCTHGIKPVVSTQLLLNGSLAEEDIIIRSENLTNNAKTIIVHLNESVEIVCTRPNNNTRKS  288
V2glycan.opt      --------------------------------------------------------------I---------  270
V2glycan.alt      --------------------------------------------------------------V---------  270

HXB2 position                  321           335
C.459             IRIGPGQTFYANNDIIGDIRQAHCNISEEKWNNTLHRVWKKLVEHFPNKTTIRFDRHSGGDLEITTHSFNCG  360
V2glycan.opt      ---------------------------K--------------------------------------------  342
V2glycan.alt      -------------E-------------K--------------------------------------------  342

C.459             GEFFYCNTSGLFNITYNSNYTYNDTKHNGTKVITLPCRIKQIINMWQEVGRAMYAPPIAGNITCTSNITGLL  432
V2glycan.opt      ------------------------------------------------------------------------  414
V2glycan.alt      ------------------------------------------------------------------------  414

C.459             LTRDGGNNSTETETFRPGGGDMRDNWRSELYKYKVVEIKPLGIAPTGAKRRVVEREKRAVGIGAVFLGFLGA  504
V2glycan.opt      ------------------------------------------------------------------------  486
V2glycan.alt      ------------------------------------------------------------------------  486

C.459             AGSTMGAASITLTVQARQLLSGIVQQQSNLLKAIEAQQHLLQLTVWGIKQLQTRVLAIERYLKDQQLLGLWG  576
V2glycan.opt      ------------------------------------------------------------------------  558
V2glycan.alt      ------------------------------------------------------------------------  558

HXB2 position                                            636
C.459             CSAKLICTTAVPWNSSWSNKSETEIWNNMTWMQWDREINNYTNTIYRLLEESQNQQEKNENDLLALDKWNSL  648
V2glycan.opt      --------------------------------------S---------------------------------  630
V2glycan.alt      --------------------------------------S---------------------------------  630

C.459             WDWFGISNWLWYIR                                                            662
V2glycan.opt      --------------                                                            644
V2glycan.alt      --------------                                                            644
```

Table S7. Signature vaccine design based on dataset 1 and dataset 2, providing background detail in support of Figure 5. (A) The modifications made to 459C WT to create the trivalent vaccine designs for V2 Opt and V2 Alt immunogens. The signatures used for vaccine design were generated at a time when only datasets 1 and 2 were available and the antibodies informing the signature at that time are listed at the top. The contact region for each class of antibody is underlined and contact residues and PNGS are highlighted (see the key, top right). Red amino acids were associated with resistance, blue with sensitivity, green with either sensitivity or resistance depending on which antibody was assessed in the set, and black had no statistical support for an association. The size of each letter in the LOGO corresponds to the frequency of that AA in the M group panel data and we attempted to capture common AAs in signature sites at a global level. In both Figures A and B, 459C WT is shown under the LOGO in the same signature positions, AAs are colored in blue, red, green, or black according to their neutralization associations at the population level, and changes made in the Opt or Alt designs are indicated by vertical lines. Comments related to decisions about specific modifications are included; in general, we tried to represent relative diversity within the epitope (the underlined region), including AAs associated with resistance, favoring more common AAs, and grouping neighboring AAs to yield common motifs. Outside of the epitope we favored sensitivity, but if this could be achieved with inclusion of distinct variants that were either both sensitive or neutral signatures, we included the variant in the Alt versions to better represent natural HIV-1 diversity. **(B) Precise modifications introduced into the V2 SET vaccine mapped on to protein sequences.** 459C WT (GenBank accession JN681242) baseline gp140 protein sequence, with modifications introduced based on part (A). The CRF02.T250_4 (GenBank accession EU513189) V1 and V2 regions replaced those of 459C WT and these regions are shown in green. CRF02.T250_4 was exquisitely sensitive to both V2 and V3 bNAbs and had desirable loop characteristics for both classes of antibodies (though it was resistant to CD4bs bNAbs) (Fig. S1). Our intent was to enhance V2 responses, but thought that V3 epitope accessibility in the SET Alt and Opt designs might also be improved by using a hybrid of 459C WT with CRF02.T250_4 V1 and V2 regions. The periods (.) indicate gaps to maintain the alignment; T250 is shorter in these regions than 459C. The dashes (-) indicate identity with 459C WT. Key HXB2 position numbers are indicated above the alignment.
